# Aerobic Visible-Light-Driven Borylation of Heteroarenes in a Gel Nanoreactor

**DOI:** 10.1021/acs.orglett.1c00451

**Published:** 2021-03-02

**Authors:** Jorge C. Herrera-Luna, David Díaz Díaz, Alex Abramov, Susana Encinas, M. Consuelo Jiménez, Raúl Pérez-Ruiz

**Affiliations:** † Departamento de Química, Universitat Politècnica de València (UPV), Camino de Vera S/N, 46022, Valencia, Spain; ‡ Departamento de Química Orgánica, Universidad de La Laguna, Avda. Astrofísico Francisco Sánchez 3, 38206, La Laguna, Spain; § Instituto de Bio-Orgánica Antonio González, Universidad de La Laguna, Avda. Astrofísico Francisco Sánchez 3, 38206, La Laguna, Spain; ∥ Institut für Organische Chemie, Universität Regensburg, Universitätsstr. 31, 93053, Regensburg, Germany

## Abstract

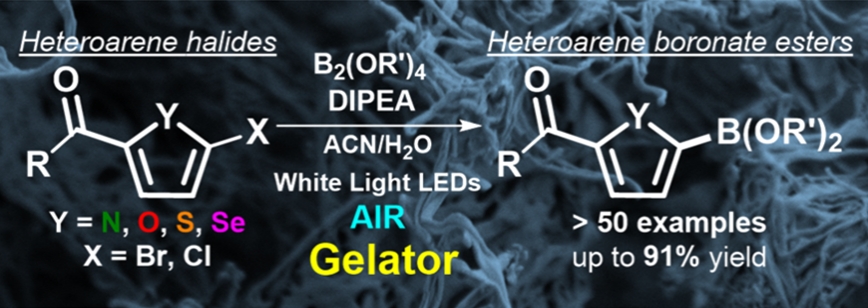

Heteroarene boronate esters constitute valuable intermediates in modern organic synthesis. As building blocks, they can be further applied to the synthesis of new materials, since they can be easily transformed into any other functional group. Efforts toward novel and efficient strategies for their preparation are clearly desirable. Here, we have achieved the borylation of commercially available heteroarene halides under very mild conditions in an easy-to-use gel nanoreactor. Its use of visible light as the energy source at room temperature in photocatalyst-free and aerobic conditions makes this protocol very attractive. The gel network provides an adequate stabilizing microenvironment to support wide substrate scope, including furan, thiophene, selenophene, and pyrrole boronate esters.

Organoboron-containing molecules continue to attract considerable interest from scientists that seek new synthetic approaches since the reactivity of these entities is broad.^1^ Their incorporation in appropriate cores by combination of a multitude of methods and their ability to continually expand by converting carbon–boron bonds into nearly any other functional group makes organoboronates a key functional group in modern organic synthesis, material science, and drug discovery.^2^ The importance of these molecules is further enhanced by their capacity to undergo stereospecific transformations, generating an extensive range of enantioenriched building blocks for synthesis.^3^ In this decade, we have witnessed notable developments in numerous strategies using either transition metal catalysis^4^ or noncatalytic methods^5^ for the synthesis of organoboron derivatives and their subsequent assemblage.

Aryl halides are frequently employed as precursors of aryl boronate esters due to their widespread and cheap availability in the market. Among methods for thermally induced borylation of aryl halides by transition metals,^6−11^ photocatalysis has emerged as a powerful tool for the construction of aryl boronate esters.^12^ For instance, procedures using UV-light have allowed the borylation of aryl halides, mesitylates, and ammonium salts;^13^ however, the use of high-intensity UV photolysis could form undesired products, limiting the technique’s applicability. In terms of selectivity, visible-light-driven processes are considered a superior strategy to generate aryl boronates from aryl halides, and many examples using metal or metal-free photocatalyst systems have been reported.^14^

In this vein, we have recently contributed to this field reporting a novel, straightforward, and rapid protocol to produce boron-containing thiophenes from thiophene halides, employing visible light under mild conditions.^15^ The merits of this methodology mainly reside in the absence of any external photocatalyst system together with a drastic shortening in irradiation times (0.5–2 h). However, an anaerobic atmosphere is crucial since there is no such reaction in the presence of oxygen.

To circumvent this drawback, we envision employing viscoelastic supramolecular gels, often made of low-molecular-weight (LMW) compounds self-assembled through noncovalent interactions as compartmentalized reaction media.^16^ Although many studies utilizing viscoelastic gels as reaction vessels and/or nanoreactors for other type of processes have been reported,^16^ some examples of photochemical reactions in gel media can be found in literature.^17^

Indeed, the reactivity to air-sensitive photochemical transformations has demonstrated that such gel networks provide a suitable stabilizing microenvironment under aerobic conditions.^17b−17d^

Accomplishing the borylation of heteroarene halides under milder conditions, including photocatalyst-free, visible-light irradiation at room temperature under an aerobic atmosphere, appears challenging. Here, we have explored this option using physical gels as confined reaction media. Our results show the feasibility of the procedure, expanding the scope of the borylated reactions not only to thiophene halides but also to furan, pyrrole, and selenophene halides. Thus, application of this method may be extended beyond borylations to prepare bioactive molecules (Figure 1A,B).

**Figure 1:**
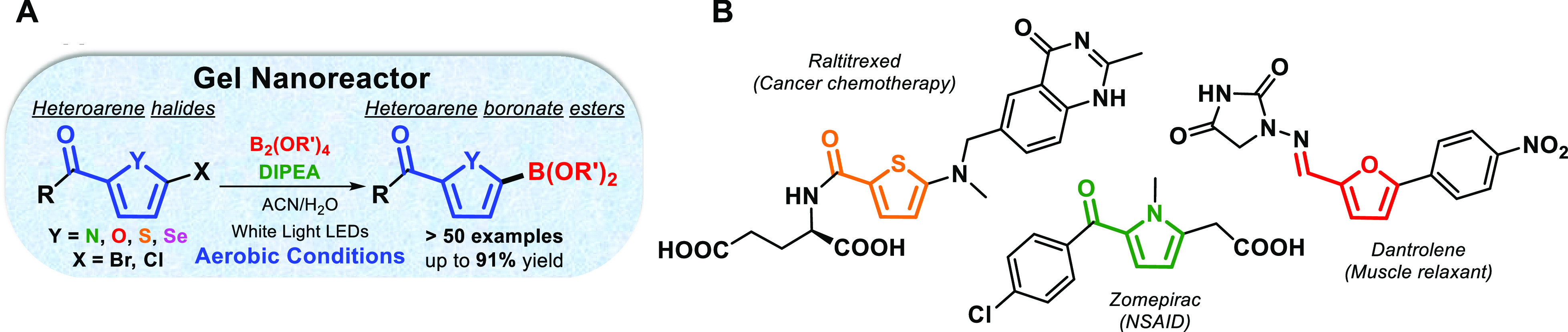
(A) Visible light-driven borylation of heteroarenes in gel media under air conditions. (B) Examples of pharmaceutical agents containing thiophene, furan, and pyrrole moieties.

Based on our previous work,^15^ we first screened the photolysis of 2-acetyl-5-chlorothiophene (**1a**) with bis(pinacolato)diboron (B_2_pin_2_) and *N,N*-diisopropylethylamine (DIPEA, Hünig’s base) in aerated MeCN/H_2_O (9/1 v/v) solution. The expected borylated thiophene **2aa** was not observed (Table 1, entry 1), confirming that the reaction was completely blocked by the dissolved molecular oxygen, presumably shifting the reaction mechanism to other unwanted pathways (*vide infra*). Conversely, the desired product **2aa** was formed in high yields when the physical gel formed by **G1** (*N,N′*-bis(octadecyl)-l-boc-glutamic diamide, molecular structure in Table 1)^18^ was used as a confined medium under otherwise identical conditions (Table 1, entry 3; the balance of conversion was the dehalogenated product). Optimal conditions involved lower reagent loading than reported elsewhere^15^ (10 equiv of B_2_pin_2_ and 1.2 equiv of DIPEA), with irradiation in the visible range at 410–700 nm with cold-white LEDs in **G1** medium for 2 h under aerobic conditions. The result within the aerobic gel phase was gratifyingly comparable to that obtained in solution in a strict inert atmosphere (Table 1, entry 2). The model reaction was also carried out under an oxygen-free atmosphere instead of aerobic conditions (Table 1, entries 4 versus 3), yielding a similar amount of **2aa**. This outcome reveals that the gel network offers an efficient confinement effect for visible-light-induced radical reactions in air.

**Table 1:**
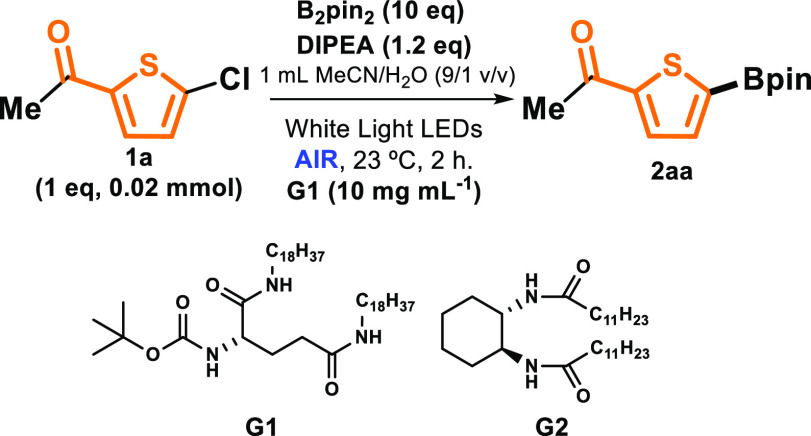
Optimization of Reaction Conditions^a^

Entry	Deviations for the conditions shown	Yield (%)^b^
1	without **G1**	0
2	purged N_2_/without **G1**	56 (75)^c^
3	–	72 (100)
4	purged N_2_	60 (73)
5	0.04 mmol of **1a**	64 (100)
6	5 equiv of B_2_pin_2_	57 (92)
7	1 equiv of DIPEA	35 (46)
8	no DIPEA, or dark reaction	0 each
9	**G1**^d^ (8 mg mL^–1^)	43 (70)
10	**G1**^d^ (15 mg mL^–1^)	37 (59)
11	**G2**^d^ (10 mg mL^–1^)	56 (86)

a Optimal conditions.

b GC-FID yields of **2aa** (**1a** conversion in parentheses) using internal 1-dodecanonitrile. Estimated error from randomly duplicated experiments independently ±3% (see Supporting Information (SI)).

c 3 h irradiation.

d Gelator self-assembly process in organic solvents is driven by hydrogen bonds and van der Waals forces, leading to tangled fibrillar nanostructures over a wide concentration range (2–21 g L^–1^ and 2–44 g L^–1^ for **G1** and **G2**, respectively).^18,19^

Varying the amount of each reactant did result in lower yields of **2aa**, although full conversion of **1a** was observed in some cases (Table 1, entries 5–7). The absence of DIPEA or light in control experiments confirmed the key role of these elements in the chemical transformation (Table 1, entry 8). Additionally, employment of other bases did not offer better yields (see Table S1 in SI).

This visible-light-driven thiophene borylation thus improved considerably due to the gel network, which permitted the process to occur in air. Full conversion of **1a** and maximized **2aa** yield were obtained with an optimal concentration of **G1** (10 mg mL^–1^); reductions in yield were observed at **G1** concentrations below 10 mg mL^–1^ (Table 1, entry 9). Perhaps the oxygen diffusion rate through the gel phase is faster, leading to the process being partially blocked. The diffusion of reactants might decrease inside the solvent pools above the optimal **G1** concentration, also reducing yield (Table 1, entry 10). Moreover, the effect of light scattering could be minimized by adjusting the solvent volume (see Table S1, entries 11, 13, and 14). Thus, the lower the volume the higher the process efficiency, i.e., 72% yield (1 mL), 64% yield (2 mL), and 53% yield (4 mL).

To check whether this reaction may be associated specifically with gelator **G1**, the model reaction was performed in the gel of **G2** (*N,N′*-((1*S*,2*S*)-cyclohexane-1,2-diyl)didodecanamide,^19^ molecular structure in Table 1), which assembles with a different matrix. A 56% yield of **2aa** was produced under optimal conditions (Table 1, entry 11); therefore, **G2** also offered a suitable microenvironment for the investigated reaction. Note that the gelator can be easily separated by filtration and reused in subsequent experiments without detriment to its gelation properties (see SI).

The standardized conditions (Table 1, entry 3) were next applied to a diverse set of heteroarene halides and various diboron derivatives (Scheme 1). First, upon variation of both starting materials, thiophene boronate esters (**2aa**―**2fd**) were obtained in moderate-to-high yields (23–89%); these are important scaffolds in pharmaceuticals^20^ and conjugated materials,^21^ alongside other applications. The reactivity was generally similar in all cases, except for thiophene halides bearing the −COOMe group, which presented lower conversions and yields (**2ca**―**cd**). To rule out that this reaction was specifically for thiophenes and the involvement of the sulfur atom in the radical process, the generality and the versatility of this protocol were explored using different haloheterocycles such as furan, pyrrole, and selenophene halides. All were submitted to visible light irradiation in the presence of DIPEA and various diboron derivatives, employing **G1** under air. After an easy procedure for recovering the products (see details in SI), the results indicated that borylation of the corresponding heteroarenes succeeded, with gratifyingly high yields in some cases (for instance, 85% for **3ab** or 91% for **4aa** or 88% for **5ac**).

**Scheme 1:**
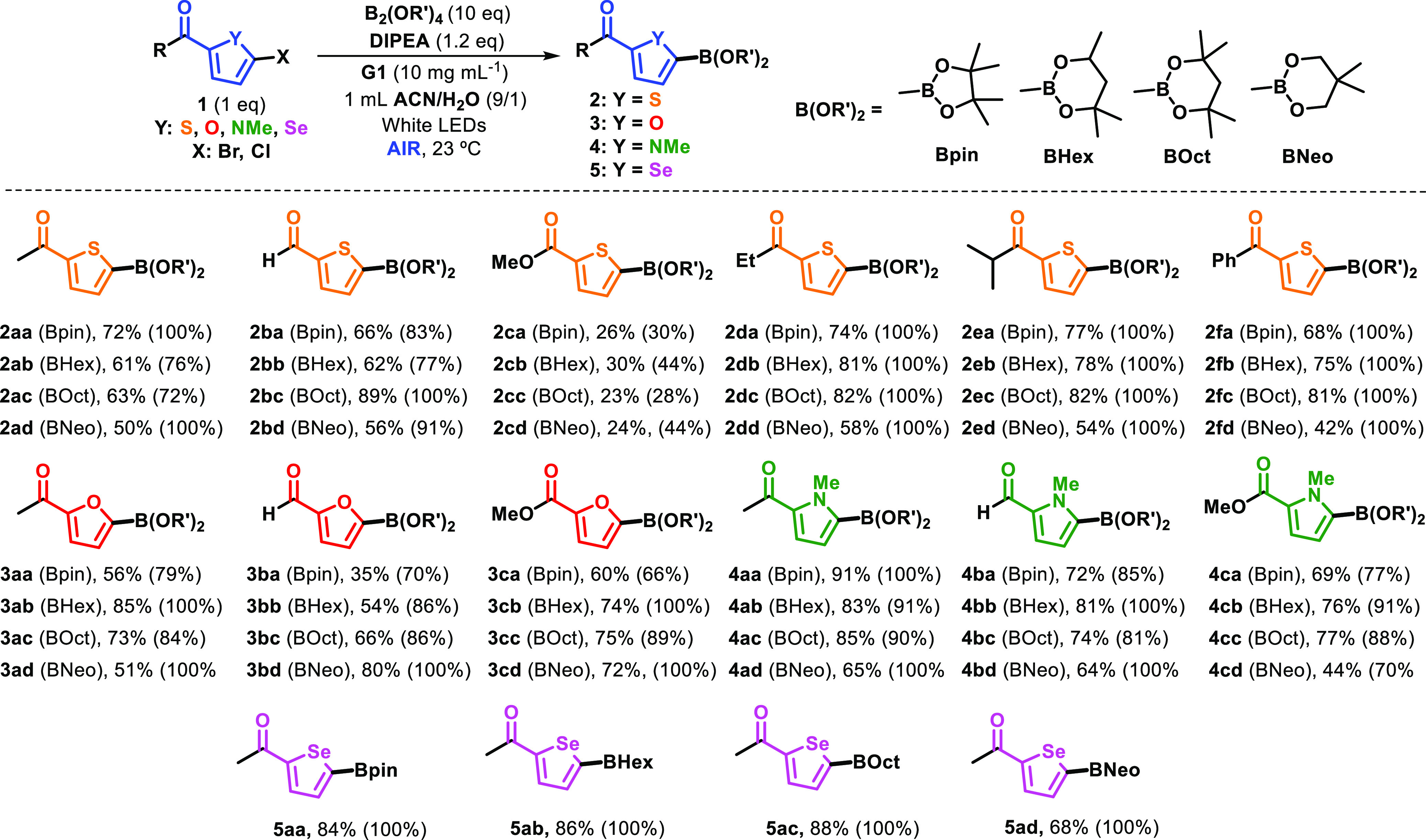
Coupling of Heteroarene Halides with Diboron Derivatives (Conversion of **1** in Parentheses)

To highlight that this photochemical reaction represents a useful method for organic synthesis, the model reaction was carried out as follows: (i) in a higher scale moving from 0.02 to 1 mmol, obtaining a 63% yield of **2aa** (see details in SI) and (ii) under outdoor sunlight after 4 h, leading to the formation of the desired product in 66% yield (see all details in SI). Further, it provides access to a vast number of new borylated derivatives (more than 50 examples) with low toxicity, and presumably suitable reactivity to be employed as versatile precursors in organic reactions such as Suzuki–Miyaura^22^ and Chan–Lam^23^ coupling reactions.

The role of the viscoelastic gel network as an effective nanoreactor was supported by a combination of experimental measurements. First, kinetic studies of the model reaction revealed that conversion of starting material **1a** was faster in aerated gel medium than in inert solution for the same irradiation time (Figure S1). This was well-correlated with the formation of **2aa**, where yields were found to be higher in gel (Figure S2). Interestingly, production of **2aa** was negligible from a frozen (193 K) aerated MeCN/H_2_O solution of the model reaction due to, as expected, restricted molecular diffusion; conversely, a 30% yield of **2aa** was obtained under the same conditions in the presence of **G1** (details in SI). This could be interpreted as meaning reactants are not only localized in the solvent pools between fibers, but may also spread through fibers, permitting photochemical reaction in a confined by dynamic space. In addition, field-emission scanning electron microscopy (FESEM) images were used to show that inclusion of the reactants within the supramolecular gel provoked a slight densification of the network, while its morphological features were preserved after irradiation (Figure 2 and Figure S3).

**Figure 2:**
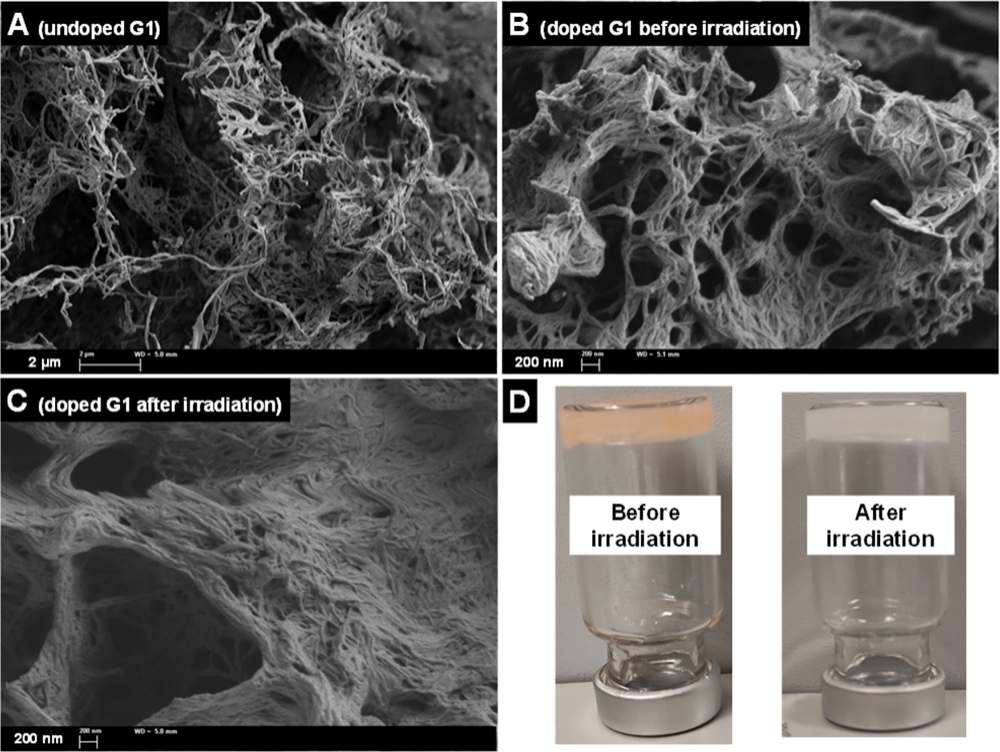
Representative field-emission scanning electron microscopy (FESEM) images: **A**: undoped gel of **G1** (10 mg mL^–1^) in 1 mL MeCN/H_2_O (9/1 v/v); **B, C**: gel of **G1** (10 mg mL^–1^) doped with **1a** (3.2 mg), B_2_pin_2_ (50 mg) and DIPEA (3.1 mg) in 1 mL MeCN/H_2_O (9/1 v/v); **D**: Photograph of the doped gel with 5-bromo-2-furaldehyde+B_2_pin_2_+DIPEA at standard conditions before/after irradiation.

Such densification could be interpretated by partial incorporation of reactants into the fibers that, gratifyingly, did not affect the thermal stability of the gel network, as supported by the same gel-to-sol transition temperature (*T*_gel_) observed for both the undoped gel made of **G1** and the doped gel (i.e., as described in Figure 2) even after irradiation (*T*_gel_ = 50 ± 2 °C).^24^ Visual inspection of the materials after the irradiation experiments suggested no change in the viscoelastic properties of the gels (i.e., no gravitational flow; see Figure 2D).

Based on literature,^15^ the proposed reaction mechanism is outlined in Scheme 2. Complex **A** was formed at the ground state in all cases as confirmed by UV–vis spectrophotometry (Figure S4); a marked absorbance in the visible region was observed, permitting initiation of the photoreaction and generation of the corresponding excited states (A*). Under aerobic conditions in solution, this species could be efficiently quenched by molecular oxygen through energy transfer (EnT), giving rise to the starting materials and singlet oxygen (^1^O_2_). Electron transfer (ET) from ^1^O_2_ to DIPEA would occur,^25^ and the resulting oxygen radical anion (O_2_^•–^) might abstract a H from the DIPEA radical cation (DIPEA^•+^), forming an aminoalkyl radical which would react with the diboron derivative to produce the corresponding byproduct. Not even traces of the desired heteroarene boronate ester were detected in the crude, supporting an oxygen-locked process. Besides, spectroscopic measurements provided evidence of ^1^O_2_ reactivity, with its lifetime dramatically decreased under the ideal reaction conditions (Figure S5). The scenario differed when supramolecular gels were used as confined media. The oxygen diffusion was negligible in this case, and the reaction proceeded following the mechanism we previously published.^15^ Indeed, trapping experiments using diphenyldisulfide (PhSSPh) confirmed the involvement of the heteroarene radical as an intermediate (see SI for details).

**Scheme 2:**
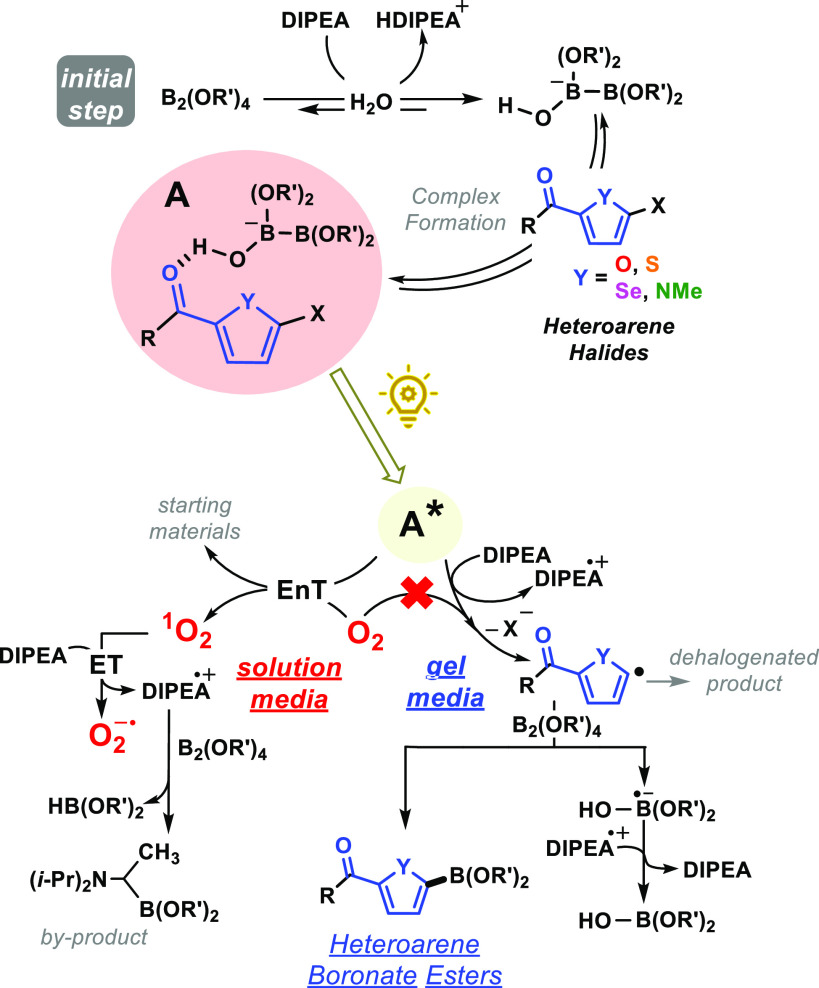
Proposed Reaction Mechanism

In conclusion, we report an attractive and efficient methodology for building heteroarene boronate esters under very mild conditions. A simple operation that requires only commercially available reagents, visible light, room temperature, and ambient pressure and proceeds photocatalyst-free and under an aerobic atmosphere has been effectively employed in an LMW gel nanoreactor. A wide variety of products have been obtained that may act as versatile precursors for further synthetic work. The use of supramolecular viscoelastic gels has allowed us not only to protect against oxygen poisoning but also to accelerate the reaction relative to standard conditions.

## Supporting Information Available

Materials and methods, general procedures, optimization of reaction conditions, microscopy measurements, singlet oxygen experiments, GC chromatograms, characterization of products, and spectroscopical data of all compounds (PDF)
