# Supplementary material for: Aerobic Visible-Light-Driven Borylation of Heteroarenes in a Gel Nanoreactor
Source: Org Lett. 2021 Mar 2;23(6):2320–5. doi: 10.1021/acs.orglett.1c00451 (PMC8719754; doi:10.1021/acs.orglett.1c00451)

Supplementary information

Aerobic Visible Light-Driven Borylation of Heteroarenes in a Gel Nanoreactor

Jorge C. Herrera-Luna,[a] David Díaz Díaz,[b,c,d] Alex Abramov,[d] Susana Encinas,[a] M. Consuelo Jiménez,*[a] and Raúl Pérez-Ruiz*[a]

[a] Departamento de Química, Universitat Politècnica de València (UPV), Camino de Vera S/N, 46022, Valencia, Spain.

[b] Departamento de Química Orgánica, Universidad de La Laguna, Avda. Astrofísico Francisco Sánchez 3, 38206, La Laguna, Spain.

[c] Instituto de Bio-Orgánica Antonio González, Universidad de La Laguna, Avda. Astrofísico Francisco Sánchez 3, 38206, La Laguna, Spain.

[d] Institut für Organische Chemie, Universität Regensburg, Universitätsstr. 31, 93053, Regensburg, Germany.

Table of contents

- **Materials and methods** **S2**
- **General procedure** **S3**
- **Synthesis of LMW Gelators** **S4**
- **Searching the optimal conditions** **S5**
- **Kinetic studies** **S6**
- **Frozen model reaction procedure** **S7**
- **Field-emission scanning electron microscopy (FESEM)** **S8**
- **Determination of gel-to-sol transition temperature (T_{gel})** **S10**
- **UV-vis absorption spectra** **S11**
- **Singlet oxygen** **S12**
- **GCMS byproduct** **S13**
- **Trapping reaction** **S14**
- **Sunlight irradiation and 1 mmol scale** **S15**
- **GC Chromatograms** **S16**
- **Characterization of compounds** **S27**
- **NMR spectra** **S45**

Materials and methods

All reagents (≥97% purity) and solvents (≥99% purity) were purchased from commercial suppliers (Merck, TCI, Apollo Scientific, Fluorochem, Scharlab) and used as received unless otherwise indicated. Reactions were carried out in Metria®-Crimp Headspace clear vial flat bottom (10 mL, Ø 20 mm) sealed with Metria®-aluminium crimp cap with moulded septum butyl/natural PTFE (Ø 20 mm). Irradiation was performed with a cool white LED (LED Cree MK-R, cold-white, 11.6 V, 700 mA, P = 8.5 W). TLC was performed on commercial SiO_2-coated aluminium and plastic sheets (DC60 F254, Merck). Visualization was done by UV-light (254nm). Product were isolated materials after TLC on silica gel (Merck, mesh 35-70, 60 Å pore size) and their corresponding yields were determined by quantitative GC-FID measurements on an Agilent 8860 GC-System with N_2 as carrier gas. Dodecanenitrile was used as an internal standard in the GC-FID quantitative measurements; yield products were estimated as: [conversion × selectivity]/mass balance. Determination of purity and structure confirmation of the literature known products was performed by ^1H NMR, ^{13}C NMR and high-resolution mass spectrometry (HRMS) in case of unknown products. NMR spectral data were collected on a Bruker Advance 400 (400 MHz for ^1H and 101 MHz for ^{13}C) spectrometer at 20 °C. Chemical shifts are reported in δ/ppm, coupling constants J are given in Hertz. Solvent residual peaks were used as internal standard for all NMR measurements. The quantification of ^1H cores was obtained from integrations of appropriate resonance signals. Abbreviations used in NMR spectra: s – singlet, d – doublet, t – triplet, q – quartet, m – multiplet, bs – broad singlet, dd – doublet of doublet, ddd – doublet of doublet of doublet. HRMS was carried out was performed in the mass facility of SCSIE University of Valencia. LRMS was carried out on an HP 6890 Series GC System with Agilent 5973 Network Mass Selective Detector and H_2 as carrier gas.

General procedure

A vial (10 mL) was charged with the hetereoarene derivative (20 µmol, 1.0 eq.), diborylates (200 µmol, 10.0 eq.) and the correspondent gelator (G1, 10 mg/mL). Acetonitrile/H_2O (9/1 v/v) mixture (1.0 mL) was poured. Then, DIPEA (24 µmol, 1.2 eq.) and dodecanenitrile (20 µmol, 1.0 eq.) were added with 10 µL Hamilton syringe. Quickly, the vial was sealed with a septum. It was heated to 150 °C for 1.5 minutes with manual stirring until complete clear solution. The vial cooled to room temperature until gel formation was observed. The reaction was irradiated with an external LED through the plain bottom side of the vial at 23 °C during the corresponding time. Then, brine (2 mL) was added, and the aqueous phase was extracted with dichloromethane (1 mL). The reaction was monitored by GC-FID analysis. The organic phase was dried over anhydrous sodium sulfate, filtered from the drying agent, and concentrated in vacuo. After recrystallization of the excess of starting diboron derivative, which is recovered, the crude was purified via TLC plastic sheet using a hexane/ethyl acetate mixture as the mobile phase. Note: The gelator can be easily separated by filtration and reused in subsequent experiments without any detriment of its gelation properties.

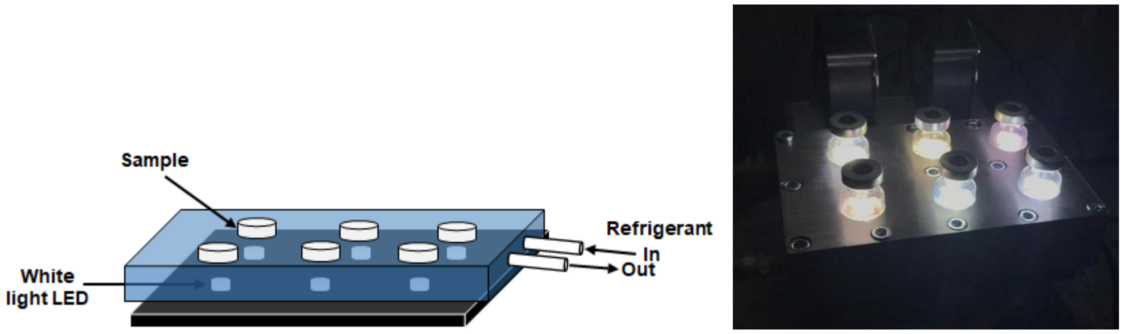

Irradiation setup

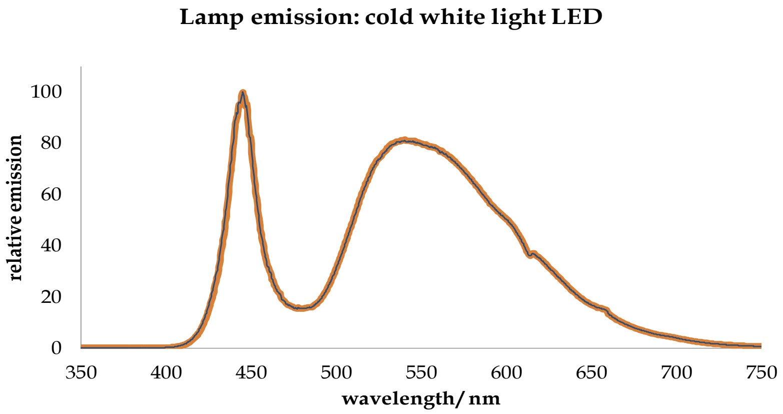

Synthesis of Low Molecular Weight Gelators (LMW Gelators)

*N,N'-Bis(octadecyl)-L-Boc-glutamic Diamide (**G1**).*

Boc-glutamic acid (0.01 mol, 1.0 equiv) and octadecylamine (0.02 mol, 2.0 equiv) in dichloromethane (200 mL) were mixed. Then, 1-ethyl-3-(3-(dimethylamino)propyl)carbodiimide hydrochloride (EDC·HCl) (0.022 mol, 2.2 equiv) was added to the mixture and stirred at RT for 72 h. The obtained white solid was isolated by filtration and washed three times with dichloromethane. The crude product was dissolved in THF and precipitated by water. A fine white solid was obtained (80%). Following the procedure reported previously *Soft Matter* **2007**, *3*, 1312−1317.

^{1}H NMR (300 MHz $CDCl_3$) δ 6.69 (br, 1H), 6.32 (br, 1H), 5.77 (br, 1H), 4.08 (br, 1H), 3.26−3.22 (m, 4H), 2.41−2.27 (m, 2H), 2.06−1.93 (m, 2H), 1.58−1.46 (m, 4H), 1.43 (s, 9H), 1.25 (s, 60H), 0.87 (t, 6H) ppm.

(S,S)-Dodecyl-3-[2(3-dodecyl-ureido)cyclohexyl]urea (**G2**).

A solution of dodecylisocyanate (15 mmol, 2.0 equiv) in toluene (20 mL) was slowly added to a solution of (*S,S*)-1,2-cyclohexyldiamine (7 mmol, 1.0 equiv) in toluene (100 mL). The reaction mixture was stirred for 16 h at RT and 2 h at 100 °C. After cooling to RT, the gel-like reaction mixture was filtered to give a white waxy solid. The waxy solid was further stirred for 16 h with dichloromethane (50 mL) and collected by filtration. This procedure was repeated with diethyl ether. After drying, a white solid was obtained (70%). Following the procedure reported previously *Chem. - Eur. J.* **1999**, *5*, 937−950

^{1}H NMR (300 MHz, $CDCl_3$) δ 5.18 (br, 2H), 4.67 (br, 2H), 3.42 (m, 2H), 3.08 (m, 4H), 2.03 (d, 2H), 1.72 (m, 6H), 1.45 (m, 4H), 1.25 (s, 36H), 0.87 (t, 6H) ppm.

Searching the optimal conditions

Table S1.

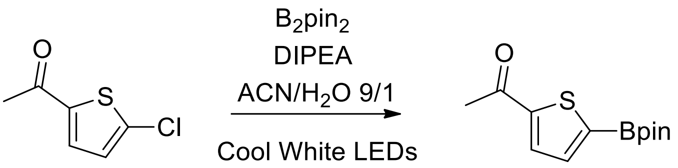

1a
0.02M

2aa

Entry	*B_2pin_2 (eq)*	*DIPEA (eq)*	*G1/solvent*	*Conv (%)*	*Yield of **2aa** (%)*
1	3	1.5	21 mg/2mL	**100**	**16**
2	5	1.2	10 mg/1mL	**92**	**57**
3	5	1.5	21 mg/2mL	**100**	**28**
4	15	1.2	10 mg/1mL	**26**	**18**
5	10	1.5	21 mg/2mL	**100**	**60**
6	20	1.5	21 mg/2mL	**92**	**69**
7	10	1	21 mg/2mL	**94**	**62**
8	10	1	10 mg/1mL	**46**	**35**
9	10	3	21 mg/2mL	**100**	**38**
10	10	3	10 mg/1mL	**100**	**58**
11	10	1.2	21 mg/2mL	**100**	**64**
12	10	1.5	42 mg/4mL	**92**	**40**
13	10	1.2	42 mg/4mL	**100**	**53**
14	10	1.2	10 mg/1mL	**100**	**72**
15	10	1.2	8 mg/1mL	**70**	**43**
16	10	1.2	15 mg/1mL	**59**	**37**
17	10	1.2	[a]8.5 mg/1mL	**59**	**38**
18	10	1.2	[a]10 mg/1mL	**86**	**56**
19	10	1.2	[a]5 mg/1mL	**55**	**44**
20	10	1.2	Air[b]	**-**	**-**
21	10	1.2	N_2[b]	**75**	**56**
22	10	1.2	G1/N_2[b]	**46**	**34**
23	10	-	10 mg/1mL	-	-
24	-	1.2	10 mg/1mL	-	-
25[c]	10	1.2	10 mg/1mL	-	-
26	10	Et_3N 1.2	10 mg/1mL	**19**	**11**
27	10	DIPA 1.2	10 mg/1mL	**29**	**29**
28	10	DBU 1.2	10 mg/1mL	**66**	**45**
29	10	DABCO 1.2	10 mg/1mL	**15**	**7.8**
30	10	Ph_3N 1.2	10 mg/1mL	-	-
31	10	1.2	Frozen No G1	-	-
32	10	1.2	Frozen; 10 mg/1mL	**35**	**30**
33	10	1.2	[d]N_2 and 10 mg/1mL	**73**	**60**

[a]G2 as gelator was used. [b]Solution reaction without gel. [c]Without 1a. [d]Purged with N_2 before gel formation.

Kinetic studies

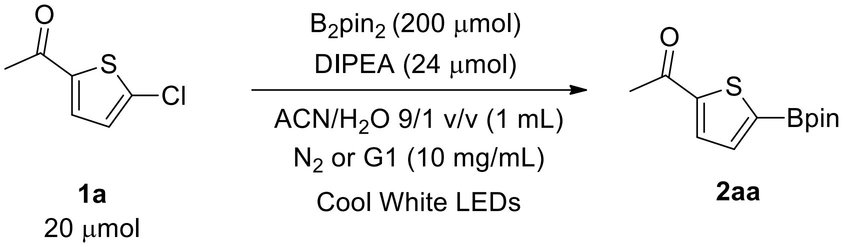

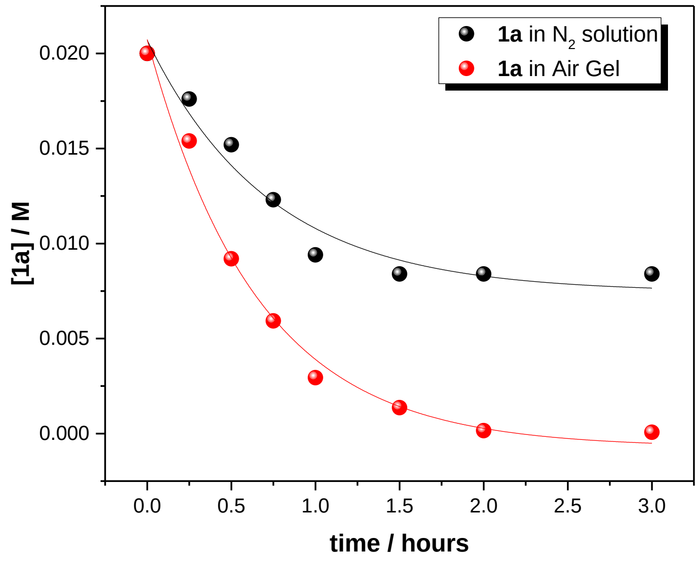

Figure S1. Variation of **1a** concentration upon irradiation time in different conditions.

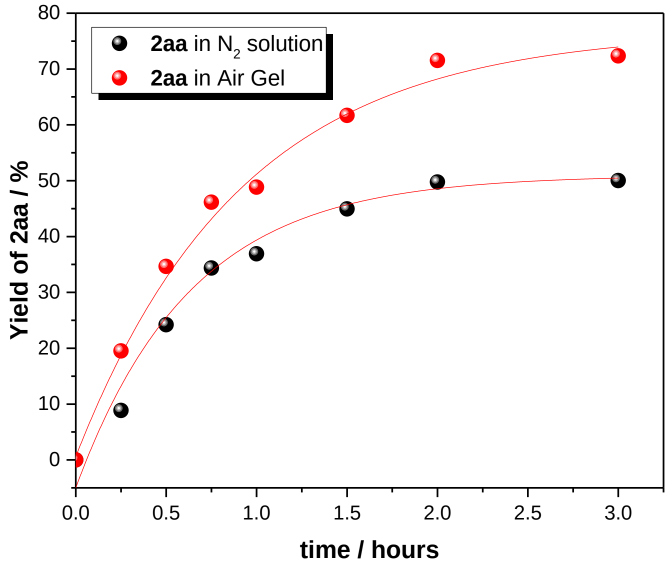

Figure S2. Formation of **2aa** upon irradiation time in different conditions.

Frozen model reaction procedure

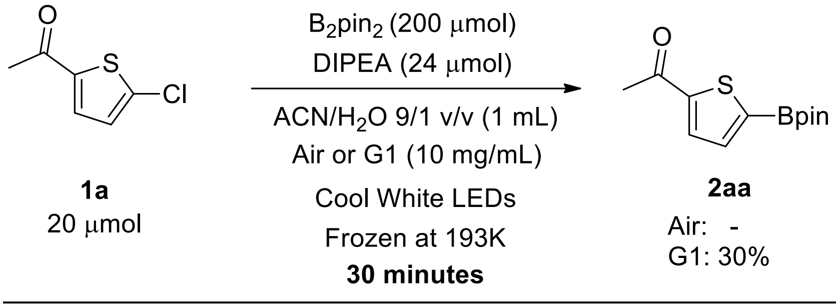

A vial (10 mL) was charged with 1-(5-chlorothiophen-2-yl)ethenone (3.2 mg, 20 µmol, 1.0 equiv.), bispinacolato diboron (50 mg, 200 µmol, 10.0 equiv.) and the correspondent gelator (G1, 10 mg/mL). Acetonitrile/H_2O (9/1 v/v) mixture (1.0 mL) was poured, and it was stirred until almost complete solution. Then, DIPEA (4.2 µL, 24 µmol, 1.2 equiv.) and dodecanenitrile (4.4 µL, 20 µmol, 1.0 equiv.) were added with 10 µL Hamilton syringe. Quickly, the vial was sealed with a septum. It was heated to 150 °C with heatgun for 1.5 minutes with manual stirring until complete clear solution. The vial cooled to room temperature until gel formation was observed. The vial was frozen at 193K for 2 hours. Then, the reaction was irradiated with an external LED through the plain bottom side of the vial at 23 °C for 30 minutes. The reaction was monitored by GC-FID analysis. The gel was broken with dichloromethane (2 mL) and it was clean with brine (6 mL). The organic phase was dried over anhydrous sodium sulfate, filtered from the drying agent, and concentrated in vacuo.

Field-emission scanning electron microscopy (FESEM)

The equipment in operation in the UPV Microscopy Service is the ZEISS ULTRA 55 model, incorporating the following detectors:
·A Secondary Electron Detector (SE2), which provides an SEM topography image of the sample surface with a large depth of field.
· A Secondary Electron In-Lens Detector located inside the electron column, which works with low energy secondary electrons and provides images with a higher resolution.
· A Backscattered Electron Detector (AsB) which is sensitive to the variation of atomic number in the elements present in the sample; therefore, it is used to observe changes in the chemical composition of the specimen.
· A Backscattered Electron In-lens Detector (EsB), independent of the secondary In-lens detector, which provides a pure backscattered signal with no secondary electron contamination and very low acceleration potential.
· An X-Ray Dispersive Energy Detector, EDS, (Oxford Instruments) which receives x-rays from each surface point the electron beam passes over.

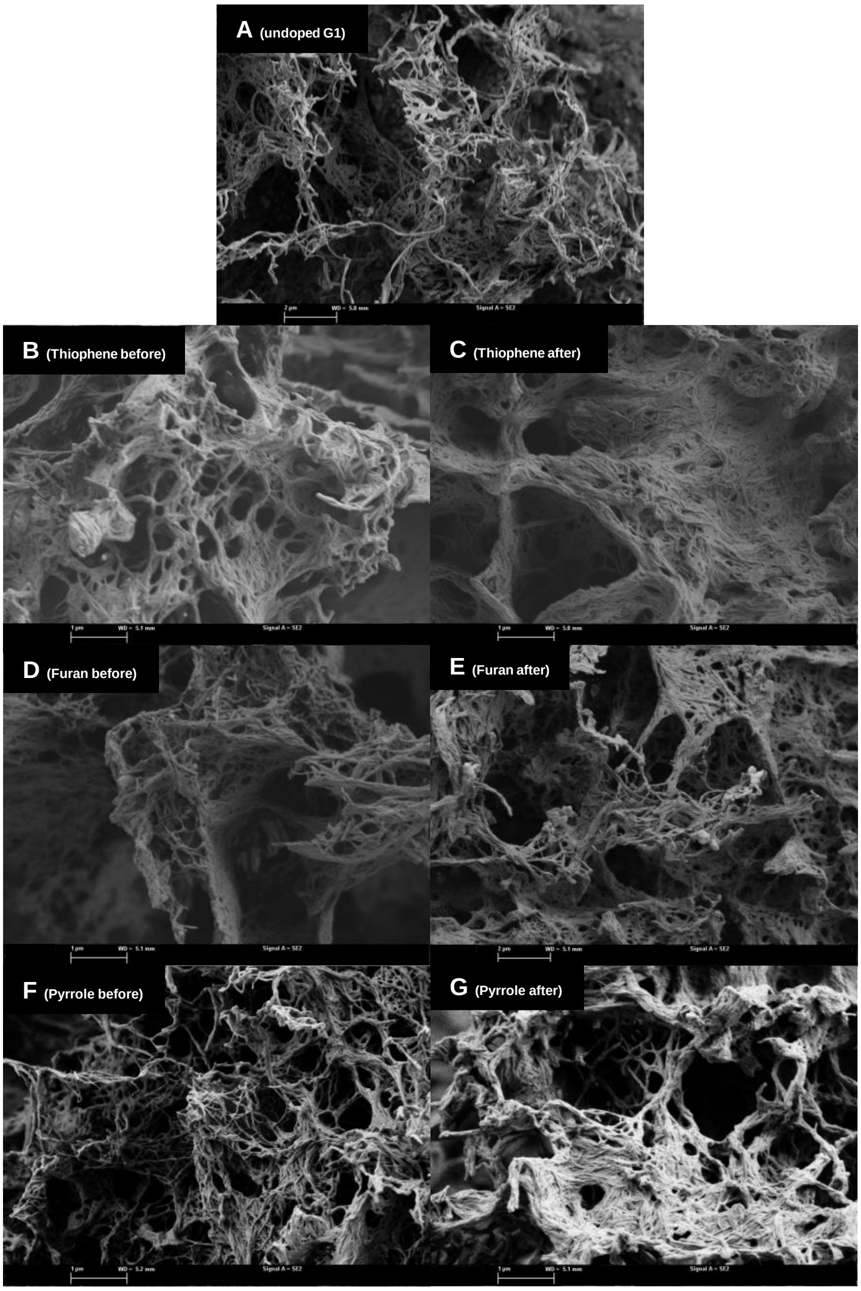

Figure S3. Representative field-emission scanning electron microscopy (FESEM) images. **A**: undoped gel of **G1** (10 mg/mL). **B, C**: gel of **G1** (10 mg/mL) doped with 2-acetyl-5-chlorothiophene (3.2 mg), B_2pin_2 (50 mg) and DIPEA (3.1 mg). **D, E**: gel of **G1** (10 mg/mL) doped with 2-acetyl-5-bromofuran (3.8 mg), B_2pin_2 (50 mg) and DIPEA (3.1 mg). **F, G**: gel of **G1** (10 mg/mL) doped with 2-acetyl-5-bromo-1H-methylpyrrole (4.0 mg), B_2pin_2 (50 mg) and DIPEA (3.1 mg).

Determination of gel-to-sol transition temperature (T_{gel})

T_{gel} values were determined using a custom-made set-up where a sealed vial was placed into a mold of an alumina block and heated up at 1 °C/5 min using an electric heating plate equipped with a temperature control couple as previously described (see for instance, *Chem. Commun.* **2015**, *51*, 16848-16851). The temperature at which the gel started to break was defined as T_{gel} with an estimated error of ± 2 ºC after several heating-cooling cycles.

UV-vis absorption spectra

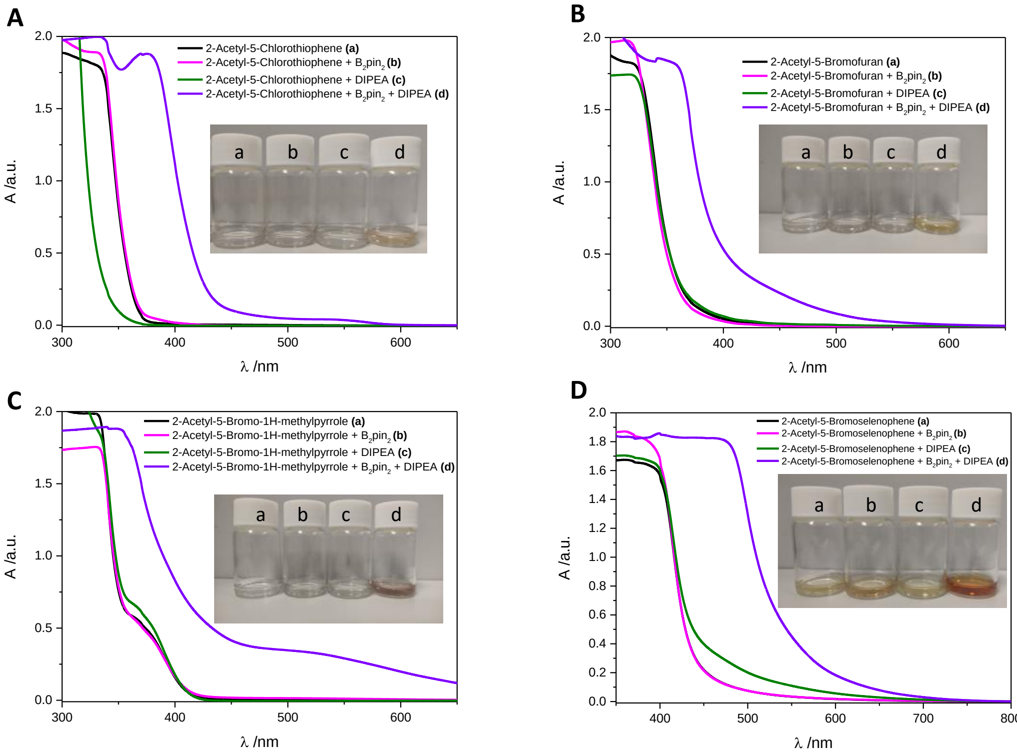

Figure S4. UV-vis absorption spectra. Inset: photographs of different solutions. **A**: 2-Acetyl-5-Chlorothiophene (0.02 mmol), B_2pin_2 (0.2 mmol) and DIPEA (0.024 mmol) in ACN/H_2O 9/1 v/v 1 mL. **B**: 2-Acetyl-5-Bromofuran (0.02 mmol), B_2pin_2 (0.2 mmol) and DIPEA (0.024 mmol) in ACN/H_2O 9/1 v/v 1 mL. **C**: 2-Acetyl-5-Bromo-1H-methylpyrrole (0.02 mmol), B_2pin_2 (0.2 mmol) and DIPEA (0.024 mmol) in ACN/H_2O 9/1 v/v 1 mL. **D**: 2-Acetyl-5-Bromoselenophene (0.02 mmol), B_2pin_2 (0.2 mmol) and DIPEA (0.024 mmol) in ACN/H_2O 9/1 v/v 1 mL.

Singlet Oxygen

Time-resolved measurements of $^1O_2$ luminiscence were performed in a front face setup in FLS 1000 (Edinburgh Instruments) spectrophotometer by making use of a 450W Xe lamp and a NIR sensitive PMT 1700 detector (H10330C-75, Hamamatsu) in Peltier Cooled Housing.

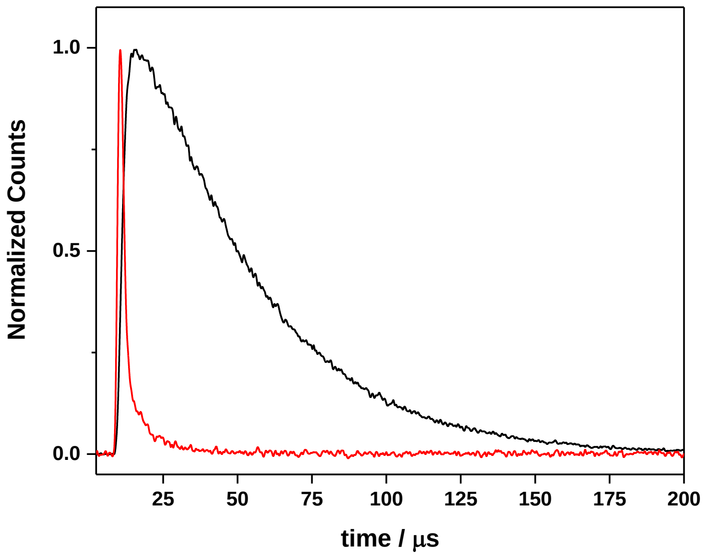

Figure S5. Decay traces of the $^1O_2$ emission (1275 nm) generated by an aerobic ACN/H_2O (9/1 v/v) solution of; **black**: Rose Bengal (0.1 mM, λ_{exc} = 532 nm); **red**: 1a+B_2pin_2+DIPEA (20 mM, 200 mM and 24 mM, respectively, λ_{exc} = 400 nm).

GC-MS Byproduct

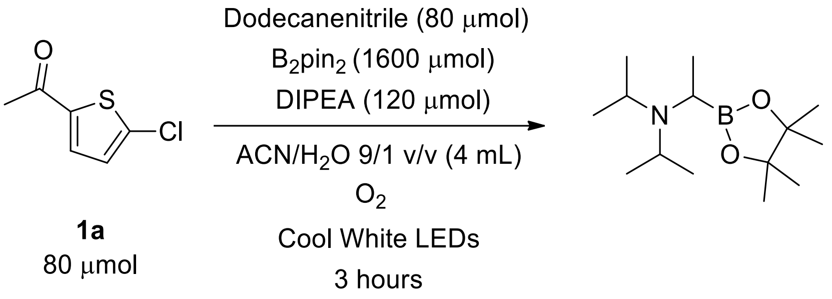

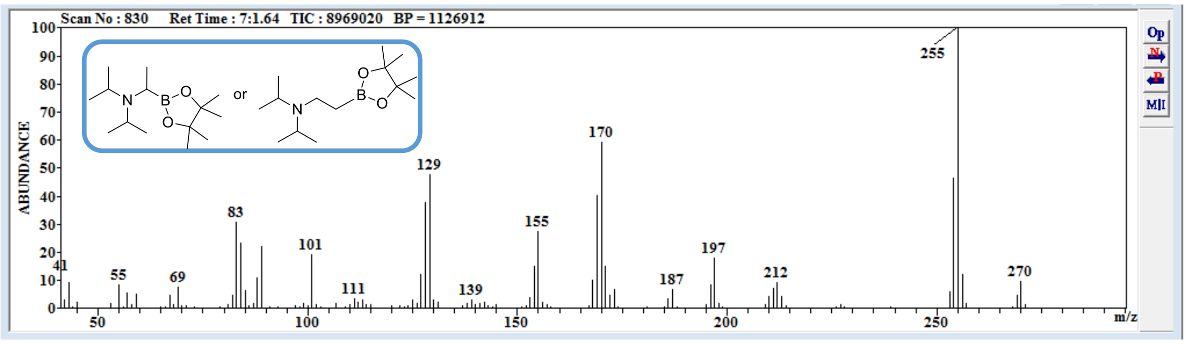

Trapping reaction

A vial (10 mL) was charged with heteroarene derivative (20 µmol, 1.0 equiv.), diborylates (200 µmol, 10.0 equiv.), diphenyldisulfide (20 µmol, 1.0 equiv.) and the correspondent gelator (G1, 10 mg/mL). Acetonitrile/H_2O (9/1 v/v) mixture (1.0 mL) was poured, and it was stirred until almost complete solution. Then, DIPEA (24 µmol, 1.2 equiv.) and dodecanenitrile (20 µmol, 1.0 equiv.) were added with 10 µL Hamilton syringe. Quickly, the vial was sealed with a septum. It was heated to 150 °C for 1.5 minutes with manual stirring until complete clear solution. The vial cooled to room temperature until gel formation was observed. The reaction was irradiated with an external LED through the plain bottom side of the vial at 23 °C for 3 hours. Then, brine (6 mL) was added, and the aqueous phase was extracted with dichloromethane (2 mL). The reaction was monitored by GC-FID analysis. The organic phase was dried over anhydrous sodium sulfate, filtered from the drying agent, and concentrated in vacuo. After crystallization of the excess of starting diboron derivative, the crude was purified via TLC plastic sheet using a hexane/ethyl acetate mixture as the mobile phase.

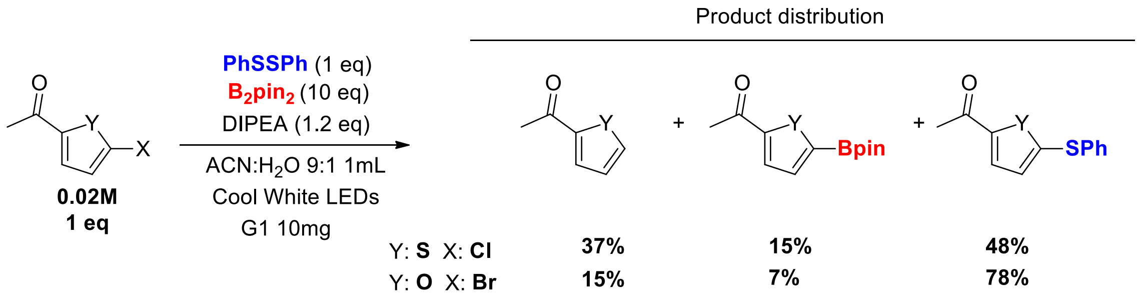

Mechanism

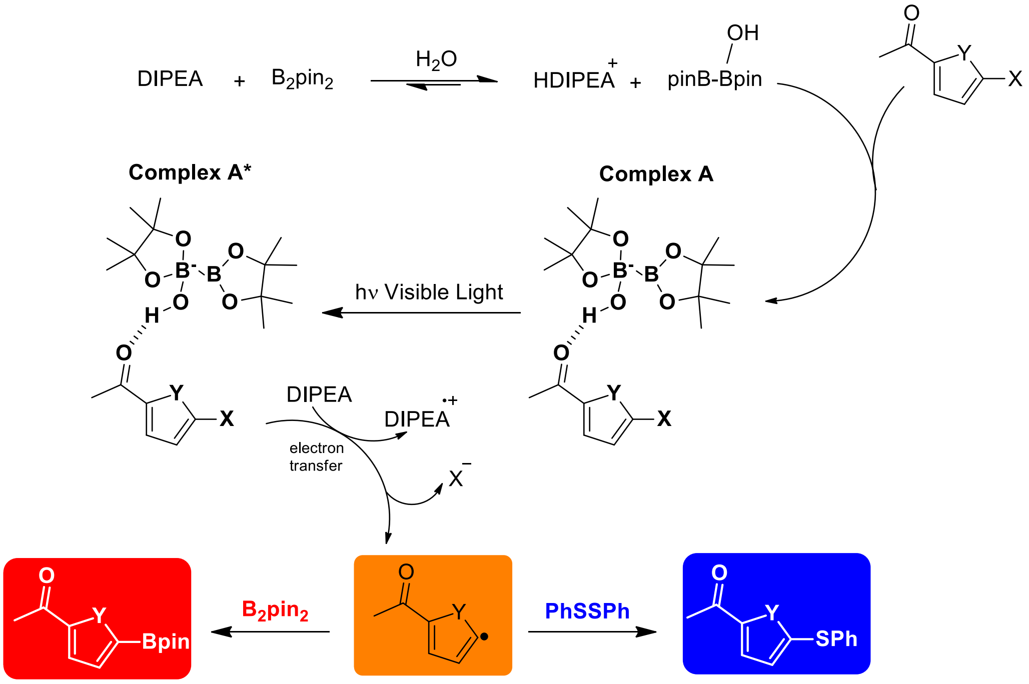

Sunlight irradiation and 1 mmol scale

Sunlight irradiation

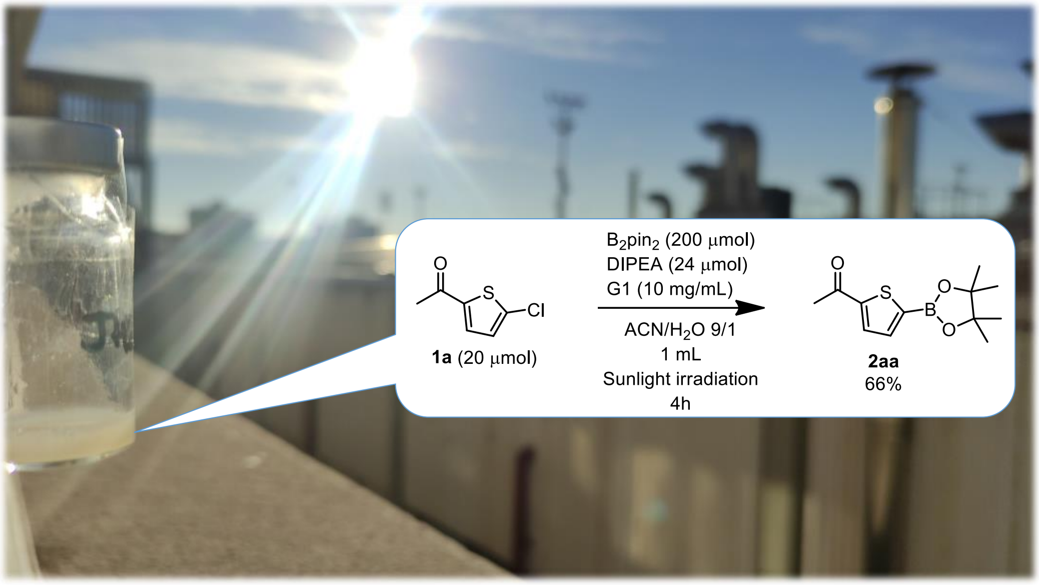

The compound **2aa** (CAS 942070-32-8) was prepared according to the general procedure using 1-(5-chlorothiophen-2-yl)ethenone (3.2 mg, 20 µmol, 1.0 equiv.), bis(pinacolato)diboron (50.6 mg, 200 µmol, 10.0 equiv.), dodecanenitrile (4.4 µL, 20 µmol, 1.0 equiv.) as internal standard and DIPEA (4.2 µL, 24 µmol, 1.2 equiv.) and G1 (10 g/L). The reaction mixture was irradiated with sunlight for 4 hours, obtaining 66% product yield according to GC-FID analysis.
Location: Camí de Vera S/N, Chemistry Department, Universitat Politècnica de València (UPV), Valencia, Spain (coordinate: 39.482917, -0.341642), temperature: 12-16 °C, from 9:00 to 13:00. Date: 23/12/2020.

1 mmol scale

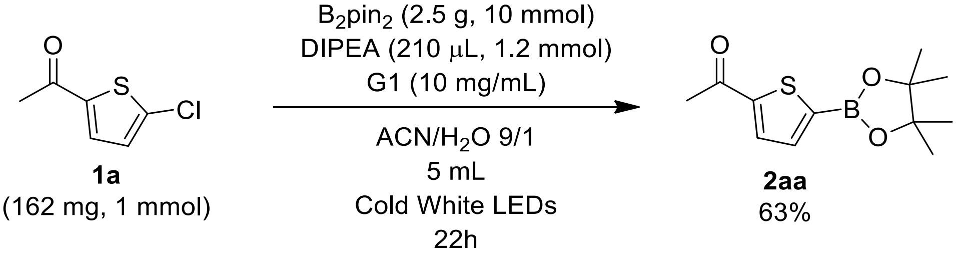

The compound **2aa** (CAS 942070-32-8) was prepared according to the general procedure using 1-(5-chlorothiophen-2-yl)ethenone (162 mg, 1 mmol, 1.0 eq.), bis(pinacolato)diboron (2.5 g, 10 mmol, 10.0 eq.), dodecanenitrile (220 µL, 1 mmol, 1.0 equiv.) as internal standard and DIPEA (210 µL, 1.2 mmol, 1.2 eq.) and G1 (10 mg/mL) in 5 mL. The reaction mixture was irradiated for 22 hours, obtaining 63% product yield according to GC-FID analysis.

GC chromatograms

Thiophenes

2aa

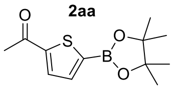

Before

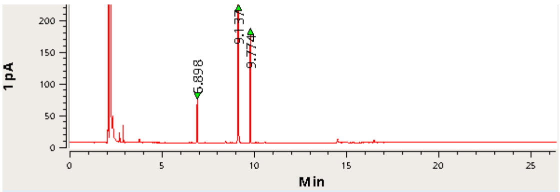

After

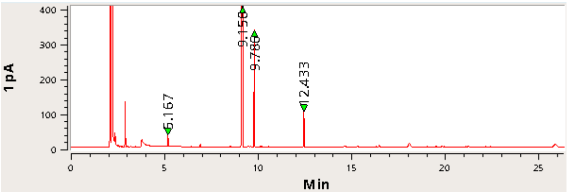

2ab

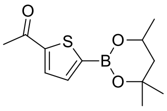

Before

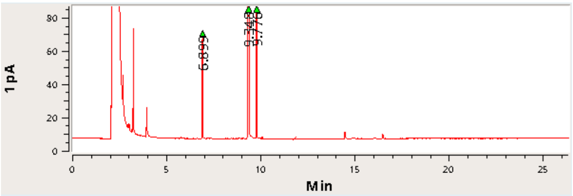

After

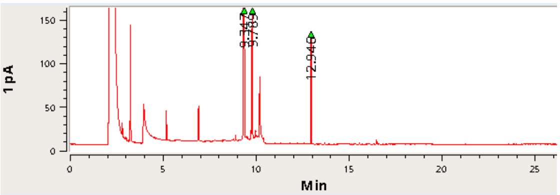

2ac

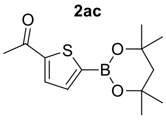

Before

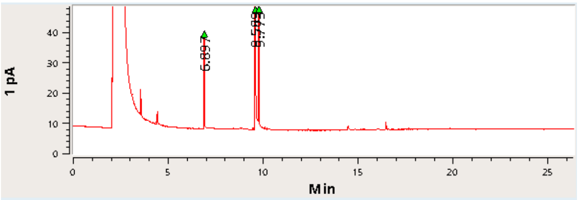

After

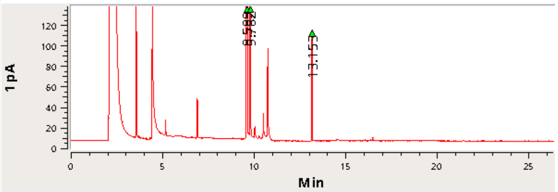

2ad

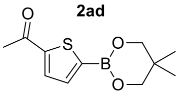

Before

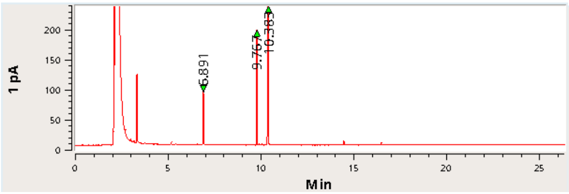

After

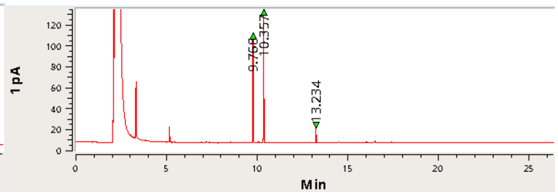

2ba

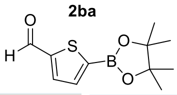

Before

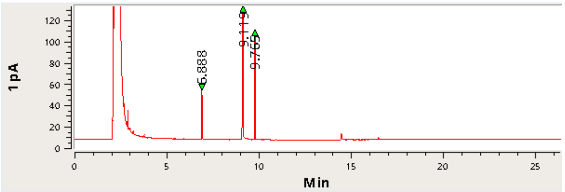

After

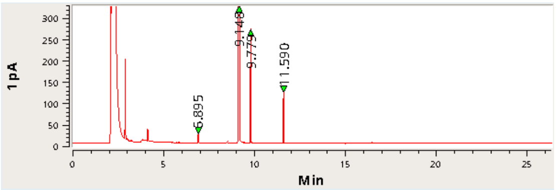

2bb

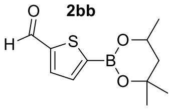

Before

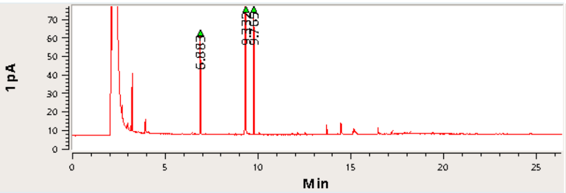

After

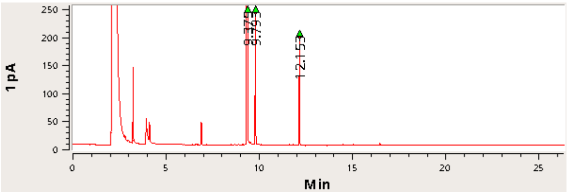

2bc

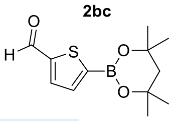

Before

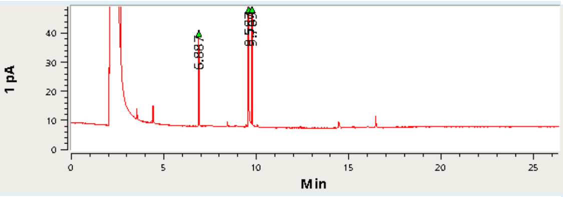

After

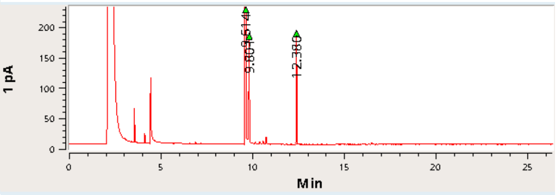

2bd

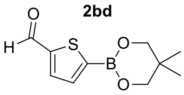

Before

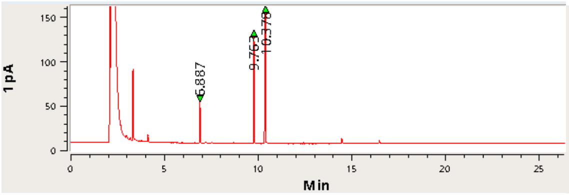

After

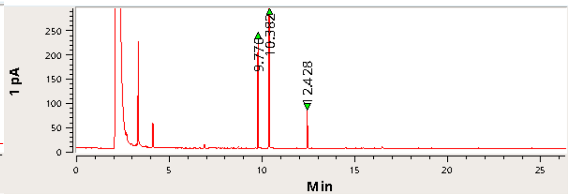

2ca

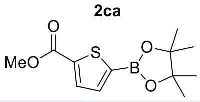

Before

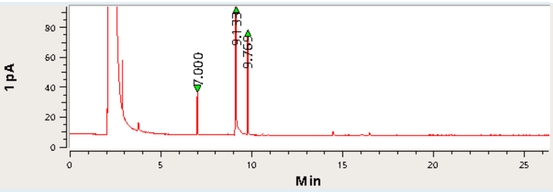

After

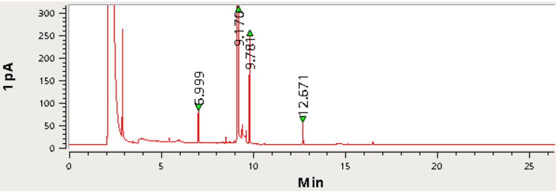

2cb

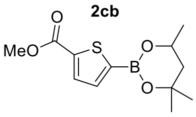

Before

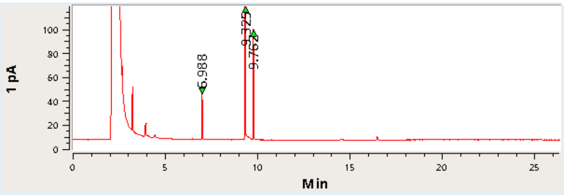

After

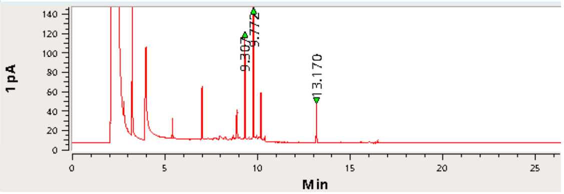

2cc

Before

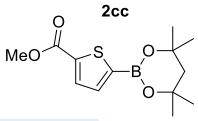

After

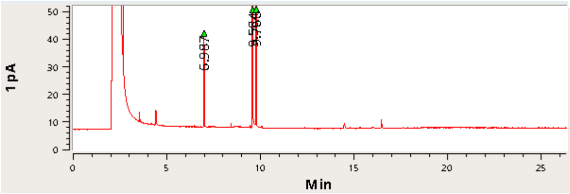

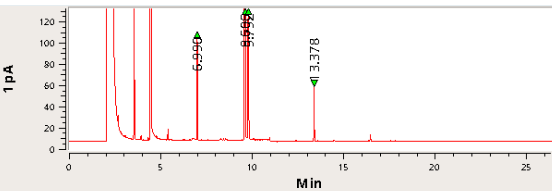

2cd

Before

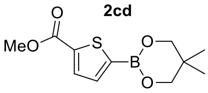

After

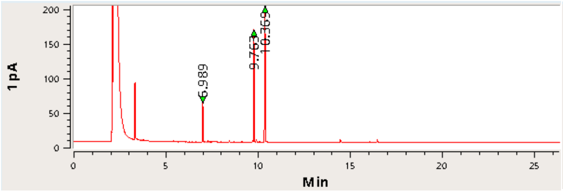

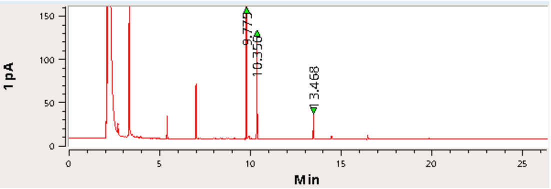

2da

Before

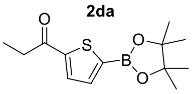

After

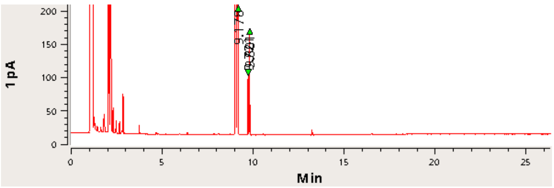

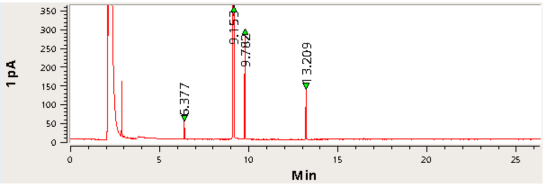

2db

Before

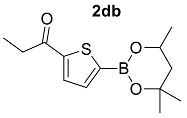

After

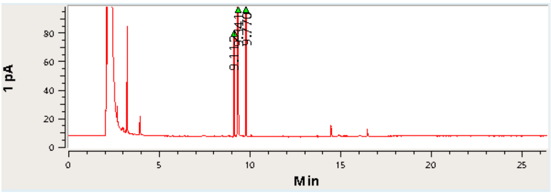

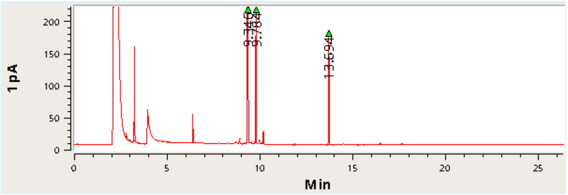

2dc

Before

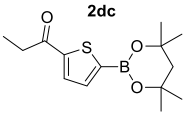

After

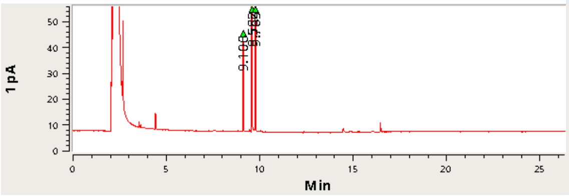

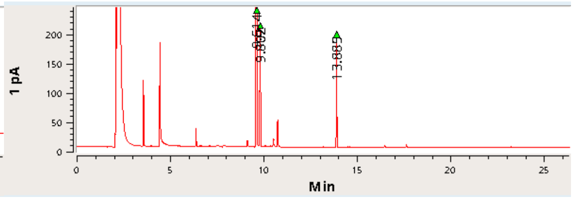

2dd

Before

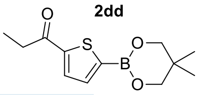

After

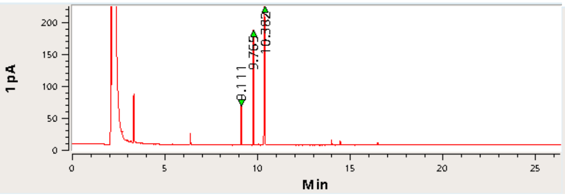

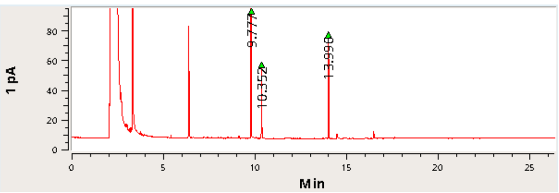

2ea

Before

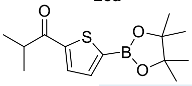

After

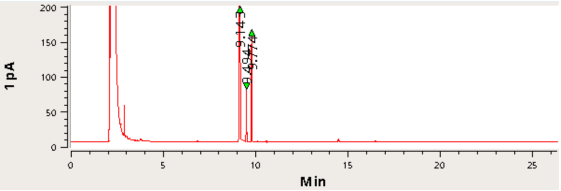

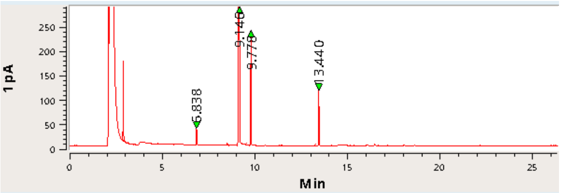

2eb

Before

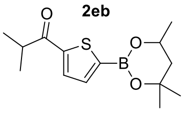

After

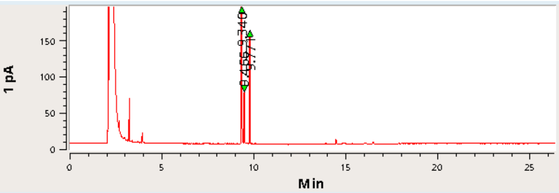

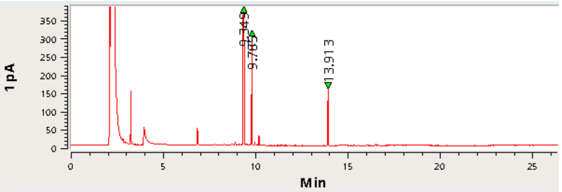

2ec

Before

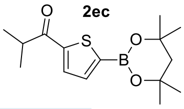

After

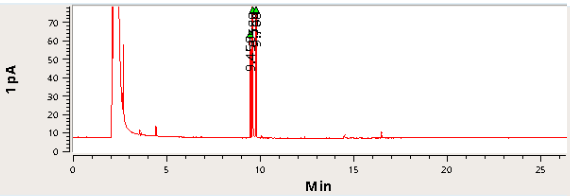

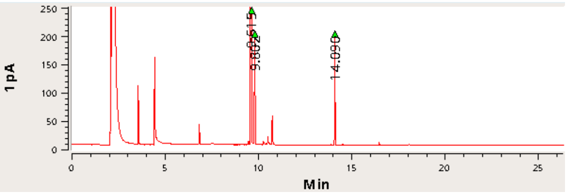

2ed

Before

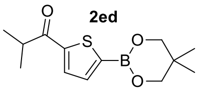

After

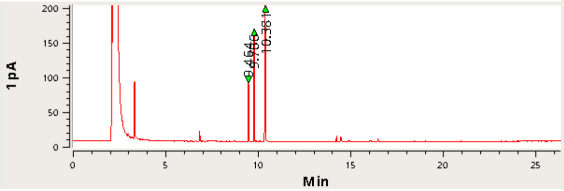

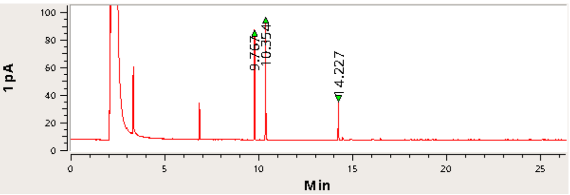

2fa

Before

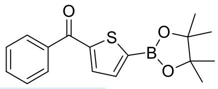

After

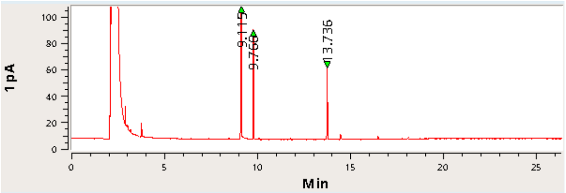

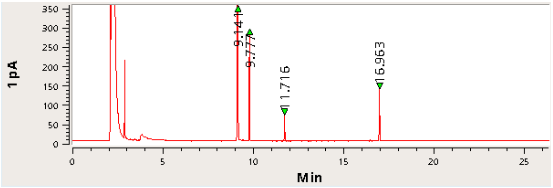

2fb

Before

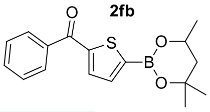

After

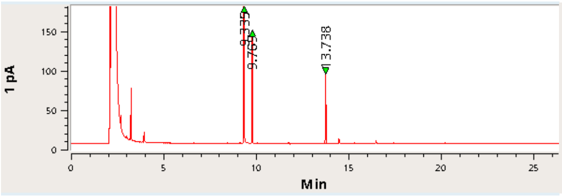

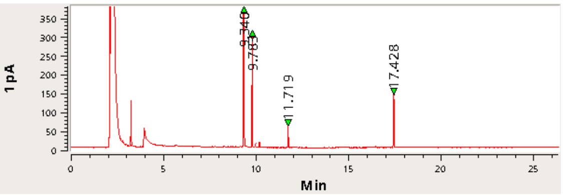

2fc

Before

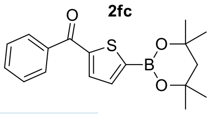

After

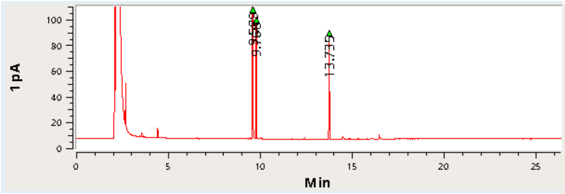

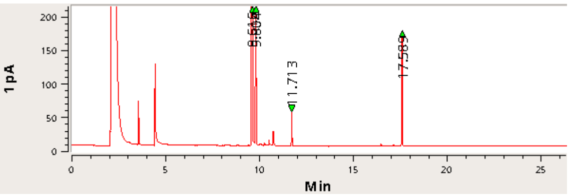

2fd

Before

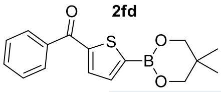

After

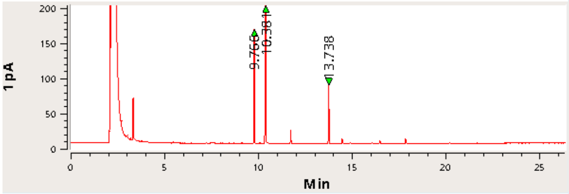

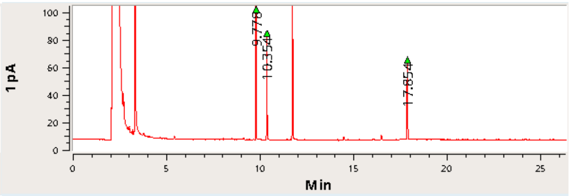

Furans

3aa

Before

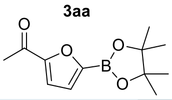

After

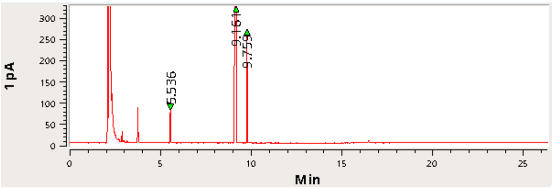

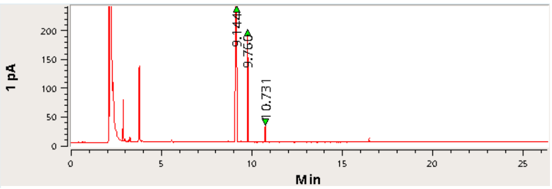

3ab

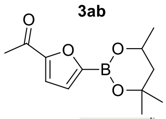

Before

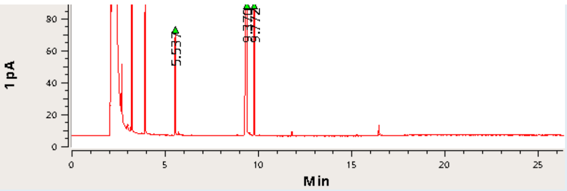

After

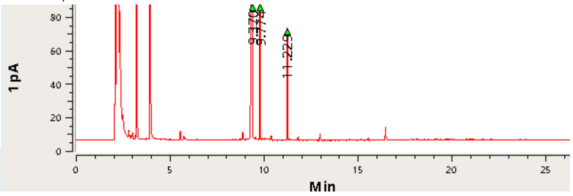

3ac

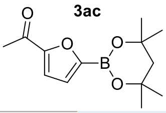

Before

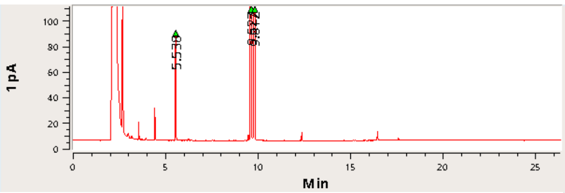

After

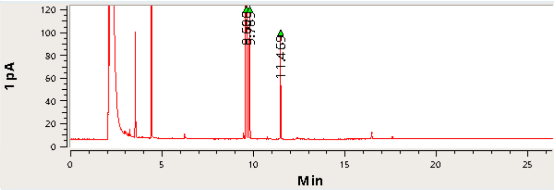

3ad

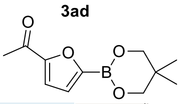

Before

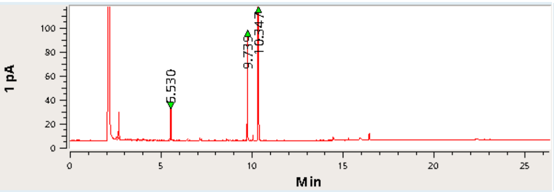

After

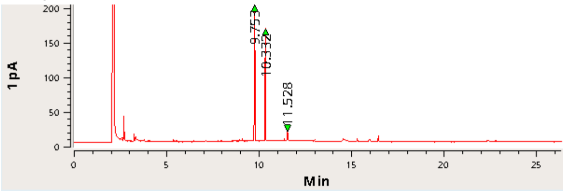

3ba

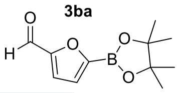

Before

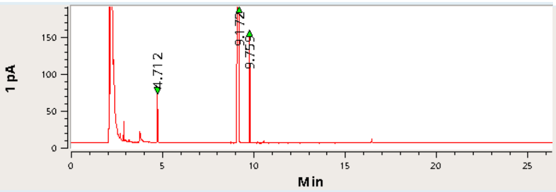

After

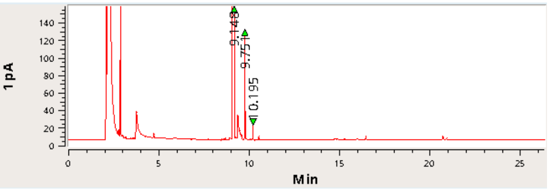

3bb

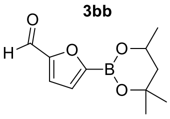

Before

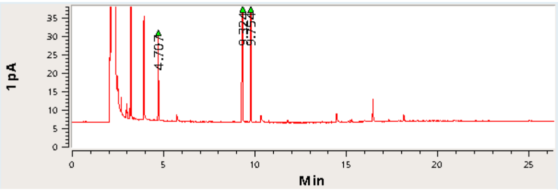

After

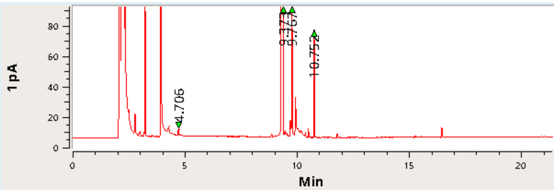

Before

3bc

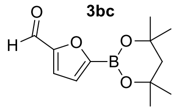

After

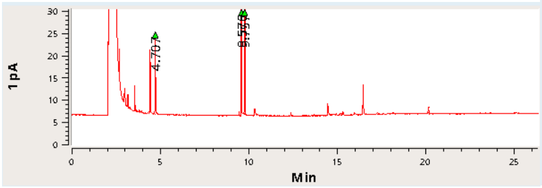

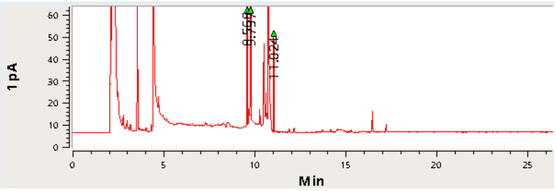

Before

3bd

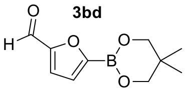

After

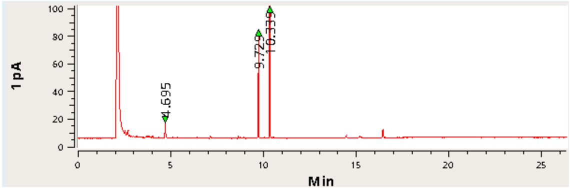

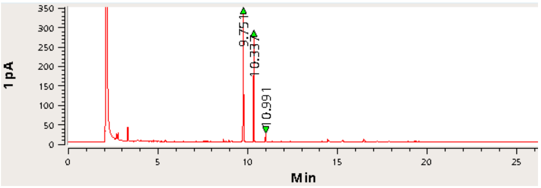

Before

3ca

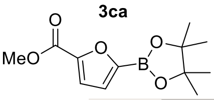

After

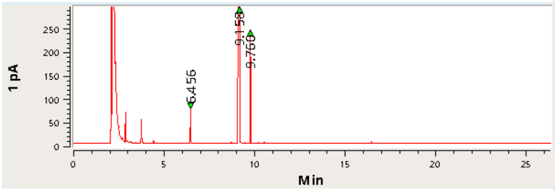

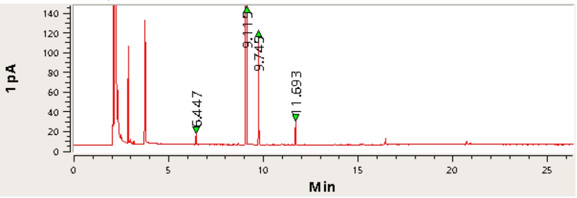

Before

3cb

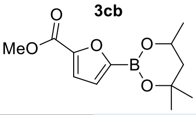

After

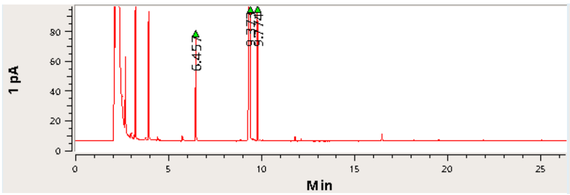

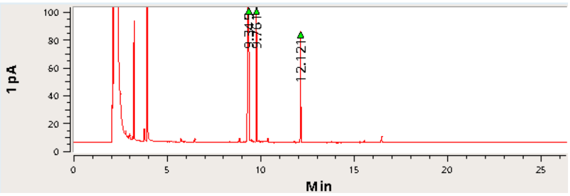

Before

3cc

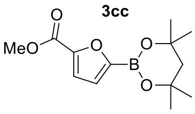

After

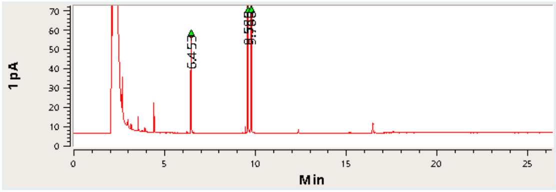

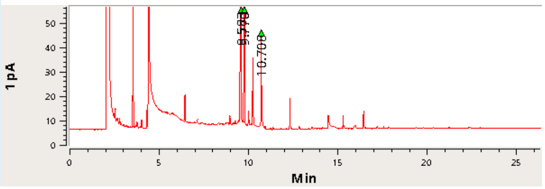

3cd

Before

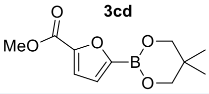

After

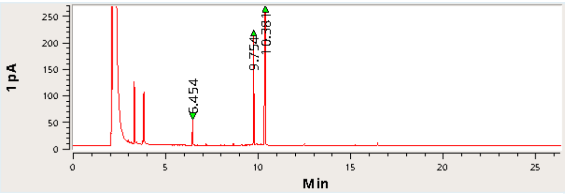

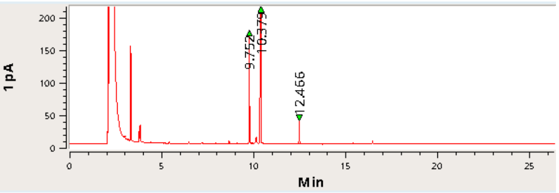

Pyrroles

4aa

Before

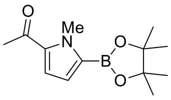

After

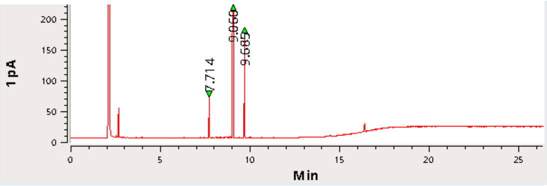

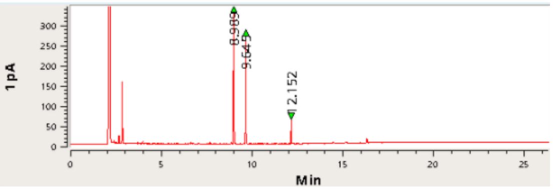

4ab

Before

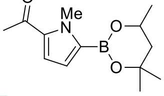

After

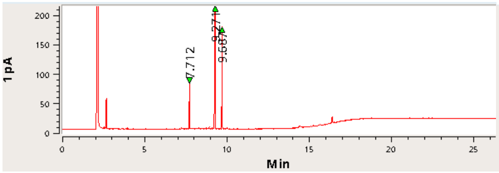

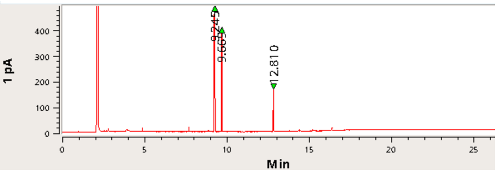

4ac

Before

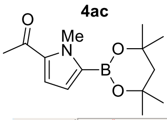

After

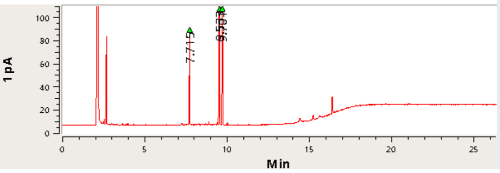

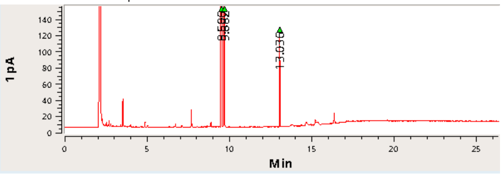

4ad

Before

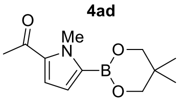

After

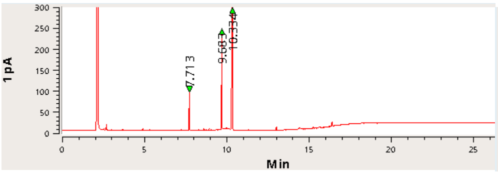

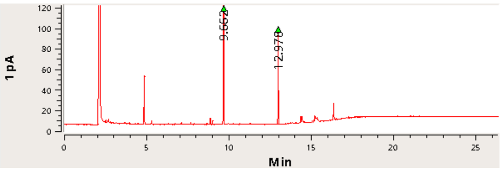

4ba

Before

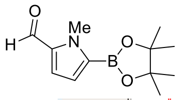

After

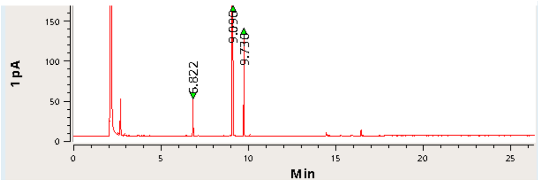

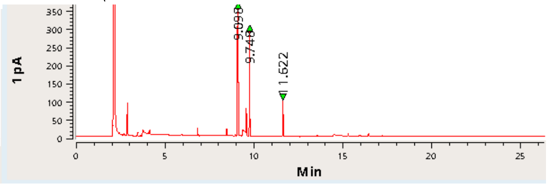

4bb

Before

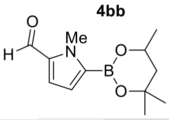

After

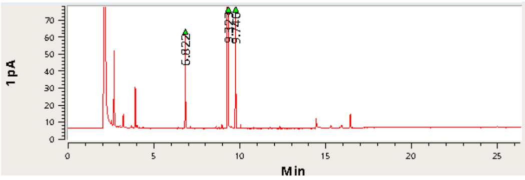

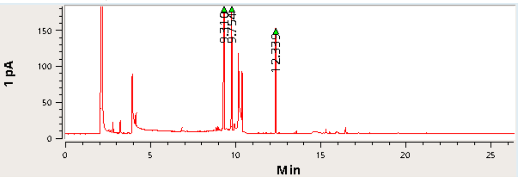

4bc

Before

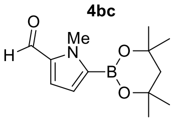

After

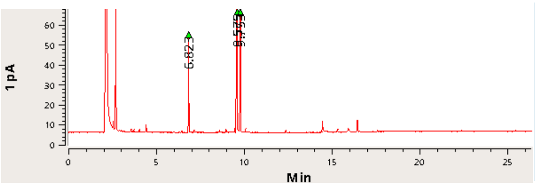

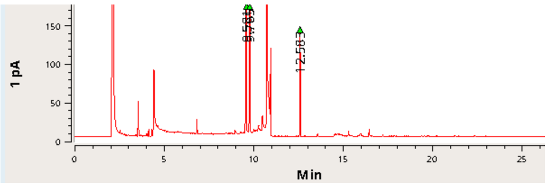

4bd

Before

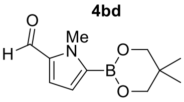

After

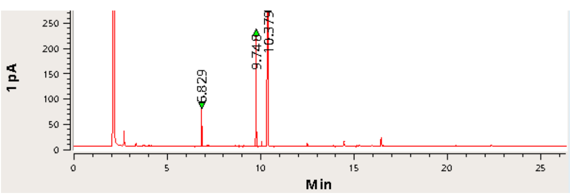

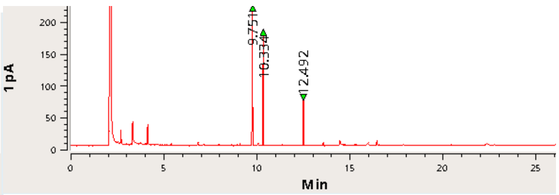

4ca

Before

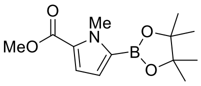

After

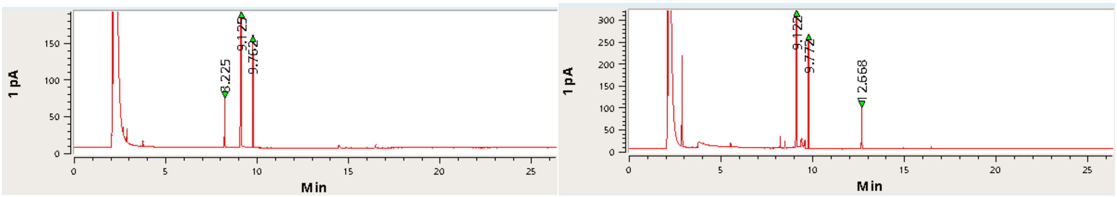

4cb

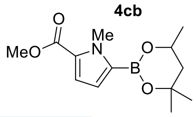

Before | After

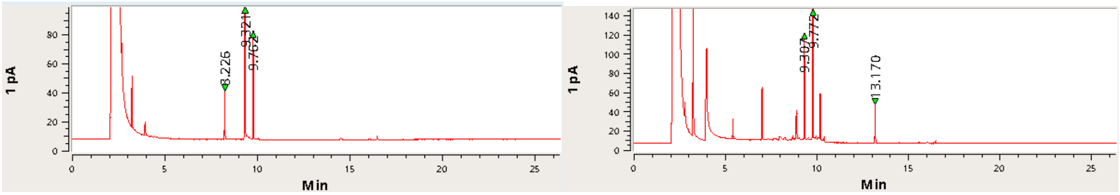

4cc

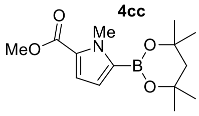

Before | After

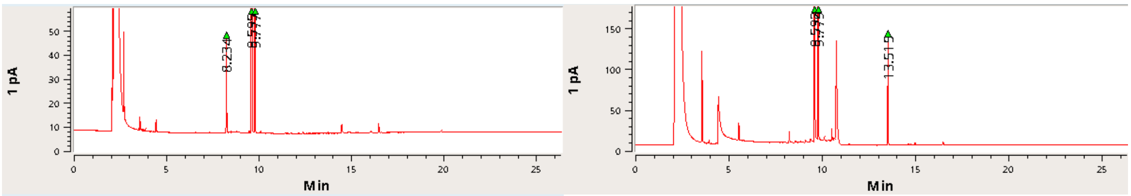

4cd

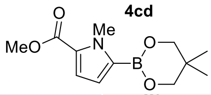

Before | After

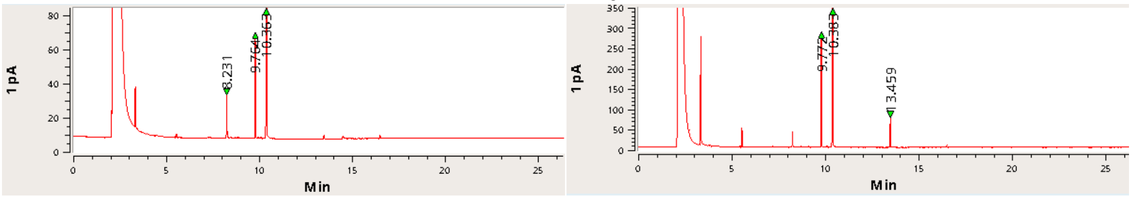

Selenophenes

5aa

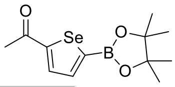

Before | After

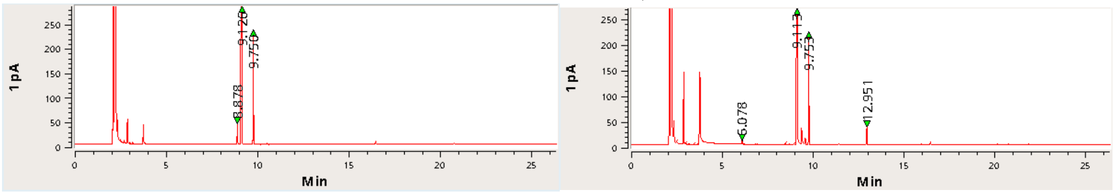

5ab

Before

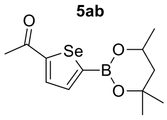

After

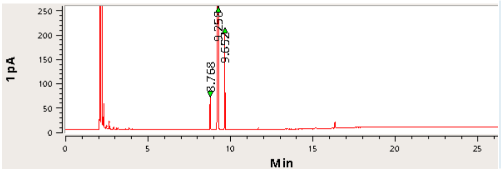

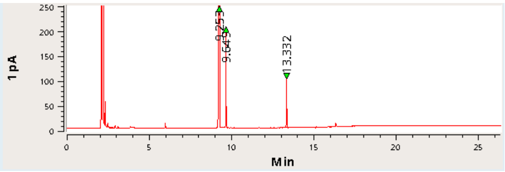

5ac

Before

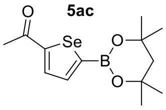

After

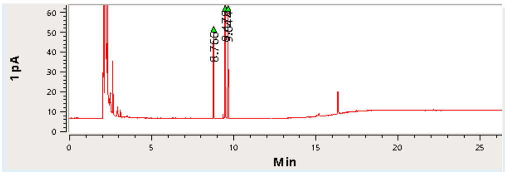

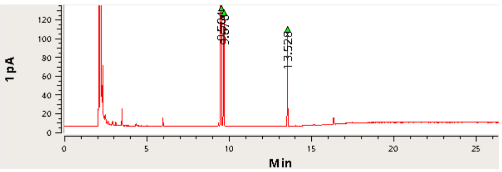

5ad

Before

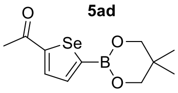

After

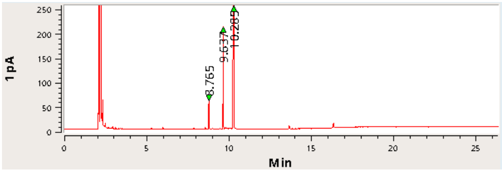

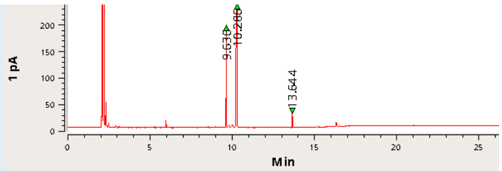

Characterization of compounds

THIOPHENES

2aa. 1-(5-(4,4,5,5-tetramethyl-1,3,2-dioxaborolan-2-yl)thiophen-2-yl)ethenone

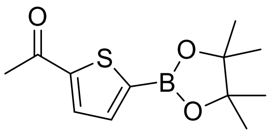

The compound (CAS 942070-32-8) was prepared according to the general procedure using 1-(5-chlorothiophen-2-yl)ethenone (3.2 mg, 20 µmol, 1.0 equiv.), bis(pinacolato)diboron (50.6 mg, 200 µmol, 10.0 equiv.), dodecanenitrile (4.4 µL, 20 µmol, 1.0 equiv.) as internal standard and DIPEA (4.2 µL, 24 µmol, 1.2 equiv.) and G1 (10 g/L). The reaction mixture was irradiated for 2 hours, obtaining 72% product yield according to GC-FID analysis (63% isolated yield as white powder).

^{1}H NMR (400 MHz, $CDCl_3$) δ 7.72 (d, *J* = 3.7 Hz, 1H), 7.58 (d, *J* = 3.7 Hz, 1H), 2.57 (s, 3H), 1.35 (s, 12H) ppm.

^{13}C NMR (101 MHz, $CDCl_3$) δ 190.8 (C), 149.6 (C), 137.4 (CH), 132.8 (CH), 84.8 (C), 27.6 (CH_3), 24.9 (CH_3).

Spectral data were consistent with literature.[1]

2ab. 1-(5-(4,4,6-trimethyl-1,3,2-dioxaborinan-2-yl)thiophen-2-yl)ethenone

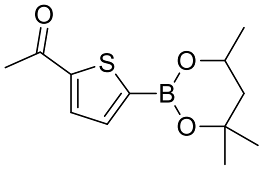

The compound (CAS 2414250-10-3) was prepared according to the general procedure using 1-(5-chlorothiophen-2-yl)ethenone (3.2 mg, 20 µmol, 1.0 equiv.), bis(hexyleneglycolato)diboron (53.0 mg, 200 µmol, 10.0 equiv.), dodecanenitrile (4.4 µL, 20 µmol, 1.0 equiv.) as internal standard and DIPEA (4.2 µL, 24 µmol, 1.2 equiv.) and G1 (10 g/L). The reaction mixture was irradiated for 4 hours, obtaining 61% product yield according to GC-FID analysis (50% isolated yield as white powder).

^{1}H NMR (400 MHz, $CDCl_3$) δ 7.63 (d, *J* = 3.7 Hz, 1H), 7.43 (d, *J* = 3.7 Hz, 1H), 4.29 (ddd, *J* = 11.6, 6.1, 2.9 Hz, 1H), 2.50 (s, 3H), 1.82 (dd, *J* = 14.0, 2.9 Hz, 1H), 1.56 (dd, *J* = 13.9, 11.7 Hz, 1H), 1.31 (s, 6H), 1.28 (d, *J* = 6.2 Hz, 3H) ppm.

^{13}C NMR (101 MHz, $CDCl_3$) δ 190.8 (C), 148.2 (C), 135.5 (CH), 132.7 (CH), 72.1 (C), 65.8 (CH), 45.6 (CH_2), 31.1 (CH_3), 28.0 (CH_3), 27.7 (CH_3), 23.0 (CH_3) ppm.

Spectral data were consistent with literature.[1]

2ac. 1-(5-(4,4,6,6-tetramethyl-1,3,2-dioxaborinan-2-yl)thiophen-2-yl)ethenone

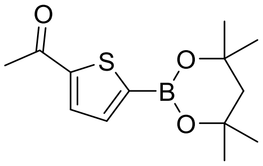

The compound (CAS 2414250-15-8) was prepared according to the general procedure using 1-(5-chlorothiophen-2-yl)ethenone (3.2 mg, 20 µmol, 1.0 equiv.), 4,4,4',4',6,6,6',6'-octamethyl-2,2'-bi(1,3,2-dioxaborinane) (56.4 mg, 200 µmol, 10.0 equiv.), dodecanenitrile (4.4 µL, 20 µmol, 1.0 equiv.) as internal standard and DIPEA (4.2 µL, 24 µmol, 1.2 equiv.) and G1 (10 g/L). The reaction mixture was irradiated for 3 hours, obtaining 63% product yield according to GC-FID analysis (50% isolated yield as white powder).

^{1}H NMR (400 MHz, $CDCl_3$) δ 7.68 (d, *J* = 3.7 Hz, 1H), 7.50 (d, *J* = 3.7 Hz, 1H), 2.55 (s, 3H), 1.92 (s, 2H), 1.41 (s, 12H) ppm.

---

[1] J. C. Herrera-Luna, D. Sampedro, M. C. Jiménez and R. Pérez-Ruiz, *Organic Letters*, 2020, **22**, 3273-3278.

^{13}**C NMR** (101 MHz, $CDCl_3$) δ 191.0 (C), 148.3 (C), 135.5 (CH), 132.8 (CH), 71.9 (C), 49.1 (CH_2), 31.8 (CH_3), 27.5 (CH_3) ppm.
Spectral data were consistent with literature.[1]

2ad. 1-(5-(5,5-dimethyl-1,3,2-dioxaborinan-2-yl)thiophen-2-yl)ethenone

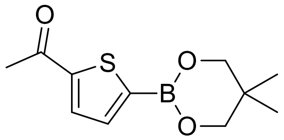

The compound (CAS 2414250-20-5) was prepared according to the general procedure using 1-(5-chlorothiophen-2-yl)ethenone (3.2 mg, 20 µmol, 1.0 equiv.), bis(neopentylglycolato)diboron (45.2 mg, 200 µmol, 10.0 equiv.), dodecanenitrile (4.4 µL, 20 µmol, 1.0 equiv.) as internal standard and DIPEA (4.2 µL, 24 µmol, 1.2 equiv.) and G1 (10 g/L). The reaction mixture was irradiated for 3 hours, obtaining 50% product yield according to GC-FID analysis (45% isolated yield as white powder).

^{1}**H NMR** (400 MHz, $CDCl_3$) δ 7.69 (d, *J* = 3.7 Hz, 1H), 7.50 (d, *J* = 3.7 Hz, 1H), 3.75 (s, 4H), 2.55 (s, 3H), 1.01 (s, 6H) ppm.
^{13}**C NMR** (101 MHz, $CDCl_3$) δ 190.9 (C), 148.7 (C), 135.9 (CH), 132.9 (CH), 73.1 (CH_2), 32.1 (C), 27.5 (CH_3), 21.9 (CH_3) ppm.
Spectral data were consistent with literature.[1]

1-(5-(phenylthio)thiophen-2-yl)ethenone

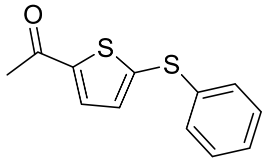

The compound (CAS: 90680-26-5) was prepared according to the general procedure using 1-(5-chlorothiophen-2-yl)ethenone (3.2 mg, 20 µmol, 1.0 equiv.), bis(pinacolato)diboron (50.6 mg, 200 µmol, 10.0 equiv.), diphenyldisulfide (4.4 mg, 80 µmol, 1.0 equiv.) dodecanenitrile (4.4 µL, 20 µmol, 1.0 equiv.) as internal standard and DIPEA (4.2 µL, 24 µmol, 1.2 equiv.) and G1 (10g/L). The reaction mixture was irradiated for 3 hours, obtaining 78% product yield according to GC-FID analysis.

^{1}**H NMR** (400 MHz, $CDCl_3$) δ 7.56 (d, *J* = 3.9 Hz, 1H), 7.43 – 7.38 (m, 2H), 7.36 – 7.28 (m, 3H), 7.10 (d, *J* = 3.9 Hz, 1H), 2.50 (s, 3H) ppm.
^{13}**C NMR** (101 MHz, $CDCl_3$) δ 190.0 (C), 146.3 (C), 145.7 (C), 135.2 (C), 132.8 (CH), 132.1 (CH), 130.9 (CH), 129.6 (CH), 128.2 (CH), 26.6 (CH_3) ppm.
Spectral data were consistent with literature.[1]

2ba. 5-(4,4,5,5-tetramethyl-1,3,2-dioxaborolan-2-yl)thiophene-2-carbaldehyde

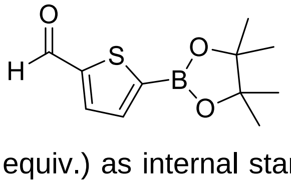

The compound (CAS 1040281-83-1) was prepared according to the general procedure using 5-bromothiophene-2-carbaldehyde (2.5 µL, 20 µmol, 1.0 equiv.), bis(pinacolato)diboron (50.6 mg, 200 µmol, 10.0 equiv.), dodecanenitrile (4.4 µL, 20 µmol, 1.0 equiv.) as internal standard and DIPEA (4.2 µL, 24 µmol, 1.2 equiv.) and G1 (10 g/L). The reaction mixture was irradiated for 3 hours, obtaining 66% product yield according to GC-FID analysis (52% isolated yield as white powder).

^{1}**H NMR** (400 MHz, $CDCl_3$) δ 9.97 (s, 1H), 7.79 (d, *J* = 3.7 Hz, 1H), 7.65 (d, *J* = 3.7 Hz, 1H), 1.35 (s, 12H).
^{13}**C NMR** (101 MHz, $CDCl_3$) δ 183.1 (C), 149.1 (C), 137.4 (CH), 136.2 (CH), 85.0 (C), 24.9 (CH_3) ppm.
Spectral data were consistent with literature.[1]

2bb. 5-(4,4,6-trimethyl-1,3,2-dioxaborinan-2-yl)thiophene-2-carbaldehyde

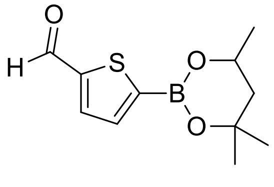

The compound (CAS 2414250-11-4) was prepared according to the general procedure using 5-bromothiophene-2-carbaldehyde (2.5 µL, 20 µmol, 1.0 equiv.), bis(hexyleneglycolato)diboron (53.0 mg, 200 µmol, 10.0 equiv.), dodecanenitrile (4.4 µL, 20 µmol, 1.0 equiv.) as internal standard and DIPEA (4.2 µL, 24 µmol, 1.2 equiv.) and G1 (10 g/L). The reaction mixture was irradiated for 3 hours, obtaining 62% product yield according to GC-FID analysis (49% isolated yield as white powder).

^{1}H NMR (400 MHz, $CDCl_3$) δ 9.87 (s, 1H), 7.69 (d, *J* = 3.7 Hz, 1H), 7.49 (d, *J* = 3.7 Hz, 1H), 4.29 (ddd, *J* = 11.6, 6.1, 2.9 Hz, 1H), 1.82 (dd, *J* = 14.1, 2.9 Hz, 1H), 1.60 – 1.51 (m, 1H), 1.30 (s, 6H), 1.27 (d, *J* = 6.2 Hz, 3H) ppm.
^{13}C NMR (101 MHz, $CDCl_3$) δ 183.1 (C), 147.8 (C), 136.2 (CH), 135.5 (CH), 71.9 (C), 65.7 (CH), 45.6 (CH_2), 31.0 (CH_3), 27.7 (CH_3), 22.9 (CH_3) ppm.
Spectral data were consistent with literature.[1]

2bc. 5-(4,4,6,6-tetramethyl-1,3,2-dioxaborinan-2-yl)thiophene-2-carbaldehyde

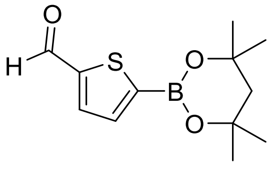

The compound (CAS 2414250-16-9) was prepared according to the general procedure using 5-bromothiophene-2-carbaldehyde (2.5 µL, 20 µmol, 1.0 equiv.), 4,4,4',4',6,6,6',6'-octamethyl-2,2'-bi(1,3,2-dioxaborinane) (56.4 mg, 200 µmol, 10.0 equiv.), dodecanenitrile (4.4 µL, 20 µmol, 1.0 equiv.) as internal standard and DIPEA (4.2 µL, 24 µmol, 1.2 equiv.) and G1 (10 g/L). The reaction mixture was irradiated for 5 hours, obtaining 89% product yield according to GC-FID analysis (73% isolated yield as white powder).

^{1}H NMR (400 MHz, $CDCl_3$) δ 9.93 (s, 1H), 7.75 (d, *J* = 3.6 Hz, 1H), 7.57 (d, *J* = 3.6 Hz, 1H), 1.92 (s, 2H), 1.41 (s, 12H) ppm.
^{13}C NMR (101 MHz, $CDCl_3$) δ 183.3 (C), 147.9 (C), 136.3 (CH), 135.5 (CH), 72.0 (C), 49.1 (CH_2), 31.7 (CH_3) ppm.
Spectral data were consistent with literature.[1]

2bd. 5-(5,5-dimethyl-1,3,2-dioxaborinan-2-yl)thiophene-2-carbaldehyde

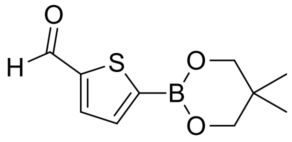

The compound (CAS 2414250-21-6) was prepared according to the general procedure using 5-bromothiophene-2-carbaldehyde (2.5 µL, 20 µmol, 1.0 equiv.), bis(neopentylglycolato)diboron (45.2 mg, 200 µmol, 10.0 equiv.), dodecanenitrile (4.4 µL, 20 µmol, 1.0 equiv.) as internal standard and DIPEA (4.2 µL, 24 µmol, 1.2 equiv.) and G1 (10 g/L). The reaction mixture was irradiated for 2 hours, obtaining 56% product yield according to GC-FID analysis (41% isolated yield as white powder).

^{1}H NMR (400 MHz, $CDCl_3$) δ 9.96 (s, 1H), 7.78 (d, *J* = 3.7 Hz, 1H), 7.59 (d, *J* = 3.7 Hz, 1H), 3.78 (s, 4H), 1.03 (s, 6H) ppm.
^{13}C NMR (101 MHz, $CDCl_3$) δ 183.3 (C), 148.3 (C), 136.4 (CH), 136.0 (CH), 72.7 (CH_2), 32.2 (C), 22.0 (CH_3) ppm.
Spectral data were consistent with literature.[1]

2ca. Methyl 5-(4,4,5,5-tetramethyl-1,3,2-dioxaborolan-2-yl)thiophene-2-carboxylate

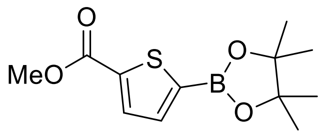

The compound (CAS 916138-13-1) was prepared according to the general procedure using methyl 5-bromothiophene-2-carboxylate (2.6 µL, 20 µmol, 1.0 equiv.), bis(pinacolato)diboron (50.6 mg, 200 µmol, 10.0 equiv.), dodecanenitrile (4.4 µL, 20 µmol, 1.0 equiv.) as internal standard and DIPEA (4.2 µL, 24 µmol, 1.2 equiv.) and G1 (10 g/L). The reaction mixture was irradiated for 20 hours, obtaining 26% product yield according to GC-FID analysis (19% isolated yield as white powder).
Spectral data were consistent with literature.[2]

^{1}H NMR (400 MHz, $CDCl_3$) δ 7.80 (d, J = 3.7 Hz, 1H), 7.54 (d, J = 3.6 Hz, 1H), 3.88 (s, 3H), 1.34 (s, 12H) ppm.
^{13}C NMR (101 MHz, $CDCl_3$) δ 162.8 (C), 139.5 (C), 137.0 (CH), 134.1 (CH), 84.7 (C), 52.4 (O-CH_3), 24.9 (CH_3) ppm.

2cb. Methyl 5-(4,4,6-trimethyl-1,3,2-dioxaborinan-2-yl)thiophene-2-carboxylate

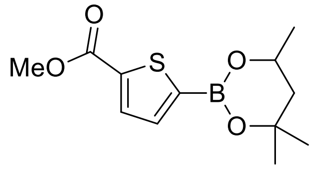

The compound was prepared according to the general procedure using methyl 5-bromothiophene-2-carboxylate (2.6 µL, 80 µmol, 1.0 equiv.), bis(hexyleneglycolato)diboron (53.0 mg, 200 µmol, 10.0 equiv.), dodecanenitrile (4.4 µL, 20 µmol, 1.0 equiv.) as internal standard and DIPEA (4.2 µL, 24 µmol, 1.2 equiv.) and G1 (10 g/L). The reaction mixture was irradiated for 19 hours, obtaining 30% product yield according to GC-FID analysis (25% isolated yield as white powder).

^{1}H NMR (400 MHz, $CDCl_3$) δ 7.77 (d, J = 3.6 Hz, 1H), 7.47 (d, J = 3.6 Hz, 1H), 4.35 (ddd, J = 11.6, 6.0, 2.9 Hz, 1H), 3.87 (s, 3H), 1.87 (dd, J = 14.0, 2.9 Hz, 1H), 1.56 – 1.44 (m, 1H), 1.36 (s, 6H), 1.33 (d, J = 6.2 Hz, 3H) ppm.
^{13}C NMR (101 MHz, $CDCl_3$) δ 163.1 (C), 138.0 (C), 135.2 (CH), 134.0 (CH), 72.0 (C), 65.7 (CH), 52.2 (O-CH_3), 46.1 (CH_2) 31.05 (CH_3), 28.2 (CH_3), 21.18 (CH_3) ppm.
GC-MS (EI): m/z (relative intensity): 268.1 (40) [M+•], 253.1 (35), 237.1 (20), 169.0 (20), 137.0 (20), 43.1 (100).

2cc. Methyl 5-(4,4,6,6-tetramethyl-1,3,2-dioxaborinan-2-yl)thiophene-2-carboxylate

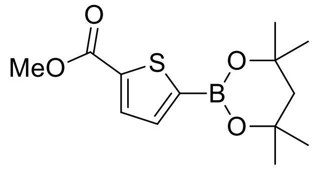

The compound was prepared according to the general procedure using methyl 5-bromothiophene-2-carboxylate (2.6 µL, 80 µmol, 1.0 equiv.), 4,4,4',4',6,6,6',6'-octamethyl-2,2'-bi(1,3,2-dioxaborinane) (56.4 mg, 200 µmol, 10.0 equiv.), dodecanenitrile (4.4 µL, 20 µmol, 1.0 equiv.) as internal standard and DIPEA (4.2 µL, 24 µmol, 1.2 equiv.) and G1 (10 g/L). The reaction mixture was irradiated for 5 hours, obtaining 23% product yield according to GC-FID analysis (15% isolated yield as white powder).

^{1}H NMR (400 MHz, $CDCl_3$) δ 7.77 (d, J = 3.6 Hz, 1H), 7.48 (d, J = 3.6 Hz, 1H), 3.87 (s, 3H), 1.92 (s, 2H), 1.41 (s, 12H) ppm.
^{13}C NMR (101 MHz, $CDCl_3$) δ 163.03 (C), 137.96 (C), 135.18 (CH), 134.00 (CH), 119.97 (C), 71.81 (C), 52.17 (O-CH_3), 49.14 (CH_2), 31.76 (CH_3) ppm.

[2] Ghayoor A. Chotana, Venkata A. Kallepalli, Robert E. Maleczka Jr., Milton R. Smith III. *Tetrahedron* **2008**, *64*, 6103–6114.

GC-MS (EI): m/z (relative intensity): 282.1 (100) [M+•], 267.1 (100), 251.1 (30), 183.0 (50), 169.0 (50), 137.0 (60), 99.1 (40), 56.1 (80), 43.1 (80).

2cd. Methyl 5-(5,5-dimethyl-1,3,2-dioxaborinan-2-yl)thiophene-2-carboxylatecarboxylate

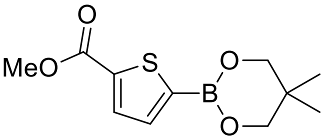

The compound was prepared according to the general procedure using methyl 5-bromothiophene-2-carboxylate (2.6 µL, 80 µmol, 1.0 equiv.), bis(neopentylglycoltao)diboron (45.2 mg, 200 µmol, 10.0 equiv.), dodecanenitrile (4.4 µL, 20 µmol, 1.0 equiv.) as internal standard and DIPEA (4.2 µL, 24 µmol, 1.2 equiv.) and G1 (10 g/L). The reaction mixture was irradiated for 5 hours, obtaining 24% product yield according to GC-FID analysis (16% isolated yield as rose-pale powder).

^{1}H NMR (400 MHz, $CDCl_3$) δ 7.79 (d, J = 3.6 Hz, 1H), 7.49 (d, J = 3.6 Hz, 1H), 3.88 (s, 3H), 3.76 (s, 4H), 1.03 (s, 6H) ppm.
^{13}C NMR (101 MHz, $CDCl_3$) δ 163.3 (C), 135.7 (C), 134.1 (CH), 129.2 (CH), 72.6 (CH_2), 52.3 (O-CH_3), 32.2 (C), 22.0 (CH_3) ppm.
GC-MS (EI): m/z (relative intensity): 254.1 (40) [M+•], 223.1 (100), 136.9 (25), 111.0 (20), 70.1 (20), 61.0 (20), 43.1 (100).

2da. 1-(5-(4,4,5,5-tetramethyl-1,3,2-dioxaborolan-2-yl)thiophen-2-yl)propan-1-one

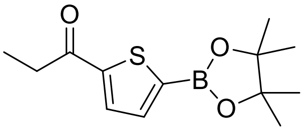

The compound (CAS 2414250-08-9) was prepared according to the general procedure using 1-(5-bromothiophen-2-yl)propan-1-one (4.4 mg, 20 µmol, 1.0 equiv.), bis(pinacolato)diboron (50.6 mg, 200 µmol, 10.0 equiv.), dodecanenitrile (4.4 µL, 20 µmol, 1.0 equiv.) as internal standard and DIPEA (4.2 µL, 24 µmol, 1.2 equiv.) and G1 (10 g/L). The reaction mixture was irradiated for 3 hours, obtaining 74% product yield according to GC-FID analysis (62% isolated yield as white powder).

^{1}H NMR (400 MHz, $CDCl_3$) δ 7.71 (d, J = 3.7 Hz, 1H), 7.55 (d, J = 3.7 Hz, 1H), 2.92 (q, J = 7.3 Hz, 2H), 1.32 (s, 12H), 1.21 – 1.17 (t, 3H) ppm.
^{13}C NMR (101 MHz, $CDCl_3$) δ 193.9 (C), 149.2 (C), 137.3 (CH), 132.0 (CH), 84.7 (C), 33.2 (CH_2), 24.6 (CH_3), 8.5 (CH_3) ppm.
Spectral data were consistent with literature.[1]

2db. 1-(5-(4,4,6-trimethyl-1,3,2-dioxaborinan-2-yl)thiophen-2-yl)propan-1-one

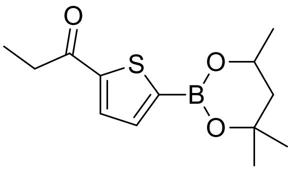

The compound (CAS 2414250-12-5) was prepared according to the general procedure using 1-(5-bromothiophen-2-yl)propan-1-one (4.4 mg, 20 µmol, 1.0 equiv.), bis(hexyleneglycolato)diboron (53.0 mg, 200 µmol, 10.0 equiv.), dodecanenitrile (4.4 µL, 20 µmol, 1.0 equiv.) as internal standard and DIPEA (4.2 µL, 24 µmol, 1.2 equiv.) and G1 (10 g/L). The reaction mixture was irradiated for 3 hours, obtaining 81% product yield according to GC-FID analysis (69% isolated yield as white powder).

^{1}H NMR (400 MHz, $CDCl_3$) δ 7.68 (d, J = 3.7 Hz, 1H), 7.48 (d, J = 3.7 Hz, 1H), 4.33 (dqd, J = 12.4, 6.2, 2.9 Hz, 1H), 2.92 (q, J = 7.3 Hz, 2H), 1.86 (dd, J = 14.0, 2.9 Hz, 1H), 1.60 (dd, J = 13.9, 11.7 Hz, 1H), 1.35 (s, 6H), 1.32 (d, J = 6.2 Hz, 3H), 1.19 (t, J = 7.3 Hz, 3H) ppm.

^{13}C NMR (101 MHz, $CDCl_3$) δ 194.1 (C), 148.0 (C), 135.5 (CH), 132.0 (CH), 72.0 (C), 65.7 (CH), 46.1 (CH_2), 33.1 (CH_2), 31.2 (CH_3), 28.1 (CH_3), 8.6 (CH_3) ppm.
Spectral data were consistent with literature.[1]

2dc. 1-(5-(4,4,6,6-tetramethyl-1,3,2-dioxaborinan-2-yl)thiophen-2-yl)propan-1-one

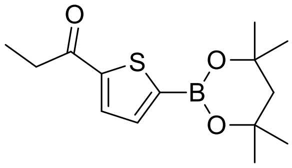

The compound (CAS 2414250-17-0) was prepared according to the general procedure using 1-(5-bromothiophen-2-yl)propan-1-one (4.4 mg, 20 µmol, 1.0 equiv.), 4,4,4',4',6,6,6',6'-octamethyl-2,2'-bi(1,3,2-dioxaborinane) (56.4 mg, 200 µmol, 10.0 equiv.), dodecanenitrile (4.4 µL, 20 µmol, 1.0 equiv.) as internal standard and DIPEA (4.2 µL, 24 µmol, 1.2 equiv.) and G1 (10 g/L). The reaction mixture was irradiated for 3 hours, obtaining 82% product yield according to GC-FID analysis (67% isolated yield as white powder).

^{1}H NMR (400 MHz, $CDCl_3$) δ 7.71 (d, *J* = 3.7 Hz, 1H), 7.51 (d, *J* = 3.7 Hz, 1H), 2.94 (d, *J* = 7.3 Hz, 2H), 1.93 (s, 2H), 1.42 (s, 12H), 1.22 (t, *J* = 7.3 Hz, 3H) ppm.
^{13}C NMR (101 MHz, $CDCl_3$) δ 194.2 (C), 147.9 (C), 135.5 (CH), 132.1 (CH), 71.9 (C), 49.2 (CH_2), 33.2 (CH_3), 8.7 (CH_3) ppm.
Spectral data were consistent with literature.[1]

2dd. 1-(5-(5,5-dimethyl-1,3,2-dioxaborinan-2-yl)thiophen-2-yl)propan-1-one

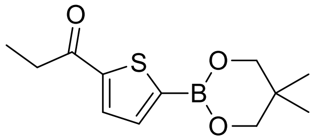

The compound (CAS 2414250-22-7) was prepared according to the general procedure using 1-(5-bromothiophen-2-yl)propan-1-one (4.4 mg, 20 µmol, 1.0 equiv.), bis(neopentylglycolato)diboron (45.2 mg, 200 µmol, 10.0 equiv.), dodecanenitrile (4.4 µL, 20 µmol, 1.0 equiv.) as internal standard and DIPEA (4.2 µL, 24 µmol, 1.2 equiv.) and G1 (10 g/L). The reaction mixture was irradiated for 4 hours, obtaining 58% product yield according to GC-FID analysis (43% isolated yield as white powder).

^{1}H NMR (400 MHz, $CDCl_3$) δ 7.72 (d, *J* = 3.7 Hz, 1H), 7.51 (d, *J* = 3.7 Hz, 1H), 3.76 (s, 4H), 2.94 (q, *J* = 7.3 Hz, 2H), 1.22 (t, *J* = 7.3 Hz, 3H), 1.03 (s, 6H) ppm.
^{13}C NMR (101 MHz, $CDCl_3$) δ 194.1 (C), 148.4 (C), 135.9 (CH), 132.1 (CH), 72.6 (CH_2), 33.2 (CH_2), 32.2 (C), 22.0 (CH_3), 8.6 (CH_3) ppm.
Spectral data were consistent with literature.[1]

2ea. 2-methyl-1-(5-(4,4,5,5-tetramethyl-1,3,2-dioxaborolan-2-yl)thiophen-2-yl)propan-1-one

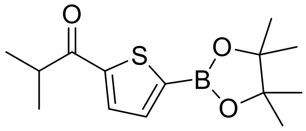

The compound (CAS 2414250-09-0) was prepared according to the general procedure using 1-(5-bromothiophen-2-yl)-2-methylpropan-1-one (3.2 µL, 20 µmol, 1.0 equiv.), bis(pinacolato)diboron (50.6 mg, 200 µmol, 10.0 equiv.), dodecanenitrile (4.4 µL, 20 µmol, 1.0 equiv.) as internal standard and DIPEA (4.2 µL, 24 µmol, 1.2 equiv.) and G1 (10 g/L). The reaction mixture was irradiated for 3 hours, obtaining 77% product yield according to GC-FID analysis (61% isolated yield as white powder).

^{1}H NMR (400 MHz, $CDCl_3$) δ 7.75 (d, *J* = 3.7 Hz, 1H), 7.58 (d, *J* = 3.7 Hz, 1H), 3.36 (dp, *J* = 13.7, 6.8 Hz, 1H), 1.34 (s, 12H), 1.23 (t, *J* = 4.4 Hz, 3H) ppm.
^{13}C NMR (101 MHz, $CDCl_3$) δ 197.6 (C), 148.7 (C), 137.4 (CH), 132.2 (CH), 84.8 (C), 38.0 (CH), 24.9 (CH_3), 19.5 (CH_3) ppm.

Spectral data were consistent with literature.[1]

2eb. 2-methyl-1-(5-(4,4,6-trimethyl-1,3,2-dioxaborinan-2-yl)thiophen-2-yl)propan-1-one

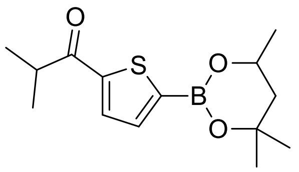

The compound (CAS 2414250-13-6) was prepared according to the general procedure using 1-(5-bromothiophen-2-yl)-2-methylpropan-1-one (3.2 µL, 20 µmol, 1.0 equiv.), bis(hexyleneglycolato)diboron (53.0 mg, 200 µmol, 10.0 equiv.), dodecanenitrile (4.4 µL, 20 µmol, 1.0 equiv.) as internal standard and DIPEA (4.2 µL, 24 µmol, 1.2 equiv.) and G1 (10 g/L). The reaction mixture was irradiated for 3 hours, obtaining 78% product yield according to GC-FID analysis (66% isolated yield as white powder).

^{1}H NMR (400 MHz, $CDCl_3$) δ 7.67 (d, J = 3.7 Hz, 1H), 7.46 (d, J = 3.7 Hz, 1H), 4.30 (ddd, J = 11.6, 6.1, 2.9 Hz, 1H), 3.33 (dt, J = 13.7, 6.8 Hz, 1H), 1.83 (dd, J = 14.0, 2.9 Hz, 1H), 1.57 (dd, J = 13.9, 11.7 Hz, 1H), 1.32 (s, 6H), 1.29 (d, J = 6.2 Hz, 3H), 1.17 – 1.12 (m, 6H) ppm.
^{13}C NMR (101 MHz, $CDCl_3$) δ 197.5 (C), 147.3 (C), 135.5 (CH), 132.0 (CH), 71.9 (C), 65.6 (CH), 46.0 (CH_2), 37.7 (CH), 31.1 (CH_3), 28.1 (CH_3), 23.0 (CH_3), 19.4 (CH_3) ppm.
Spectral data were consistent with literature.[1]

2ec. 2-methyl-1-(5-(4,4,6,6-tetramethyl-1,3,2-dioxaborinan-2-yl)thiophen-2-yl)propan-1-one

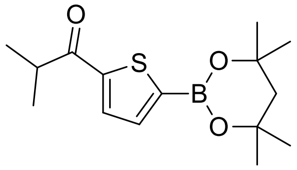

The compound (CAS 2414250-18-1) was prepared according to the general procedure using 1-(5-bromothiophen-2-yl)-2-methylpropan-1-one (3.2 µL, 20 µmol, 1.0 equiv.), 4,4,4',4',6,6,6',6'-octamethyl-2,2'-bi(1,3,2-dioxaborinane) (56.4 mg, 200 µmol, 10.0 equiv.), dodecanenitrile (4.4 µL, 20 µmol, 1.0 equiv.) as internal standard and DIPEA (4.2 µL, 24 µmol, 1.2 equiv.) and G1 (10 g/L). The reaction mixture was irradiated for 3 hours, obtaining 82% product yield according to GC-FID analysis (66% isolated yield as white powder).

^{1}H NMR (400 MHz, $CDCl_3$) δ 7.72 (d, J = 3.7 Hz, 1H), 7.52 (d, J = 3.7 Hz, 1H), 3.38 (dt, J = 13.7, 6.8 Hz, 1H), 1.93 (s, 2H), 1.42 (s, 12H), 1.23 (d, J = 6.8 Hz, 6H) ppm.
^{13}C NMR (101 MHz, $CDCl_3$) δ 197.8 (C), 147.4 (C), 135.6 (CH), 132.2 (CH), 71.9 (C), 49.2 (CH_2), 37.8 (CH), 31.8 (CH_3), 19.6 (CH_3) ppm.
Spectral data were consistent with literature.[1]

2ed. 1-(5-(5,5-dimethyl-1,3,2-dioxaborinan-2-yl)thiophen-2-yl)-2-methylpropan-1-one

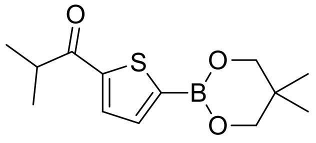

The compound (CAS 2414250-23-8) was prepared according to the general procedure using 1-(5-bromothiophen-2-yl)-2-methylpropan-1-one (3.2 µL, 20 µmol, 1.0 equiv.), bis(neopentylglycolato)diboron (45.2 mg, 200 µmol, 10.0 equiv.), dodecanenitrile (4.4 µL, 20 µmol, 1.0 equiv.) as internal standard and DIPEA (4.2 µL, 24 µmol, 1.2 equiv.) and G1 (10 g/L). The reaction mixture was irradiated for 2 hours, obtaining 54% product yield according to GC-FID analysis (40% isolated yield as white powder).

^{1}H NMR (400 MHz, $CDCl_3$) δ 7.74 (d, J = 3.7 Hz, 1H), 7.52 (d, J = 3.7 Hz, 1H), 3.77 (s, 4H), 3.43 – 3.25 (m, 1H), 1.23 (d, J = 6.8 Hz, 6H), 1.03 (s, 6H) ppm.

^{13}C NMR (101 MHz, $CDCl_3$) δ 197.7 (C), 147.8 (C), 136.0 (CH), 132.3 (C), 72.6 (CH_2), 37.9 (CH), 32.2 (C), 22.0 (CH_3), 19.5 (CH_3) ppm.
Spectral data were consistent with literature.[1]

2fa. Phenyl(5-(4,4,5,5-tetramethyl-1,3,2-dioxaborolan-2-yl)thiophen-2-yl)methanone

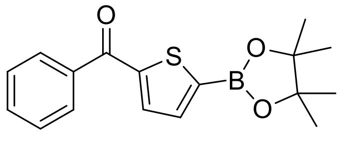

The compound (CAS 1220107-42-5) was prepared according to the general procedure using (5-bromothiophen-2-yl)(phenyl)methanone (5.5 mg, 20 µmol, 1.0 equiv.), bis(pinacolato)diboron (50.6 mg, 200 µmol, 10.0 equiv.), dodecanenitrile (4.4 µL, 20 µmol, 1.0 equiv.) as internal standard and DIPEA (4.2 µL, 24 µmol, 1.2 equiv.) and G1 (10 g/L). The reaction mixture was irradiated for 3 hours, obtaining 68% product yield according to GC-FID analysis (51% isolated yield as yellow-pale powder).

^{1}H NMR (400 MHz, $CDCl_3$) δ 7.91 – 7.84 (m, 2H), 7.70 (d, *J* = 3.7 Hz, 1H), 7.61 (d, *J* = 3.7 Hz, 1H), 7.57 (d, *J* = 7.5 Hz, 1H), 7.48 (t, *J* = 7.5 Hz, 2H), 1.35 (s, 12H) ppm.
^{13}C NMR (101 MHz, $CDCl_3$) δ 188.8 (C), 148.6 (C), 138.4 (C), 137.1 (CH), 135.0 (CH), 132.5 (CH), 129.4 (2xCH), 128.5 (2xCH), 84.8 (C), 24.7 (CH_3) ppm.
Spectral data were consistent with literature.[1]

2fb. Phenyl(5-(4,4,6-trimethyl-1,3,2-dioxaborinan-2-yl)thiophen-2-yl)methanone

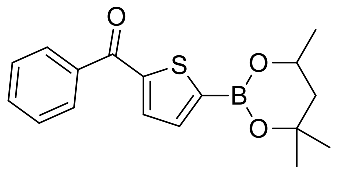

The compound (CAS 2414250-14-7) was prepared according to the general procedure using (5-bromothiophen-2-yl)(phenyl)methanone (5.5 mg, 20 µmol, 1.0 equiv.), bis(hexyleneglycolato)diboron (53.0 mg, 200 µmol, 10.0 equiv.), dodecanenitrile (4.4 µL, 20 µmol, 1.0 equiv.) as internal standard and DIPEA (4.2 µL, 24 µmol, 1.2 equiv.) and G1 (10 g/L). The reaction mixture was irradiated for 3 hours, obtaining 75% product yield according to GC-FID analysis (61% isolated yield as yellow-pale powder).

^{1}H NMR (400 MHz, $CDCl_3$) δ 7.77 (d, *J* = 7.5 Hz, 2H), 7.57 (d, *J* = 3.6 Hz, 1H), 7.49 (t, *J* = 7.1 Hz, 1H), 7.46 – 7.43 (m, 1H), 7.39 (t, *J* = 7.5 Hz, 2H), 4.35 – 4.21 (m, 1H), 1.81 (dd, *J* = 14.0, 2.7 Hz, 1H), 1.59 – 1.49 (m, 1H), 1.29 (s, 6H), 1.26 (d, *J* = 6.2 Hz, 3H) ppm.
^{13}C NMR (101 MHz, $CDCl_3$) δ 188.0 (C), 147.1 (C), 138.3 (CH), 135.2 (CH), 134.8 (CH), 132.1 (CH), 129.1 (2xCH), 128.2 (2xCH), 71.8 (C), 65.5 (CH), 45.6 (CH_2), 31.0 (CH_3), 27.6 (CH_3), 22.9 (CH_3) ppm.
Spectral data were consistent with literature.[1]

2fc. Phenyl(5-(4,4,6,6-tetramethyl-1,3,2-dioxaborinan-2-yl)thiophen-2-yl)methanone

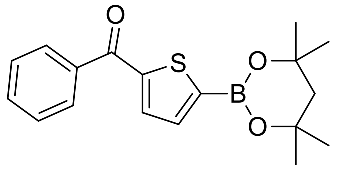

The compound (CAS 2414250-19-2) was prepared according to the general procedure using (5-bromothiophen-2-yl)(phenyl)methanone (5.5 mg, 20 µmol, 1.0 equiv.), 4,4,4',4',6,6,6',6'-octamethyl-2,2'-bi(1,3,2-dioxaborinane) (56.4 mg, 200 µmol, 10.0 equiv.), dodecanenitrile (4.4 µL, 20 µmol, 1.0 equiv.) as internal standard and DIPEA (4.2 µL, 24 µmol, 1.2 equiv.) and G1 (10 g/L). The reaction mixture was irradiated for 3 hours, obtaining 81% product yield according to GC-FID analysis (70% isolated yield as yellow-pale powder).

^{1}H NMR (400 MHz, $CDCl_3$) δ 7.91 – 7.84 (m, 2H), 7.67 (d, *J* = 3.7 Hz, 1H), 7.57 (dd, *J* = 12.3, 5.5 Hz, 2H), 7.48 (t, *J* = 7.5 Hz, 2H), 1.94 (s, 2H), 1.43 (s, 12H) ppm.
^{13}C NMR (101 MHz, $CDCl_3$) δ 188.5 (C), 147.3 (C), 138.7 (C), 135.3 (CH), 132.3 (CH), 129.4 (2xCH), 128.5 (2xCH), 71.9 (C), 49.2 (CH_2), 31.8 (CH_3) ppm.
Spectral data were consistent with literature.[1]

2fd. (5-(5,5-dimethyl-1,3,2-dioxaborinan-2-yl)thiophen-2-yl)(phenyl)methanone

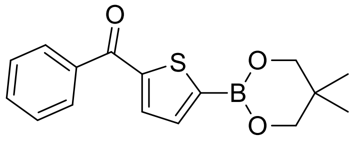

The compound (CAS 2414250-24-9) was prepared according to the general procedure using (5-bromothiophen-2-yl)(phenyl)methanone (5.5 mg, 20 µmol, 1.0 equiv.), bis(neopentylglycoltao)diboron (45.2 mg, 200 µmol, 10.0 equiv.), dodecanenitrile (4.4 µL, 20 µmol, 1.0 equiv.) as internal standard and DIPEA (4.2 µL, 24 µmol, 1.2 equiv.) and G1 (10 g/L). The reaction mixture was irradiated for 2 hours, obtaining 42% product yield according to GC-FID analysis (35% isolated yield as yellow-pale powder).

^{1}H NMR (400 MHz, CDCl3) δ 7.88 (d, J = 7.4 Hz, 2H), 7.69 (d, J = 3.7 Hz, 1H), 7.57 (dd, J = 12.0, 5.5 Hz, 2H), 7.48 (t, J = 7.6 Hz, 2H), 3.78 (s, 4H), 1.04 (s, 6H) ppm.
^{13}C NMR (101 MHz, $CDCl_3$) δ 188.4 (C), 147.7 (C), 138.5 (C), 135.7 (CH), 135.2 (CH), 132.4 (CH), 129.4 (CH), 128.5 (CH), 72.7 (CH_2), 32.2 (C), 22.0 (CH_3) ppm.
Spectral data were consistent with literature.[1]

<u>FURANS</u>

3aa. 1-(5-(4,4,5,5-tetramethyl-1,3,2-dioxaborolan-2-yl)furan-2-yl)ethanone

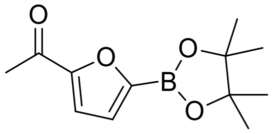

The compound was prepared according to the general procedure using 1-(5-bromofuran-2-yl)ethanone (3.8 mg, 20 µmol, 1.0 equiv.), bis(pinacolato)diboron (50.6 mg, 200 µmol, 10.0 equiv.), dodecanenitrile (4.4 µL, 20 µmol, 1.0 equiv.) as internal standard and DIPEA (4.2 µL, 24 µmol, 1.2 equiv.) and G1 (10 g/L). The reaction mixture was irradiated for 22 hours, obtaining 56% product yield according to GC-FID analysis (45% isolated yield as yellow-pale powder).

^{1}H NMR (400 MHz, $CDCl_3$) δ 7.17 (d, J = 3.5 Hz, 1H), 7.09 (d, J = 3.5 Hz, 1H), 2.54 (s, 3H), 1.36 (s, J = 5.3 Hz, 12H) ppm.
^{13}C NMR (101 MHz, $CDCl_3$) δ 188.6 (C), 163.2 (C), 124.5 (CH), 116.5 (CH), 84.9 (C), 26.6 (CH_3), 24.8 (CH_3) ppm.
HRMS (EI): m/z $(M+H)^+$ = calcd. for $C_{12}H_{18}BO_4$: 237.1293, found: 237.1297.

3ab. 1-(5-(4,4,6-trimethyl-1,3,2-dioxaborinan-2-yl)furan-2-yl)ethanone

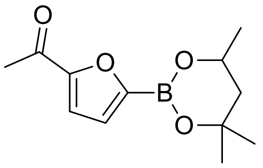

The compound was prepared according to the general procedure using 1-(5-bromofuran-2-yl)ethenone (3.8 mg, 80 µmol, 1.0 equiv.), bis(hexyleneglycolato)diboron (53.0 mg, 200 µmol, 10.0 equiv.), dodecanenitrile (4.4 µL, 20 µmol, 1.0 equiv.) as internal standard and DIPEA (4.2 µL, 24 µmol, 1.2 equiv.) and G1 (10 g/L). The reaction mixture was irradiated for 6 hours, obtaining 85% product yield according to GC-FID analysis (63% isolated yield as white powder).

^{1}H NMR (400 MHz, $CDCl_3$) δ 7.14 (d, J = 3.5 Hz, 1H), 6.97 (d, J = 3.5 Hz, 1H), 4.36 (ddd, J = 11.6, 6.1, 2.9 Hz, 1H), 2.52 (s, 3H), 1.88 (dd, J = 14.1, 2.9 Hz, 1H), 1.65-1.59 (m, 1H), 1.37 (d, J = 2.4 Hz, 6H), 1.35 (d, J = 6.2 Hz, 3H) ppm.

^{13}C NMR (101 MHz, $CDCl_3$) δ 187.8 (C), 155.8 (C), 122.6 (CH), 117.0 (CH), 72.2 (C), 65.8 (CH), 46.2 (CH_2), 31.2 (CH_3), 28.2 (CH_3), 23.1 (CH_3) ppm.
HRMS (EI): m/z $(M+H)^+$ = calcd. for $C_{12}H_{17}BO_4$: 236.0706, found: 236.0711.

3ac. 1-(5-(4,4,6,6-tetramethyl-1,3,2-dioxaborinan-2-yl)furan-2-yl)ethanone

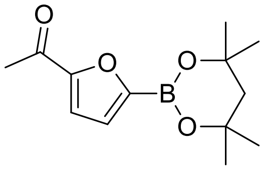

The compound was prepared according to the general procedure using 1-(5-bromofuran-2-yl)ethanone (3.8 mg, 80 µmol, 1.0 equiv.), 4,4,4',4',6,6,6',6'-octamethyl-2,2'-bi(1,3,2-dioxaborinane) (56.4 mg, 200 µmol, 10.0 equiv.), dodecanenitrile (4.4 µL, 20 µmol, 1.0 equiv.) as internal standard and DIPEA (4.2 µL, 24 µmol, 1.2 equiv.) and G1 (10 g/L). The reaction mixture was irradiated for 6 hours, obtaining 73% product yield according to GC-FID analysis (60% isolated yield as yellow-pale powder).

^{1}H NMR (400 MHz, $CDCl_3$) δ 7.10 (d, J = 3.5 Hz, 1H), 6.93 (d, J = 3.5 Hz, 1H), 2.46 (s, 3H), 1.88 (s, 2H), 1.37 (s, 12H) ppm.
^{13}C NMR (101 MHz, $CDCl_3$) δ 187.7 (C), 155.6 (C), 122.4 (CH), 117.0 (CH), 71.8 (C), 49.2 (CH_2), 31.6 (CH_3), 26.3 (CH_3) ppm.
GC-MS (EI): m/z (relative intensity): 250.1 (20) [M+•], 235.1 (30), 251.1 (30), 151.1 (20), 137.0 (20), 70.1 (20), 61.1 (20), 56.1 (10), 43.1 (100).

3ad. 1-(5-(5,5-dimethyl-1,3,2-dioxaborinan-2-yl)furan-2-yl)ethanone

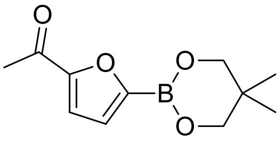

The compound was prepared according to the general procedure using 1-(5-bromofuran-2-yl)ethanone (3.8 mg, 80 µmol, 1.0 equiv.), bis(neopentylglycoltao)diboron (45.2 mg, 200 µmol, 10.0 equiv.), dodecanenitrile (4.4 µL, 20 µmol, 1.0 equiv.) as internal standard and DIPEA (4.2 µL, 24 µmol, 1.2 equiv.) and G1 (10 g/L). The reaction mixture was irradiated for 20 hours, obtaining 51% product yield according to GC-FID analysis (39% isolated yield as yellow-pale powder).

^{1}H NMR (400 MHz, $CDCl_3$) δ 7.15 (d, J = 3.5 Hz, 1H), 7.00 (d, J = 3.5 Hz, 1H), 3.77 (s, 4H), 2.53 (s, 3H), 1.03 (s, 6H) ppm.
^{13}C NMR (101 MHz, $CDCl_3$) δ 187.7 (C), 156.0 (C), 123.0 (CH), 116.8 (CH), 72.5 (C), 32.1 (CH_2), 26.5 (CH_3), 21.4 (CH_3) ppm.
HRMS (EI): m/z $(M+H)^+$ = calcd. for $C_{11}H_{15}BO_4$: 223.1117, found: 223.1125.

1-(5-(phenylthio)furan-2-yl)ethanone

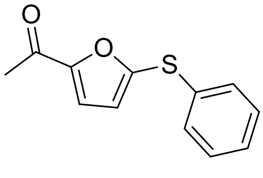

The compound (CAS: 28569-36-0) was prepared according to the general procedure using 1-(5-bromofuran-2-yl)ethanone (3.8 mg, 80 µmol, 1.0 equiv.), bis(pinacolato)diboron (50.6 mg, 200 µmol, 10.0 equiv.), diphenyldisulfide (4.4 mg, 80 µmol, 1.0 equiv.) dodecanenitrile (4.4 µL, 20 µmol, 1.0 equiv.) as internal standard and DIPEA (4.2 µL, 24 µmol, 1.2 equiv.) and G1 (10 g/L). The reaction mixture was irradiated for 3 hours, obtaining 78% product yield according to GC-FID analysis (54% isolated yield as brown-yellow powder).
This compound is commercially available, and the spectral data were consistent with the commercial one. Please find this compound in Merck:
https://www.sigmaaldrich.com/catalog/product/aldrich/t119652?lang=es&region=ES

^{1}H NMR (400 MHz, $CDCl_3$) δ 7.31 (m, 5H), 7.17 (d, J = 3.5 Hz, 1H), 6.63 (d, J = 3.5 Hz, 1H), 2.45 (s, 3H) ppm.
^{13}C NMR (101 MHz, $CDCl_3$) δ 186.5 (C), 155.1 (C), 150.6 (C), 133.2 (C), 130.3 (CH), 129.6 (CH), 127.9 (CH), 118.6 (CH), 118.3 (CH), 26.1 (CH_3) ppm.

3ba. 5-(4,4,5,5-tetramethyl-1,3,2-dioxaborolan-2-yl)furan-2-carbaldehyde

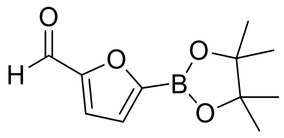

The compound (CAS 273731-82-1) was prepared according to the general procedure using 5-bromofuran-2-carbaldehyde (3.6 mg, 20 µmol, 1.0 equiv.), bis(pinacolato)diboron (50.6 mg, 200 µmol, 10.0 equiv.), dodecanenitrile (4.4 µL, 20 µmol, 1.0 equiv.) as internal standard and DIPEA (4.2 µL, 24 µmol, 1.2 equiv.) and G1 (10 g/L). The reaction mixture was irradiated for 5 hours, obtaining 35% product yield according to GC-FID analysis (23% isolated yield as yellow-pale powder).
Spectral data were consistent with literature.[3]

^{1}H NMR (400 MHz, $CDCl_3$) δ 9.80 (s, 1H), 7.23 (d, J = 3.6 Hz, 1H), 7.13 (d, J = 3.6 Hz, 1H), 1.35 (s, 12H) ppm.
^{13}C NMR (101 MHz, $CDCl_3$) δ 179.4 (C), 156.4 (C), 124.5 (CH), 118.5 (CH), 85.1 (C), 24.9 (CH_3) ppm.

3bb. 5-(4,4,6-trimethyl-1,3,2-dioxaborinan-2-yl)furan-2-carbaldehyde

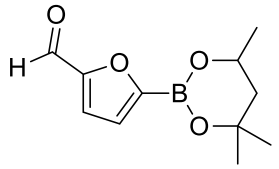

The compound (CAS 1603976-11-9) was prepared according to the general procedure using methyl 5-bromo-2-furaldehyde (3.6 mg, 80 µmol, 1.0 equiv.), bis(hexyleneglycolato)diboron (53.0 mg, 200 µmol, 10.0 equiv.), dodecanenitrile (4.4 µL, 20 µmol, 1.0 equiv.) as internal standard and DIPEA (4.2 µL, 24 µmol, 1.2 equiv.) and G1 (10 g/L). The reaction mixture was irradiated for 20 hours, obtaining 54% product yield according to GC-FID analysis (45% isolated yield as yellow-pale powder).

^{1}H NMR (400 MHz, $CDCl_3$) δ 9.77 (s, 1H), 7.21 (d, J = 3.6 Hz, 1H), 7.03 (d, J = 3.5 Hz, 1H), 4.37 (m, J = 12.3, 6.2, 2.8 Hz, 1H), 1.90 (dd, J = 14.1, 2.9 Hz, 1H), 1.65 (d, J = 12.0 Hz, 1H), 1.39 (d, J = 2.9 Hz, 6H), 1.36 (d, J = 6.2 Hz, 3H) ppm.
^{13}C NMR (101 MHz, $CDCl_3$) δ 179.4 (C), 155.8 (C), 122.6.1 (CH), 119.5 (CH), 72.4 (C), 65.9 (CH), 46.3 (CH_2), 31.2 (CH_3), 28.2 (CH_3), 23.1 (CH_3) ppm.
HRMS (EI): m/z $(M+H)^+$ = calcd. for $C_{11}H_{16}BO_4$: 223.1136, found: 223.1139.

3bc. 5-(4,4,6,6-tetramethyl-1,3,2-dioxaborinan-2-yl)furan-2-carbaldehyde

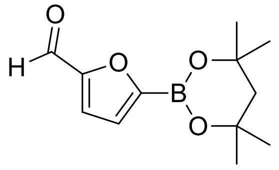

The compound was prepared according to the general procedure using methyl 5-bromo-2-furaldehyde (3.6 mg, 80 µmol, 1.0 equiv.), 4,4,4',4',6,6,6',6'-octamethyl-2,2'-bi(1,3,2-dioxaborinane) (56.4 mg, 200 µmol, 10.0 equiv.), dodecanenitrile (4.4 µL, 20 µmol, 1.0 equiv.) as internal standard and DIPEA (4.2 µL, 24 µmol, 1.2 equiv.) and G1 (10 g/L). The reaction mixture was irradiated for 20 hours, obtaining 66% product yield according to GC-FID analysis (58% isolated yield yellow-pale powder).

^{1}H NMR (400 MHz, $CDCl_3$) δ 9.77 (s, 1H), 7.22 (d, J = 3.5 Hz, 1H), 7.04 (d, J = 3.5 Hz, 1H), 1.95 (s, 2H), 1.44 (s, 12H) ppm.

---

[3] Akitake Yamaguchi, Sherif J. Kaldas, Solomon D. Appavoo, Diego B. Diaz and Andrei K. Yudi. *Chem. Commun.,* **2019**, *55*, 10567-10570.

^{13}C NMR (101 MHz, $CDCl_3$) δ 179.4 (C), 155.8 (C), 126.7 (CH), 119.6 (CH), 72.1 (C), 49.3 (CH_2), 31.8 (CH_3) ppm.
HRMS (EI): m/z $(M+H)^+$ = calcd. for $C_{12}H_{17}BO_4$: 237.1293, found: 237.1299.

3bd. 5-(5,5-dimethyl-1,3,2-dioxaborinan-2-yl)furan-2-carbaldehyde

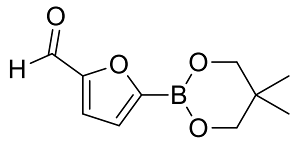

The compound (CAS 1218791-07-1) was prepared according to the general procedure using 5-bromofuran-2-carbaldehyde (3.6 mg, 80 µmol, 1.0 equiv.), bis(neopentylglycoltao)diboron (45.2 mg, 200 µmol, 10.0 equiv.), dodecanenitrile (4.4 µL, 20 µmol, 1.0 equiv.) as internal standard and DIPEA (4.2 µL, 24 µmol, 1.2 equiv.) and G1 (10g/L). The reaction mixture was irradiated for 20 hours, obtaining 80% product yield according to GC-FID analysis (62% isolated yield as yellow-pale powder).

^{1}H NMR (400 MHz, $CDCl_3$) δ 9.78 (s, 1H), 7.22 (d, J = 3.6 Hz, 1H), 7.05 (d, J = 3.6 Hz, 1H), 3.78 (s, 4H), 1.03 (s, 6H) ppm.
^{13}C NMR (101 MHz, $CDCl_3$) δ 179.2 (C), 156.0 (C), 123.0 (CH), 119.0 (CH), 72.6 (C), 32.3 (CH_2), 22.0 (CH_3) ppm.
HRMS (EI): m/z $(M+H)^+$ = calcd. for $C_{10}H_{14}BO_4$: 209.0985, found: 209.0982.

3ca. Methyl 5-(4,4,5,5-tetramethyl-1,3,2-dioxaborolan-2-yl)furan-2-carboxylate

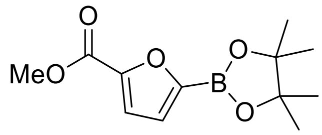

The compound (CAS 676501-87-4) was prepared according to the general procedure using methyl 5-bromofuran-2-carboxylate (4.1 mg, 20 µmol, 1.0 equiv.), bis(pinacolato)diboron (50.6 mg, 200 µmol, 10.0 equiv.), dodecanenitrile (4.4 µL, 20 µmol, 1.0 equiv.) as internal standard and DIPEA (4.2 µL, 24 µmol, 1.2 equiv.) and G1 (10g/L). The reaction mixture was irradiated for 22 hours, obtaining 60% product yield according to GC-FID analysis (51% isolated yield as yellow-pale powder).
Spectral data were consistent with literature.[4]

^{1}H NMR (400 MHz, $CDCl_3$) δ 7.16 (dd, J = 13.5, 3.5 Hz, 1H), 7.07 (d, J = 3.5 Hz, 1H), 3.88 (s, 3H), 1.33 (s, 12H) ppm.
^{13}C NMR (101 MHz, $CDCl_3$) δ 159.3 (C), 148.5 (C), 124.3 (C), 118.1 (C), 84.9 (C), 52.1 (O-CH_3), 24.2 (CH_3) ppm.

3cb. Methyl 5-(4,4,6-trimethyl-1,3,2-dioxaborinan-2-yl)furan-2-carboxylate

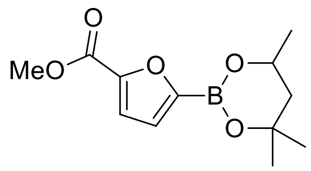

The compound (CAS 1451144-93-6) was prepared according to the general procedure using methyl 5-bromofuran-2-carboxylate (4.1 mg, 80 µmol, 1.0 equiv.), bis(hexyleneglycolato)diboron (53.0 mg, 200 µmol, 10.0 equiv.), dodecanenitrile (4.4 µL, 20 µmol, 1.0 equiv.) as internal standard and DIPEA (4.2 µL, 24 µmol, 1.2 equiv.) and G1 (10g/L). The reaction mixture was irradiated for 20 hours, obtaining 74% product yield according to GC-FID analysis (61% isolated yield as white powder).
Spectral data were consistent with literature.[5]

^{1}H NMR (400 MHz, $CDCl_3$) δ 7.11 (d, J = 3.4 Hz, 1H), 6.92 (d, J = 3.4 Hz, 1H), 4.31 (dtd, J = 12.4, 6.2, 2.9 Hz, 1H), 3.85 (s, 3H), 1.84 (dd, J = 14.0, 2.9 Hz, 1H), 1.63 – 1.52 (m, 1H), 1.33 (d, J = 3.8 Hz, 6H), 1.31 (d, J = 6.2 Hz, 3H) ppm.

---

[4] Neely, Jamie M.; Bezdek, Mate J.; Chirik, Paul J. *ACS Central Science* **2016**, *2*, (12), 935-942.
[5] Liskey, Carl W.; Hartwig, John F. *Synthesis* **2013**, *45*(13), 1837-1842.

^{13}**C NMR** (101 MHz, $CDCl_3$) δ 159.4 (C), 147.5 (C), 122.0 (C), 118.0 (CH), 116.4 (CH), 72.0 (C), 65.7 (CH), 51.9 (O-CH_3), 46.2 (CH_2), 31.0 (CH_3), 28.1 (CH_3), 23.0 (CH_3) ppm.

3cc. Methyl 5-(4,4,6,6-tetramethyl-1,3,2-dioxaborinan-2-yl)furan-2-carboxylate

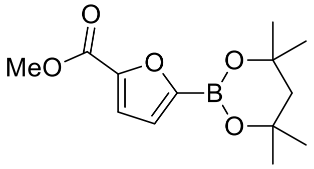

The compound was prepared according to the general procedure using methyl 5-bromofuran-2-carboxylate (4.1 mg, 80 µmol, 1.0 equiv.), 4,4,4',4',6,6,6',6'-octamethyl-2,2'-bi(1,3,2-dioxaborinane) (56.4 mg, 200 µmol, 10.0 equiv.), dodecanenitrile (4.4 µL, 20 µmol, 1.0 equiv.) as internal standard and DIPEA (4.2 µL, 24 µmol, 1.2 equiv.) and G1 (10 g/L). The reaction mixture was irradiated for 20 hours, obtaining 75% product yield according to GC-FID analysis (64% isolated yield as white powder).

^{1}**H NMR** (400 MHz, $CDCl_3$) δ 7.11 (d, J = 3.4 Hz, 1H), 6.93 (d, J = 3.4 Hz, 1H), 3.85 (s, 3H), 1.88 (s, 2H), 1.39 (s, 12H) ppm.
^{13}**C NMR** (101 MHz, $CDCl_3$) δ 159.5 (C), 147.4 (C), 122.1 (CH), 118.1 (CH), 71.8 (C), 51.9 (O-CH_3), 49.3 (CH_2), 31.7 (CH_3) ppm.
HRMS (EI): m/z $(M+H)^+$ = calcd. for $C_{13}H_{20}BO_5$: 267.1188, found: 267.1183.

3cd. Methyl 5-(5,5-dimethyl-1,3,2-dioxaborinan-2-yl)furan-2-carboxylate

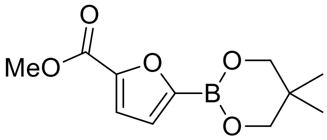

The compound was prepared according to the general procedure using methyl 5-bromofuran-2-carboxylate (4.1 mg, 80 µmol, 1.0 equiv.), bis(neopentylglycoltao)diboron (45.2 mg, 200 µmol, 10.0 equiv.), dodecanenitrile (4.4 µL, 20 µmol, 1.0 equiv.) as internal standard and DIPEA (4.2 µL, 24 µmol, 1.2 equiv.) and G1 (10g/L). The reaction mixture was irradiated for 20 hours, obtaining 72% product yield according to GC-FID analysis (60% isolated yield as white powder).

^{1}**H NMR** (400 MHz, $CDCl_3$) δ 7.17 (d, J = 3.5 Hz, 1H), 6.98 (d, J = 3.5 Hz, 1H), 3.89 (s, 3H), 3.76 (s, 4H), 1.02 (s, 6H) ppm.
^{13}**C NMR** (101 MHz, $CDCl_3$) δ 159.4 (C), 147.9 (C), 122.5 (CH), 118.2 (CH), 73.1 (C), 52.1 (O-CH_3), 32.2 (CH_2), 21.7 (CH_3) ppm.
HRMS (EI): m/z $(M+H)^+$ = calcd. for $C_{11}H_{15}BO_5$: 239.1085, found: 239.1078.

PYRROLES

4aa. 1-(1-Methyl-5-(4,4,5,5-tetramethyl-1,3,2-dioxaborolan-2-yl)-1H-pyrrol-2-yl)ethanone

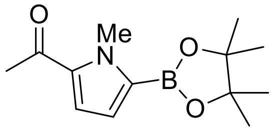

The compound was prepared according to the general procedure using 5-bromo-1-methyl-1H-pyrrole-2-carbaldehyde (4.0 mg, 20 µmol, 1.0 equiv.), bis(pinacolato)diboron (50.6 mg, 200 µmol, 10.0 equiv.), dodecanenitrile (4.4 µL, 20 µmol, 1.0 equiv.) as internal standard and DIPEA (4.2 µL, 24 µmol, 1.2 equiv.) and G1 (10g/L). The reaction mixture was irradiated for 22 hours, obtaining 91% product yield according to GC-FID analysis (72% isolated yield as yellow-pale powder).
^{1}**H NMR** (400 MHz, $CDCl_3$) δ 6.92 (d, J = 4.1 Hz, 1H), 6.70 (d, J = 4.0 Hz, 1H), 4.12 (s, 3H), 2.46 (s, 3H), 1.33 (s, 12H) ppm.
^{13}**C NMR** (101 MHz, $CDCl_3$) δ 189.6 (C), 168.7 (C), 120.3 (CH), 119.0 (CH), 84.0 (C), 36.7 (CH_3), 28.2 (CH_3), 24.9 (CH_3) ppm.
HRMS (EI): m/z $(M+H)^+$ = calcd. for $C_{13}H_{20}BNO_3$: 250.1609, found: 250.1604.

4ab. 1-(1-Methyl-5-(4,4,6-trimethyl-1,3,2-dioxaborinan-2-yl)-1H-pyrrol-2-yl)ethanone

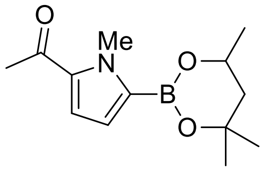

The compound was prepared according to the general procedure using 5-bromo-1-methyl-1H-pyrrole-2-carbaldehyde (4.0 mg, 20 µmol, 1.0 equiv.), bis(pinacolato)diboron (50.6 mg, 200 µmol, 10.0 equiv.), dodecanenitrile (4.4 µL, 20 µmol, 1.0 equiv.) as internal standard and DIPEA (4.2 µL, 24 µmol, 1.2 equiv.) and G1 (10g/L). The reaction mixture was irradiated for 20 hours, obtaining 83% product yield according to GC-FID analysis (61% isolated yield as yellow powder).

^{1}H NMR (400 MHz, $CDCl_3$) δ 6.90 (d, J = 4.0 Hz, 1H), 6.63 (d, J = 4.0 Hz, 1H), 4.33 (m, 1H), 4.12 (s, 3H), 2.43 (s, 3H), 1.86 (dd, J = 14.0, 2.9 Hz, 1H), 1.57 (t, J = 13.9 Hz, 1H), 1.35 (s, 6H), 1.32 (d, J = 6.1 Hz, 3H) ppm.
^{13}C NMR (101 MHz, $CDCl_3$) δ 189.4 (C), 165.7 (C), 119.1 (CH), 119.0 (CH), 71.8 (C), 65.5 (CH), 46.2 (CH_2), 36.4 (CH_3), 31.5 (CH_3), 28.3 (CH_3), 28.2 (CH_3), 23.3 (CH_3) ppm.
GC-MS (EI): m/z (relative intensity): 249.2 (30) [M+•], 234.1 (50), 134.1 (30), 70.1 (15), 61.0 (20), 56.1 (5), 43.1 (100).

4ac. 1-(1-Methyl-5-(4,4,6,6-tetramethyl-1,3,2-dioxaborinan-2-yl)-1H-pyrrol-2-yl)ethanone

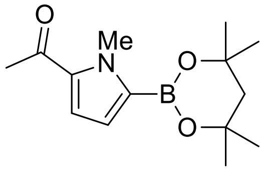

The compound was prepared according to the general procedure using 5-bromo-1-methyl-1H-pyrrole-2-carbaldehyde (4.0 mg, 20 µmol, 1.0 equiv.), bis(pinacolato)diboron (50.6 mg, 200 µmol, 10.0 equiv.), dodecanenitrile (4.4 µL, 20 µmol, 1.0 equiv.) as internal standard and DIPEA (4.2 µL, 24 µmol, 1.2 equiv.) and G1 (10g/L). The reaction mixture was irradiated for 20 hours, obtaining 85% product yield according to GC-FID analysis (63% isolated yield as yellow-pale powder).

^{1}H NMR (400 MHz, $CDCl_3$) δ 6.91 (d, J = 4.0 Hz, 1H), 6.64 (d, J = 4.0 Hz, 1H), 4.14 (s, 3H), 2.44 (s, 3H), 1.91 (s, 2H), 1.41 (s, 12H) ppm.
^{13}C NMR (101 MHz, $CDCl_3$) δ 189.4 (C), 134.8 (C), 119.1 (CH), 119.0 (CH), 71.5 (C), 49.0 (CH_2), 36.3 (CH_3), 31.9 (CH_3), 28.2 (CH_3) ppm.
GC-MS (EI): m/z (relative intensity): 263.1 (5) [M+•], 248.1 (5), 149.0 (5), 88.1 (10), 70.1 (20), 61.1 (30), 43.0 (100).

4ad. 1-(5-(5,5-dimethyl-1,3,2-dioxaborinan-2-yl)-1-methyl-1H-pyrrol-2-yl)ethanone

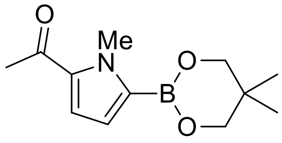

The compound was prepared according to the general procedure using 5-bromo-1-methyl-1H-pyrrole-2-carbaldehyde (4.0 mg, 20 µmol, 1.0 equiv.), bis(pinacolato)diboron (50.6 mg, 200 µmol, 10.0 equiv.), dodecanenitrile (4.4 µL, 20 µmol, 1.0 equiv.) as internal standard and DIPEA (4.2 µL, 24 µmol, 1.2 equiv.) and G1 (10g/L). The reaction mixture was irradiated for 20 hours, obtaining 65% product yield according to GC-FID analysis (44% isolated yield as yellow powder).

^{1}H NMR (400 MHz, $CDCl_3$) δ 6.91 (d, J = 3.7 Hz, 1H), 6.64 (d, J = 3.8 Hz, 1H), 4.11 (s, 3H), 3.74 (s, 4H), 2.44 (s, 3H), 1.01 (s, 6H) ppm.
^{13}C NMR (101 MHz, $CDCl_3$) δ 189.5 (C), 135.0 (C), 119.3 (CH), 119.1 (CH), 73.1 (C), 36.4 (CH_3), 32.0 (CH_2), 28.2 (CH_3), 21.7 (CH_3) ppm.
GC-MS (EI): m/z (relative intensity): 235.1 (15) [M+•], 220.1 (40), 192.0 (5), 148.9 (5), 134.0 (10), 105.9 (5), 88.0 (10), 70.0 (20), 61.0 (20), 52.9 (5), 43.1 (100).

4ba. 1-Methyl-5-(4,4,5,5-tetramethyl-1,3,2-dioxaborolan-2-yl)-1H-pyrrole-2-carbaldehyde

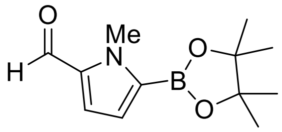

The compound was prepared according to the general procedure using 5-bromo-1-methyl-1H-pyrrole-2-carbaldehyde (3.8 mg, 20 µmol, 1.0 equiv.), bis(pinacolato)diboron (50.6 mg, 200 µmol, 10.0 equiv.), dodecanenitrile (4.4 µL, 20 µmol, 1.0 equiv.) as internal standard and DIPEA (4.2 µL, 24 µmol, 1.2 equiv.) and G1 (10g/L). The reaction mixture was irradiated for 20 hours, obtaining 72% product yield according to GC-FID analysis (56% isolated yield as reddish to black powder).

^{1}H NMR (400 MHz, $CDCl_3$) δ 9.56 (s, 1H), 6.81 (d, J = 4.0 Hz, 1H), 6.67 (d, J = 4.0 Hz, 1H), 4.05 (s, 3H), 1.25 (s, 12H) ppm.
^{13}C NMR (101 MHz, $CDCl_3$) δ 178.3 (C), 136.0 (C), 123.1 (CH), 112.6 (CH), 84.0 (C), 36.5 (CH_3), 24.0 (CH_3) ppm.
HRMS (EI): m/z $(M+H)^+$ = calcd. for $C_{12}H_{18}BNO_3$: 236.0725, found: 236.0713.

4bb. 1-Methyl-5-(4,4,6-trimethyl-1,3,2-dioxaborinan-2-yl)-1H-pyrrole-2-carbaldehyde

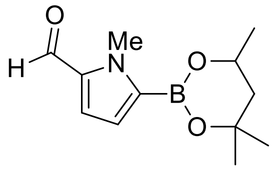

The compound was prepared according to the general procedure using 5-bromo-1-methyl-1H-pyrrole-2-carbaldehyde (3.8 mg, 80 µmol, 1.0 equiv.), bis(hexyleneglycolato)diboron (53.0 mg, 200 µmol, 10.0 equiv.), dodecanenitrile (4.4 µL, 20 µmol, 1.0 equiv.) as internal standard and DIPEA (4.2 µL, 24 µmol, 1.2 equiv.) and G1 (10g/L). The reaction mixture was irradiated for 14 hours, obtaining 81% product yield according to GC-FID analysis (65% isolated yield as reddish to black powder).

^{1}H NMR (400 MHz, $CDCl_3$) δ 9.61 (s, 1H), 6.86 (d, J = 4.0 Hz, 1H), 6.69 (d, J = 4.0 Hz, 1H), 4.34 (ddd, J = 11.6, 6.1, 2.9 Hz, 1H), 4.13 (s, 3H), 1.88 (dd, J = 14.0, 2.9 Hz, 1H), 1.60 (t, J = 12.7 Hz, 1H), 1.37 (s, 6H), 1.33 (d, J = 6.2 Hz, 3H) ppm.
^{13}C NMR (101 MHz, $CDCl_3$) δ 180.3 (C), 135.9 (C), 123.5 (CH), 120.0 (CH), 109.7 (CH), 71.9 (C), 65.6 (CH), 46.1 (CH_2), 35.5 (CH_3), 31.3 (CH_3), 28.3 (CH_3), 23.2 (CH_3) ppm.
HRMS (EI): m/z $(M+H)^+$ = calcd. for $C_{12}H_{18}BNO_3$: 236.1453, found: 236.1445.

4bc. 1-Methyl-5-(4,4,6,6-tetramethyl-1,3,2-dioxaborinan-2-yl)-1H-pyrrole-2-carbaldehyde

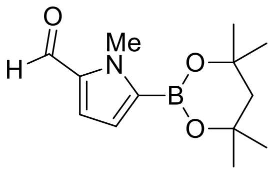

The compound was prepared according to the general procedure using 5-bromo-1-methyl-1H-pyrrole-2-carbaldehyde (3.8 mg, 80 µmol, 1.0 equiv.), 4,4,4',4',6,6,6',6'-octamethyl-2,2'-bi(1,3,2-dioxaborinane) (56.4 mg, 200 µmol, 10.0 equiv.), dodecanenitrile (4.4 µL, 20 µmol, 1.0 equiv.) as internal standard and DIPEA (4.2 µL, 24 µmol, 1.2 equiv.) and G1 (10g/L). The reaction mixture was irradiated for 20 hours, obtaining 74% product yield according to GC-FID analysis (59% isolated yield as reddish to black powder).

^{1}H NMR (400 MHz, $CDCl_3$) δ 9.59 (s, 1H), 6.85 (d, J = 4.0 Hz, 1H), 6.68 (d, J = 4.0 Hz, 1H), 4.13 (s, 3H), 1.91 (s, 2H), 1.40 (s, 12H) ppm.
^{13}C NMR (101 MHz, $CDCl_3$) δ 179.7 (C), 135.8 (C), 123.4 (CH), 109.6 (CH), 71.6 (C), 49.0 (CH_2), 35.5 (CH_3), 31.8 (CH_3) ppm.
HRMS (EI): m/z $(M+H)^+$ = calcd. for $C_{13}H_{20}BNO_3$: 250.1609, found: 250.1604.

4bd. 5-(5,5-dimethyl-1,3,2-dioxaborinan-2-yl)-1-methyl-1H-pyrrole-2-carbaldehyde

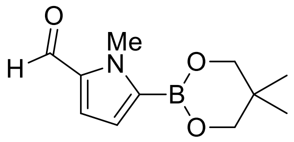

The compound was prepared according to the general procedure using 5-bromo-1-methyl-1H-pyrrole-2-carbaldehyde (3.8 mg, 80 µmol, 1.0 equiv.), bis(neopentylglycoltao)diboron (45.2 mg, 200 µmol, 10.0 equiv.), dodecanenitrile (4.4 µL, 20 µmol, 1.0 equiv.) as internal standard and DIPEA (4.2 µL, 24 µmol, 1.2 equiv.) and G1 (10 g/L). The reaction mixture was irradiated for 14 hours, obtaining 64% product yield according to GC-FID analysis (51% isolated yield reddish to black powder).

^{1}H NMR (400 MHz, $CDCl_3$) 9.59 (s, 1H), 6.85 (d, J = 4.0 Hz, 1H), 6.68 (d, J = 4.0 Hz, 1H), 4.11 (s, 3H), 3.73 (s, 4H), 1.00 (s, 6H) ppm.
^{13}C NMR (101 MHz, $CDCl_3$) δ 179.7 (C), 132.2 (C), 124.3 (CH), 109.6 (CH), 73.0 (C), 36.4 (CH_3), 32.1 (CH_2), 21.7 (CH_3) ppm.
HRMS (EI): m/z $(M+H)^+$ = calcd. for $C_{11}H_{16}BNO_3$: 223.0944, found: 223.0956.

4ca. Methyl 1-methyl-5-(4,4,5,5-tetramethyl-1,3,2-dioxaborolan-2-yl)-1H-pyrrole-2-carboxylate

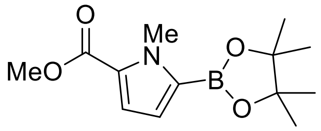

The compound (CAS 2377607-43-5) was prepared according to the general procedure using methyl 5-bromo-1-methyl-1H-pyrrole-2-carboxylate (4.4 mg, 20 µmol, 1.0 equiv.), bis(pinacolato)diboron (50.6 mg, 200 µmol, 10.0 equiv.), dodecanenitrile (4.4 µL, 20 µmol, 1.0 equiv.) as internal standard and DIPEA (4.2 µL, 24 µmol, 1.2 equiv.) and G1 (10g/L). The reaction mixture was irradiated for 18 hours, obtaining 69% product yield according to GC-FID analysis (55% isolated yield as yellow-pale oil).
^{1}H NMR (400 MHz, $CDCl_3$) δ 6.91 (d, J = 4.0 Hz, 1H), 6.70 (d, J = 4.0 Hz, 1H), 4.13 (s, 3H), 3.81 (s, 3H), 1.32 (s, 12H) ppm.
^{13}C NMR (101 MHz, $CDCl_3$) δ 161.91 (C), 127.49 (C), 120.41 (CH), 117.29 (CH), 83.85 (C), 51.27 (O-CH_3), 35.92 (CH_3), 24.92 (CH_3) ppm.
HRMS (EI): m/z $(M+H)^+$ = calcd. for $C_{13}H_{21}BNO_4$: 266.1559, found: 266.1564.

4cb. Methyl 1-methyl-5-(4,4,6-trimethyl-1,3,2-dioxaborinan-2-yl)-1H-pyrrole-2-carboxylate

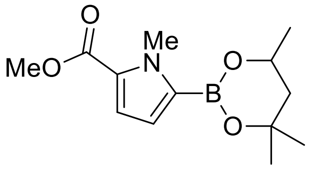

The compound was prepared according to the general procedure using methyl 5-bromo-1-methyl-1H-pyrrole-2-carboxylate (4.4 mg, 80 µmol, 1.0 equiv.), bis(hexyleneglycolato)diboron (53.0 mg, 200 µmol, 10.0 equiv.), dodecanenitrile (4.4 µL, 20 µmol, 1.0 equiv.) as internal standard and DIPEA (4.2 µL, 24 µmol, 1.2 equiv.) and G1 (10g/L). The reaction mixture was irradiated for 19 hours, obtaining 76% product yield according to GC-FID analysis (62% isolated yield as yellow-pale oil).

^{1}H NMR (400 MHz, $CDCl_3$) δ 6.89 (d, J = 3.9 Hz, 1H), 6.64 (d, J = 3.9 Hz, 1H), 4.32 (ddd, J = 11.7, 6.0, 3.0 Hz, 1H), 4.12 (s, 3H), 3.80 (s, 3H), 1.85 (dd, J = 13.9, 2.9 Hz, 1H), 1.63 – 1.52 (m, 1H), 1.36 (s, 6H), 1.32 (d, J = 6.2 Hz, 3H) ppm.
^{13}C NMR (101 MHz, $CDCl_3$) δ 162.1 (C), 126.7 (C), 119.1 (CH), 117.1 (CH), 71.6 (C), 65.4 (CH), 51.1 (O-CH_3), 46.1 (CH_2), 35.5 (CH_3), 31.4 (CH_3), 28.3 (CH_3), 23.3 (CH_3) ppm.
GC-MS (EI): m/z (relative intensity): 265.1 (60) [M+•], 233.9 (30), 206.8 (20), 166.0 (50), 152.0 (20), 134.1 (40), 108.0 (30), 72.1 (20), 43.1 (100).

4cc. Methyl 1-methyl-5-(4,4,6,6-tetramethyl-1,3,2-dioxaborinan-2-yl)-1H-pyrrole-2-carboxylate

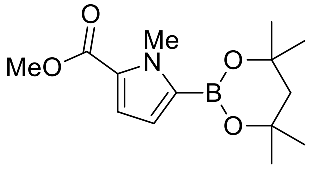

The compound was prepared according to the general procedure using methyl 5-bromo-1-methyl-1H-pyrrole-2-carboxylate (4.4 mg, 80 µmol, 1.0 equiv.), 4,4,4',4',6,6,6',6'-octamethyl-2,2'-bi(1,3,2-dioxaborinane) (56.4 mg, 200 µmol, 10.0 equiv.), dodecanenitrile (4.4 µL, 20 µmol, 1.0 equiv.) as internal standard and DIPEA (4.2 µL, 24 µmol, 1.2 equiv.) and G1 (10g/L). The reaction mixture was irradiated for 15 hours, obtaining 77% product yield according to GC-FID analysis (63% isolated yield as yellow-pale oil).

^{1}H NMR (400 MHz, $CDCl_3$) δ 6.89 (d, J = 3.9 Hz, 1H), 6.65 (d, J = 3.9 Hz, 1H), 4.14 (s, 3H), 3.80 (s, 3H), 1.91 (s, 2H), 1.41 (s, 12H) ppm.
^{13}C NMR (101 MHz, $CDCl_3$) δ 162.2 (C), 120.0 (C), 119.1 (CH), 117.1 (CH), 71.4 (C), 51.1 (O-CH_3), 49.1 (CH_2), 35.6 (CH_3), 31.9 (CH_3) ppm.
HRMS (EI): m/z $(M+H)^+$ = calcd. for $C_{14}H_{22}BNO_4$: 280.1715, found: 280.1710.

4cd. Methyl 5-(5,5-dimethyl-1,3,2-dioxaborinan-2-yl)-1-methyl-1H-pyrrole-2-carboxylate

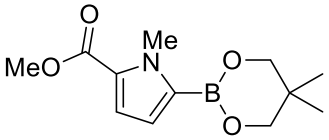

The compound was prepared according to the general procedure using methyl 5-bromo-1-methyl-1H-pyrrole-2-carboxylate (4.4 mg, 80 µmol, 1.0 equiv.), bis(neopentylglycoltao)diboron (45.2 mg, 200 µmol, 10.0 equiv.), dodecanenitrile (4.4 µL, 20 µmol, 1.0 equiv.) as internal standard and DIPEA (4.2 µL, 24 µmol, 1.2 equiv.) and G1 (10g/L). The reaction mixture was irradiated for 19 hours, obtaining 44% product yield according to GC-FID analysis (36% isolated yield as yellow-pale oil).

^{1}H NMR (400 MHz, $CDCl_3$) δ 6.90 (d, J = 3.9 Hz, 1H), 6.65 (d, J = 3.9 Hz, 1H), 4.12 (s, 3H), 3.80 (s, 3H), 3.74 (s, 4H), 1.02 (s, 6H) ppm.
^{13}C NMR (101 MHz, $CDCl_3$) δ 162.1 (C), 119.4 (C), 117.2 (CH), 111.8 (CH), 73.1 (CH_2), 51.2 (O-CH_3), 35.8 (CH_3), 32.1 (CH_3), 21.6 (CH_3) ppm.
HRMS (EI): m/z $(M+H)^+$ = calcd. for $C_{12}H_{19}BNO_4$: 252.1402, found: 252.1406.

<u>**SELENOPHENES**</u>

5aa. 1-(5-(4,4,5,5-tetramethyl-1,3,2-dioxaborolan-2-yl)selenophen-2-yl)ethenone

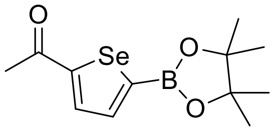

The compound was prepared according to the general procedure using 1-(5-bromoselenophen-2-yl)ethanone (5.0 mg, 20 µmol, 1.0 equiv.), bis(pinacolato)diboron (50.6 mg, 200 µmol, 10.0 equiv.), dodecanenitrile (4.4 µL, 20 µmol, 1.0 equiv.) as internal standard and DIPEA (4.2 µL, 24 µmol, 1.2 equiv.) and G1 (10g/L). The reaction mixture was irradiated for 16 hours, obtaining 84% product yield according to GC-FID analysis (70% isolated yield as yellow-brown powder).

^{1}H NMR (400 MHz, $CDCl_3$) δ 7.94 (d, J = 3.9 Hz, 1H), 7.90 (d, J = 3.9 Hz, 1H), 2.58 (s, 3H), 1.34 (s, 12H) ppm.
^{13}C NMR (101 MHz, $CDCl_3$) δ 192.2 (C), 156.8 (C), 140.0 (CH), 135.6 (CH), 84.8 (C), 26.9 (CH_3), 24.9 (CH_3) ppm.
HRMS (EI): m/z $(M+H)^+$ = calcd. for $C_{12}H_{17}BO_3Se$: 301.0509, found: 301.0515.

5ab. 1-(5-(4,4,6-trimethyl-1,3,2-dioxaborinan-2-yl)selenophen-2-yl)ethanone

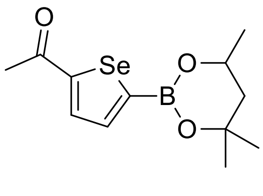

The compound was prepared according to the general procedure using 1-(5-bromoselenophen-2-yl)ethanone (5.0 mg, 20 µmol, 1.0 equiv.), bis(pinacolato)diboron (50.6 mg, 200 µmol, 10.0 equiv.), dodecanenitrile (4.4 µL, 20 µmol, 1.0 equiv.) as internal standard and DIPEA (4.2 µL, 24 µmol, 1.2 equiv.) and G1 (10g/L). The reaction mixture was irradiated for 16 hours, obtaining 86% product yield according to GC-FID analysis (73% isolated yield as yellow oil).

^{1}H NMR (400 MHz, $CDCl_3$) δ 7.91 (d, J = 3.8 Hz, 1H), 7.81 (d, J = 3.9 Hz, 1H), 4.34 (dtt, J = 12.2, 6.1, 3.1 Hz, 1H), 2.56 (s, 3H), 1.87 (dd, J = 14.0, 2.9 Hz, 1H), 1.67 – 1.56 (m, 1H), 1.36 (s, 6H), 1.33 (d, J = 6.2 Hz, 3H) ppm.
^{13}C NMR (101 MHz, $CDCl_3$) δ 192.3 (C), 155.1 (C), 137.9 (CH), 135.7 (CH), 72.1 (C), 65.8 (CH), 46.1 (CH_2), 31.2 (CH_3), 28.2 (CH_3), 26.8 (CH_3), 23.2 (CH_3) ppm.
HRMS (EI): m/z $(M+H)^+$ = calcd. for $C_{12}H_{17}BO_3Se$: 301.0509, found: 301.0502.

5ac. 1-(5-(4,4,6,6-tetramethyl-1,3,2-dioxaborinan-2-yl)selenophen-2-yl)ethanone

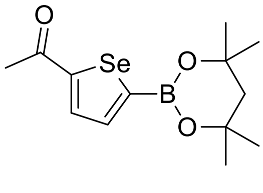

The compound was prepared according to the general procedure using 1-(5-bromoselenophen-2-yl)ethanone (5.0 mg, 20 µmol, 1.0 equiv.), bis(pinacolato)diboron (50.6 mg, 200 µmol, 10.0 equiv.), dodecanenitrile (4.4 µL, 20 µmol, 1.0 equiv.) as internal standard and DIPEA (4.2 µL, 24 µmol, 1.2 equiv.) and G1 (10g/L). The reaction mixture was irradiated for 16 hours, obtaining 88% product yield according to GC-FID analysis (69% isolated yield as yellow oil).

^{1}H NMR (400 MHz, $CDCl_3$) δ 7.92 (d, J = 3.9 Hz, 1H), 7.83 (d, J = 3.9 Hz, 1H), 2.57 (s, 3H), 1.92 (s, 2H), 1.42 (s, 12H) ppm.
^{13}C NMR (101 MHz, $CDCl_3$) δ 192.3 (C), 155.0 (C), 137.8 (CH), 135.7 (CH), 71.9 (C), 49.1 (CH_2), 31.8 (CH_3), 26.8 (CH_3) ppm.
HRMS (EI): m/z $(M+H)^+$ = calcd. for $C_{13}H_{19}BO_3Se$: 315.0665, found: 315.0658.

5ad. 1-(5-(5,5-dimethyl-1,3,2-dioxaborinan-2-yl)selenophen-2-yl)ethanone

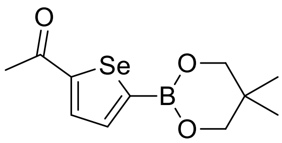

The compound was prepared according to the general procedure using 1-(5-bromoselenophen-2-yl)ethanone (5.0 mg, 20 µmol, 1.0 equiv.), bis(pinacolato)diboron (50.6 mg, 200 µmol, 10.0 equiv.), dodecanenitrile (4.4 µL, 20 µmol, 1.0 equiv.) as internal standard and DIPEA (4.2 µL, 24 µmol, 1.2 equiv.) and G1 (10g/L). The reaction mixture was irradiated for 22 hours, obtaining 68% product yield according to GC-FID analysis (51% isolated yield as yellow oil).

^{1}H NMR (400 MHz, $CDCl_3$) δ 7.93 (d, J = 3.9 Hz, 1H), 7.84 (d, J = 3.9 Hz, 1H), 3.76 (s, 4H), 2.58 (s, 3H), 1.03 (s, 6H) ppm.
^{13}C NMR (101 MHz, $CDCl_3$) δ 207.1 (C), 155.1 (C), 138.4 (CH), 135.8 (CH), 72.7 (C), 31.1 (CH_2), 26.8 (CH_3), 22.0 (CH_3) ppm.
HRMS (EI): m/z $(M+H)^+$ = calcd. for $C_{11}H_{15}BO_3Se$: 285.0060, found: 285.2277.

NMR Spectra

^{1}H 400MHz, ACN-d3

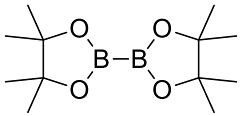

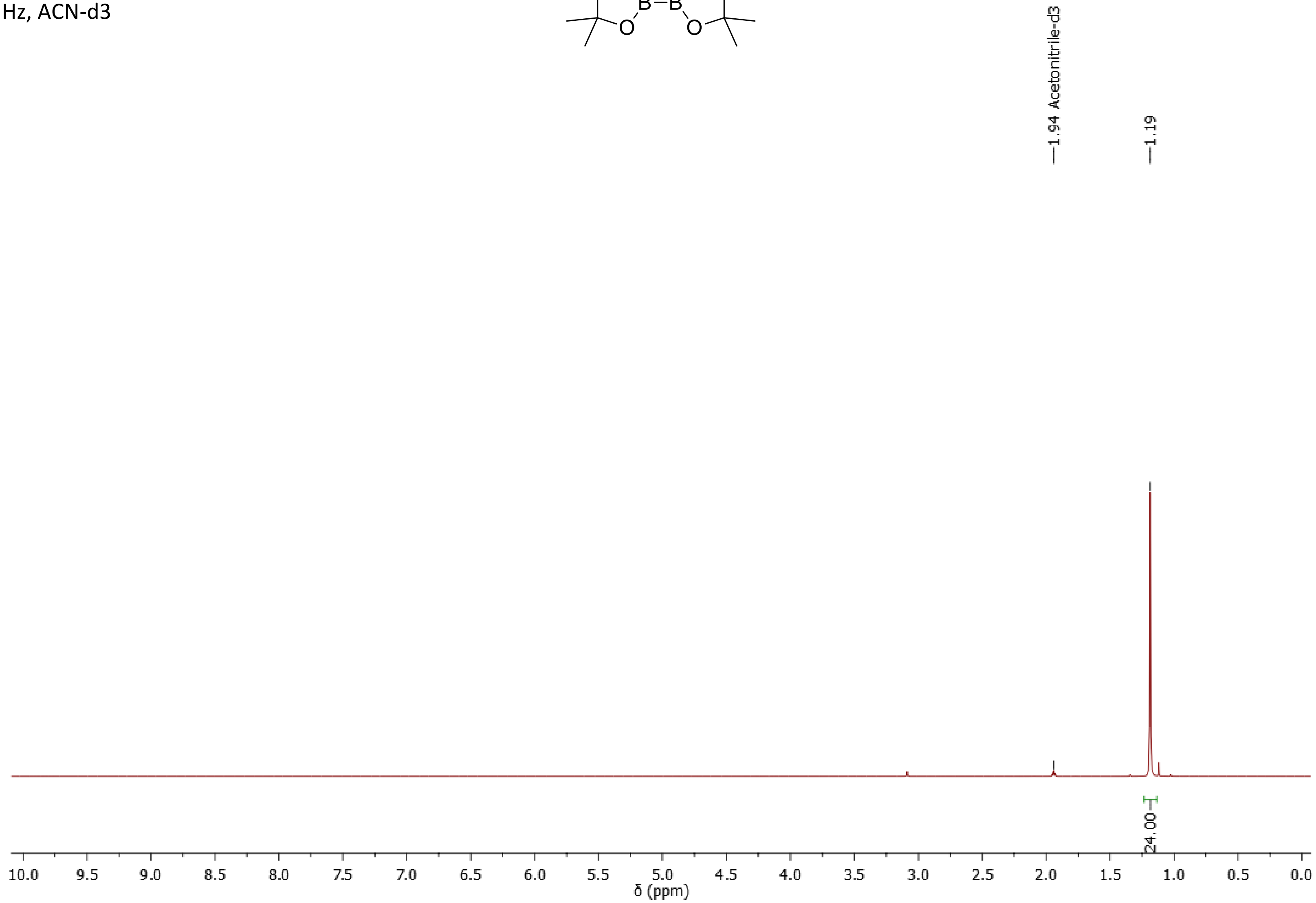

^{13}C 101MHz

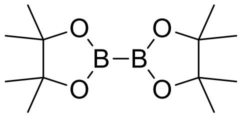
O
O
B
B
O
O

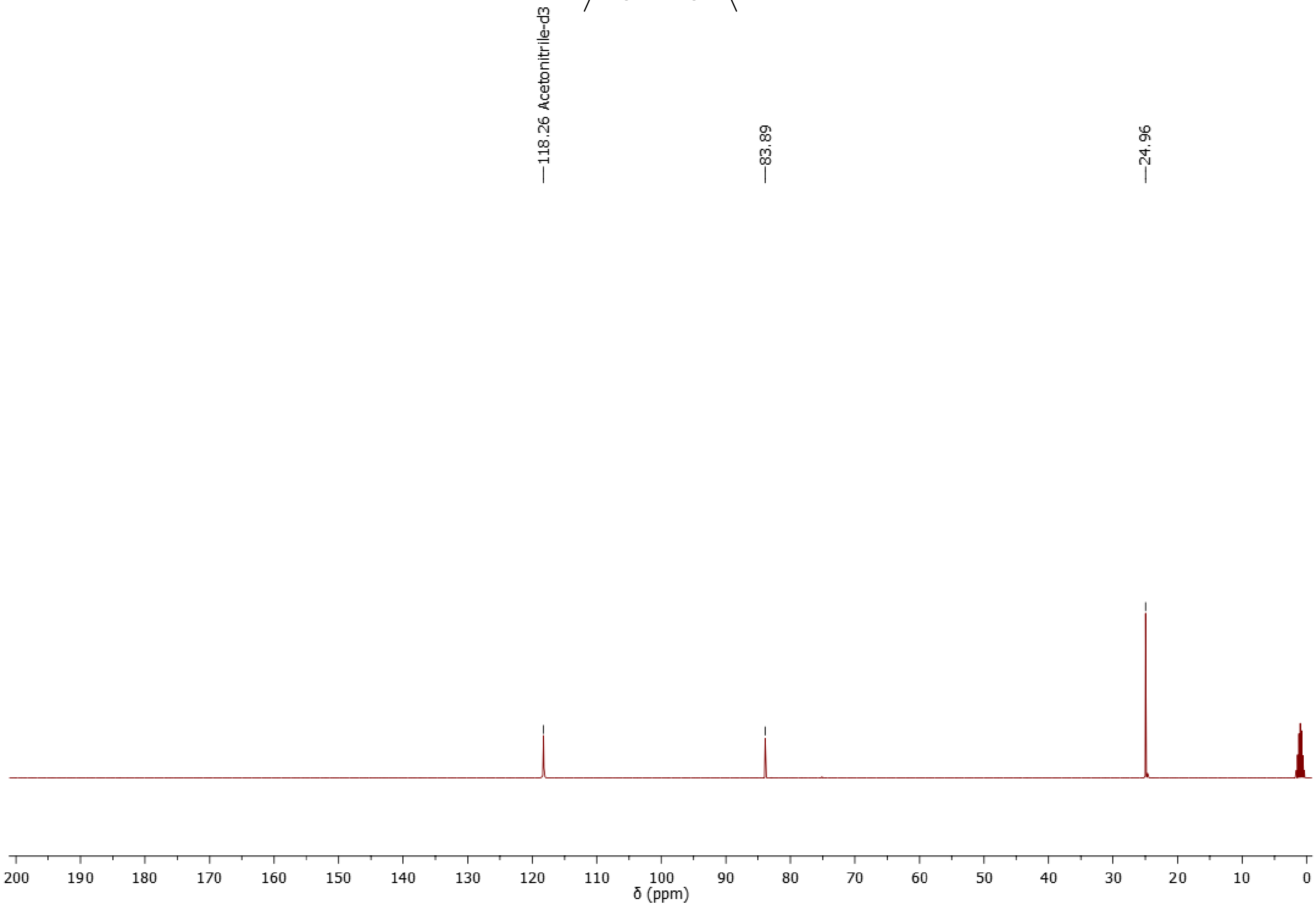
118.26 Acetonitrile-d3
83.89
24.96
200
190
180
170
160
150
140
130
120
110
100
90
80
70
60
50
40
30
20
10
0
δ (ppm)

^{1}H 400MHz, $CDCl_3$

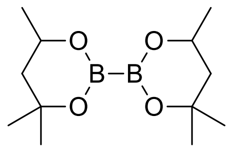

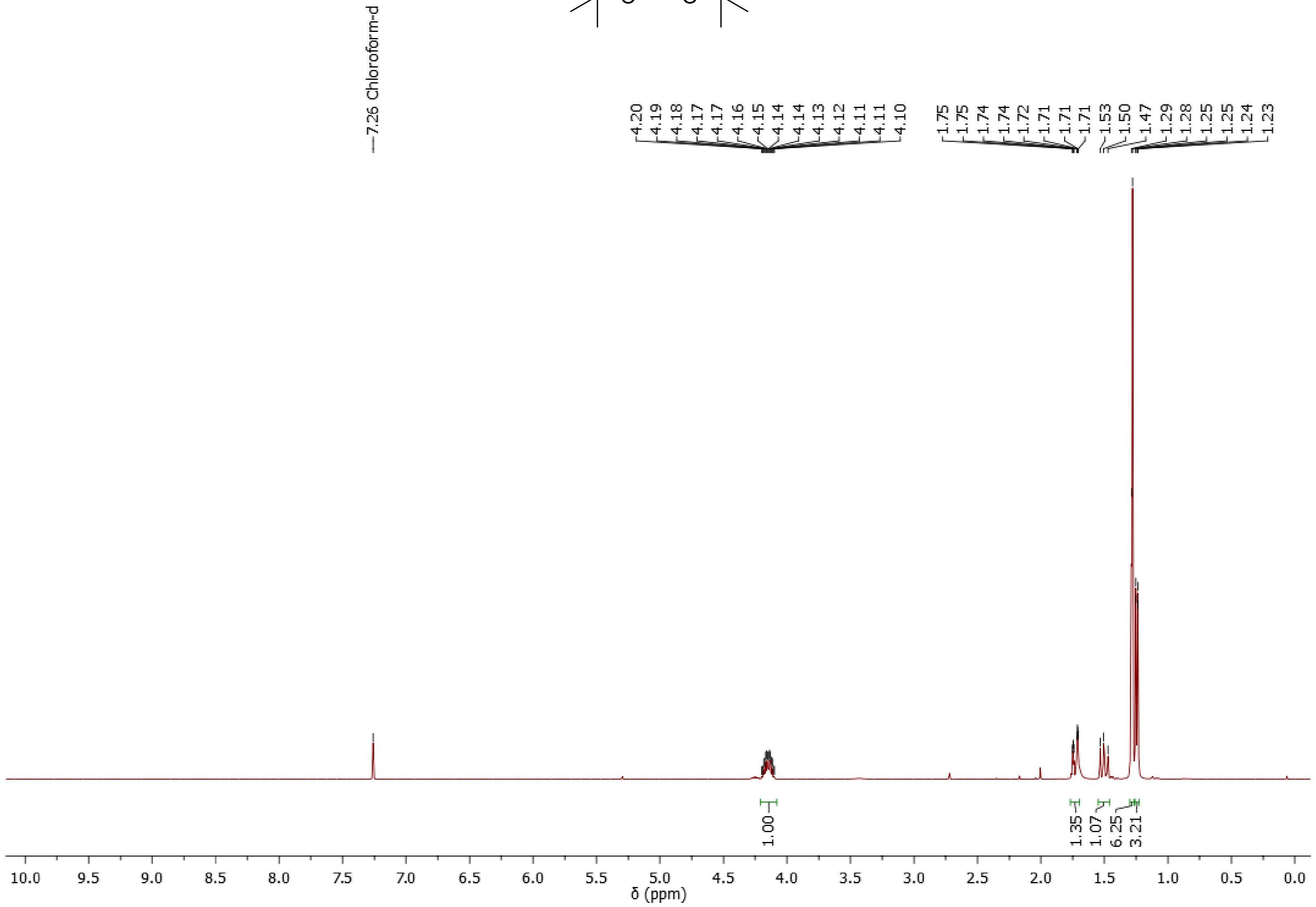

^{13}C 101MHz

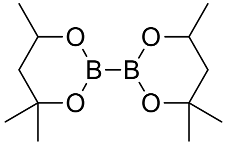

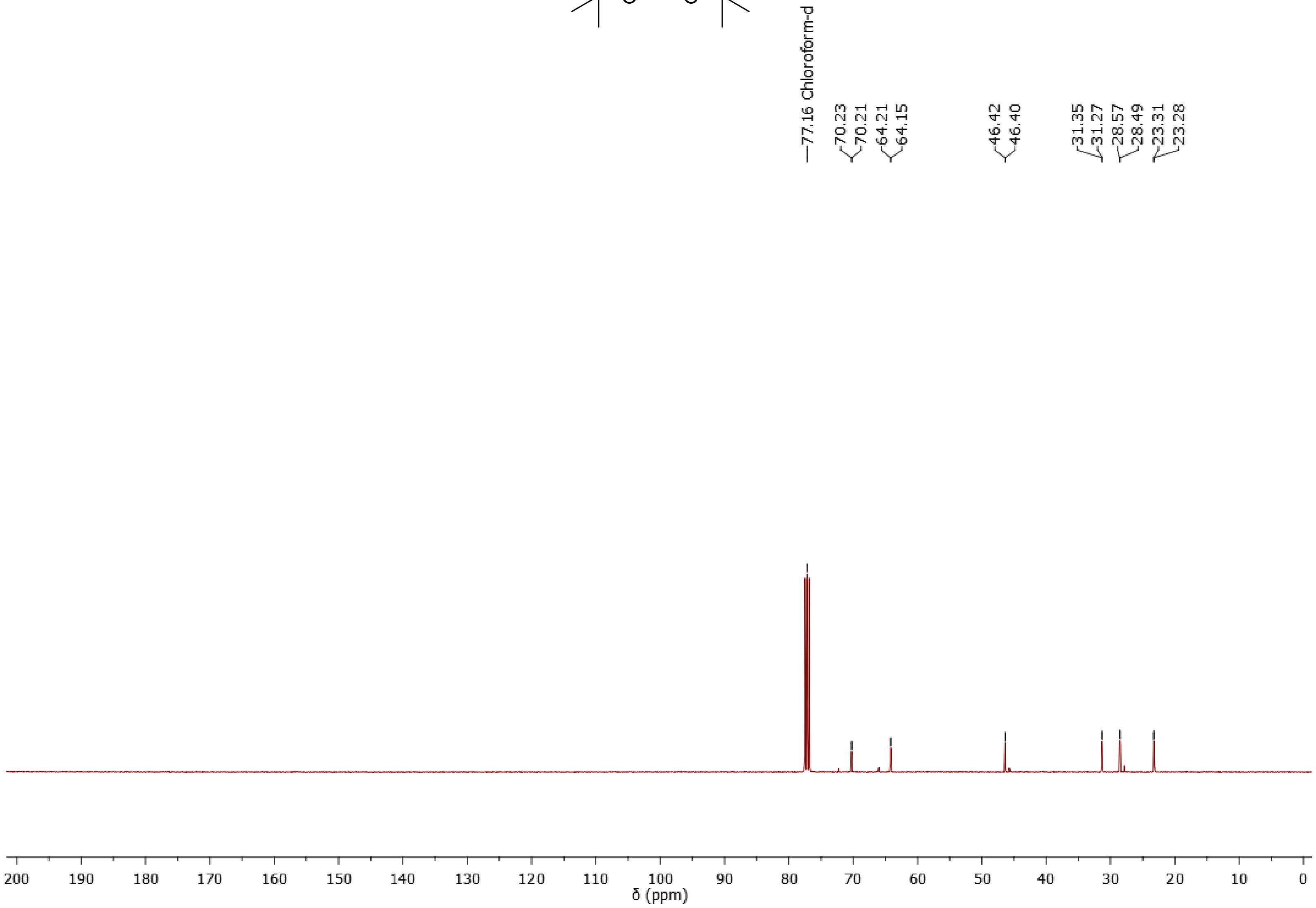
77.16 Chloroform-d
70.23
70.21
64.21
64.15
46.42
46.40
31.35
31.27
28.57
28.49
23.31
23.28
200 190 180 170 160 150 140 130 120 110 100 90 80 70 60 50 40 30 20 10 0
δ (ppm)

^{1}H 400MHz, $CDCl_3$

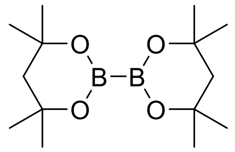

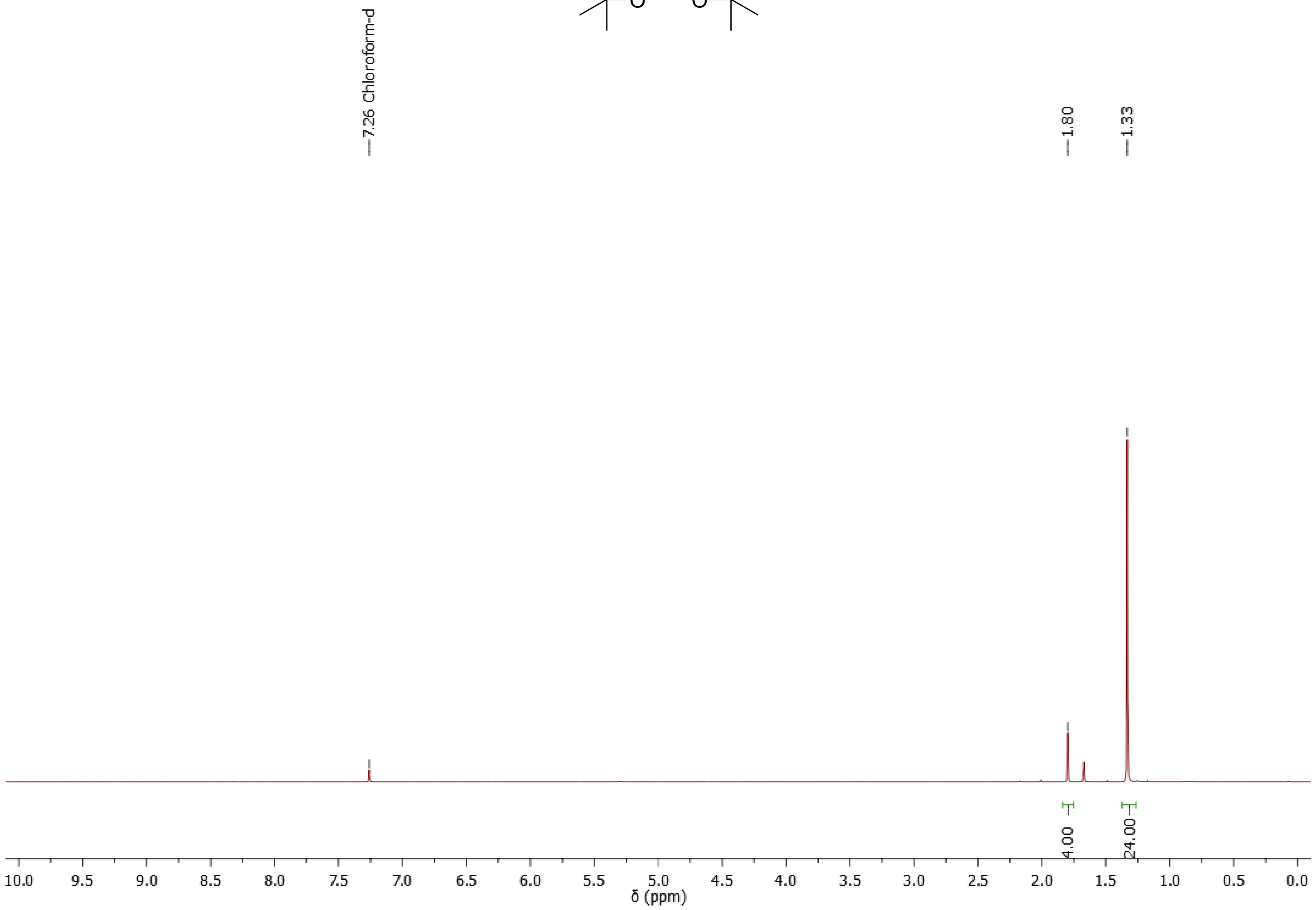

^{13}C 101MHz

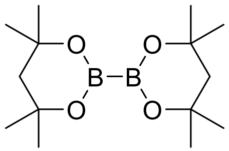

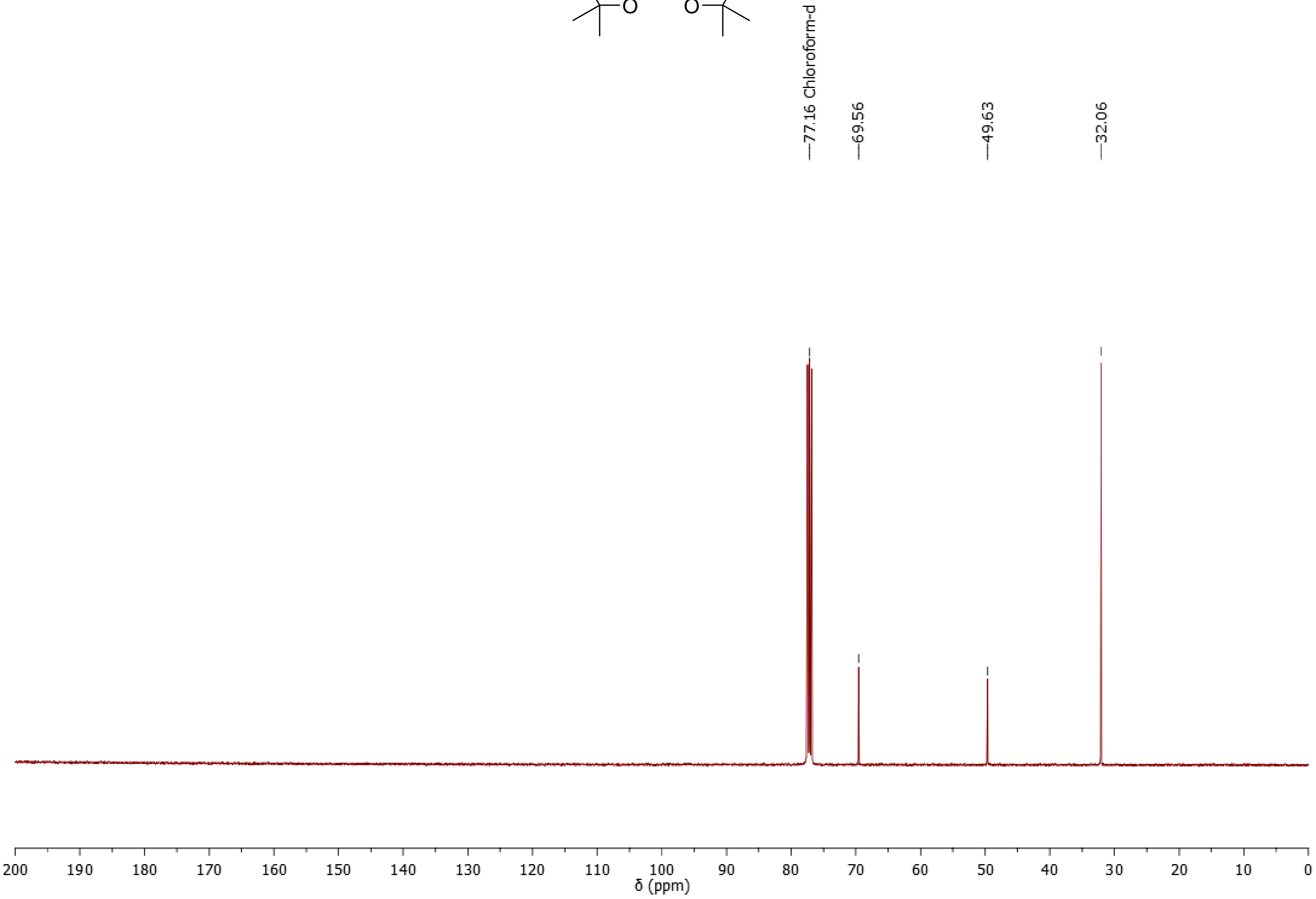
77.16 Chloroform-d
69.56
49.63
32.06
200
190
180
170
160
150
140
130
120
110
100
90
80
70
60
50
40
30
20
10
0
δ (ppm)

^{1}H 400MHz, $CDCl_3$

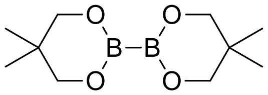

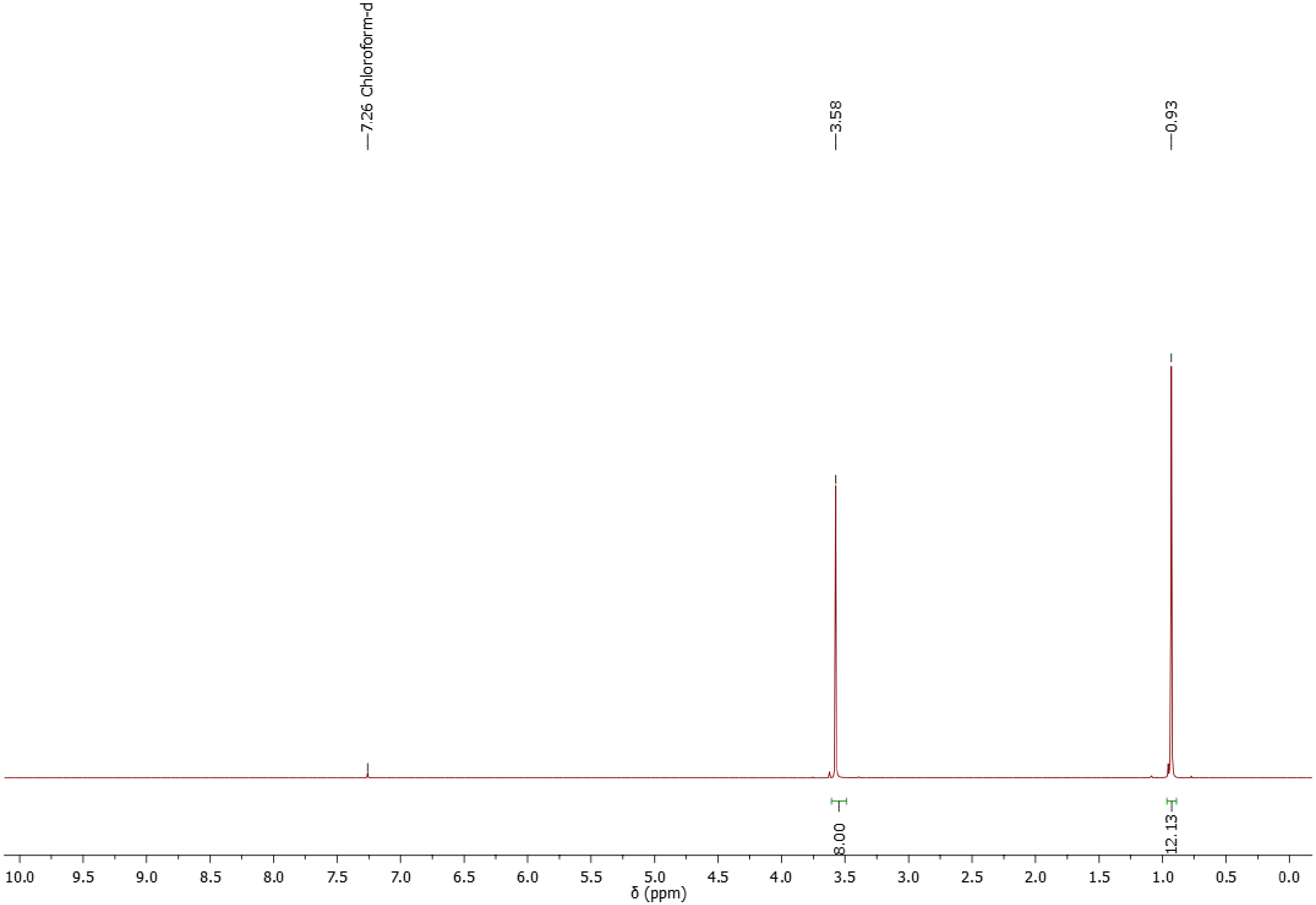

^{13}C 101MHz

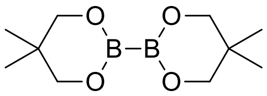
O
B
B
O
O
O

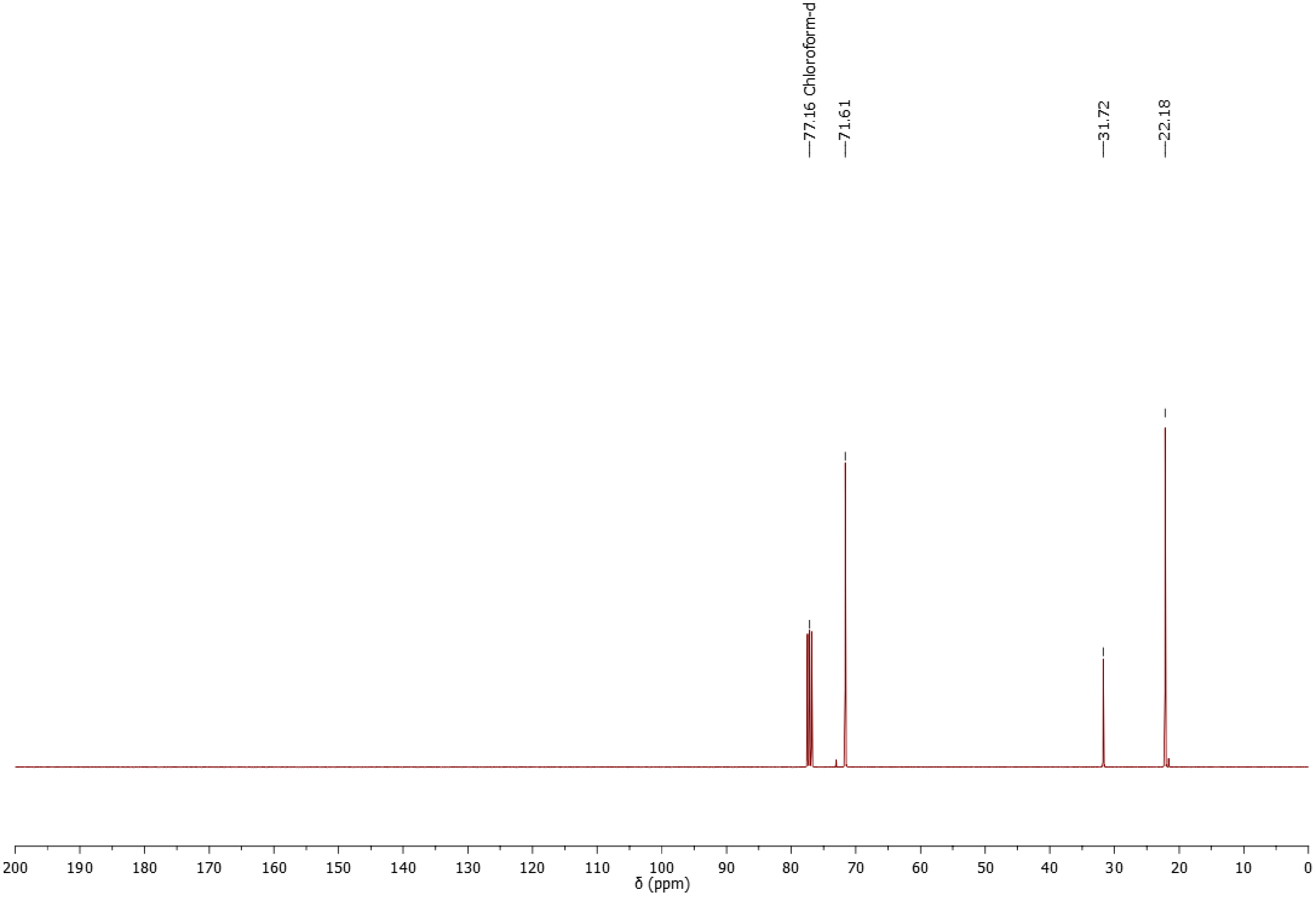
77.16 Chloroform-d
71.61
31.72
22.18
200
190
180
170
160
150
140
130
120
110
100
90
80
70
60
50
40
30
20
10
0
δ (ppm)

^{1}H 400MHz, $CDCl_3$

2aa

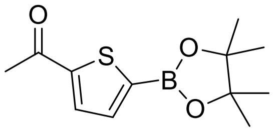

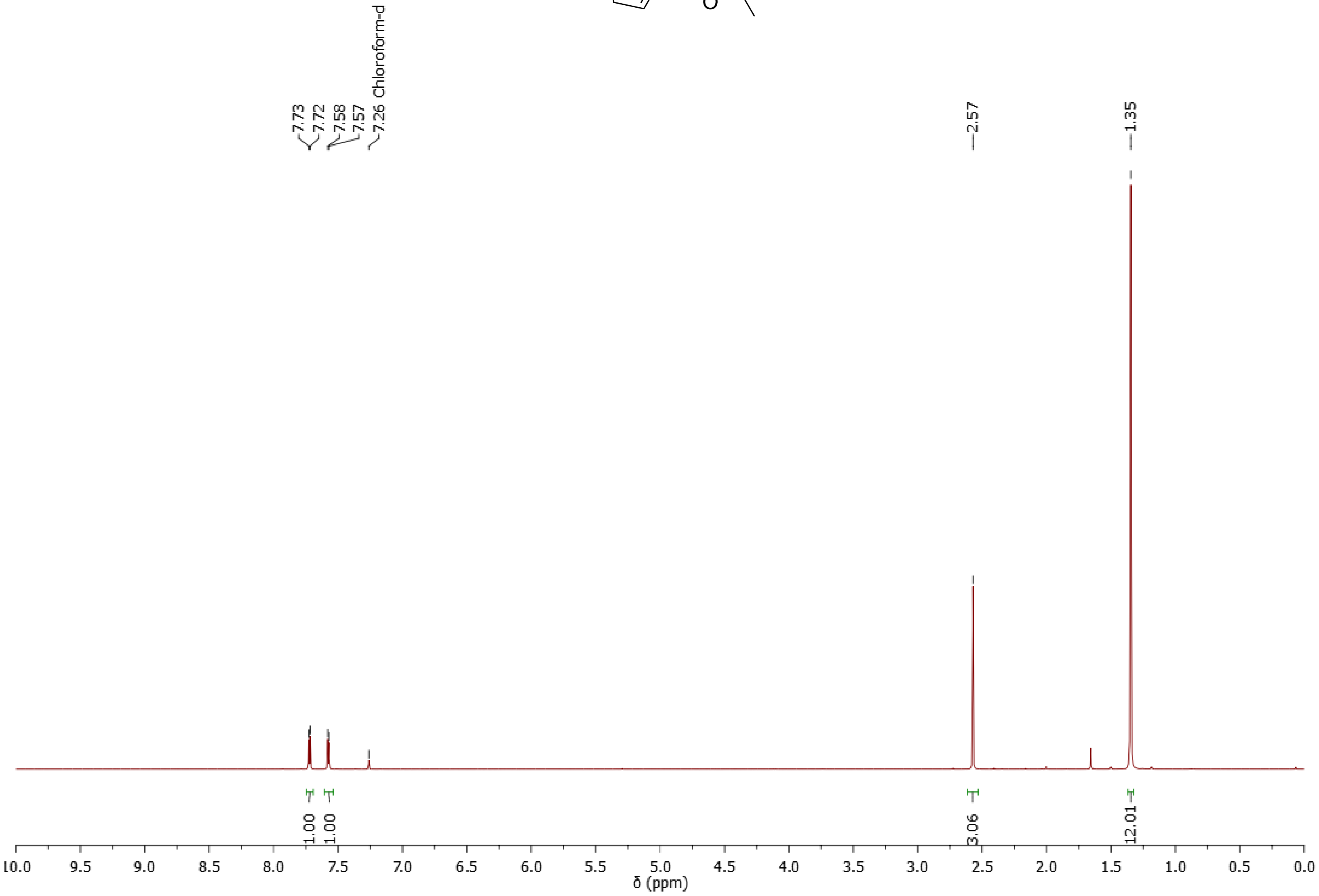

^{13}C 101MHz

2aa

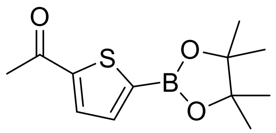

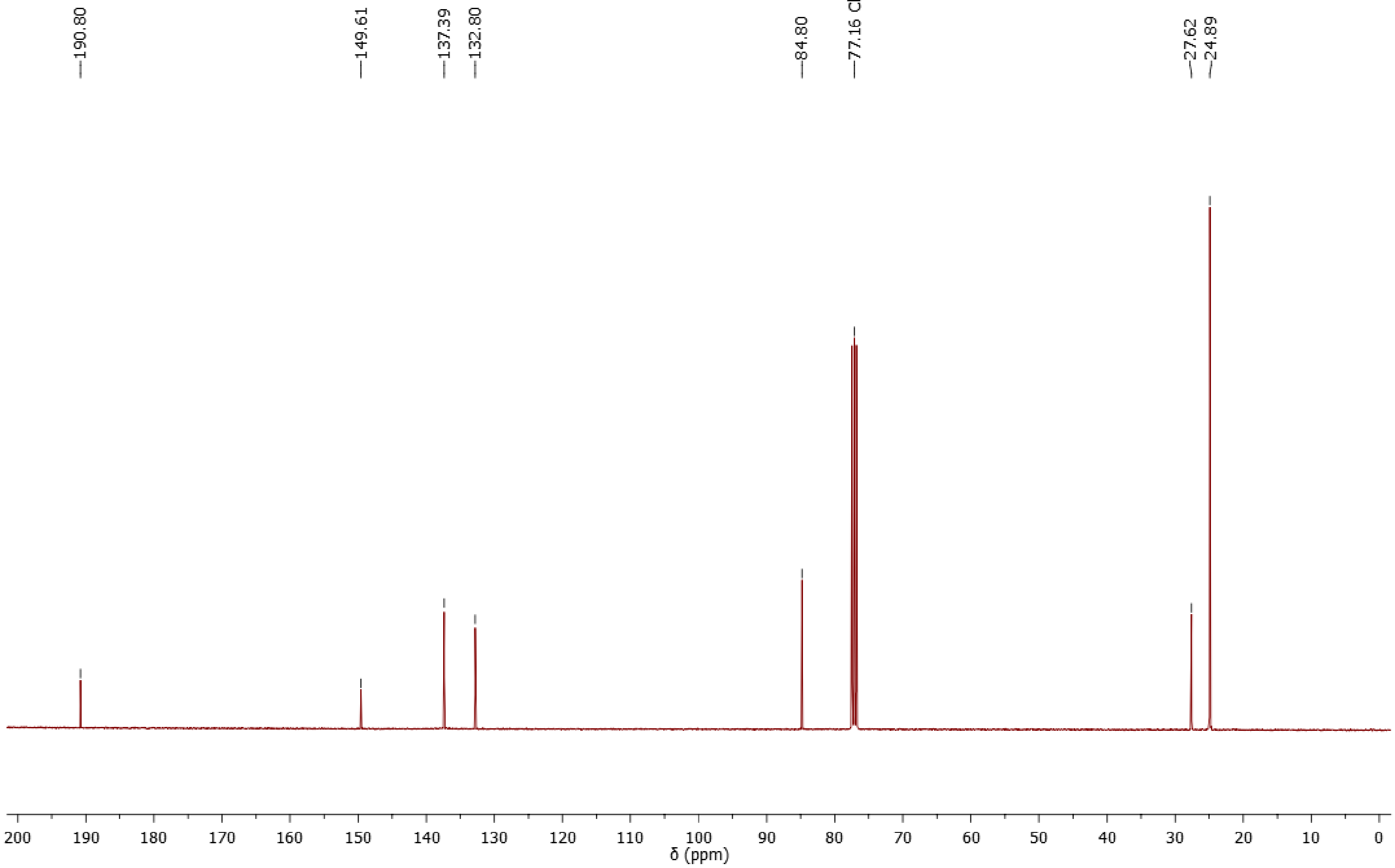

^{1}H 400MHz, $CDCl_3$

2ab

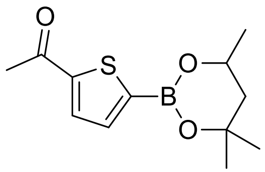

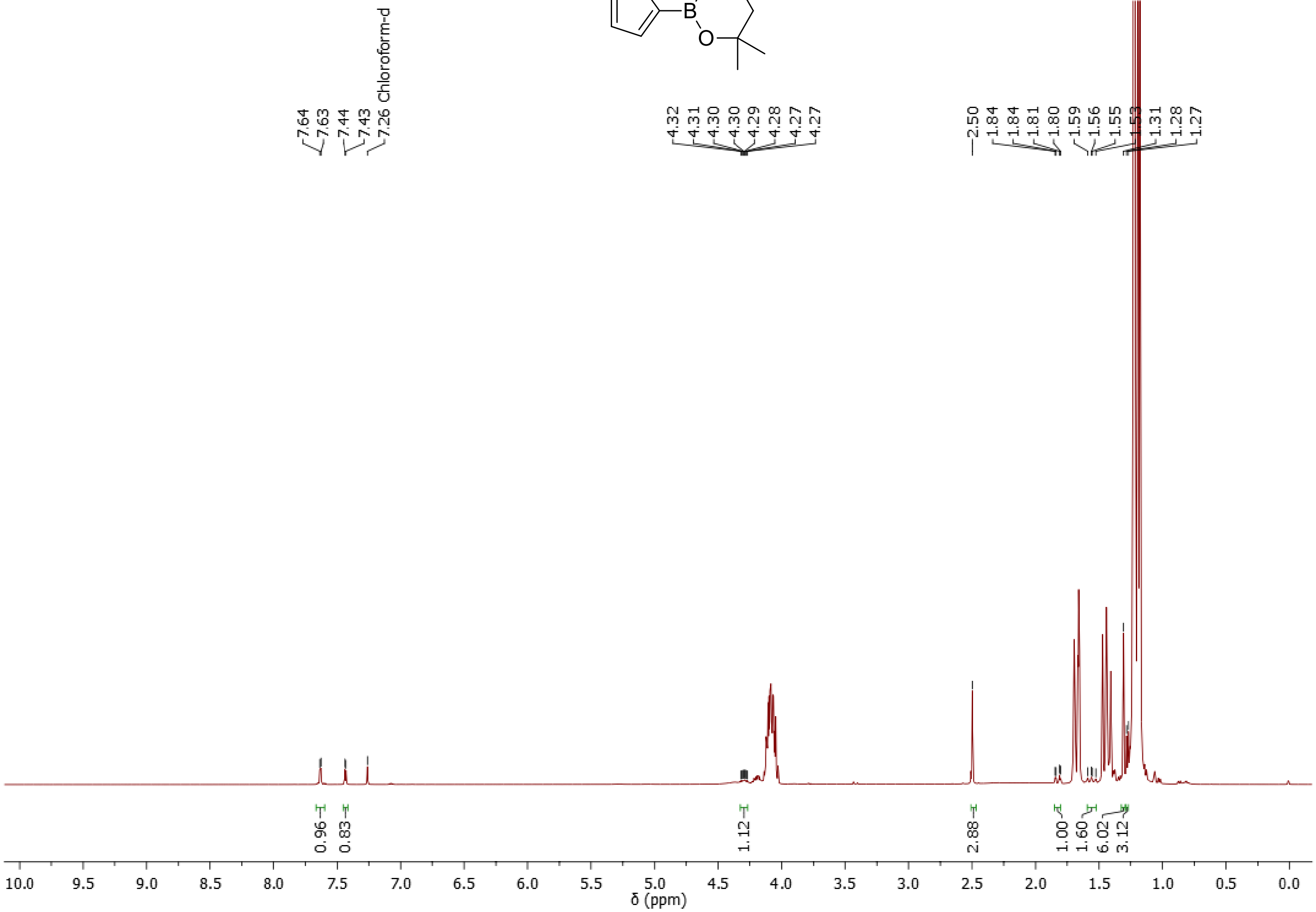

^{13}C 101MHz

2ab

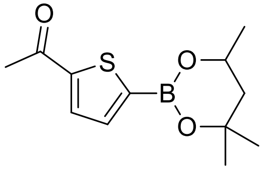

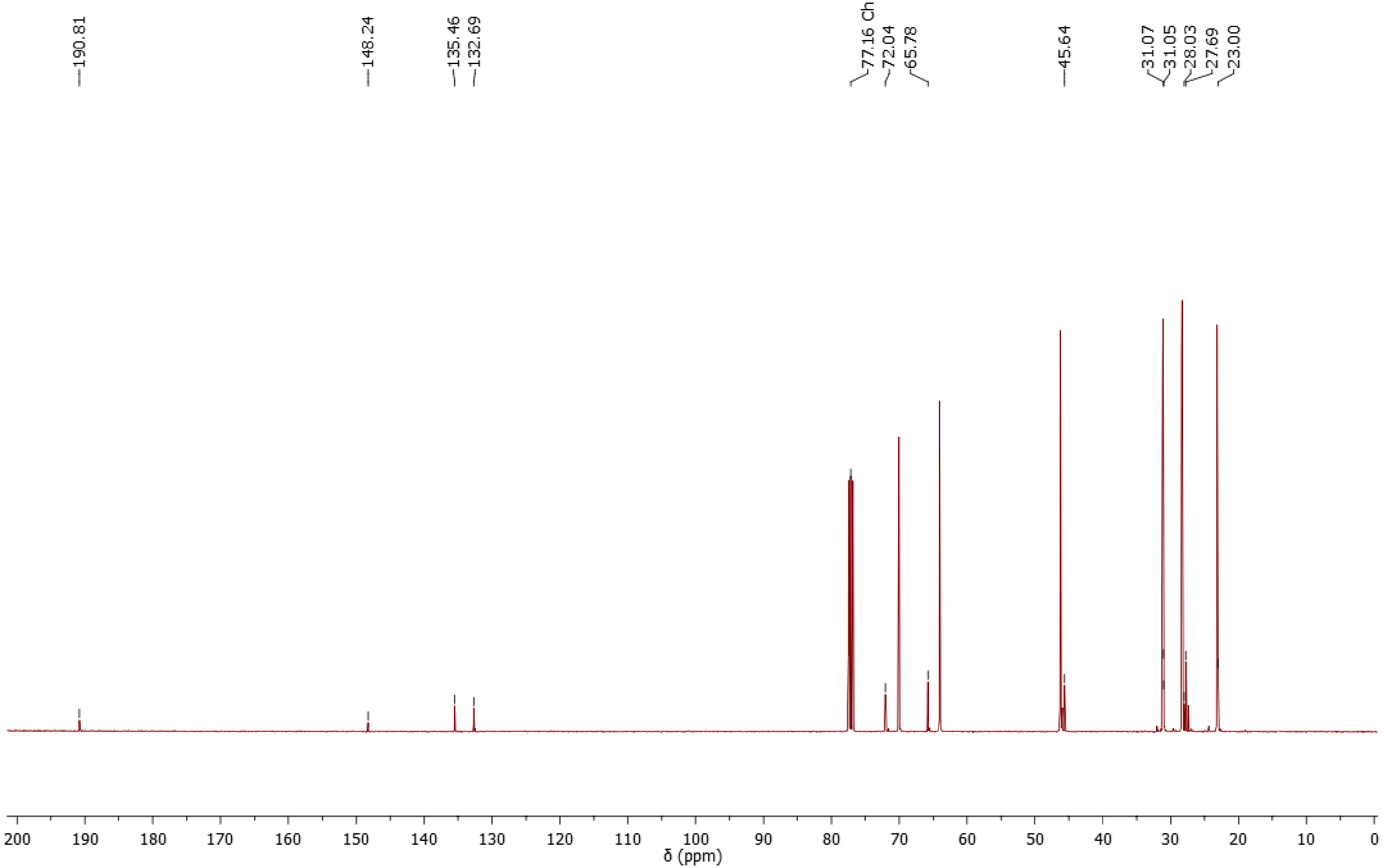

^{1}H 400MHz, $CDCl_3$

2ac

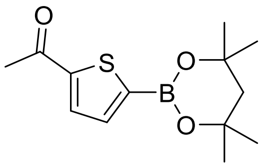

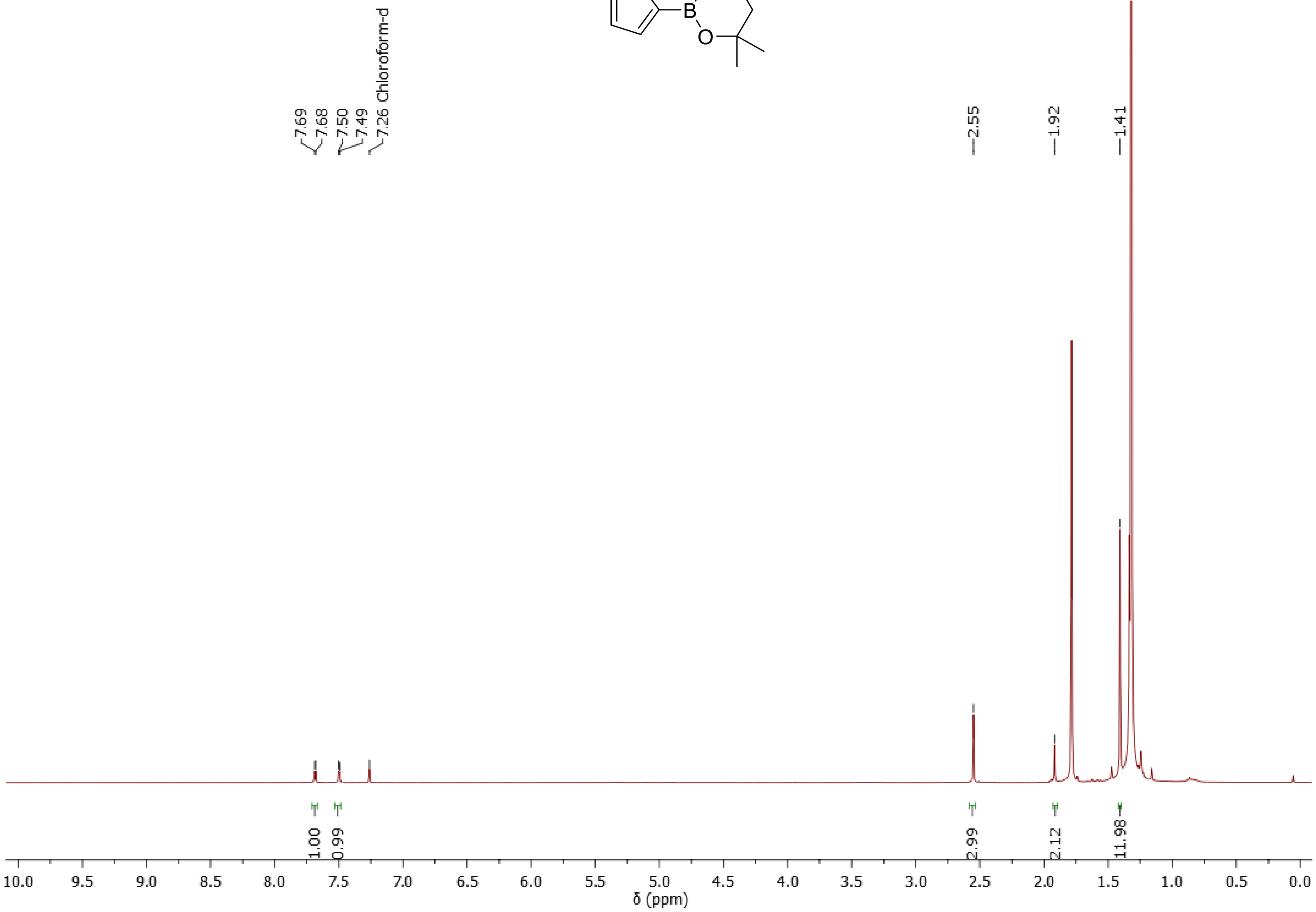

^{13}C 101MHz

2ac

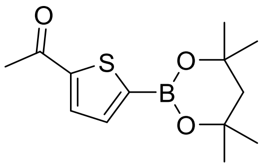

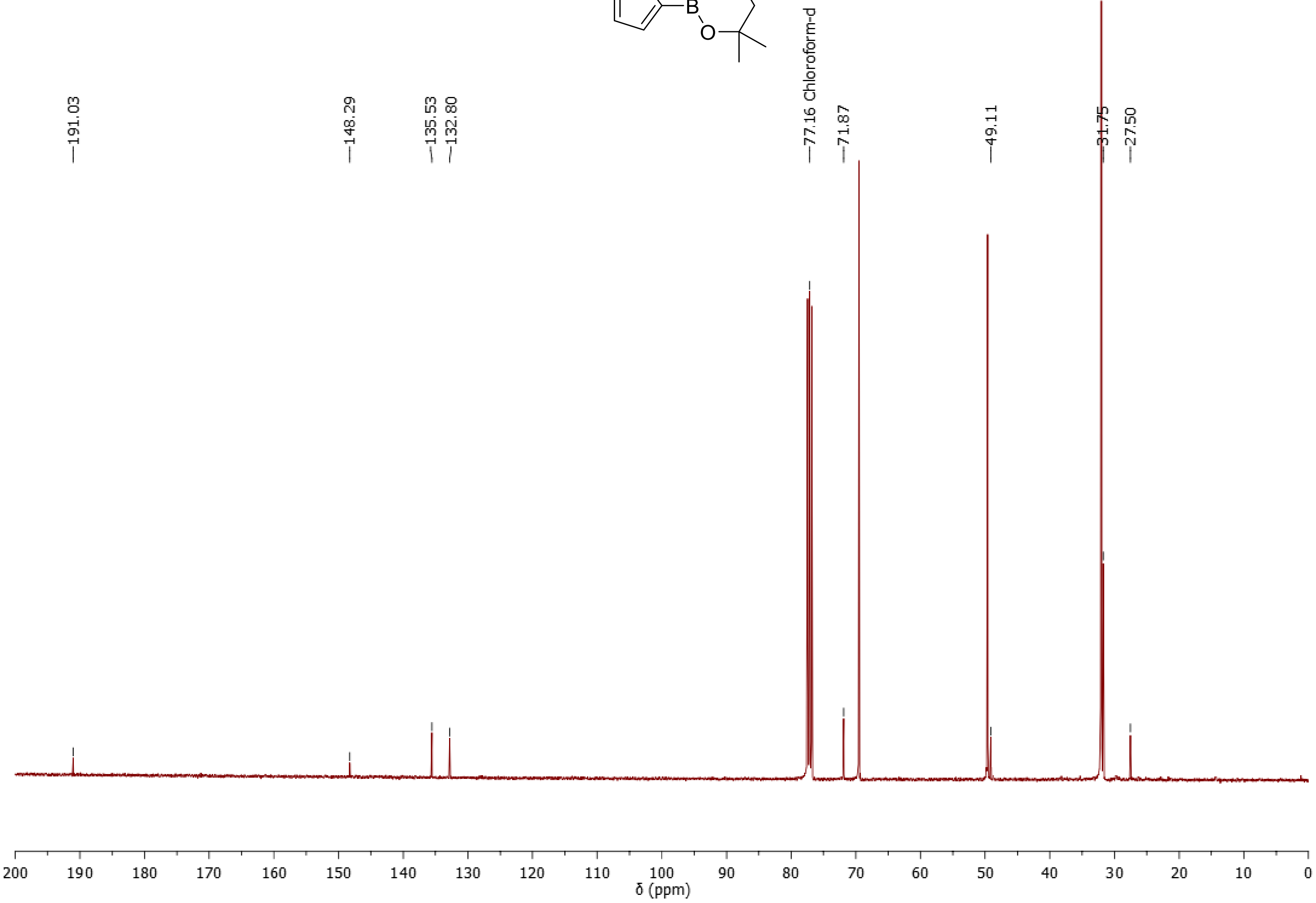

^{1}H 400MHz, $CDCl_3$

2ad

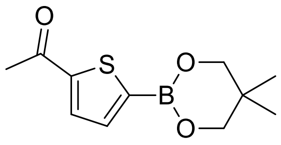

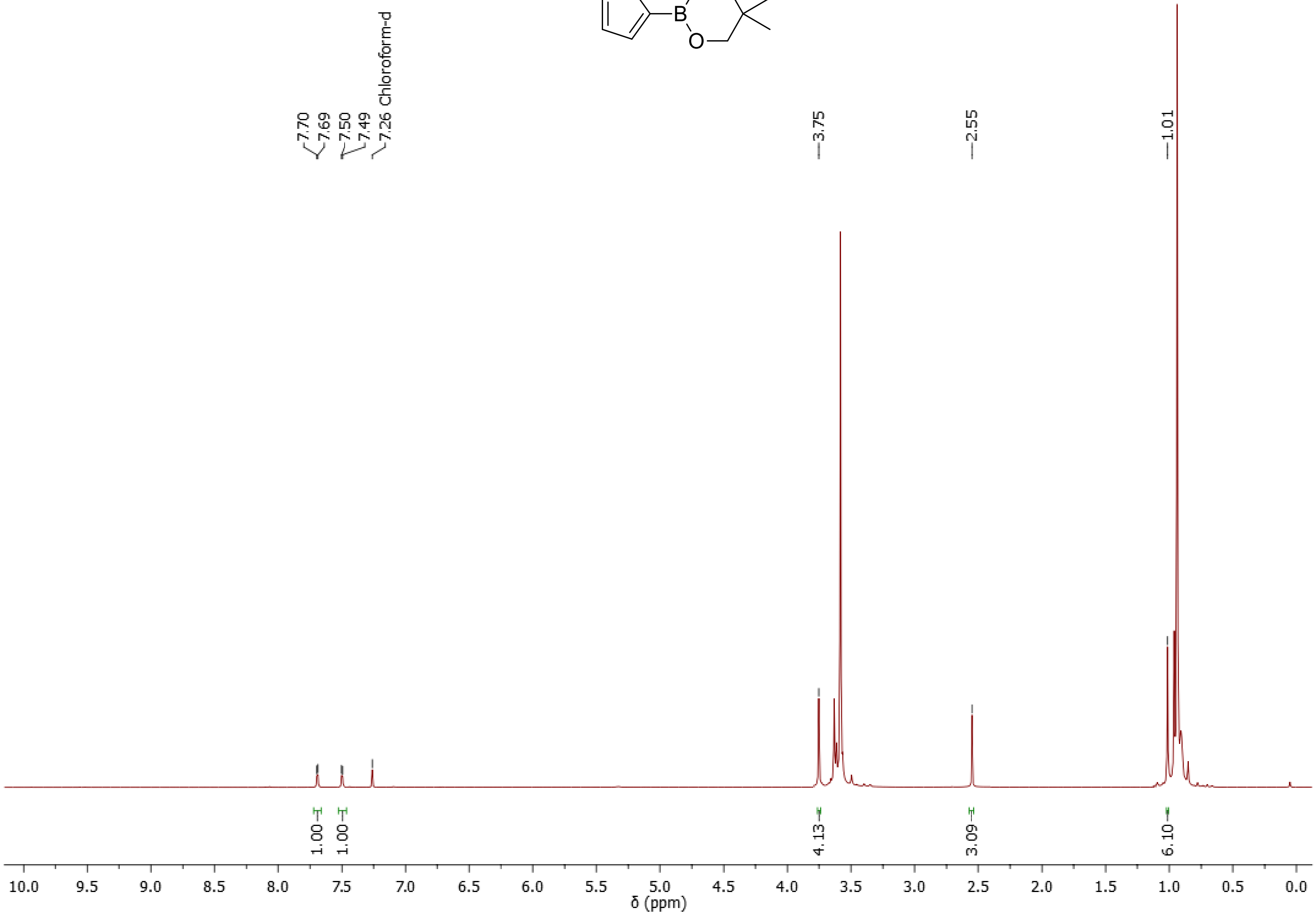

^{13}C 101MHz

2ad

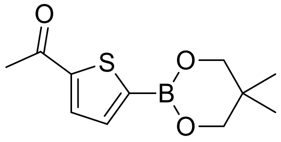

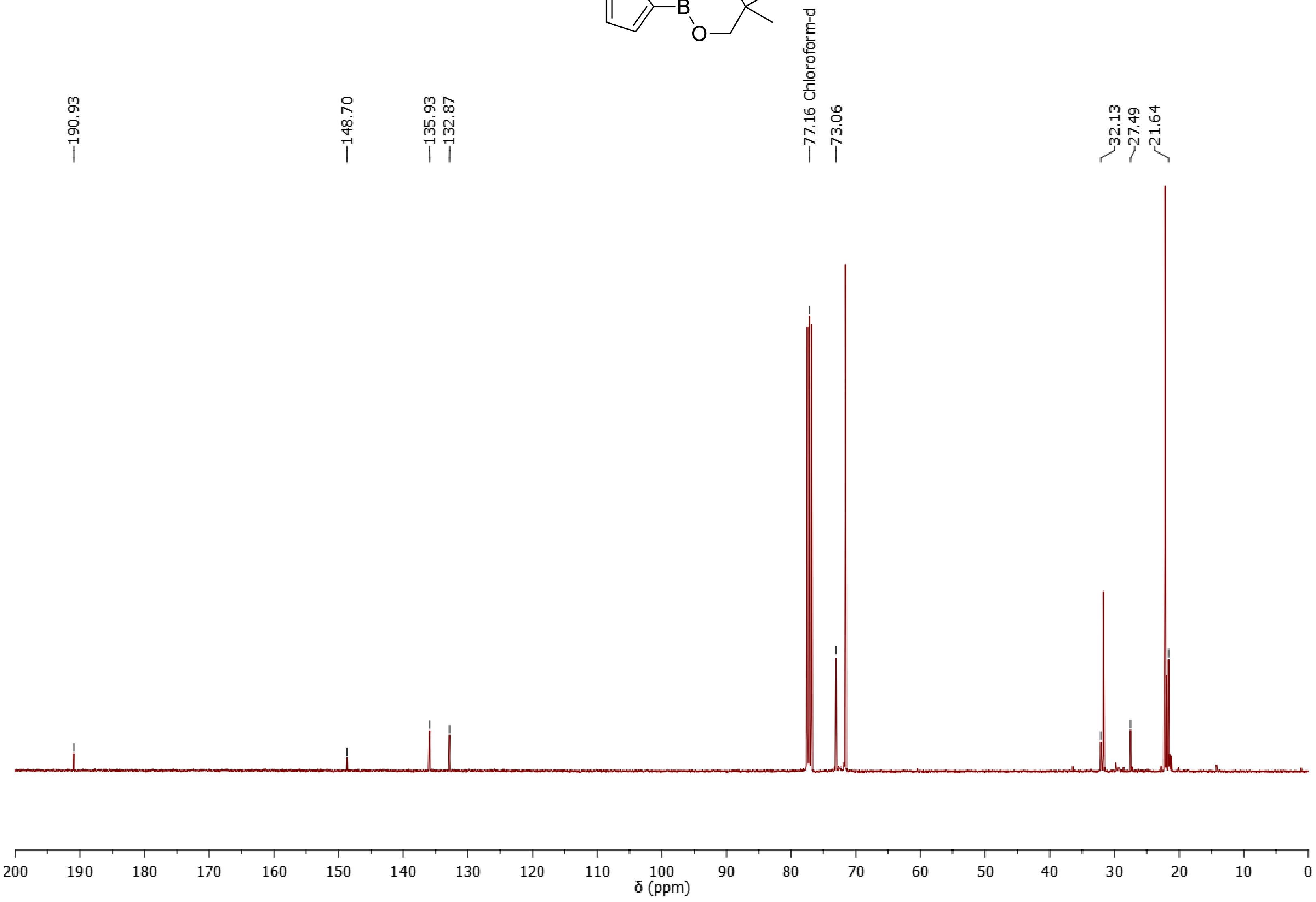

^{1}H 400MHz, $CDCl_3$

2ba

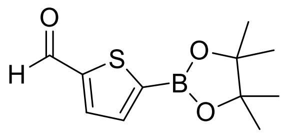

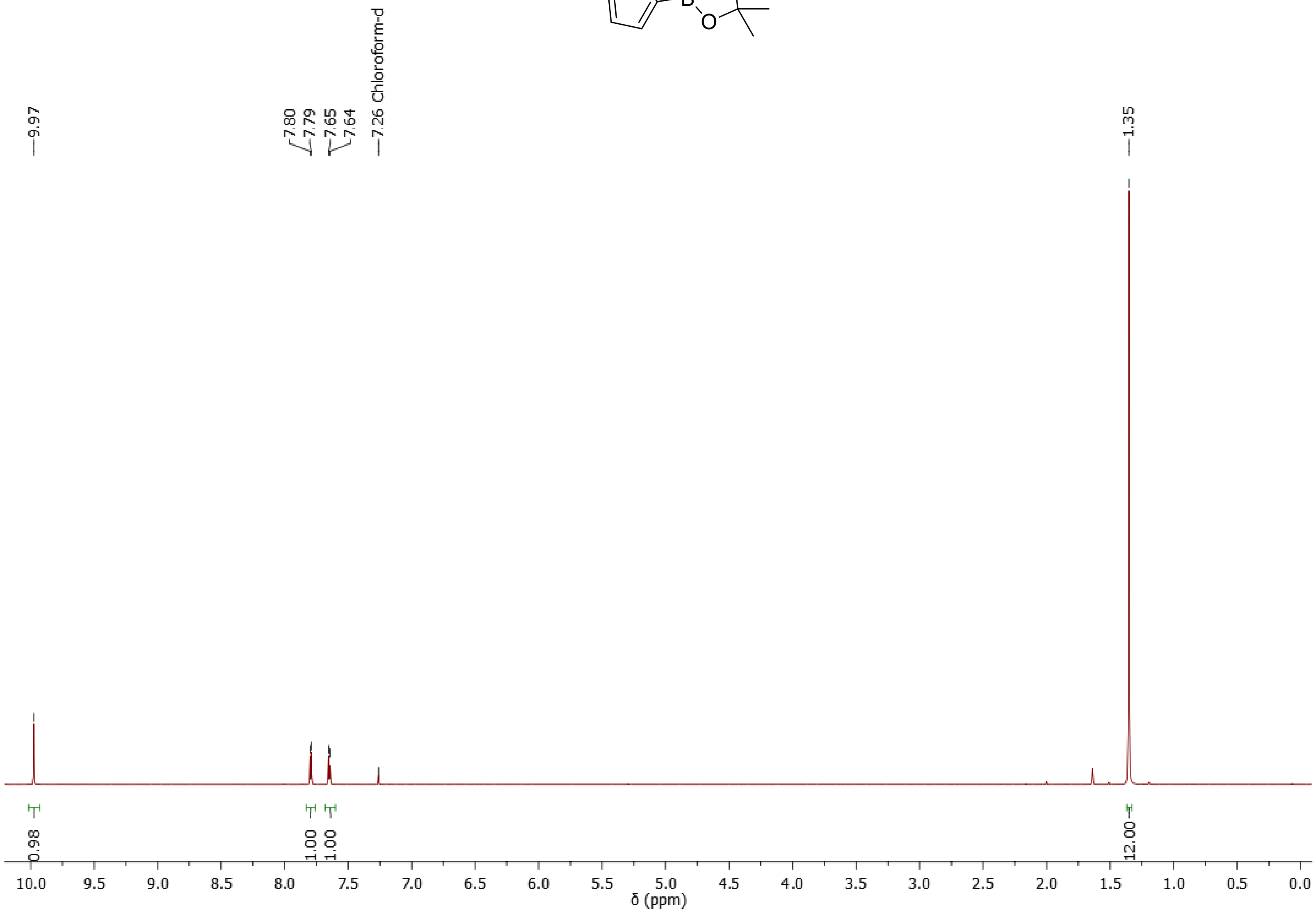

^{13}C 101MHz

2ba

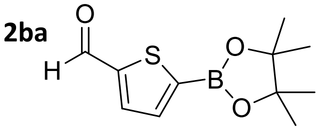

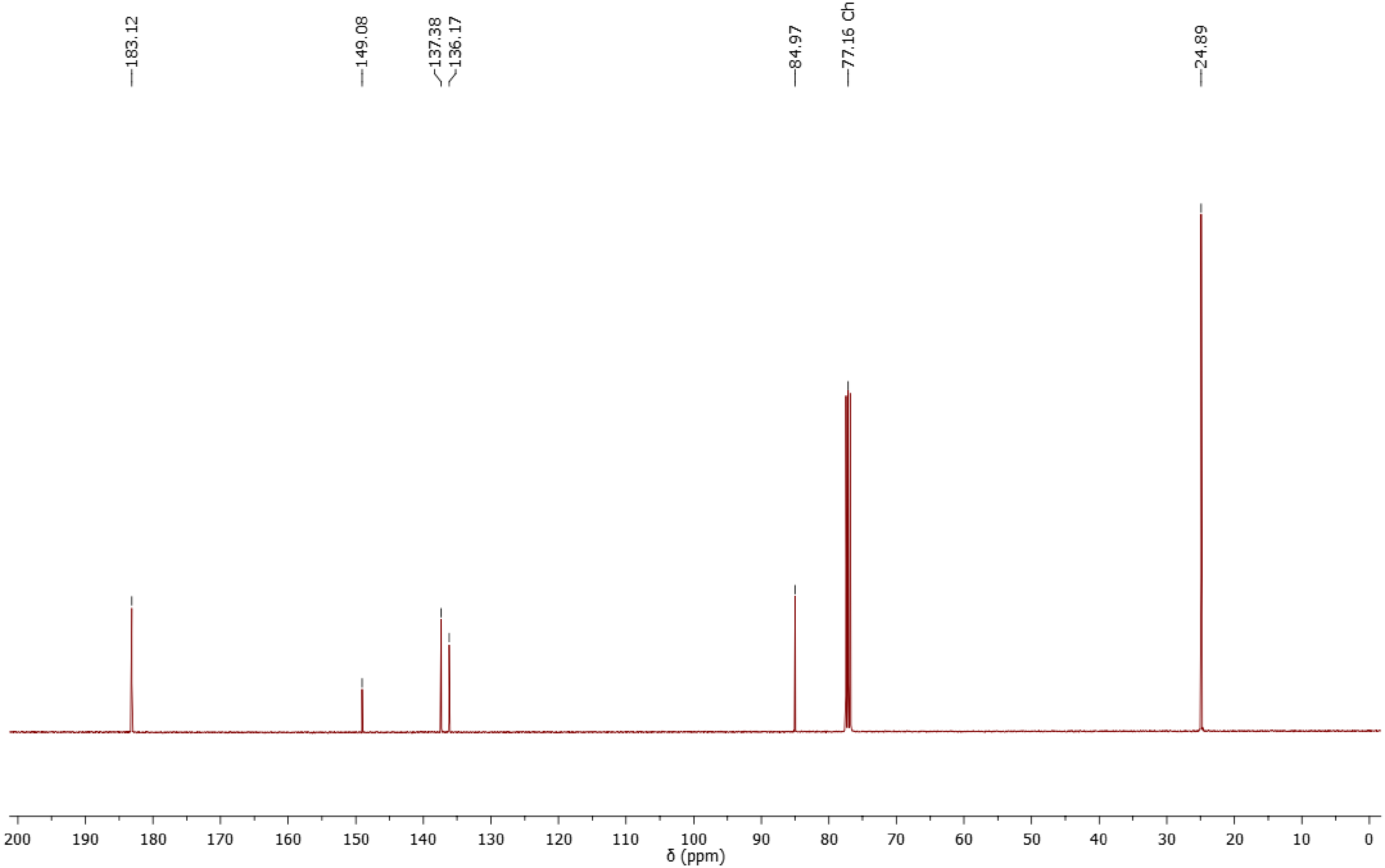

^{1}H 400MHz, $CDCl_3$

2bb

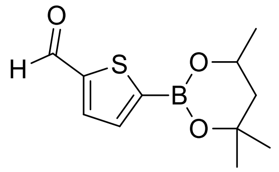

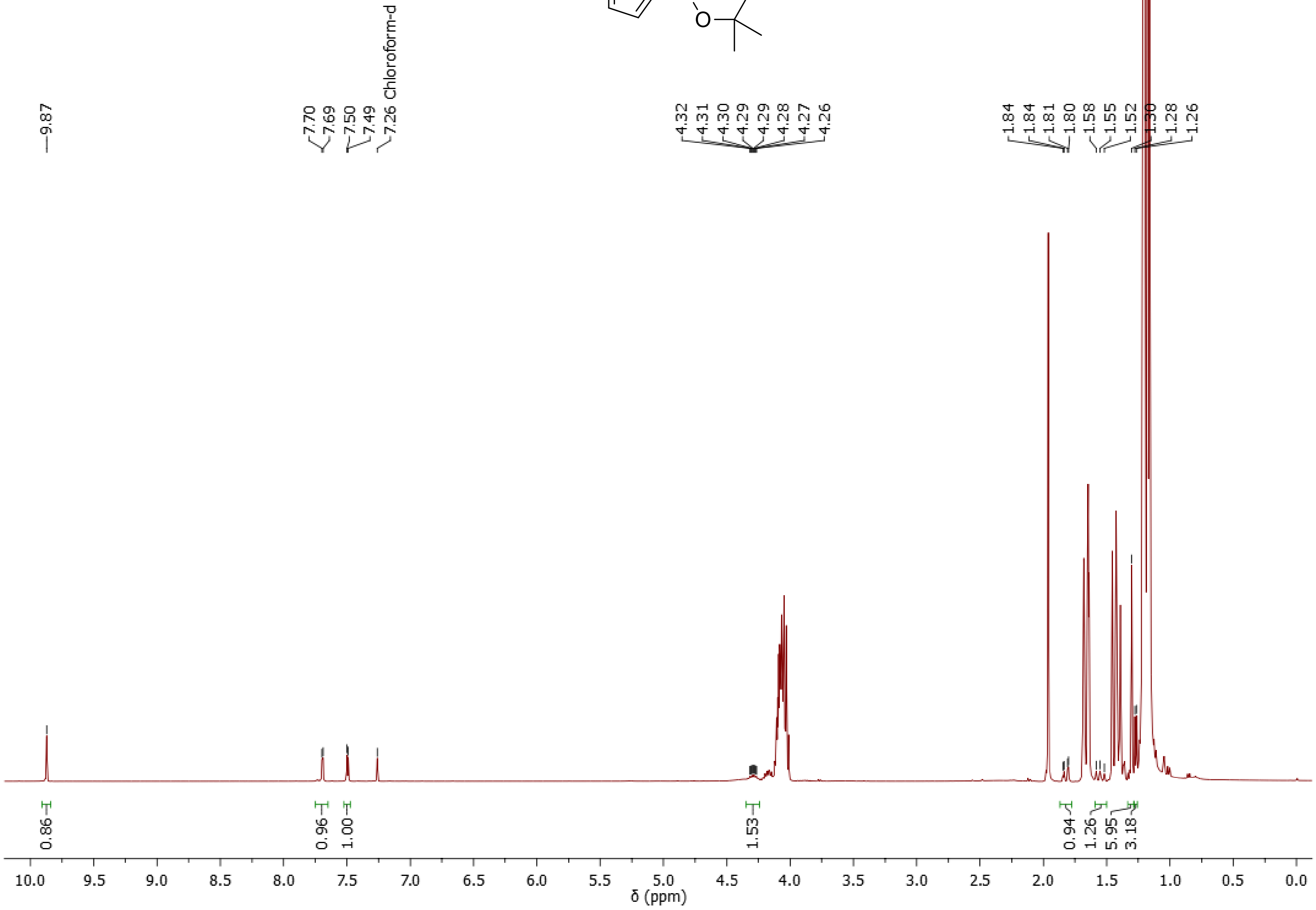

^{13}C 101MHz

2bb

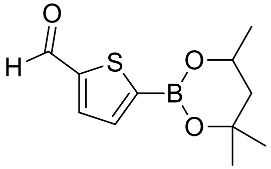

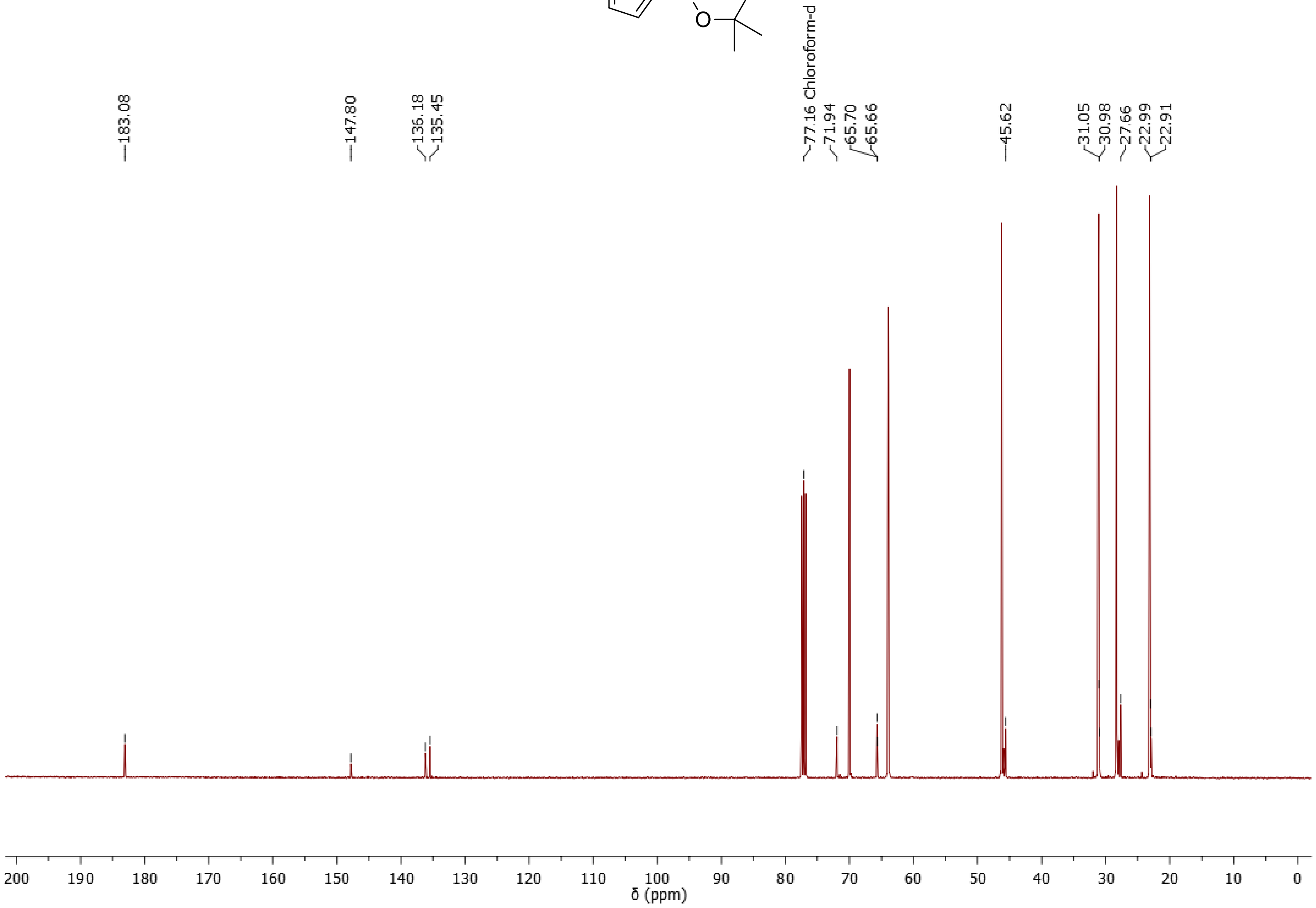

^{1}H 400MHz, $CDCl_3$

2bc

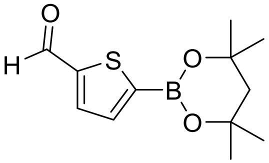

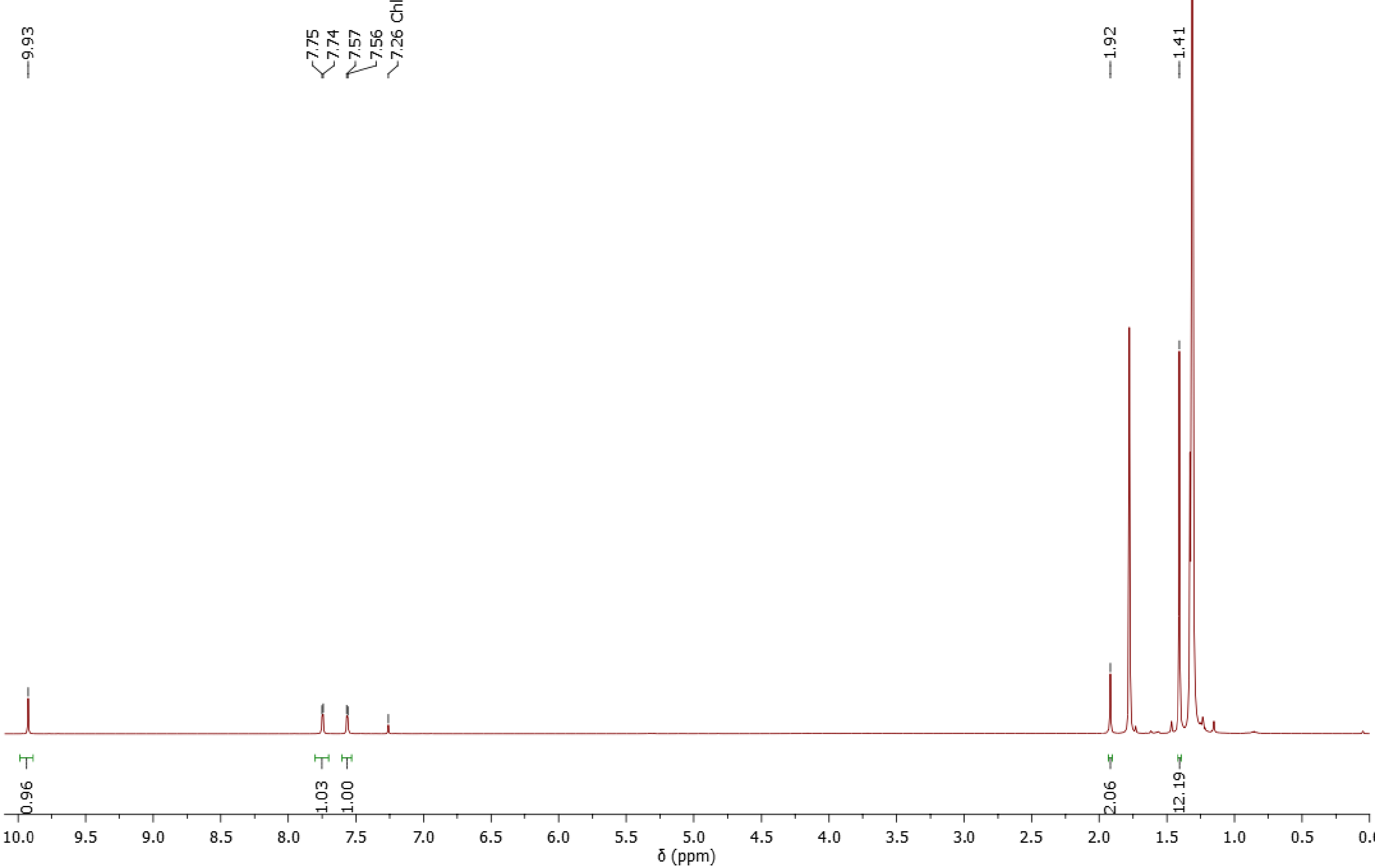

^{13}C 101MHz

2bc

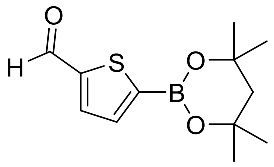

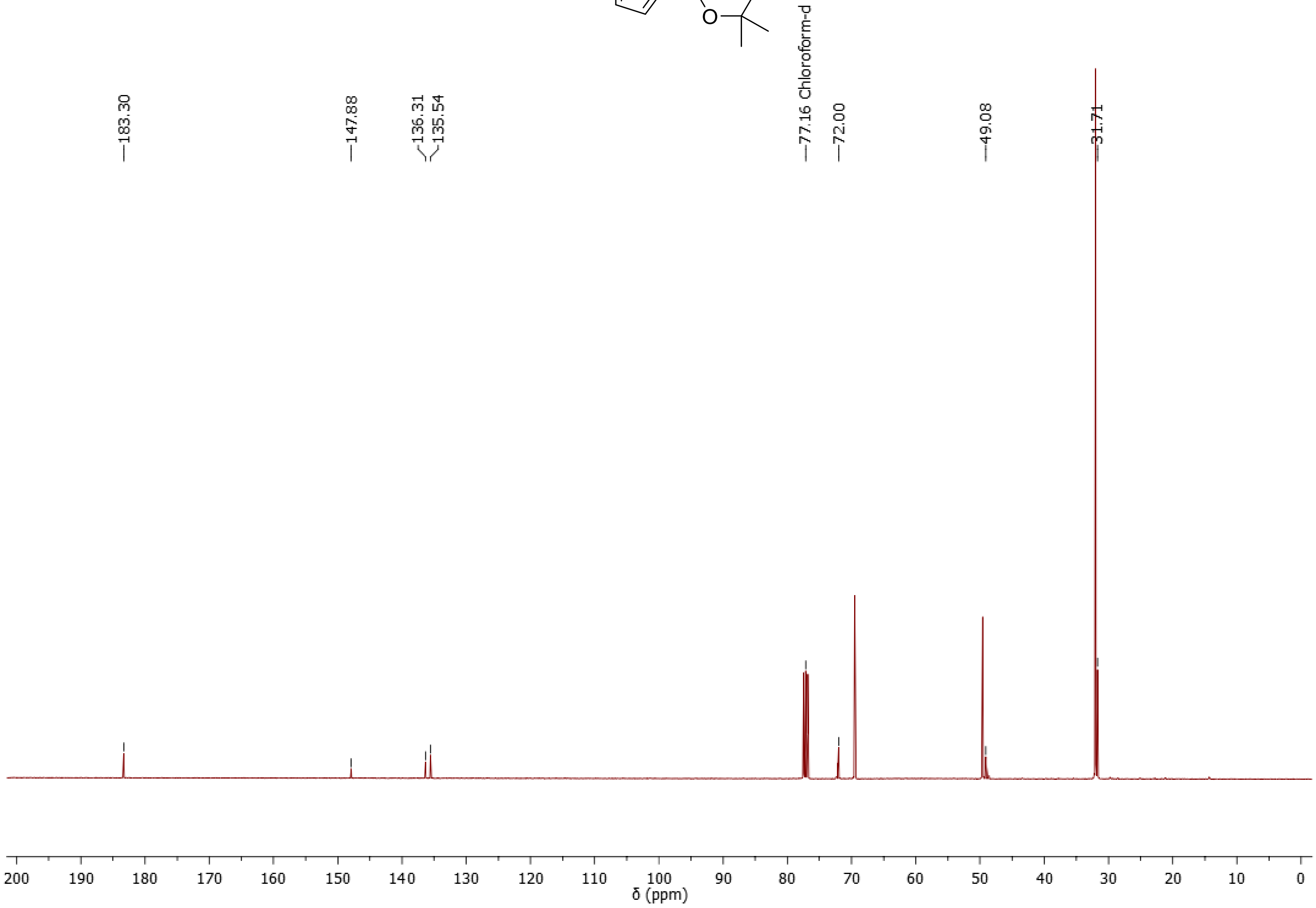

^{1}H 400MHz, $CDCl_3$

2bd

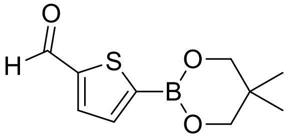

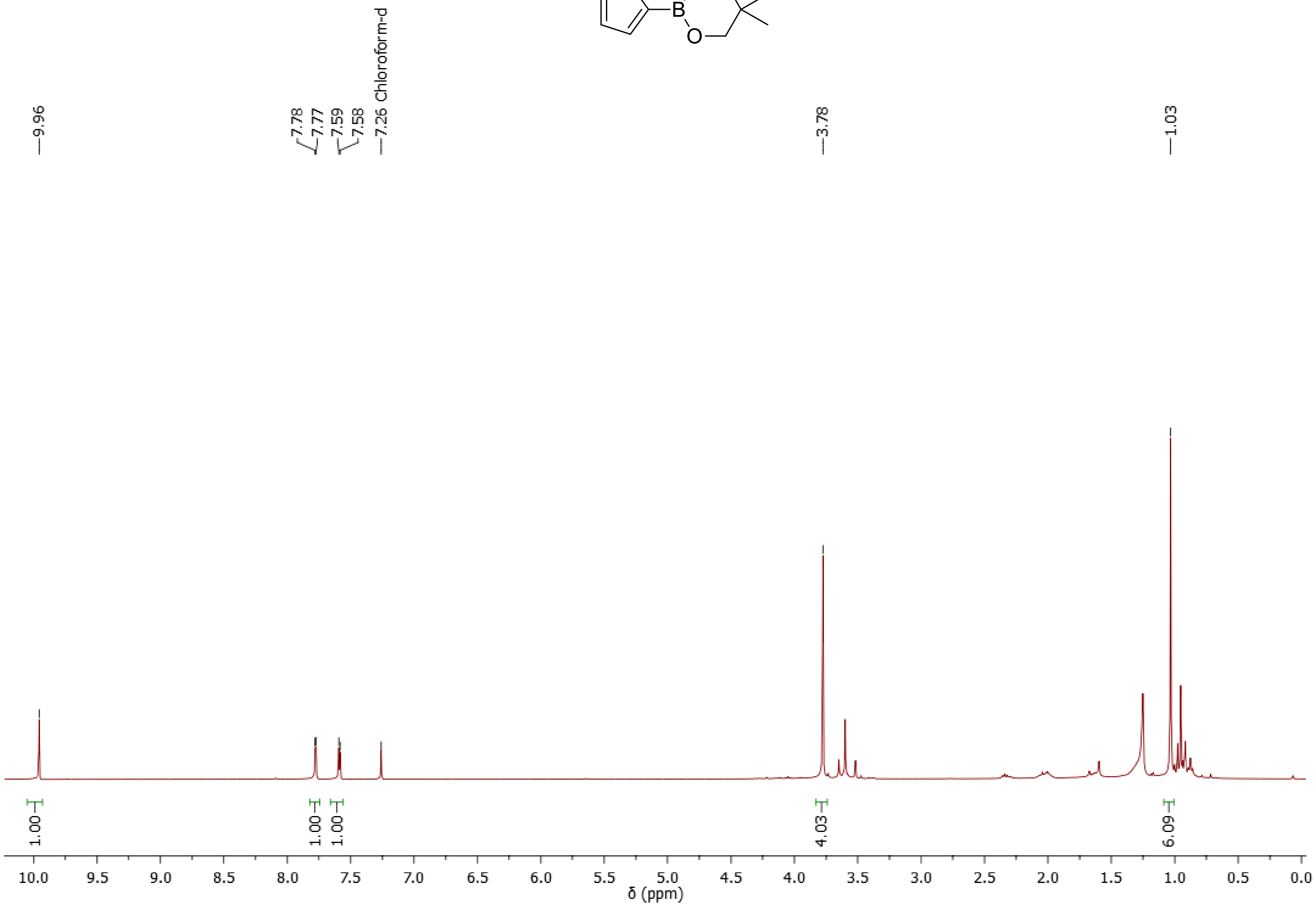

^{13}C 101MHz

2bd

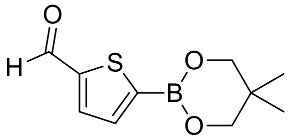

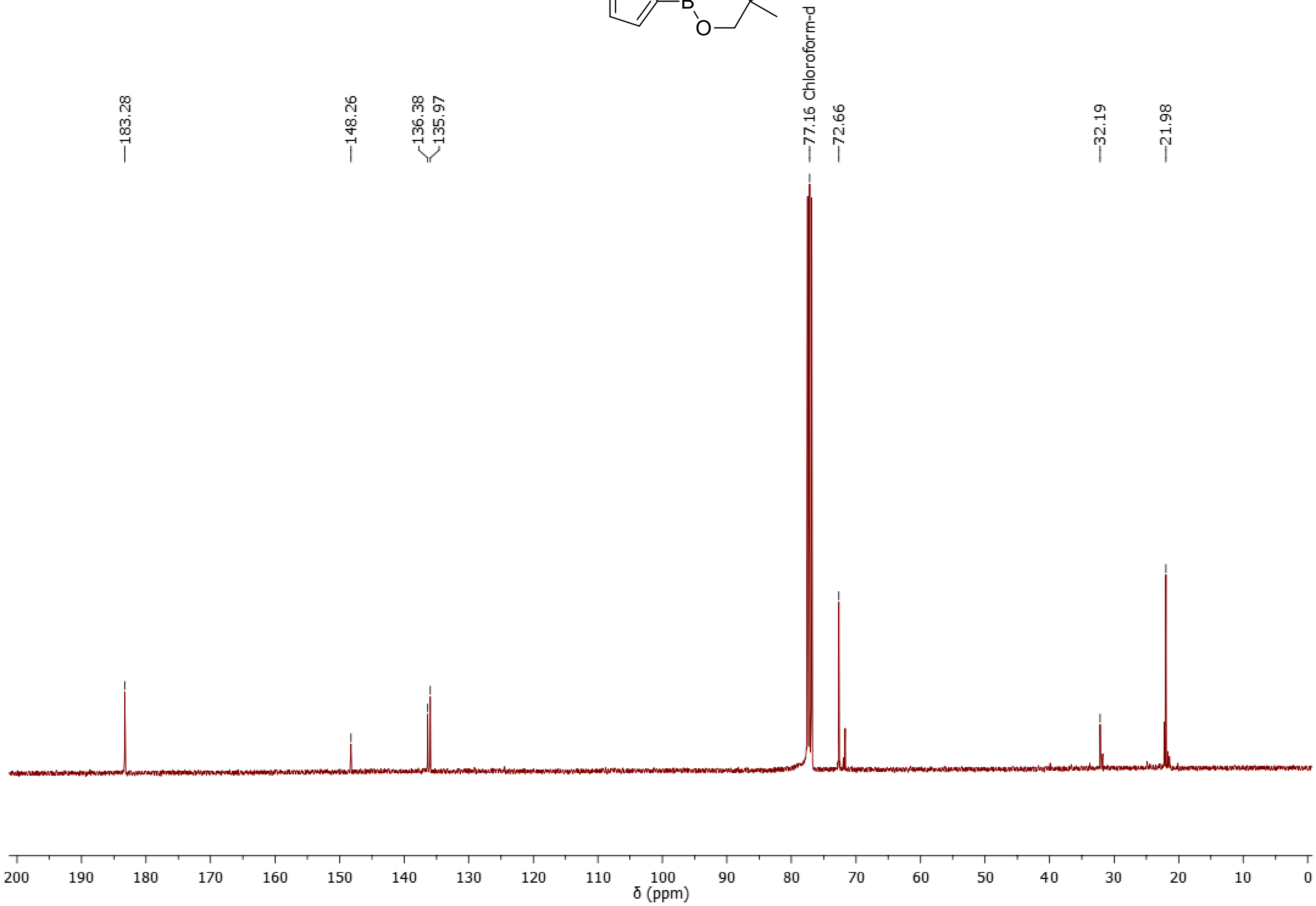

^{1}H 400MHz, $CDCl_3$

2ca

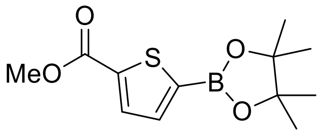

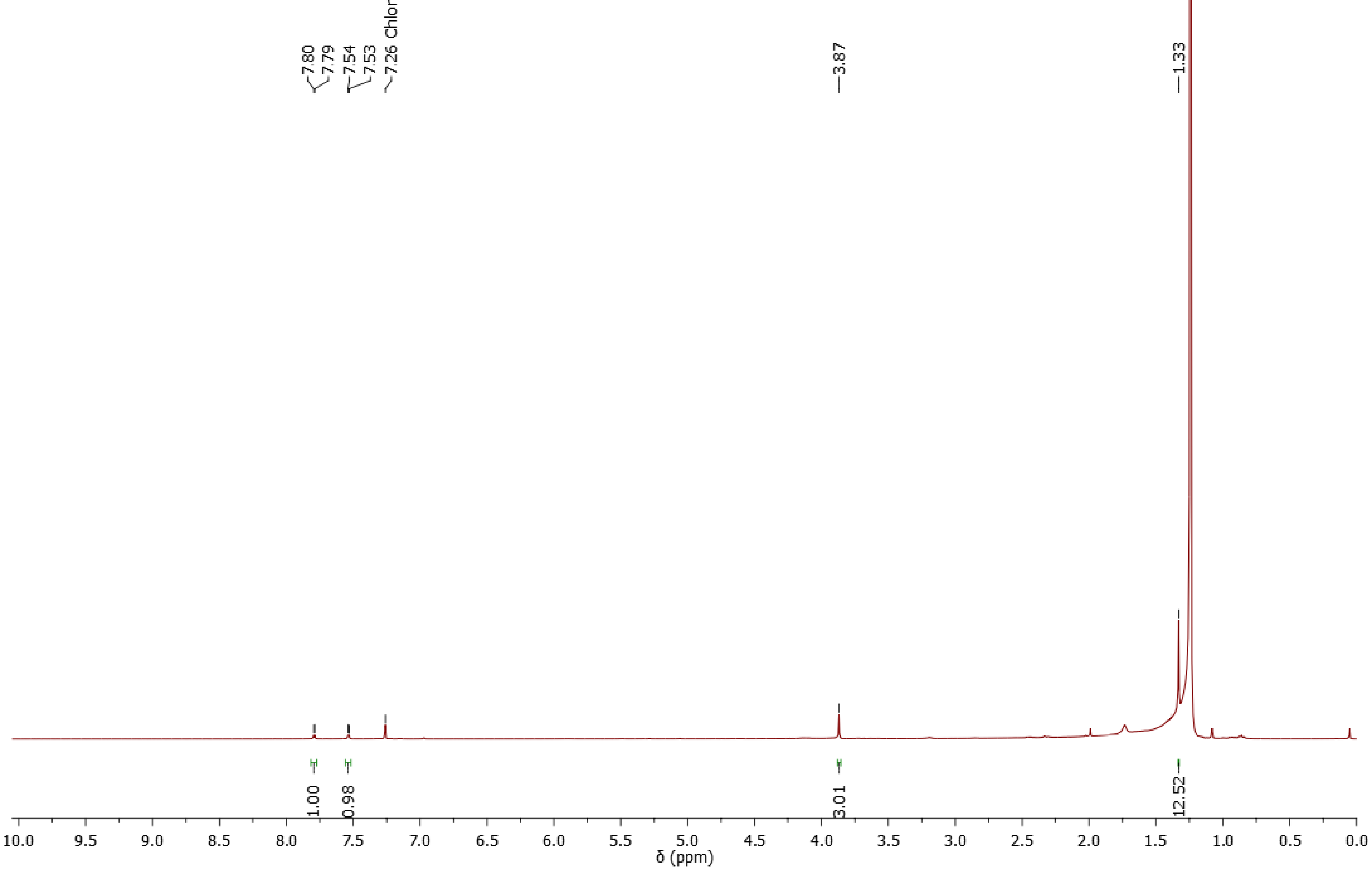

^{13}C 101MHz, $CDCl_3$

2ca

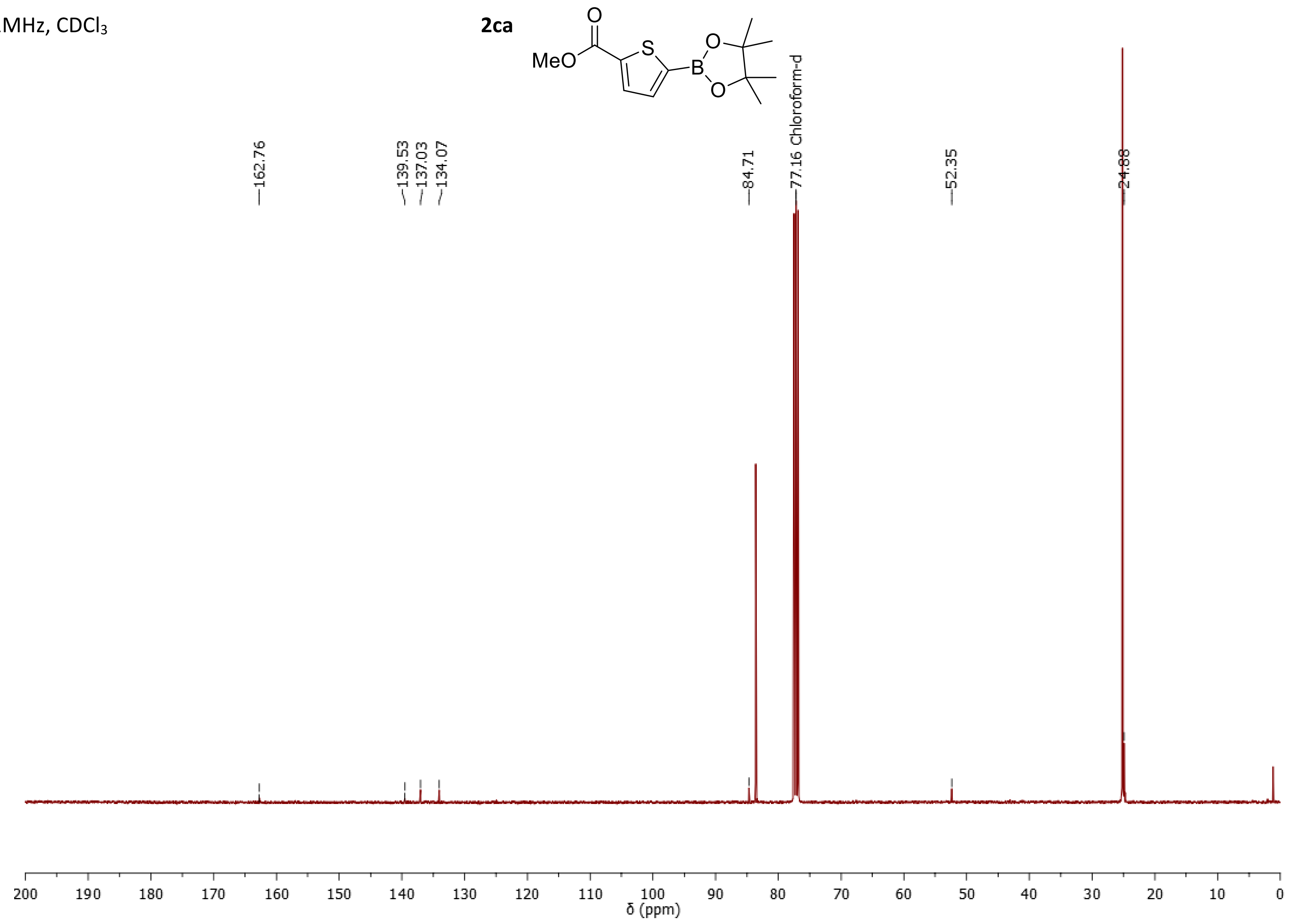

^{1}H 400MHz, $CDCl_3$

2cb

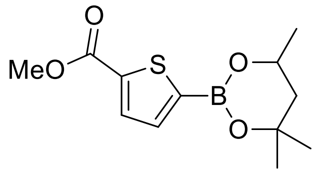

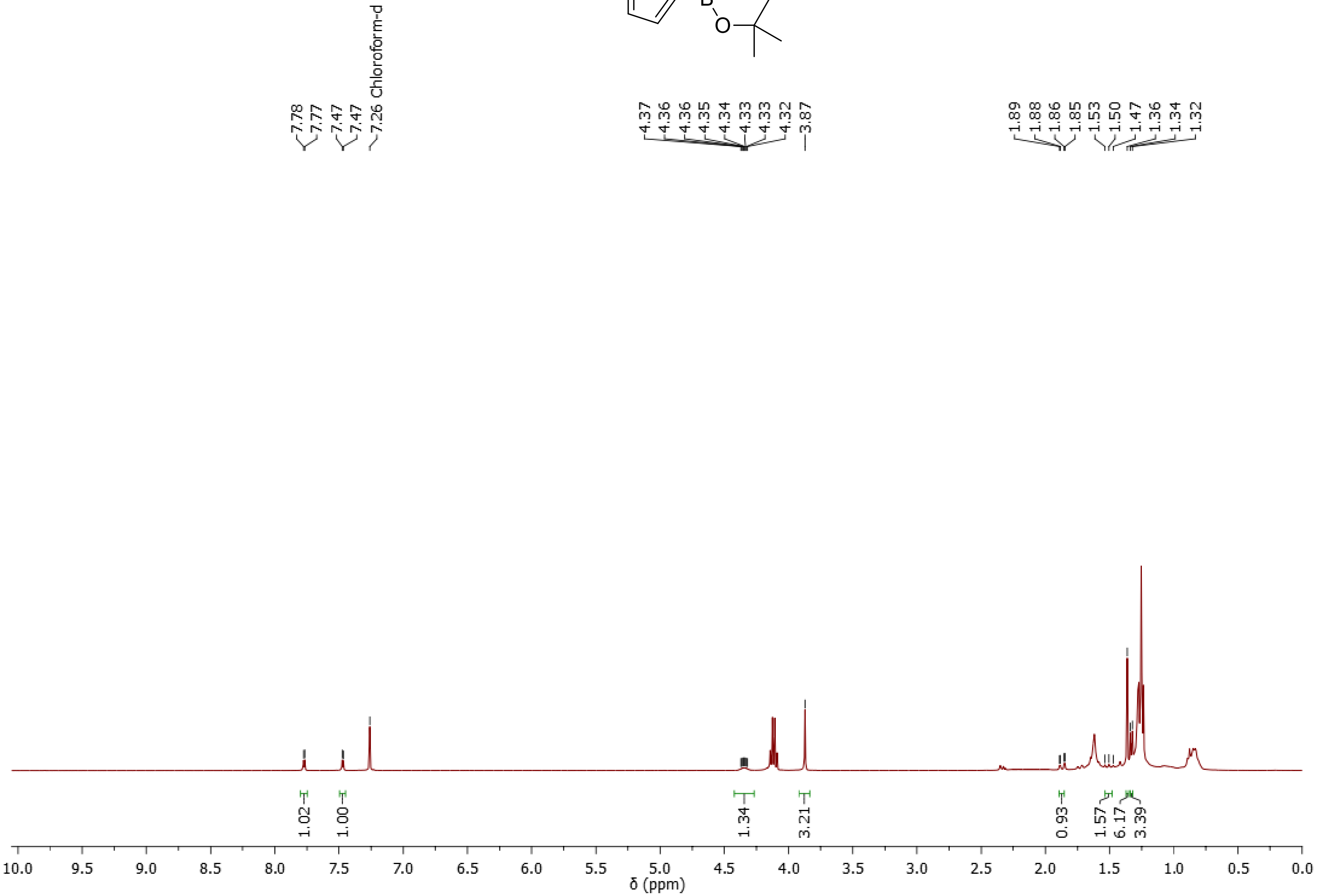

^{13}C 101MHz

2cb

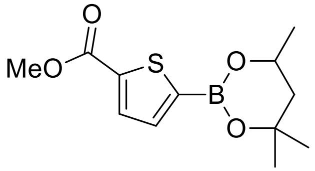

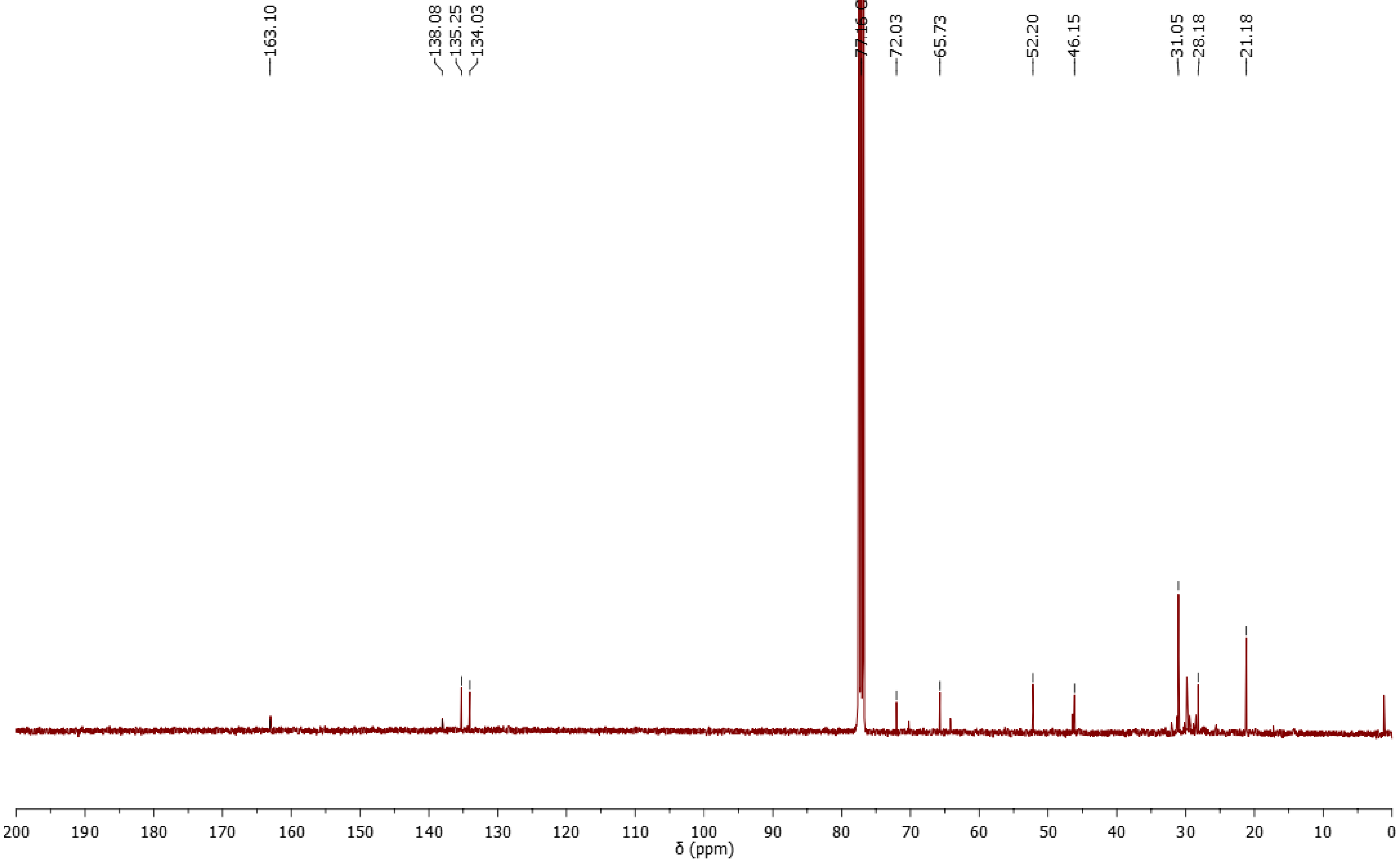

^{1}H 400MHz, $CDCl_3$

2cc

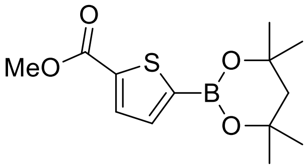

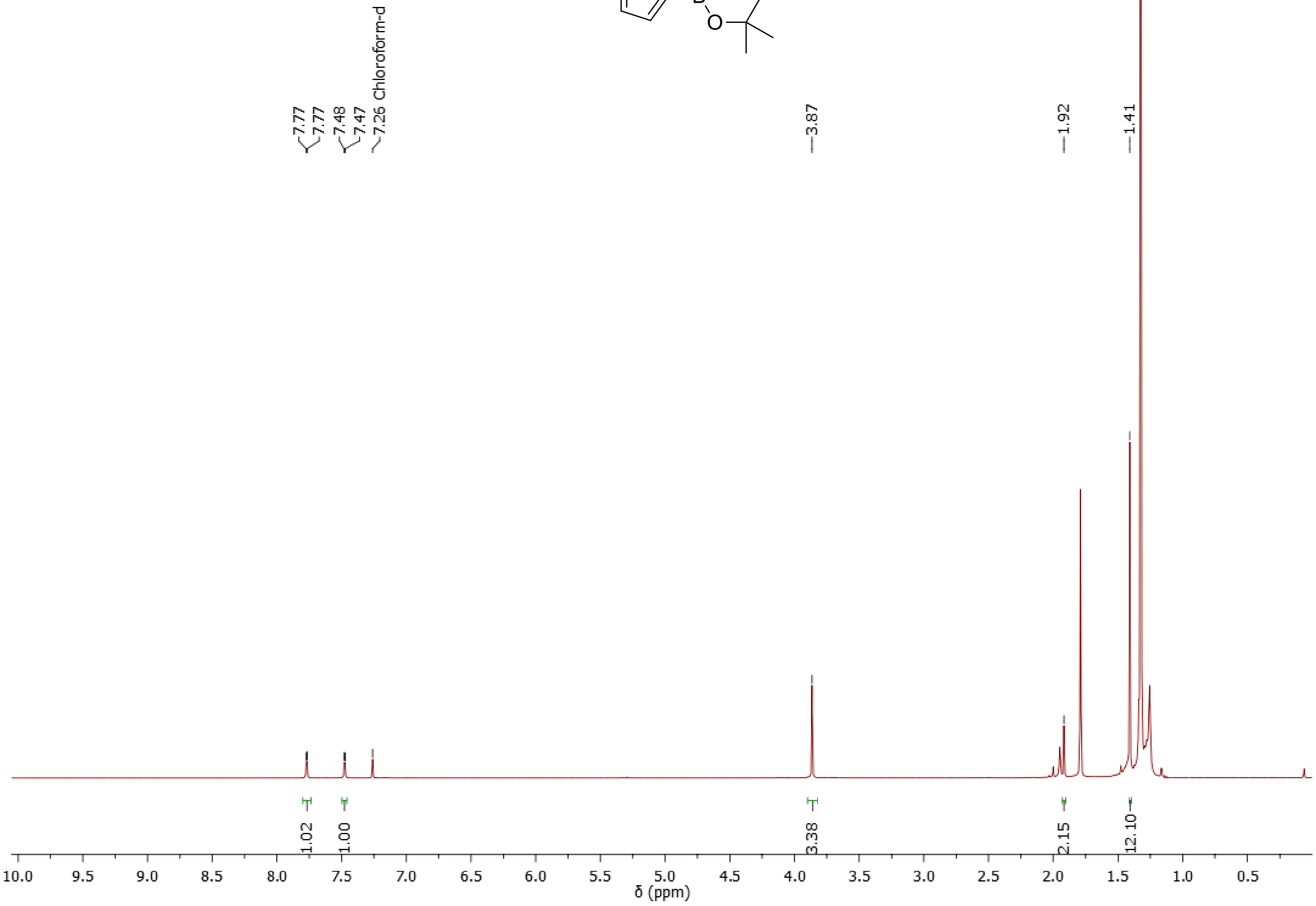

^{13}C 101MHz

2cc

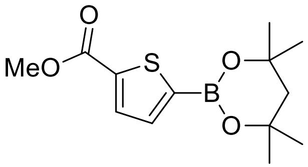

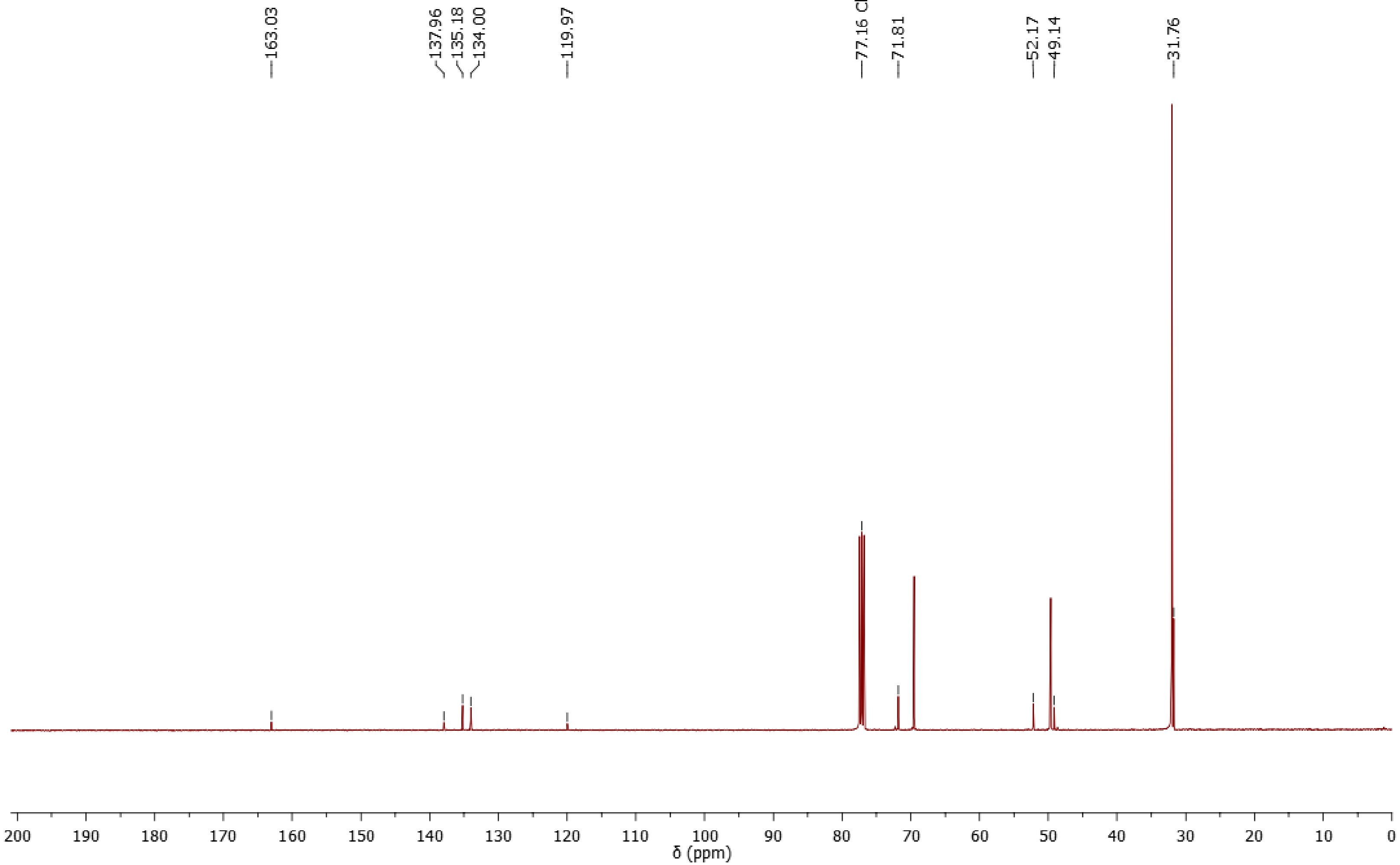

^{1}H 400MHz, $CDCl_3$

2cd

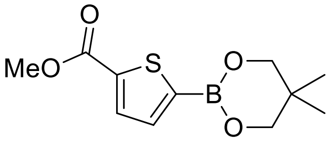

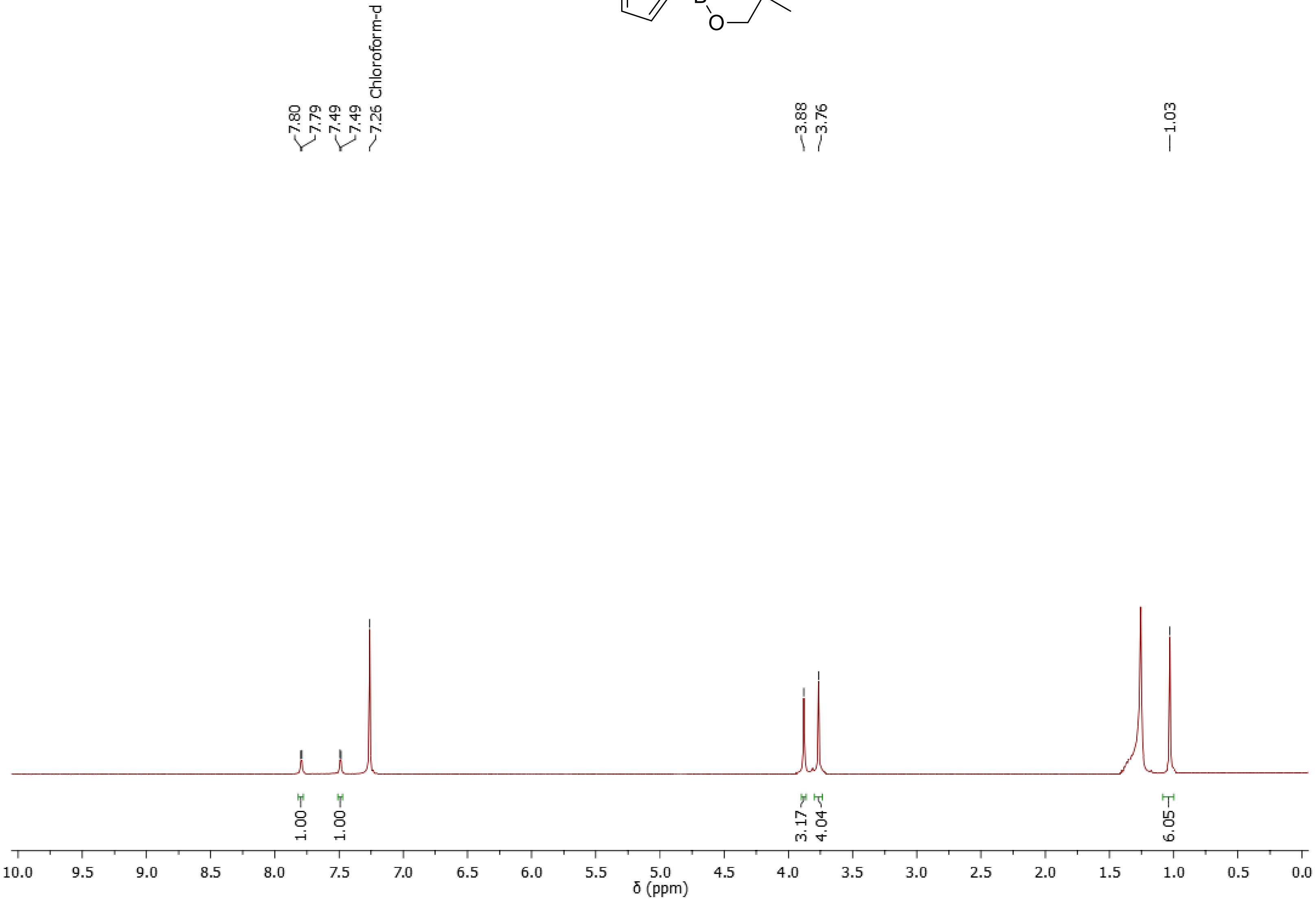

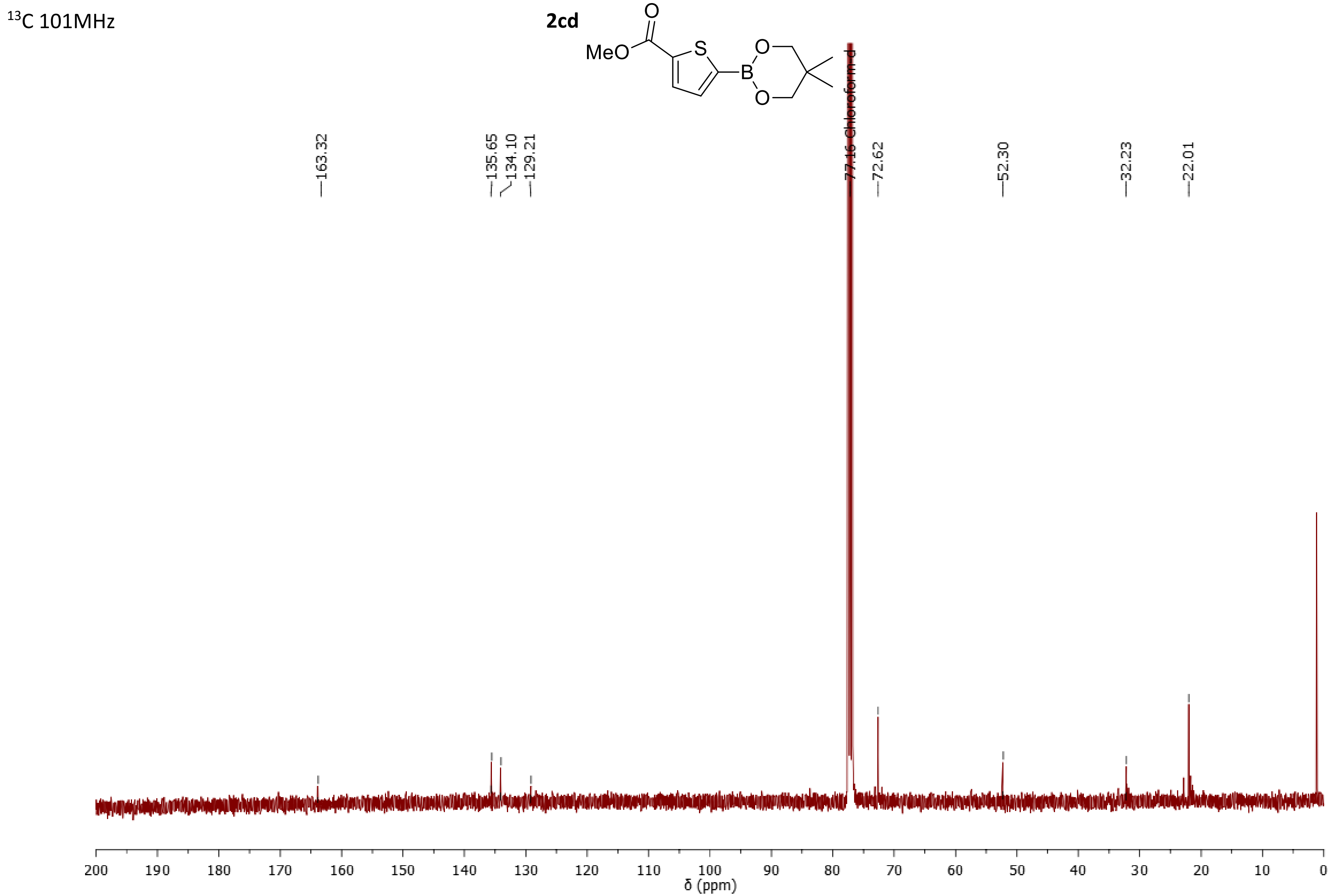

13C 101MHz
2cd
MeO
O
S
B
O
O
163.32
135.65
134.10
129.21
77.16 Chloroform-d
72.62
52.30
32.23
22.01
200
190
180
170
160
150
140
130
120
110
100
90
80
70
60
50
40
30
20
10
0
δ (ppm)

^{1}H 400MHz, $CDCl_3$

2da

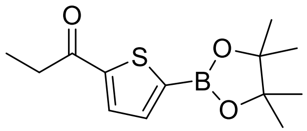

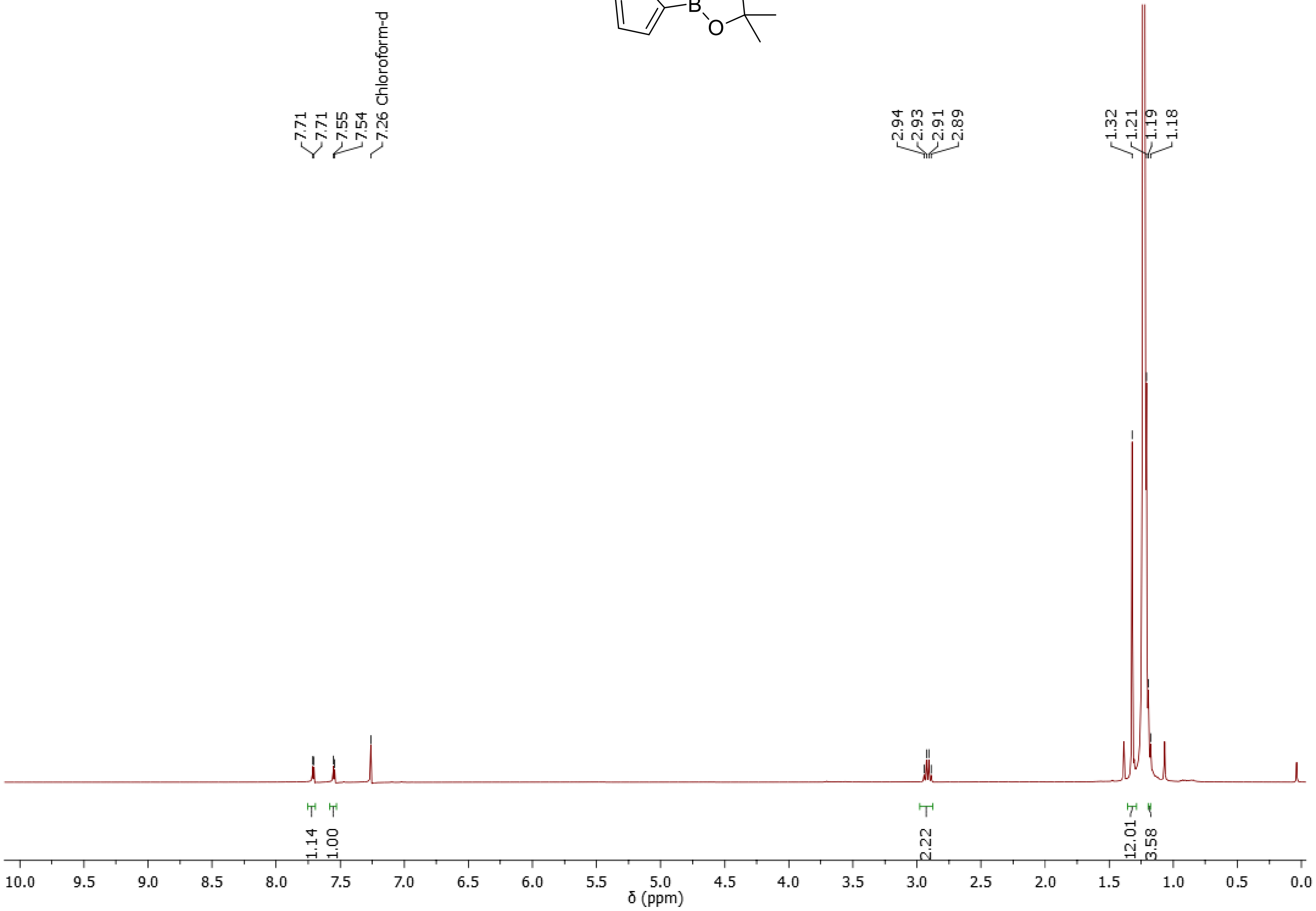

^{13}C 101MHz

2da

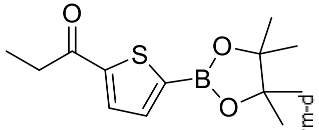

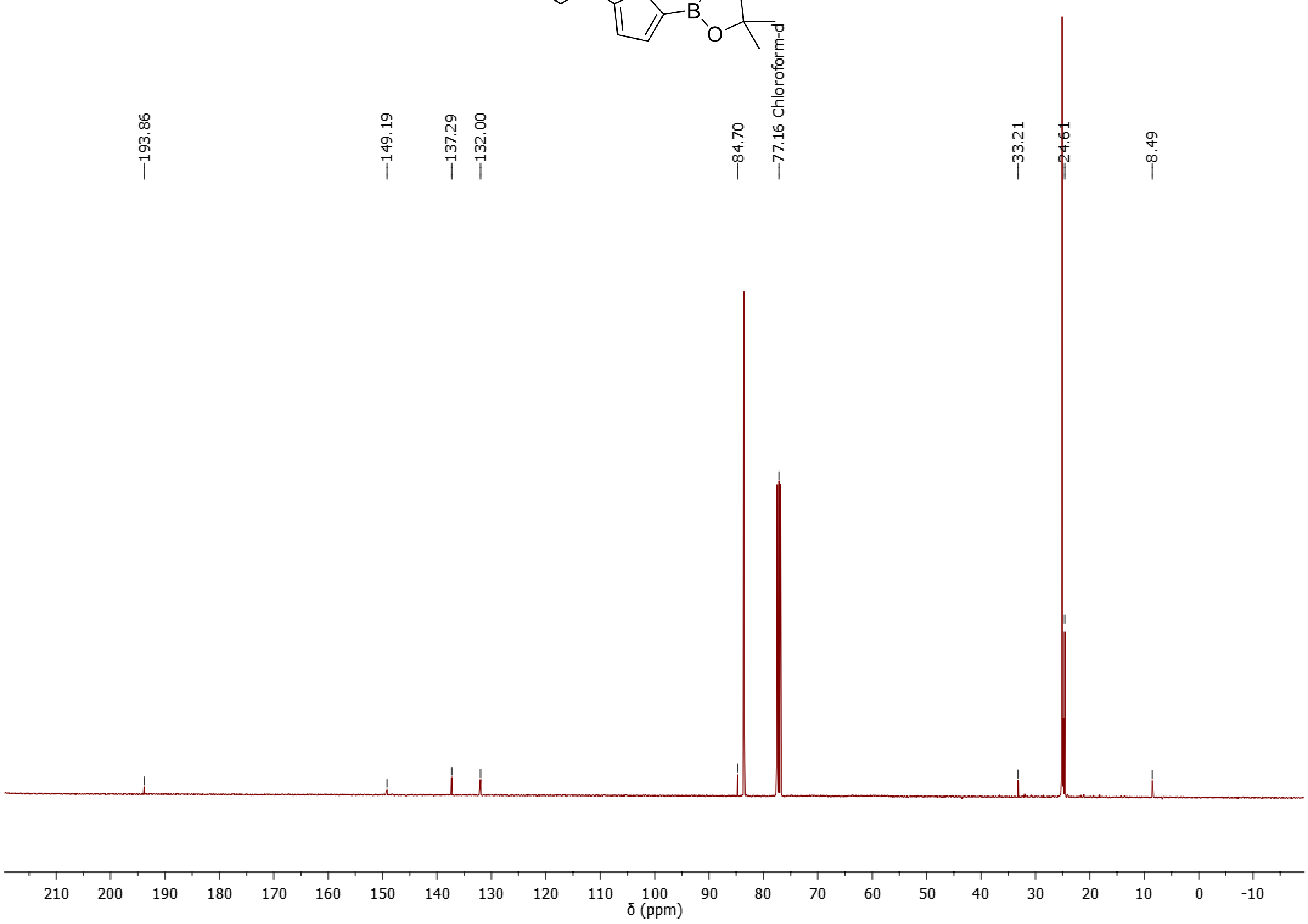

^{1}H 400MHz, $CDCl_3$

2db

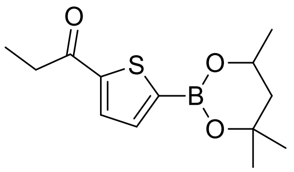

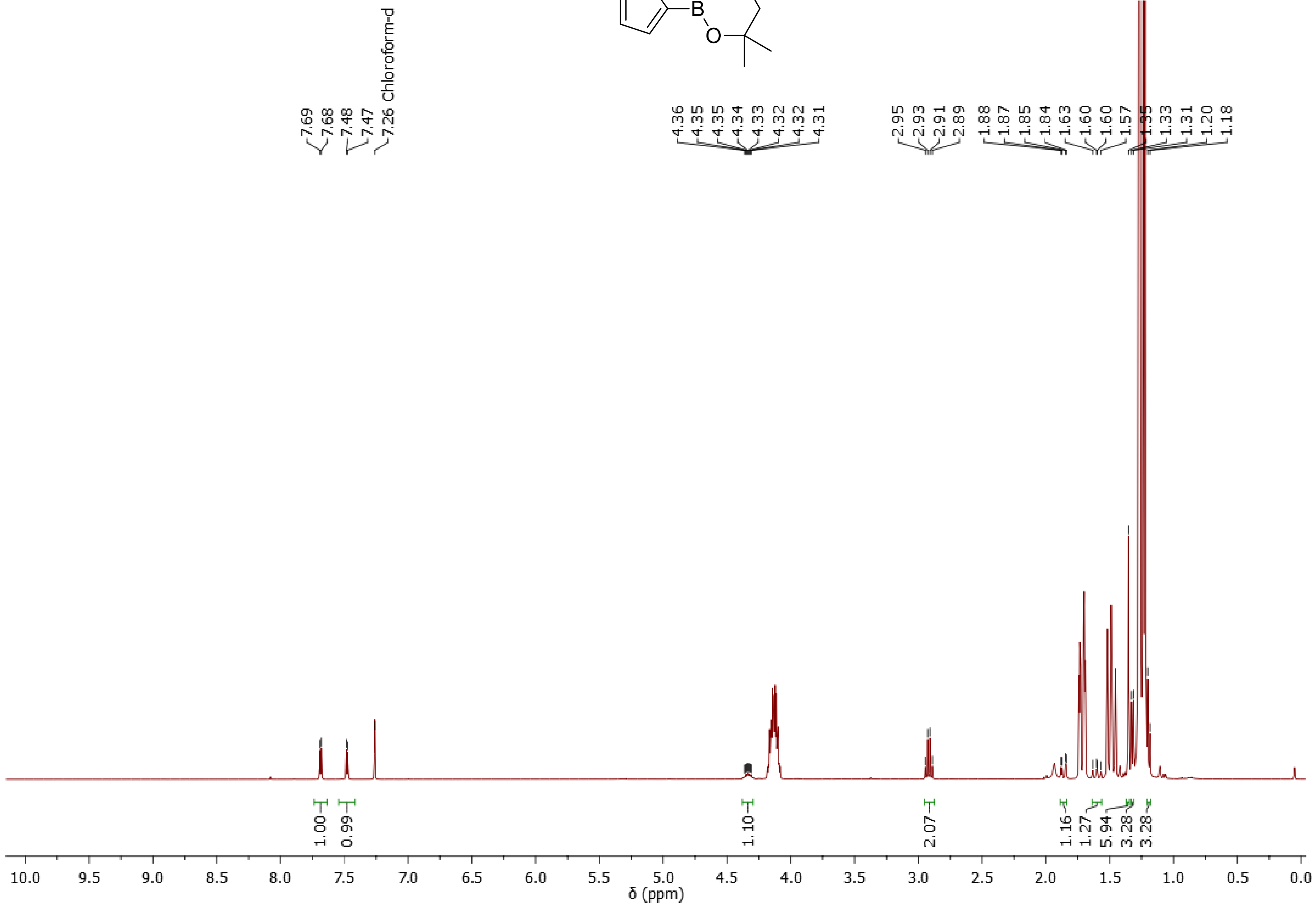

^{13}C 101MHz

2db

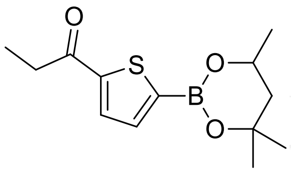

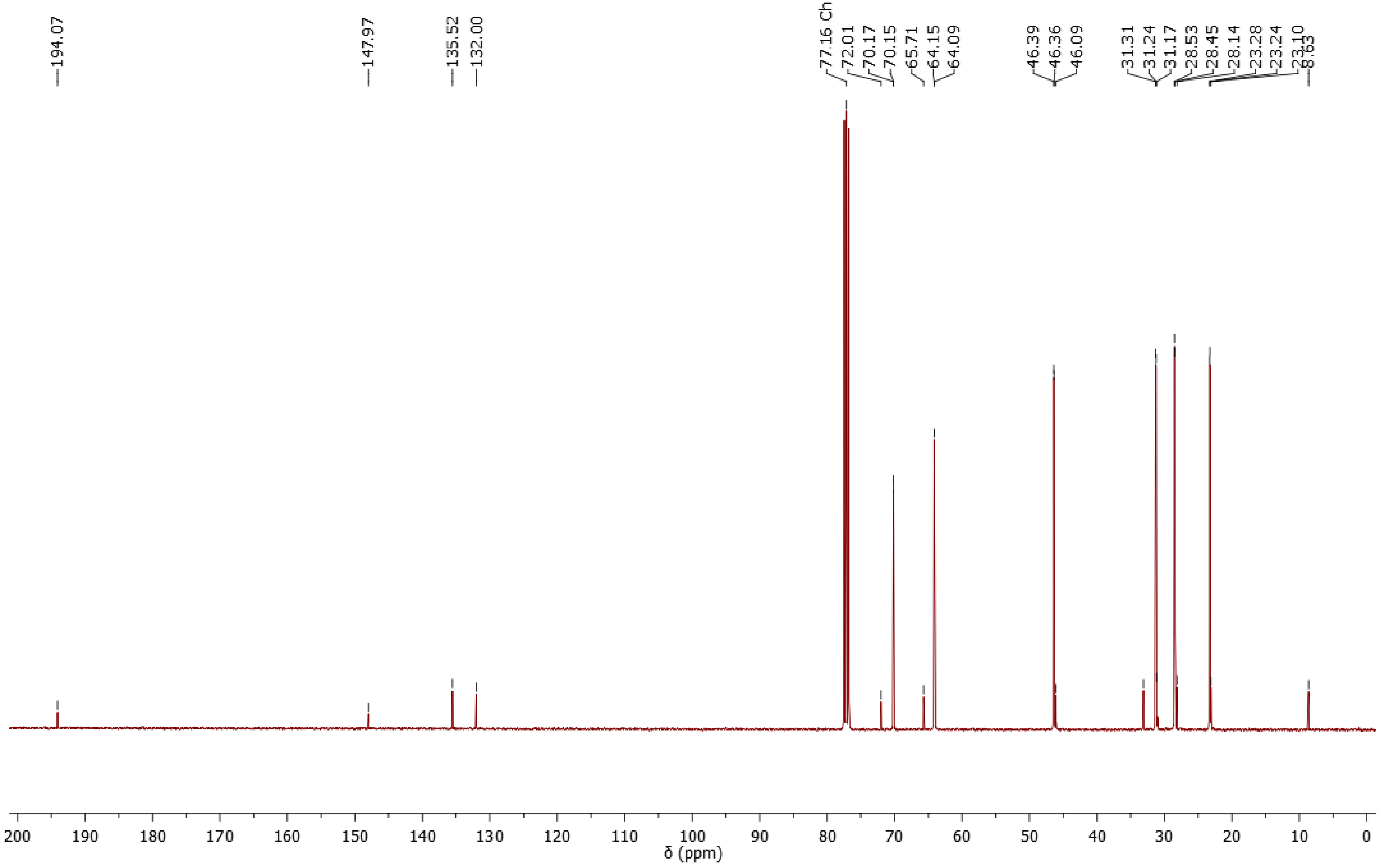

^{1}H 400MHz, $CDCl_3$

2dc

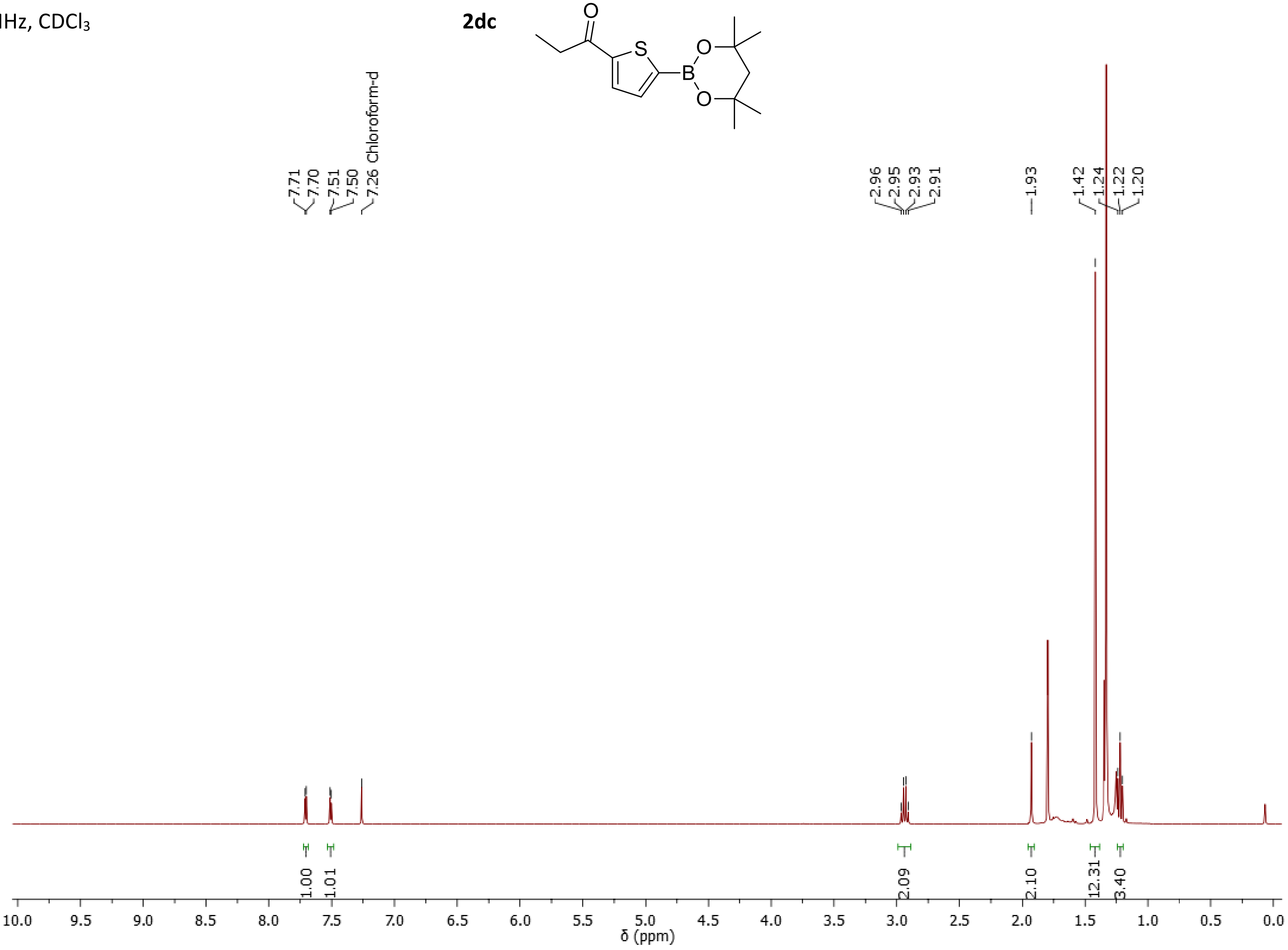

^{13}C 101MHz

2dc

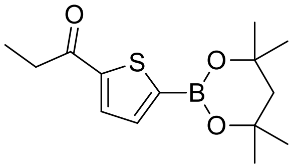

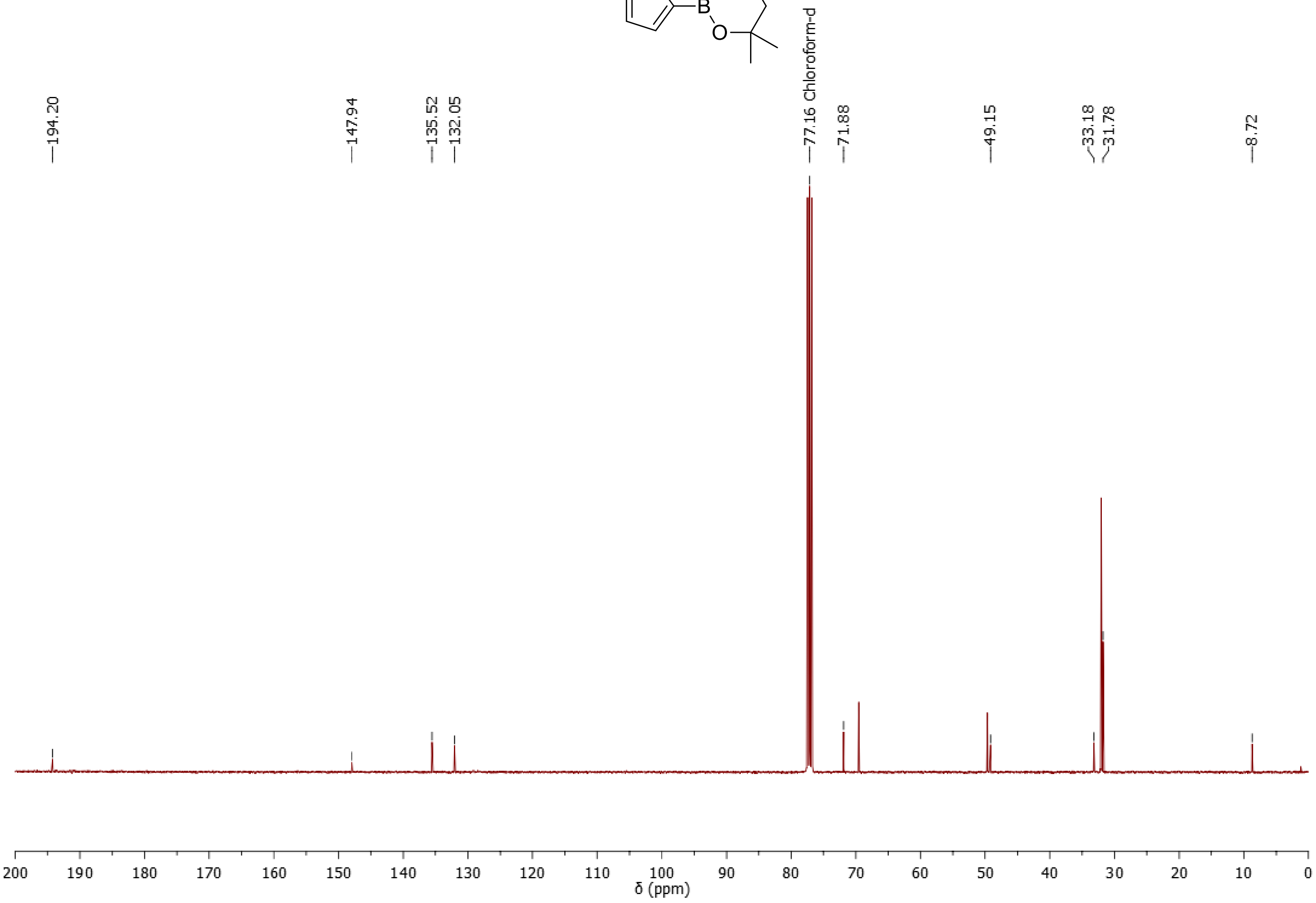

^{1}H 400MHz, $CDCl_3$

2dd

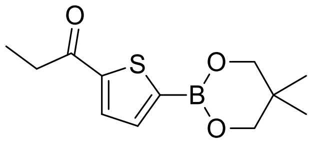

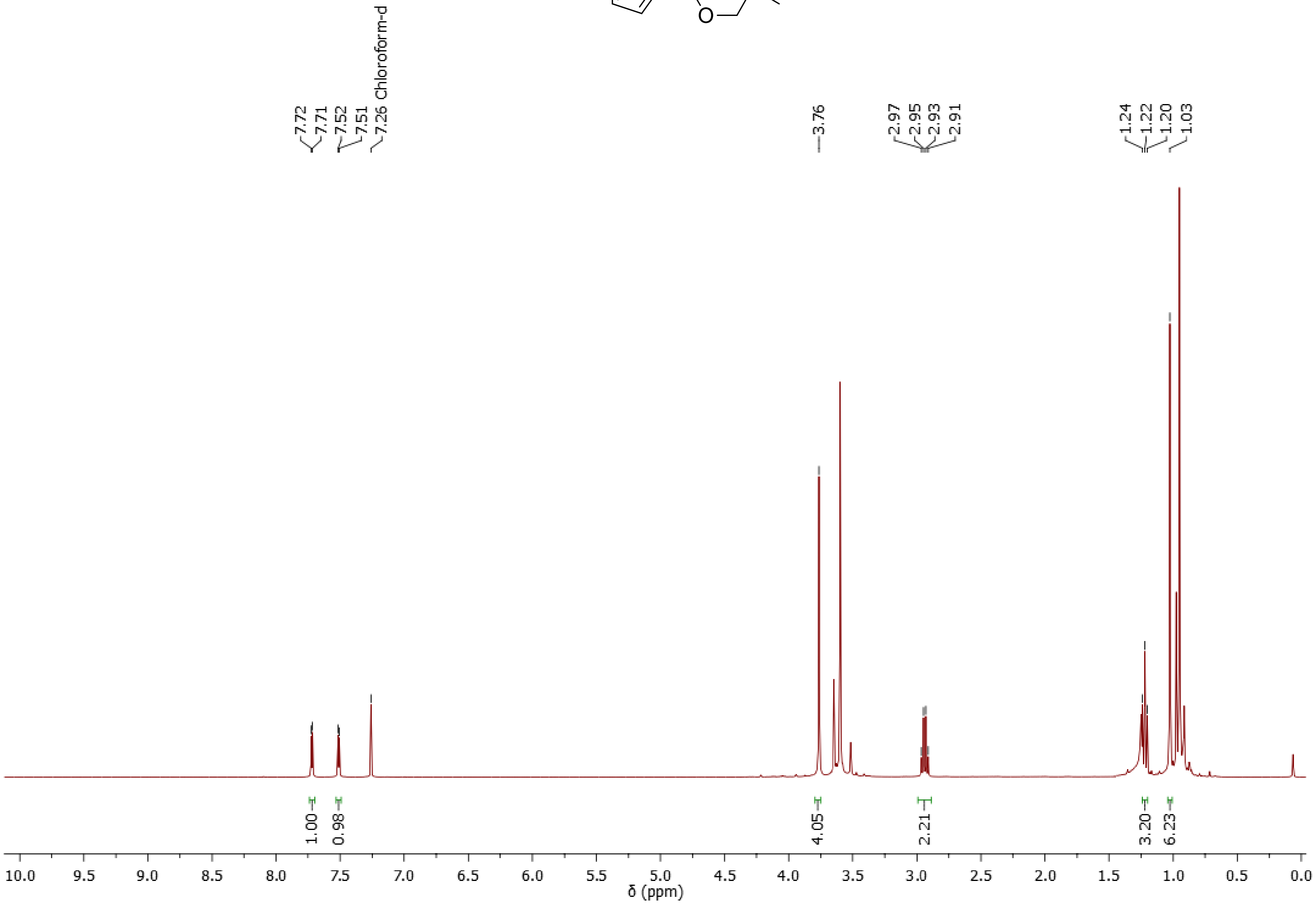

^{13}C 101MHz

2dd

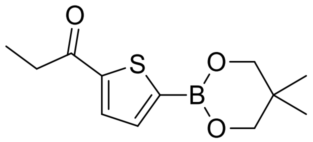

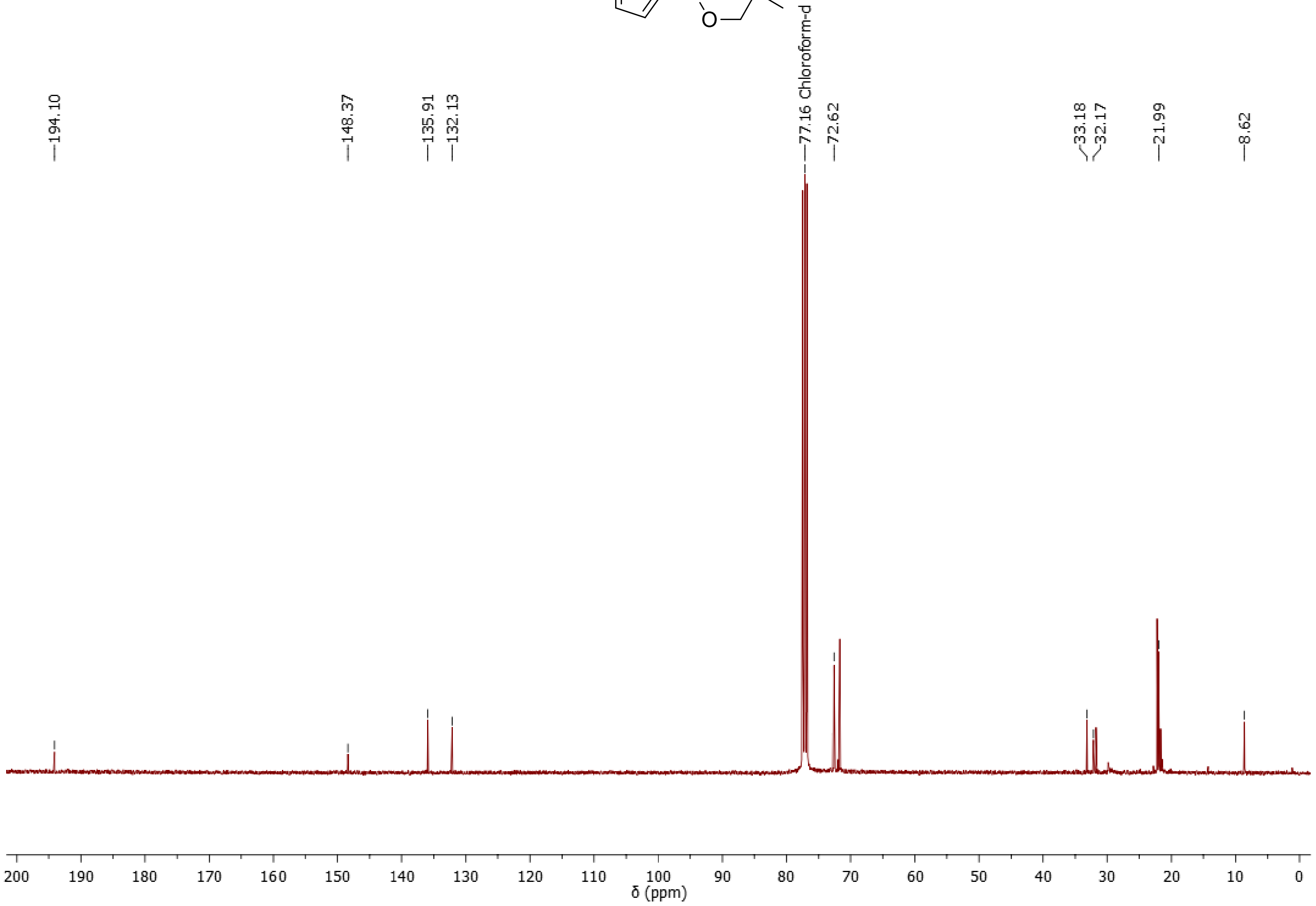

^{1}H 400MHz, $CDCl_3$

2ea

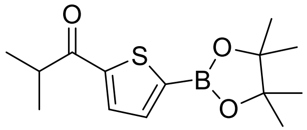

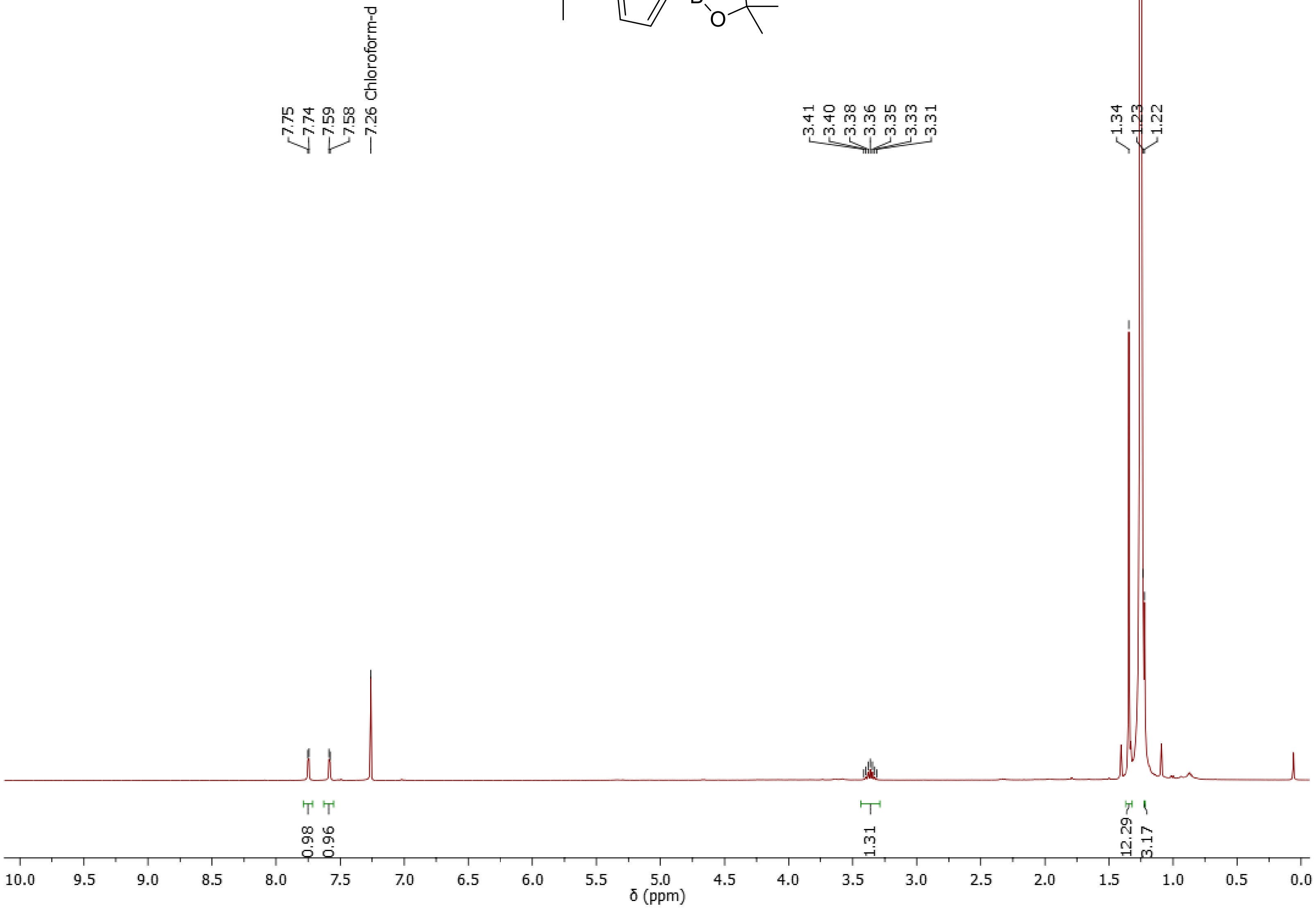

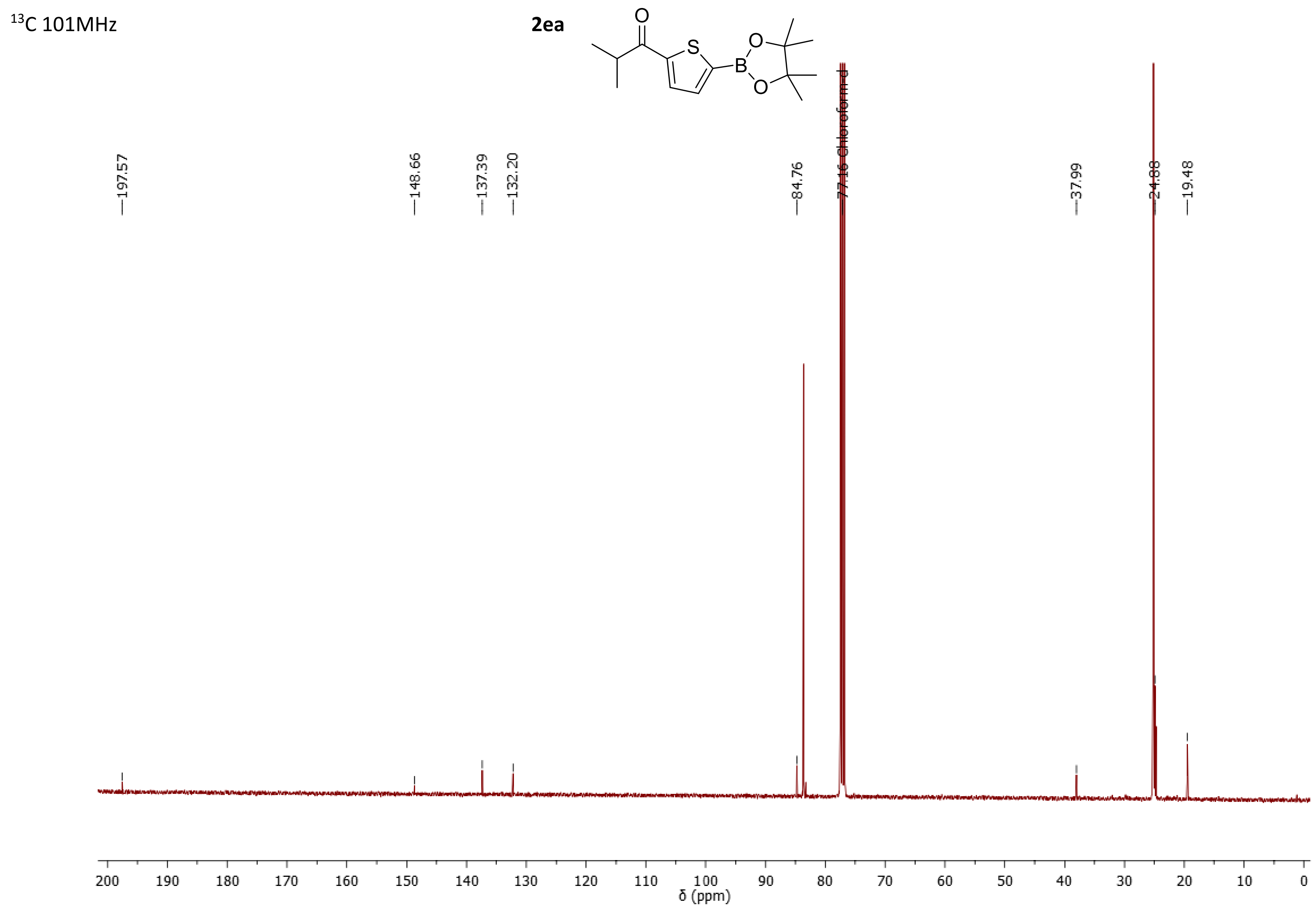
^{13}C 101MHz
2ea
197.57
148.66
137.39
132.20
84.76
77.16 Chloroform-d
37.99
24.88
19.48
δ (ppm)

^{1}H 400MHz, $CDCl_3$

2eb

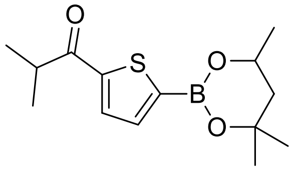

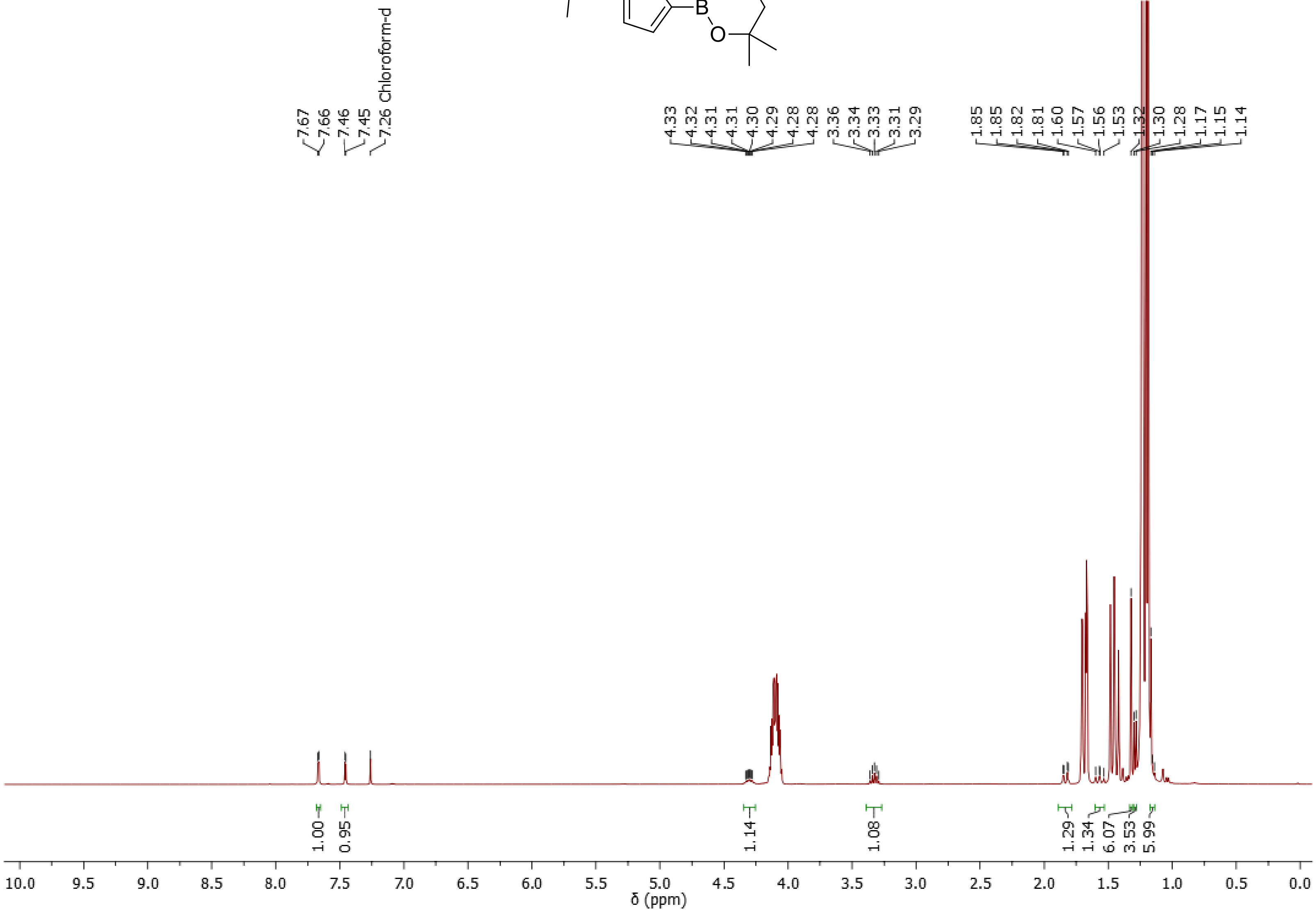

^{13}C 101MHz

2eb

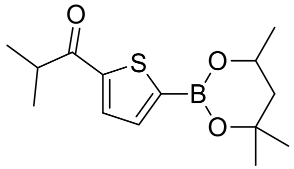

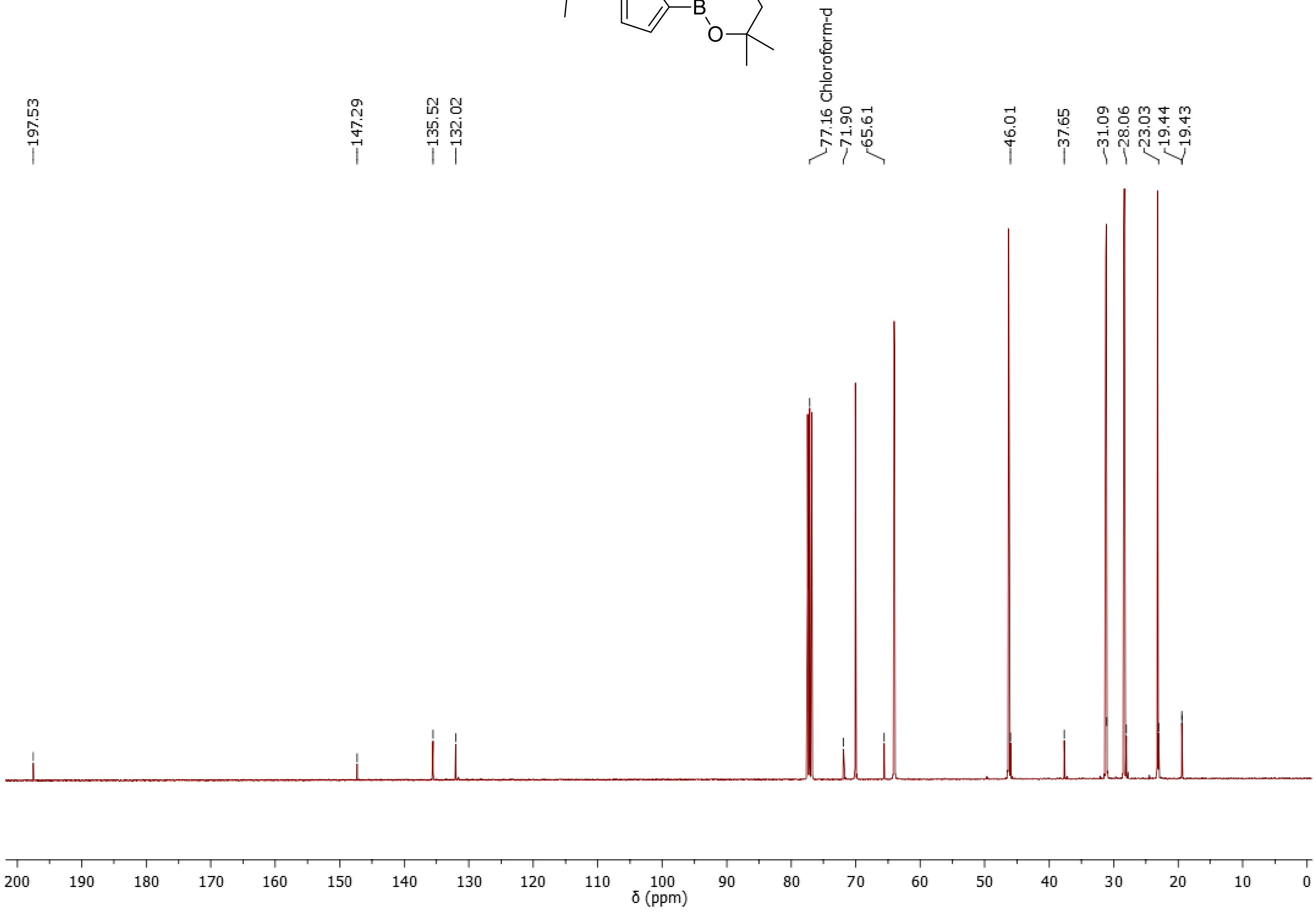

^{1}H 400MHz, $CDCl_3$

2ec

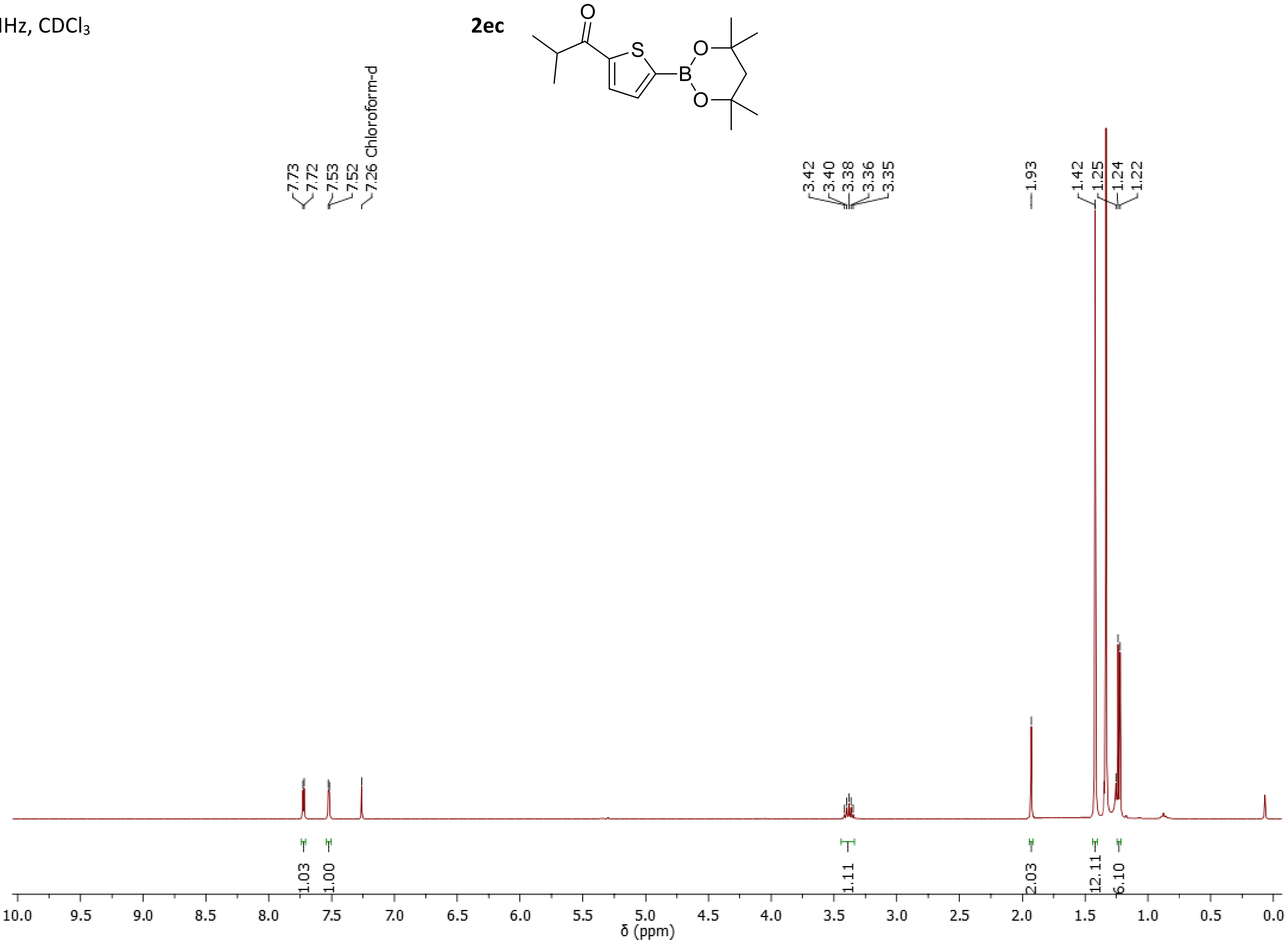

^{13}C 101MHz

2ec

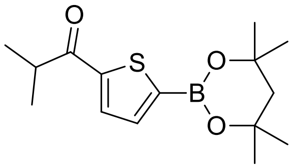

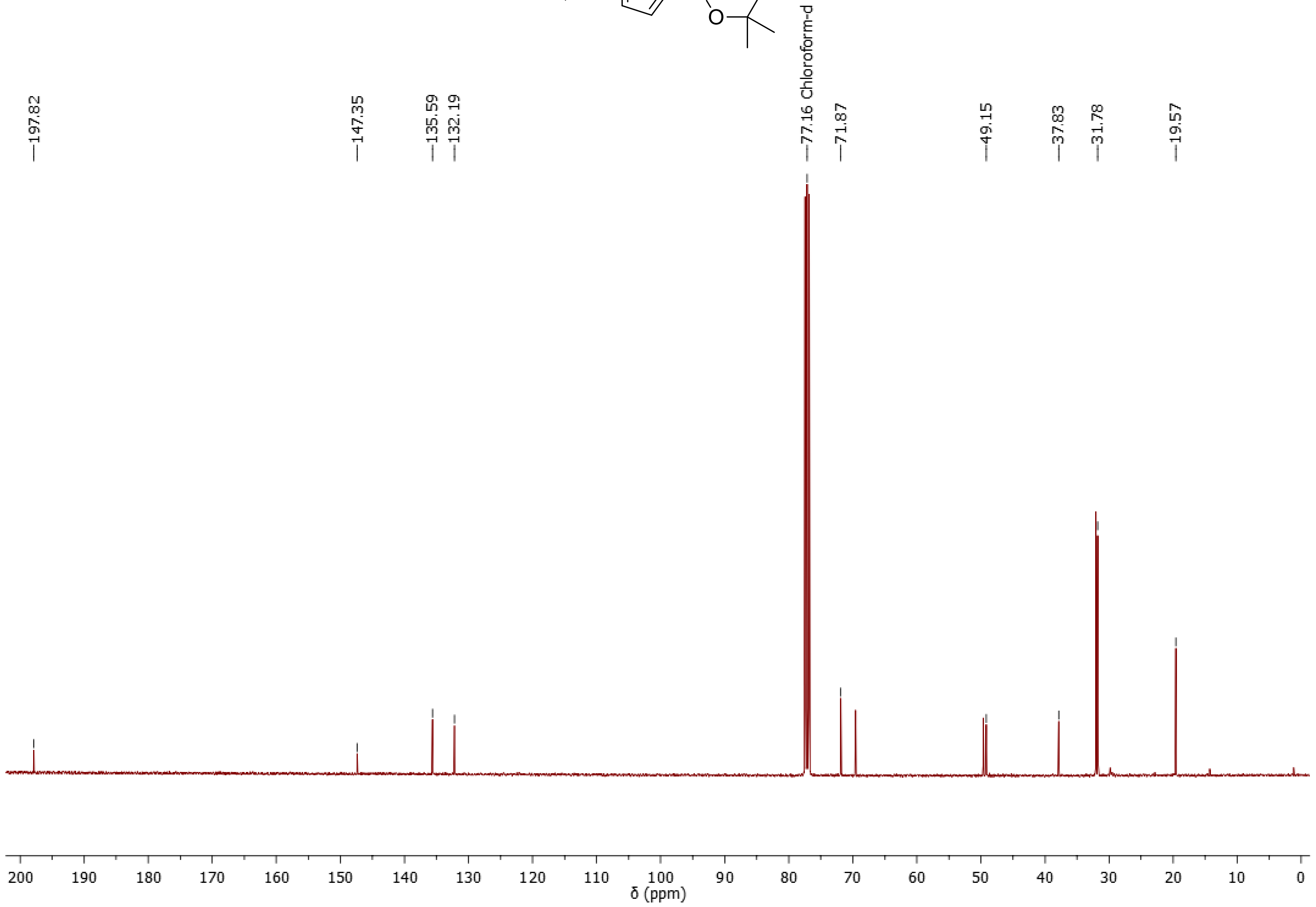

^{1}H 400MHz, $CDCl_3$

2ed

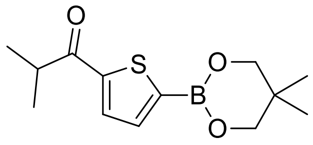

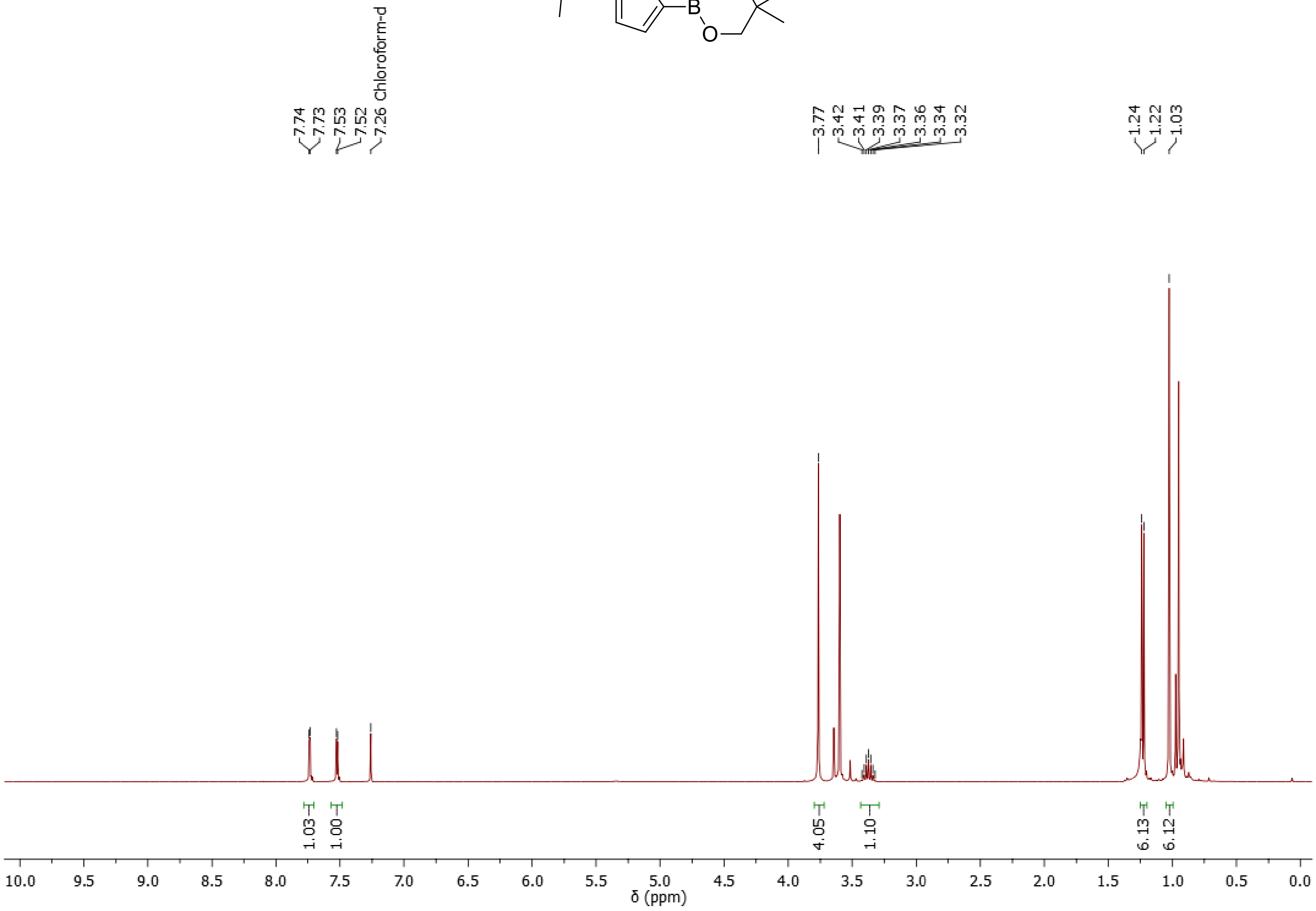

^{13}C 101MHz

2ed

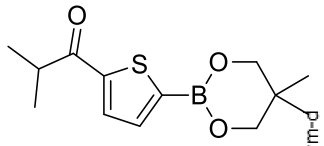

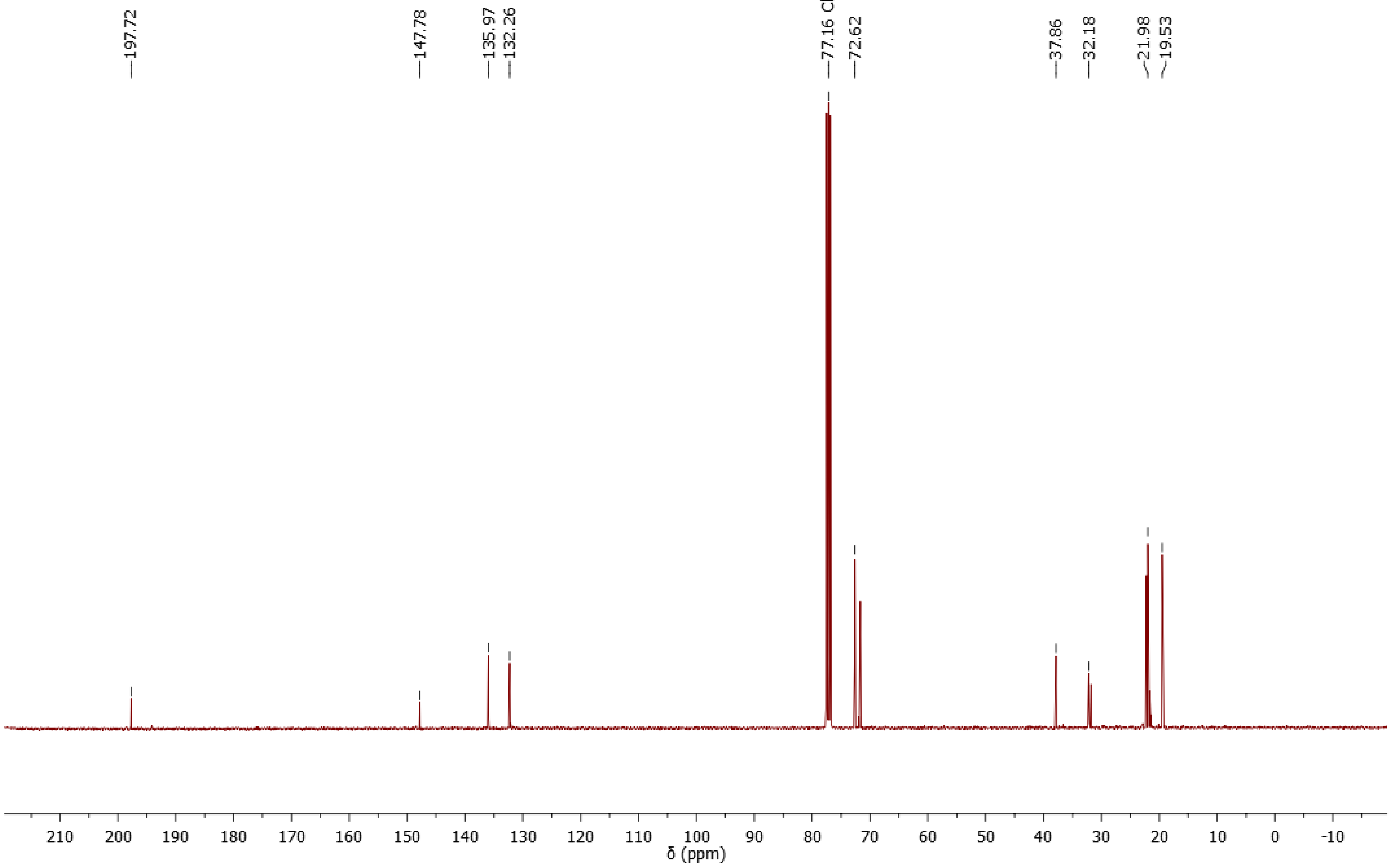

^1H 400MHz, $CDCl_3$

2fa

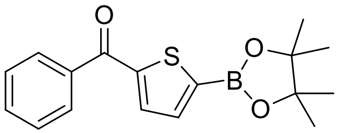

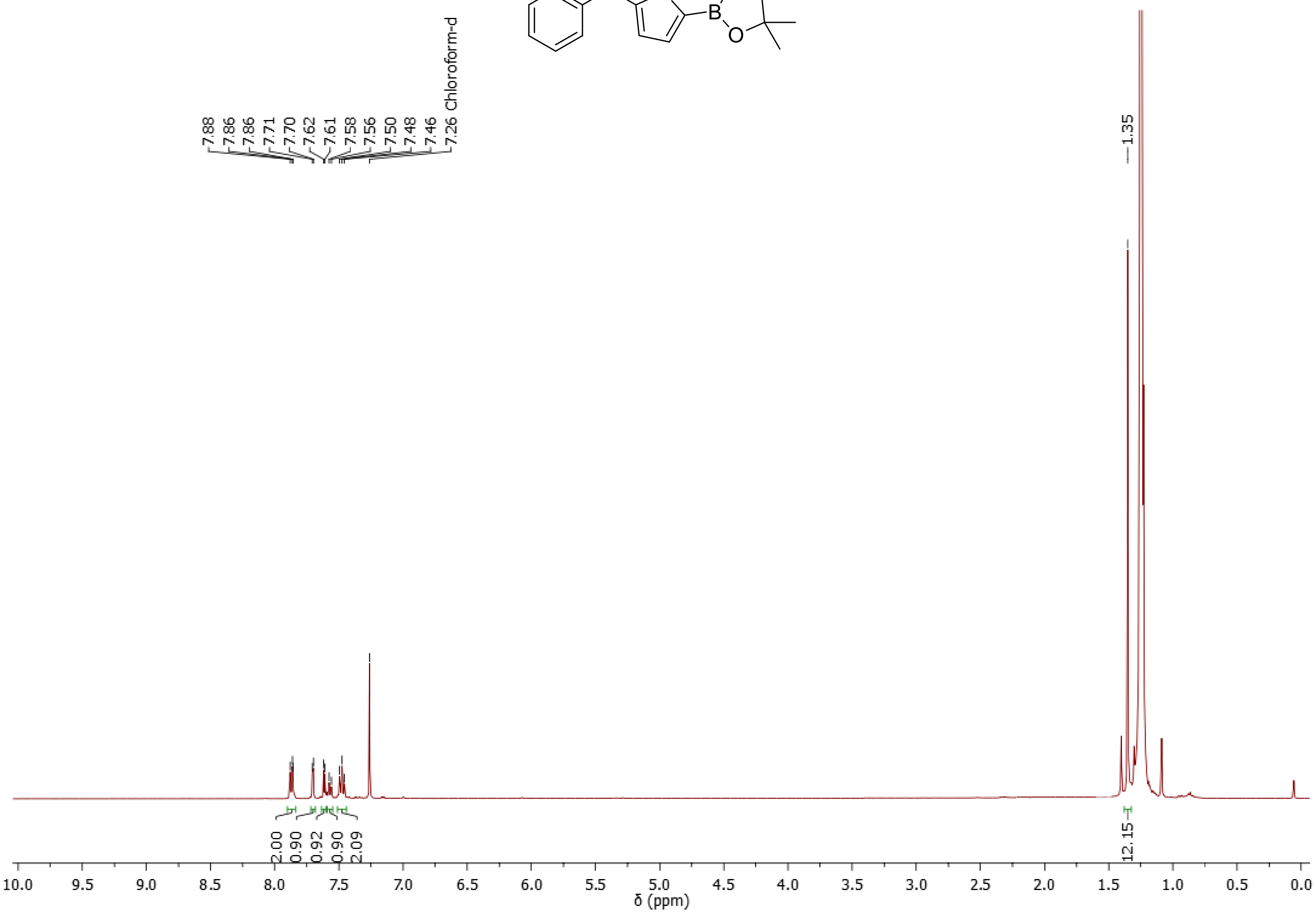

^{13}C 101MHz

2fa

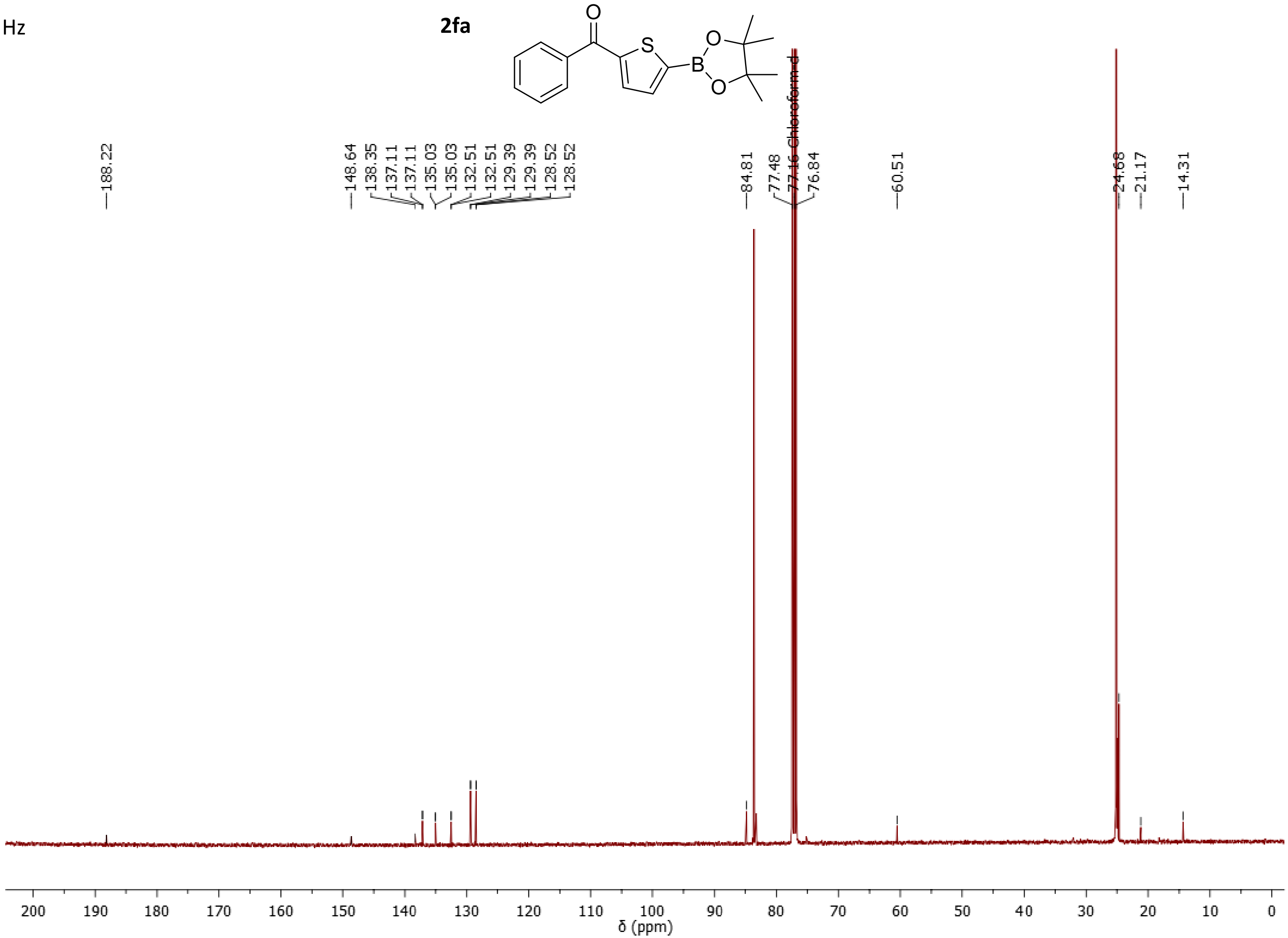

^{1}H 400MHz, $CDCl_3$

2fb

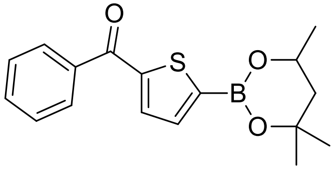

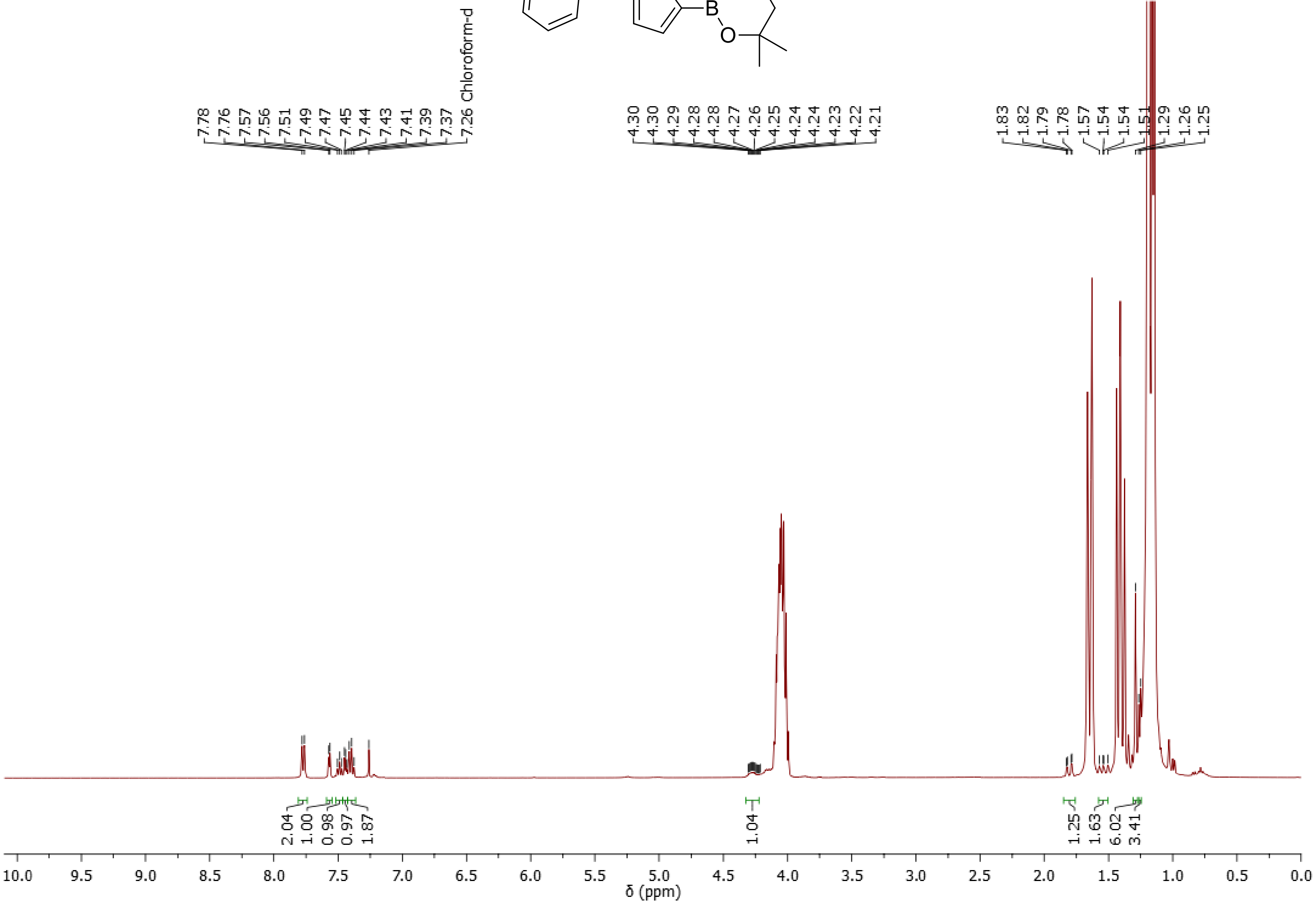

^{13}C 101MHz

2fb

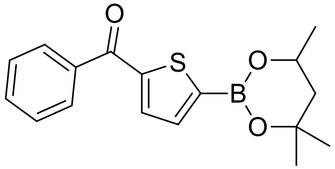

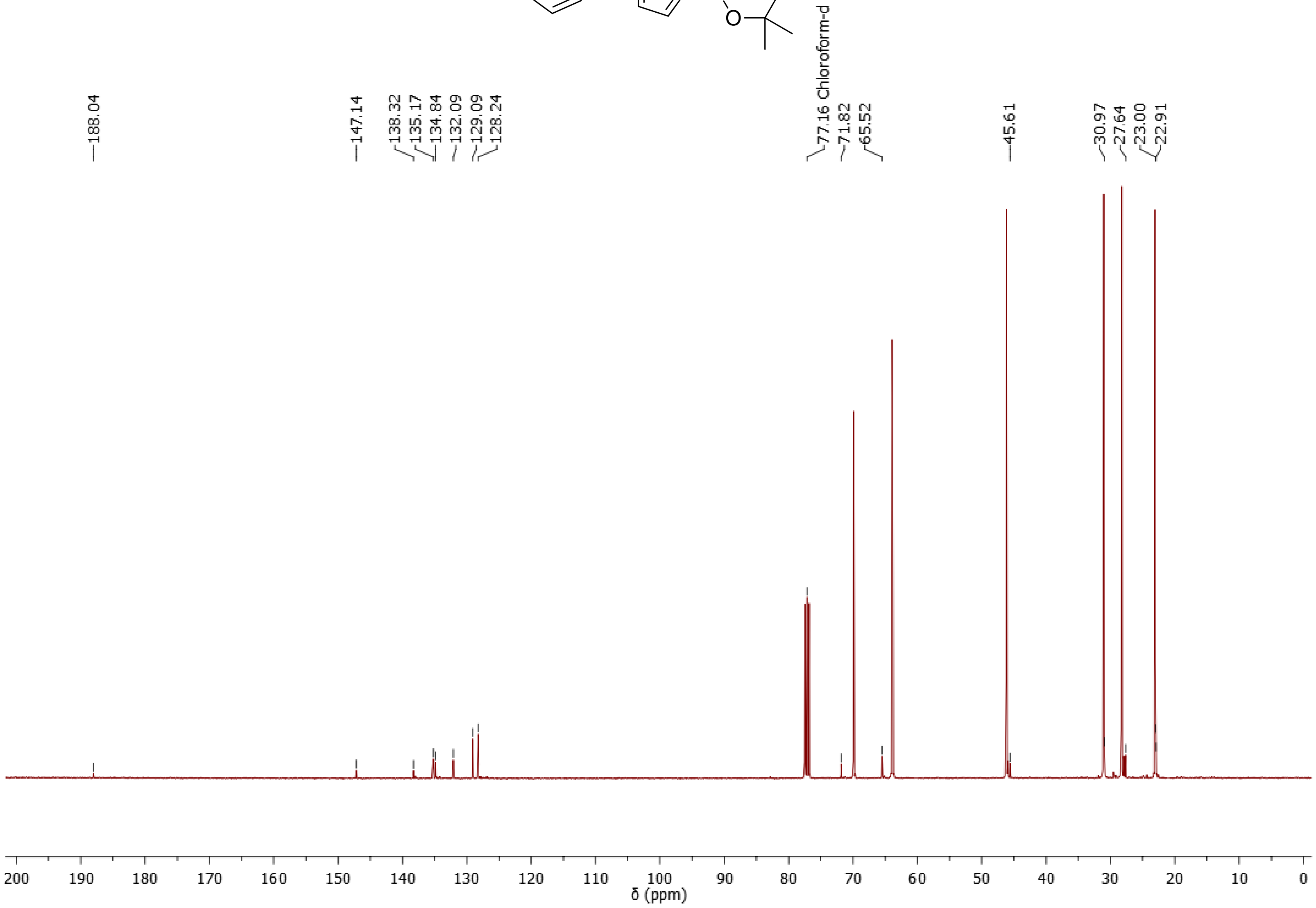

^{1}H 400MHz, $CDCl_3$

2fc

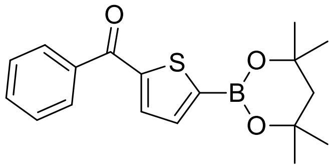

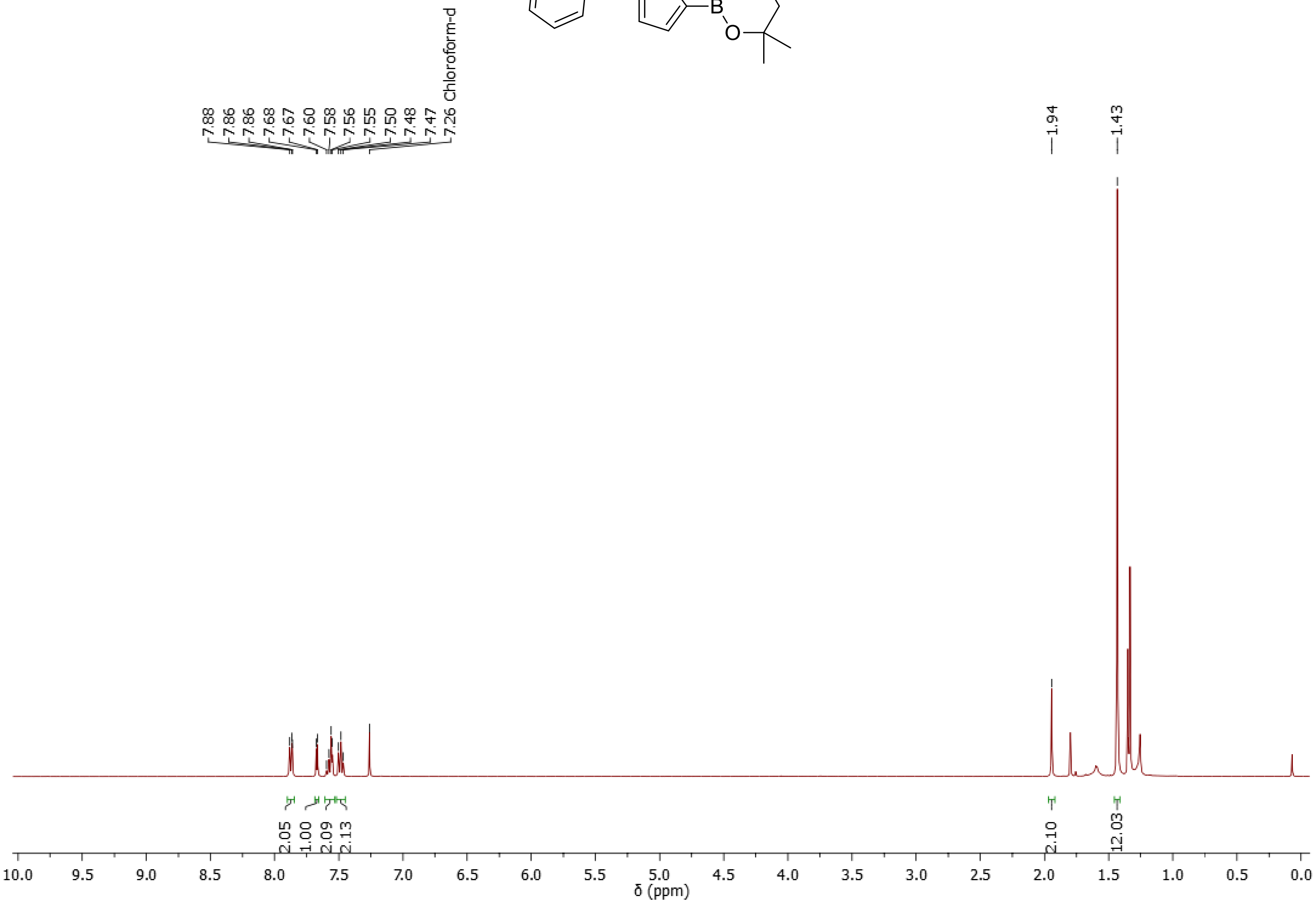

^{13}C 101MHz

2fc

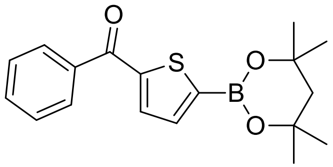

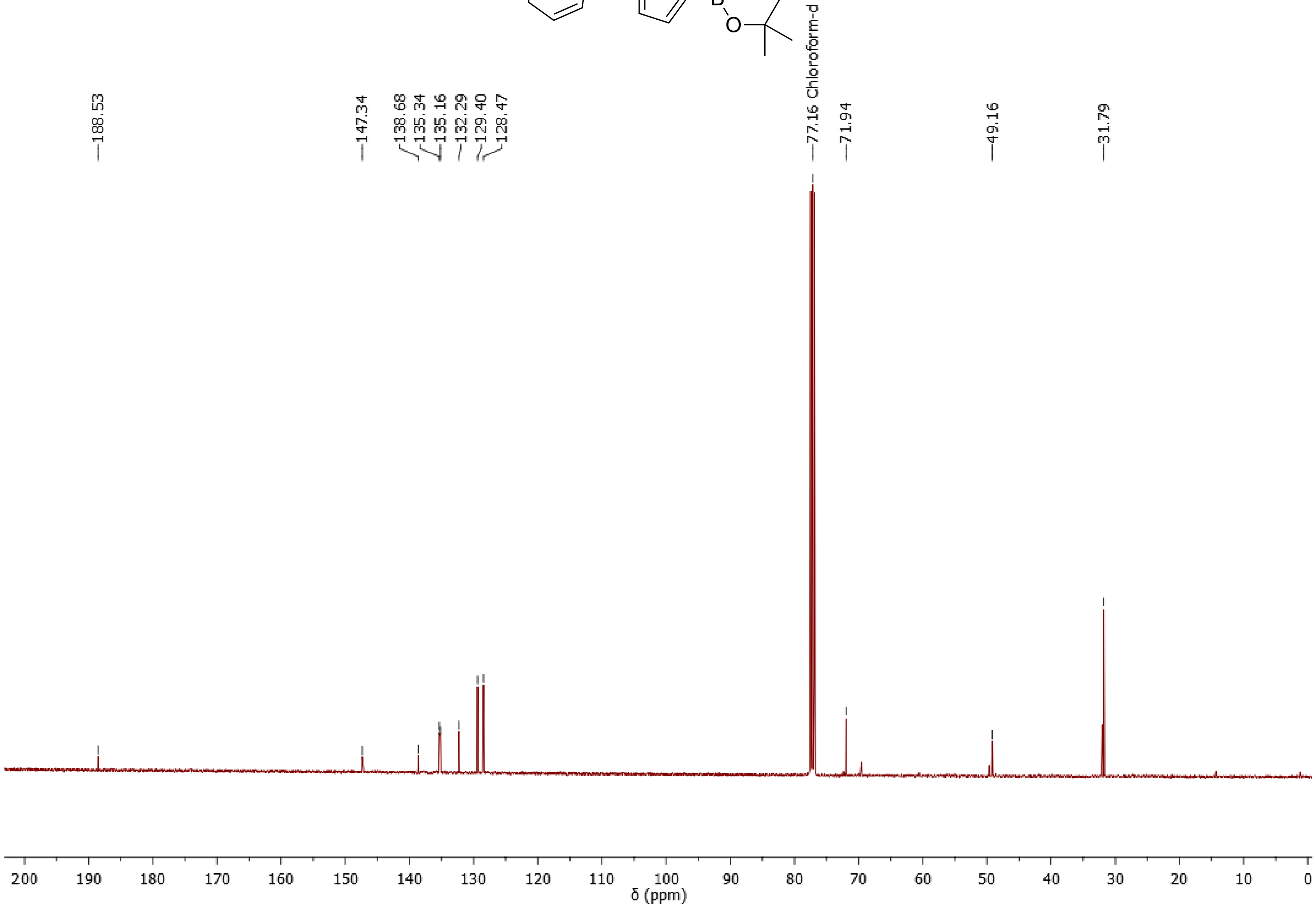

^{1}H 400MHz, $CDCl_3$

2fd

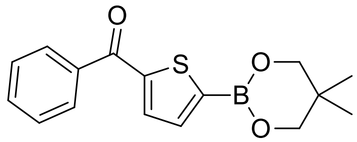

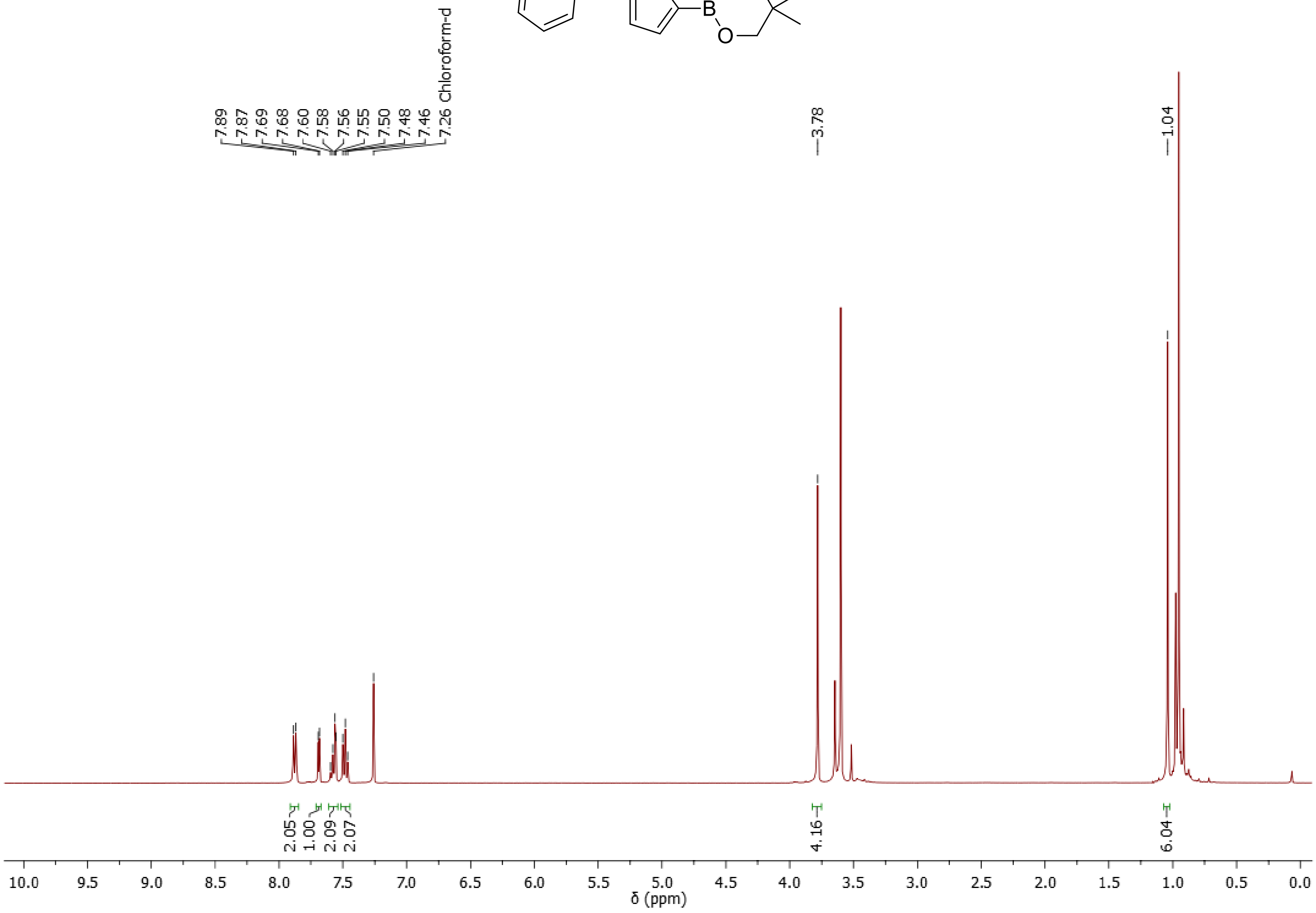

^{13}C 101MHz

2fd

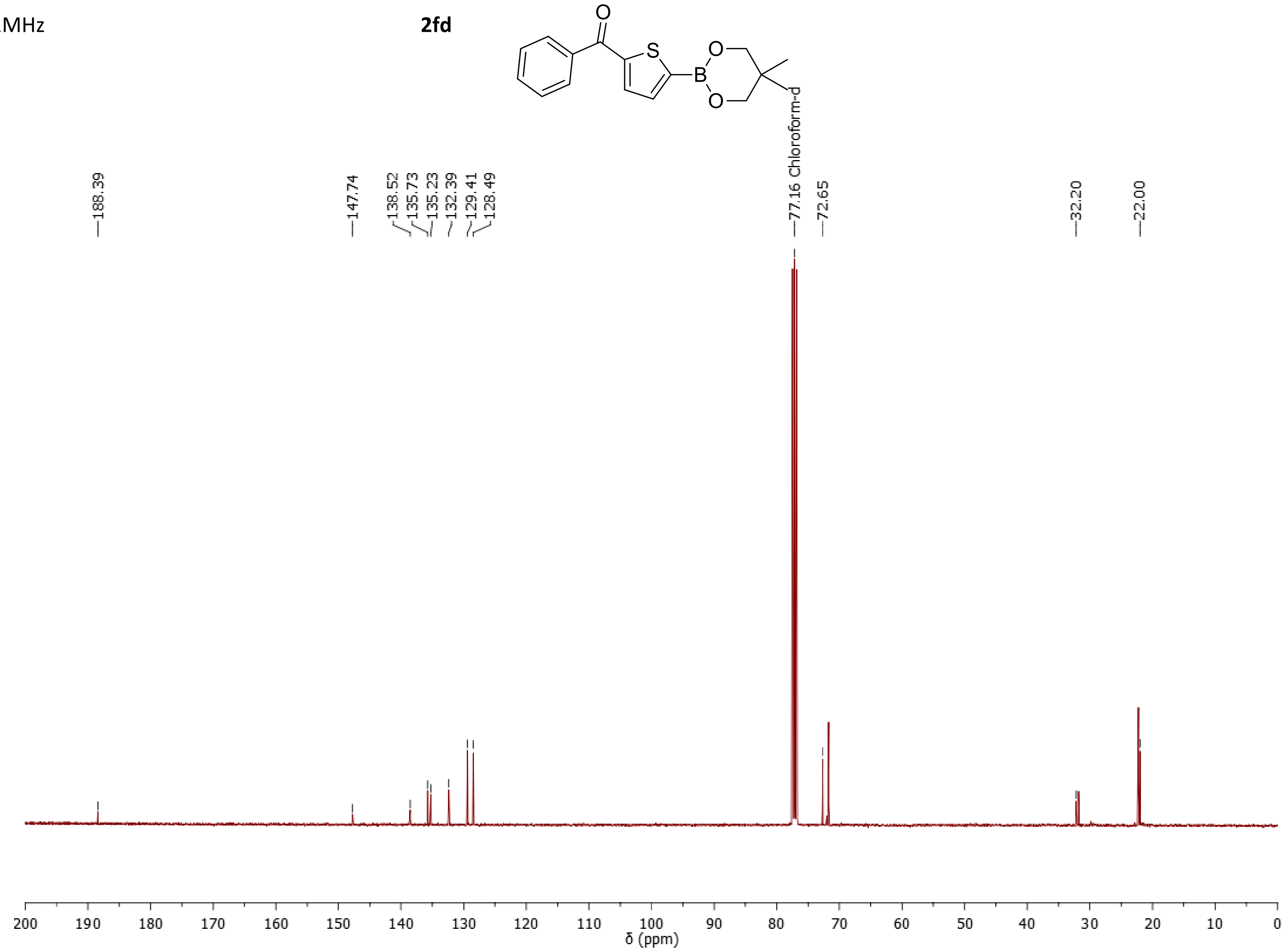

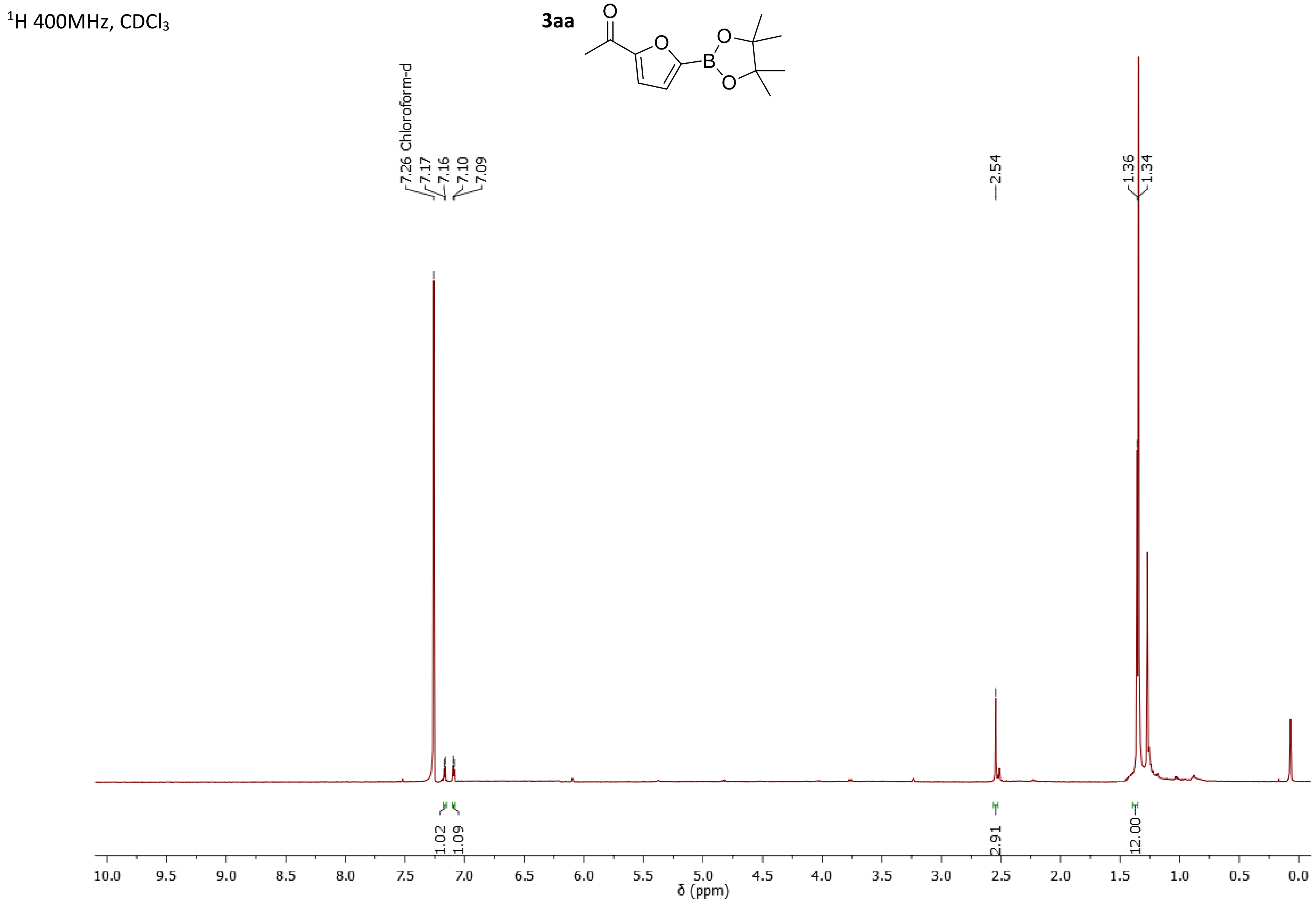

1H 400MHz, CDCl3
3aa
O
O
B
O
O
7.26 Chloroform-d
7.17
7.16
7.10
7.09
2.54
1.36
1.34
1.02
1.09
2.91
12.00
10.0
9.5
9.0
8.5
8.0
7.5
7.0
6.5
6.0
5.5
5.0
4.5
4.0
3.5
3.0
2.5
2.0
1.5
1.0
0.5
0.0
δ (ppm)

^{13}C 101MHz, $CDCl_3$

3aa

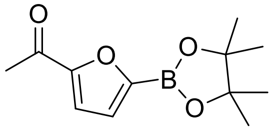

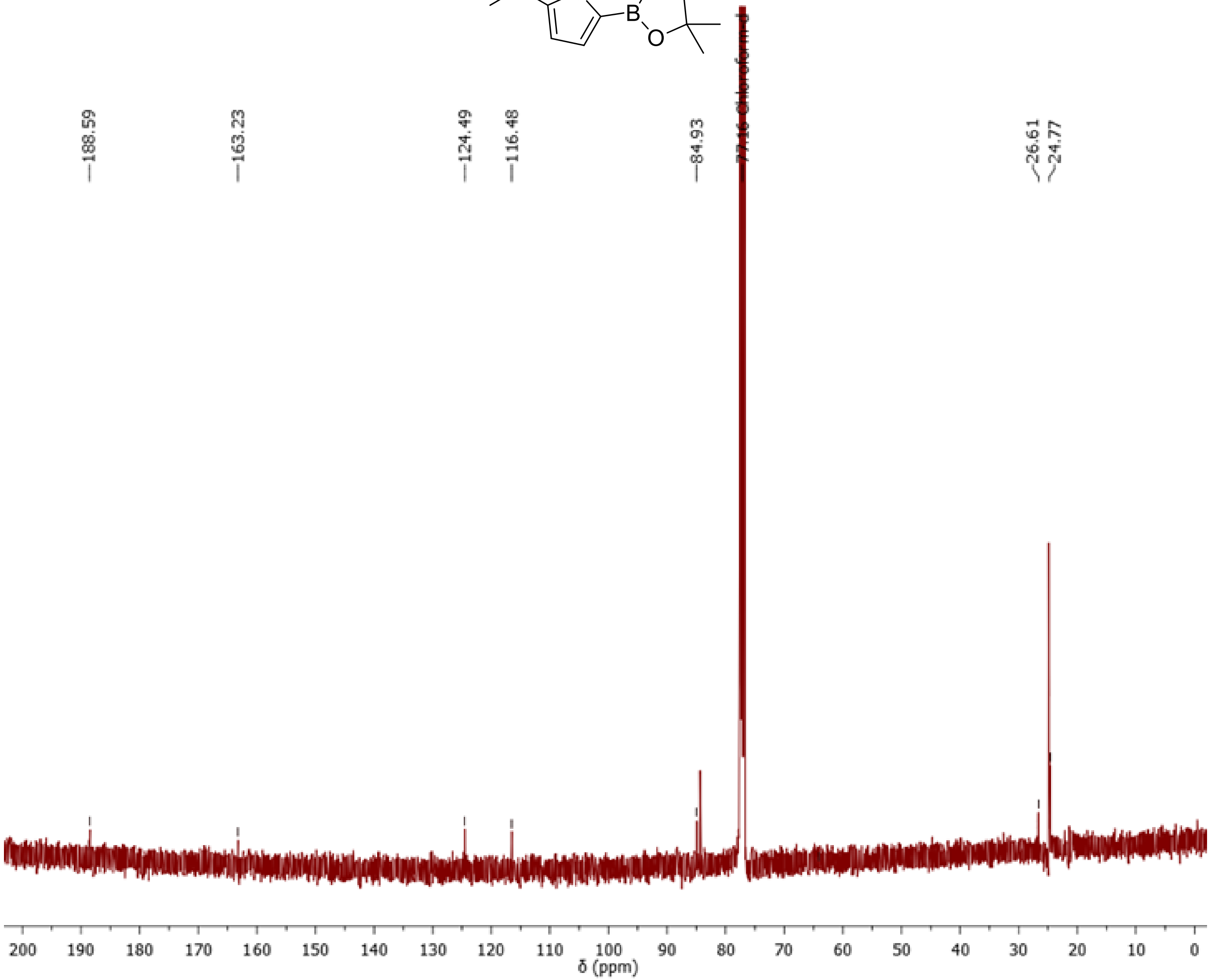

^{1}H 400MHz, $CDCl_3$

3ab

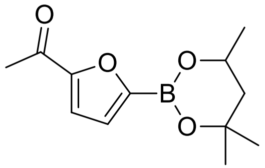

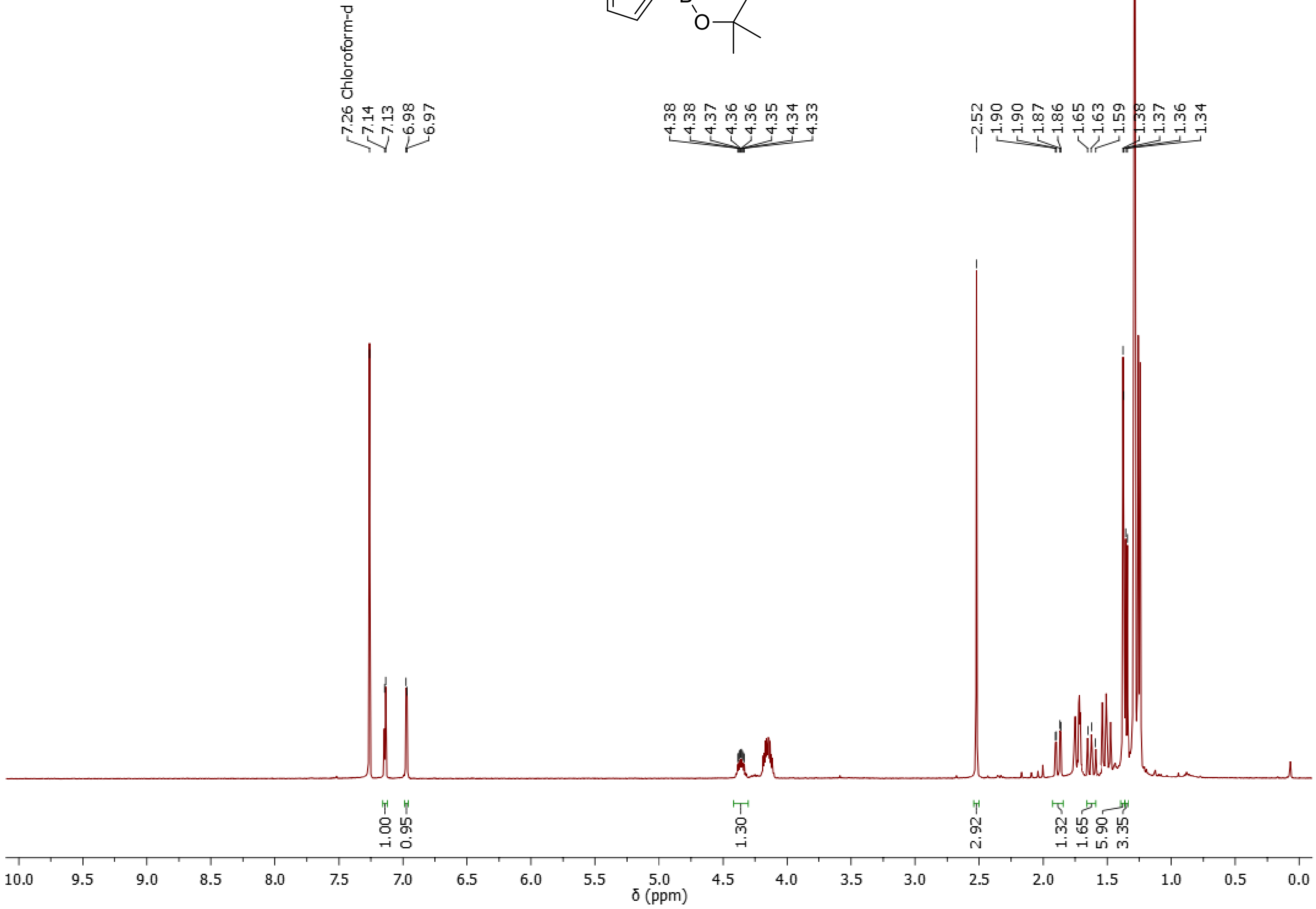

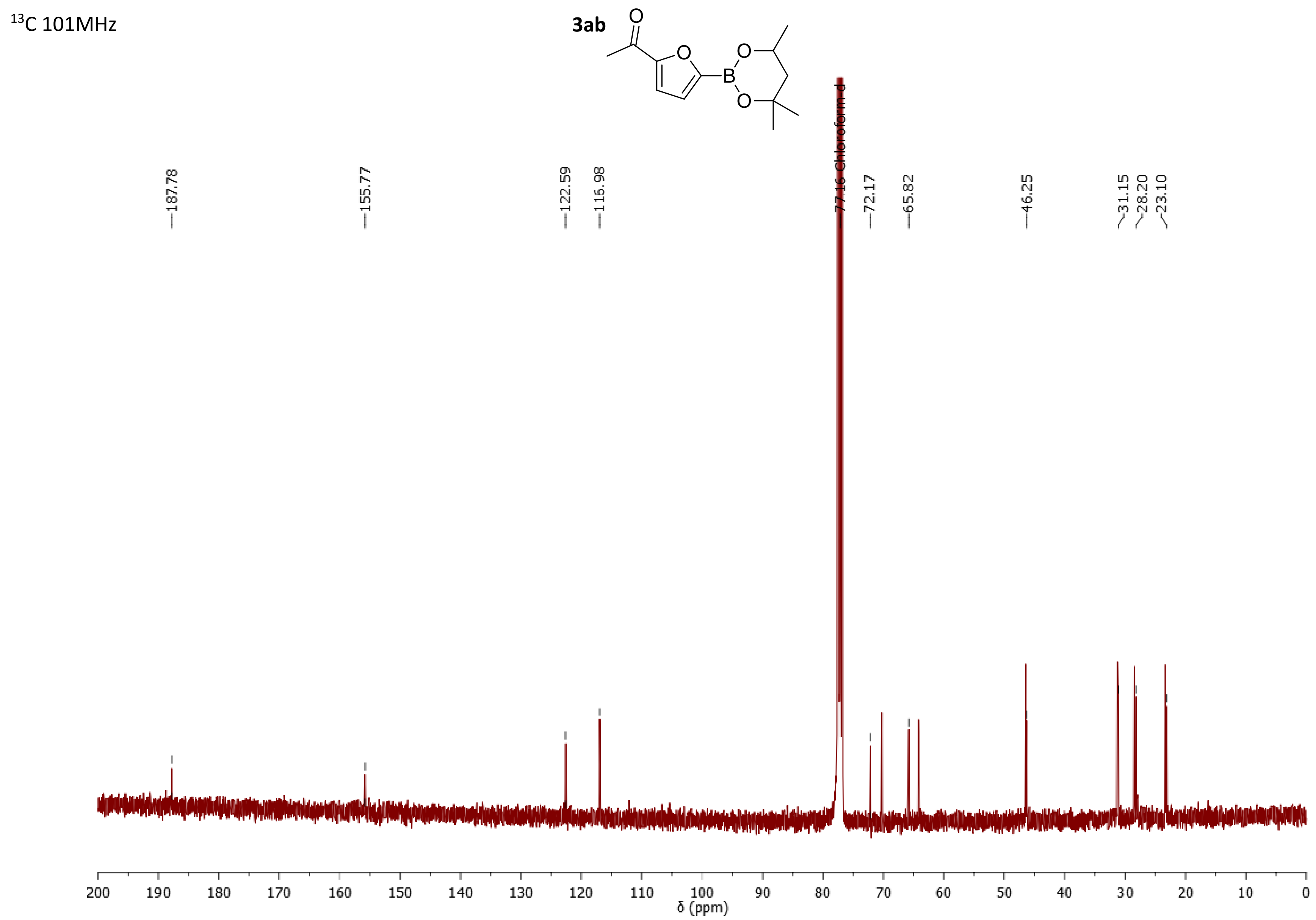

13C 101MHz
3ab
187.78
155.77
122.59
116.98
77.16 Chloroform-d
72.17
65.82
46.25
31.15
28.20
23.10
200 190 180 170 160 150 140 130 120 110 100 90 80 70 60 50 40 30 20 10 0
δ (ppm)

^1H 400MHz, $CDCl_3$

3ac

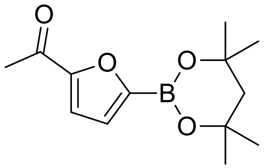

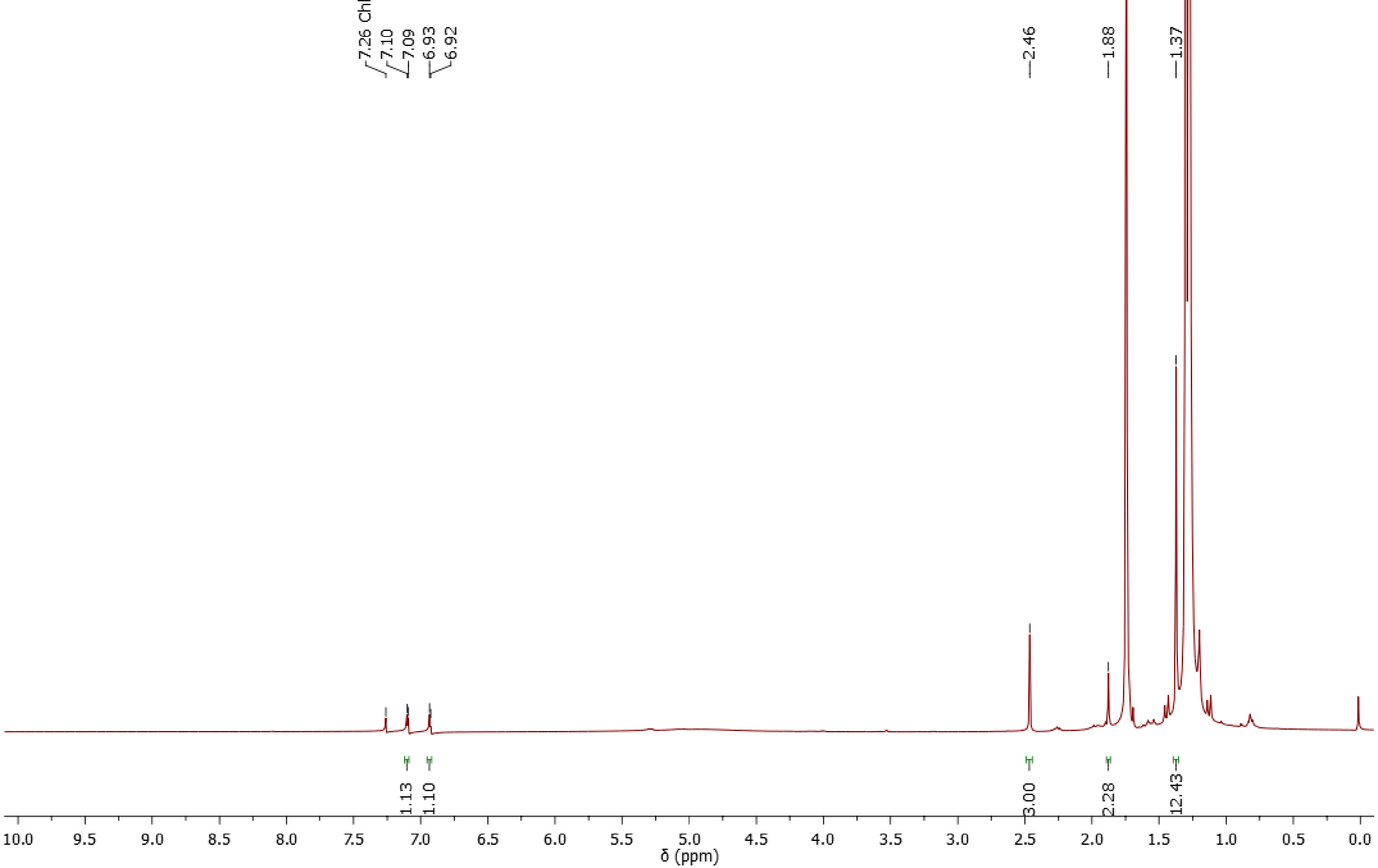

^{13}C 101MHz

3ac

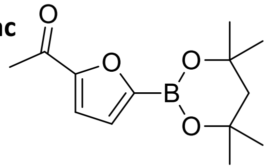

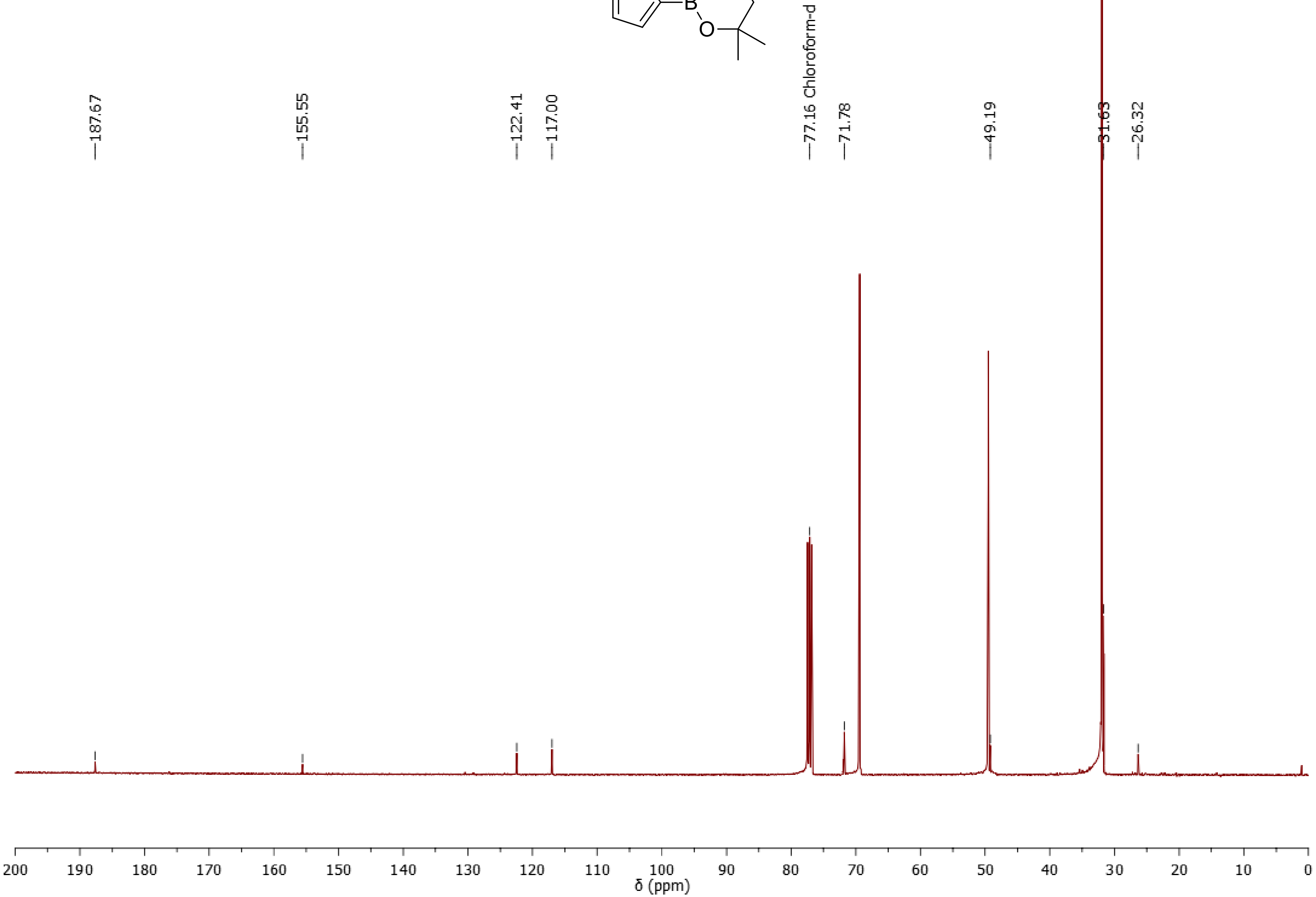

^{1}H 400MHz, $CDCl_3$

3ad

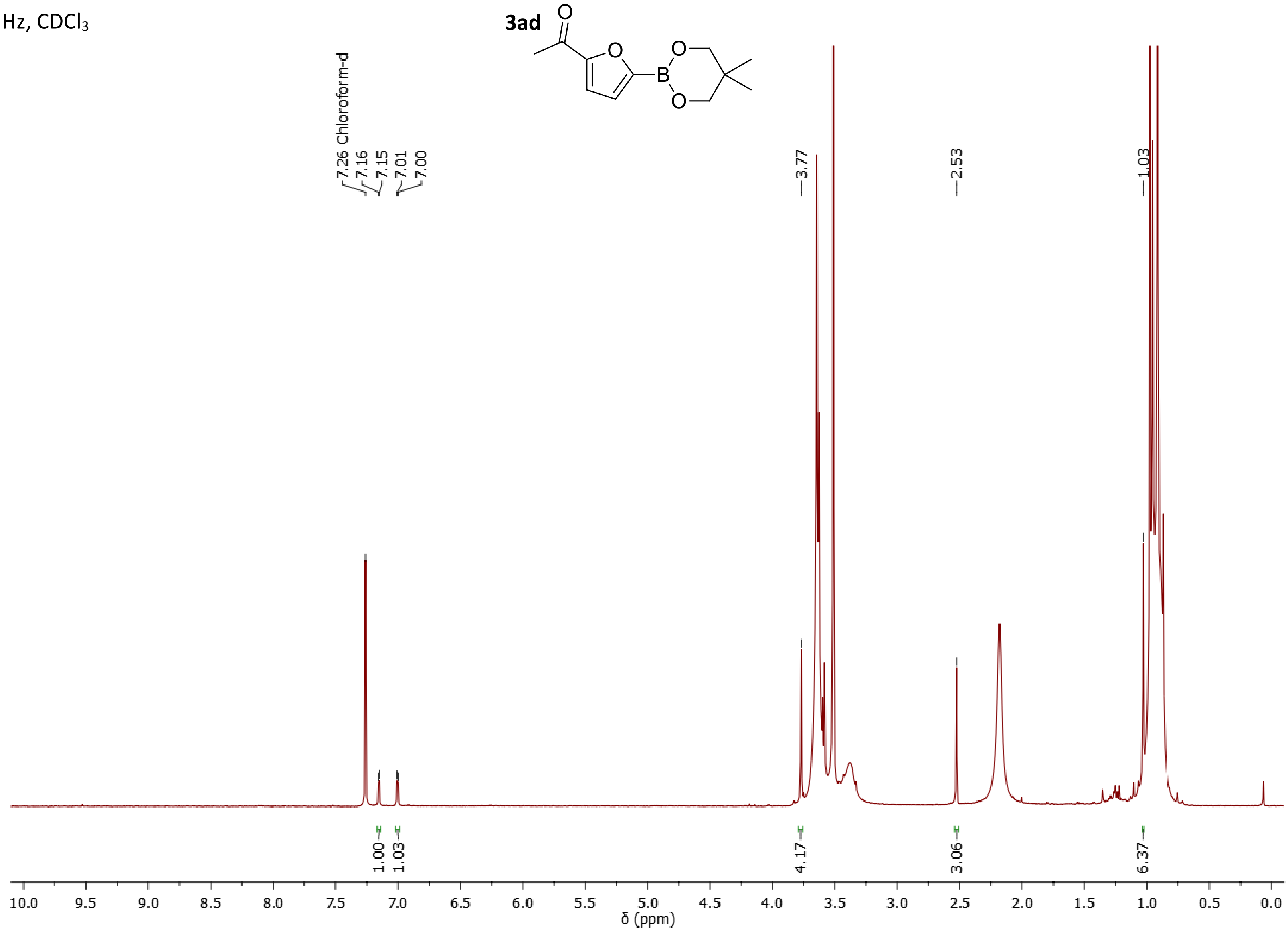

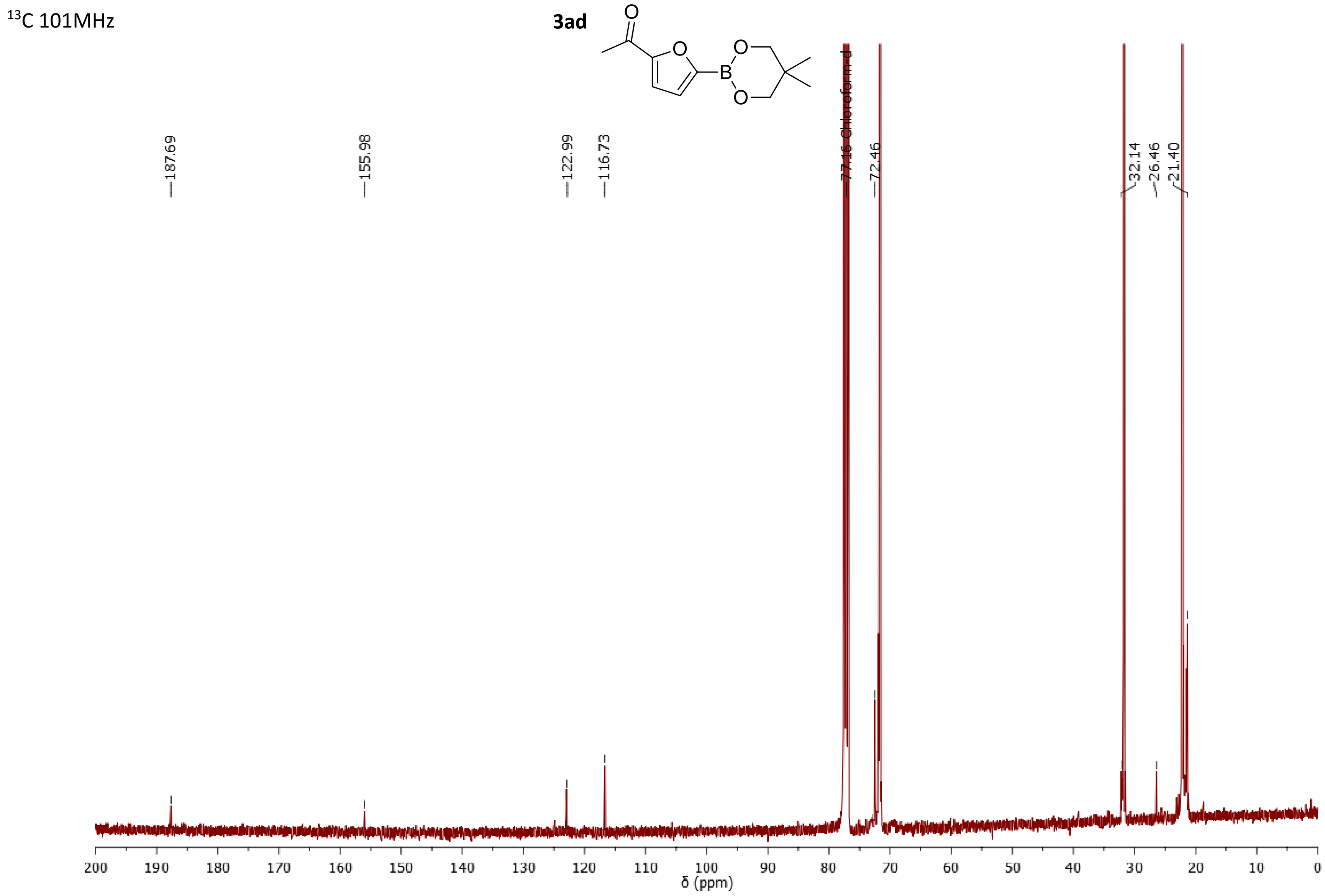
13C 101MHz
3ad
O
O
B
O
O
187.69
155.98
122.99
116.73
77.16 Chloroform-d
72.46
32.14
26.46
21.40
200
190
180
170
160
150
140
130
120
110
100
90
80
70
60
50
40
30
20
10
0
δ (ppm)

^{1}H 400MHz, $CDCl_3$

3ba

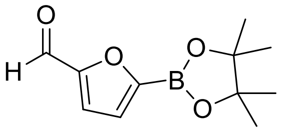

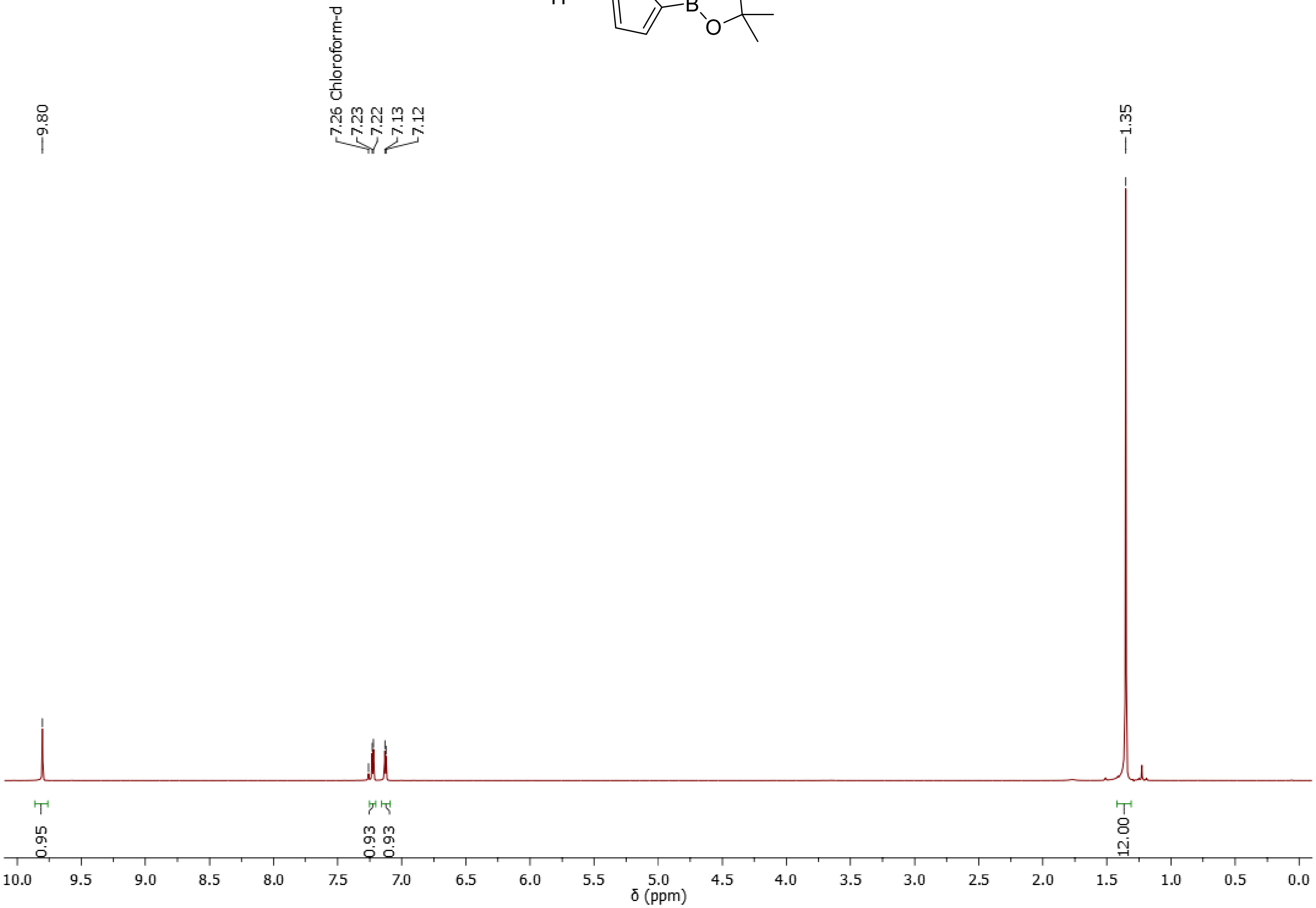

^{13}C 101MHz, $CDCl_3$

3ba

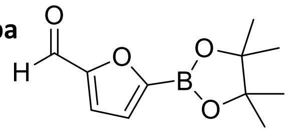

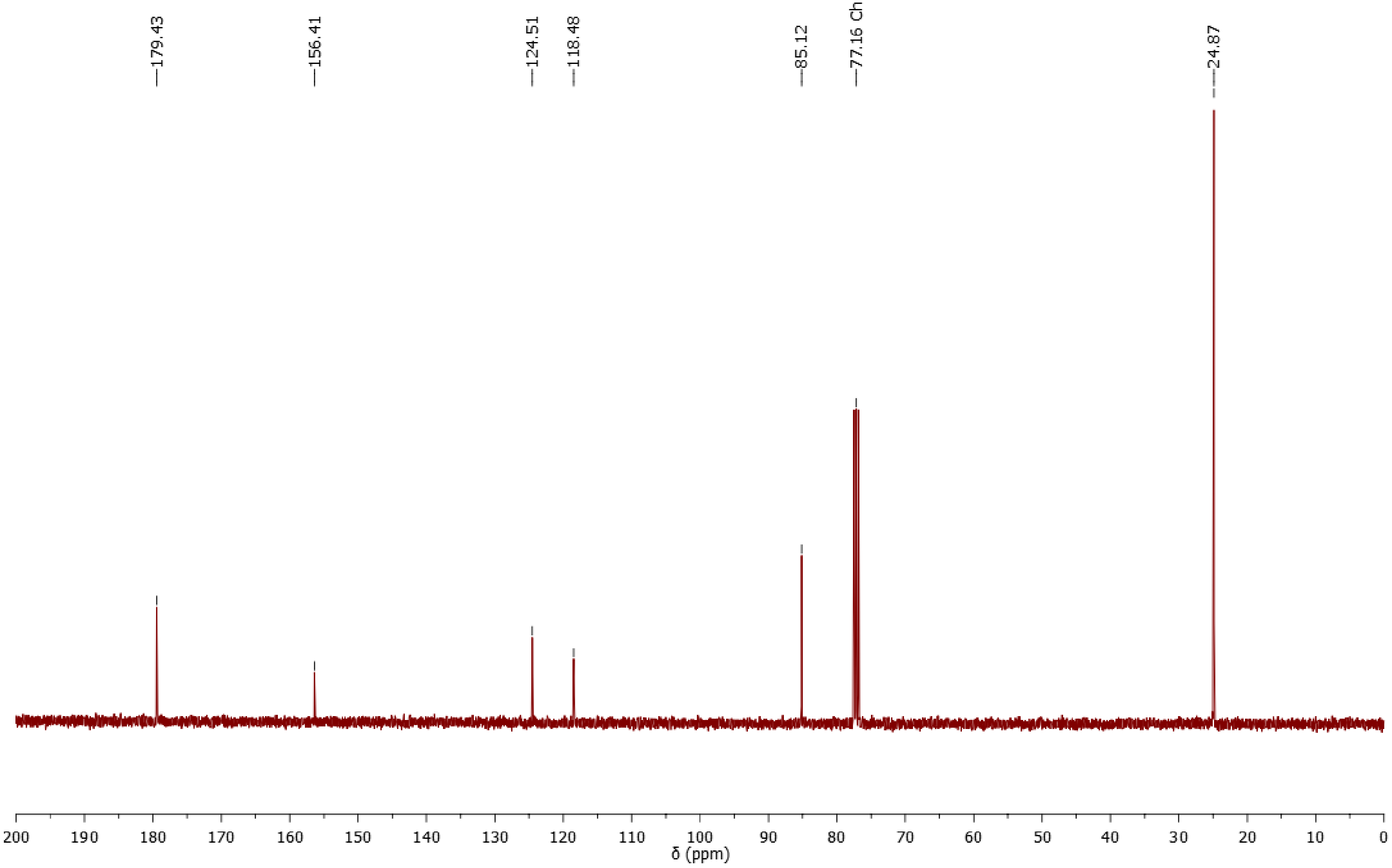

^{1}H 400MHz, $CDCl_3$

3bb

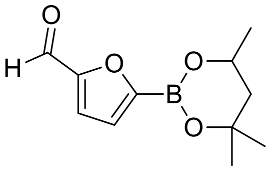

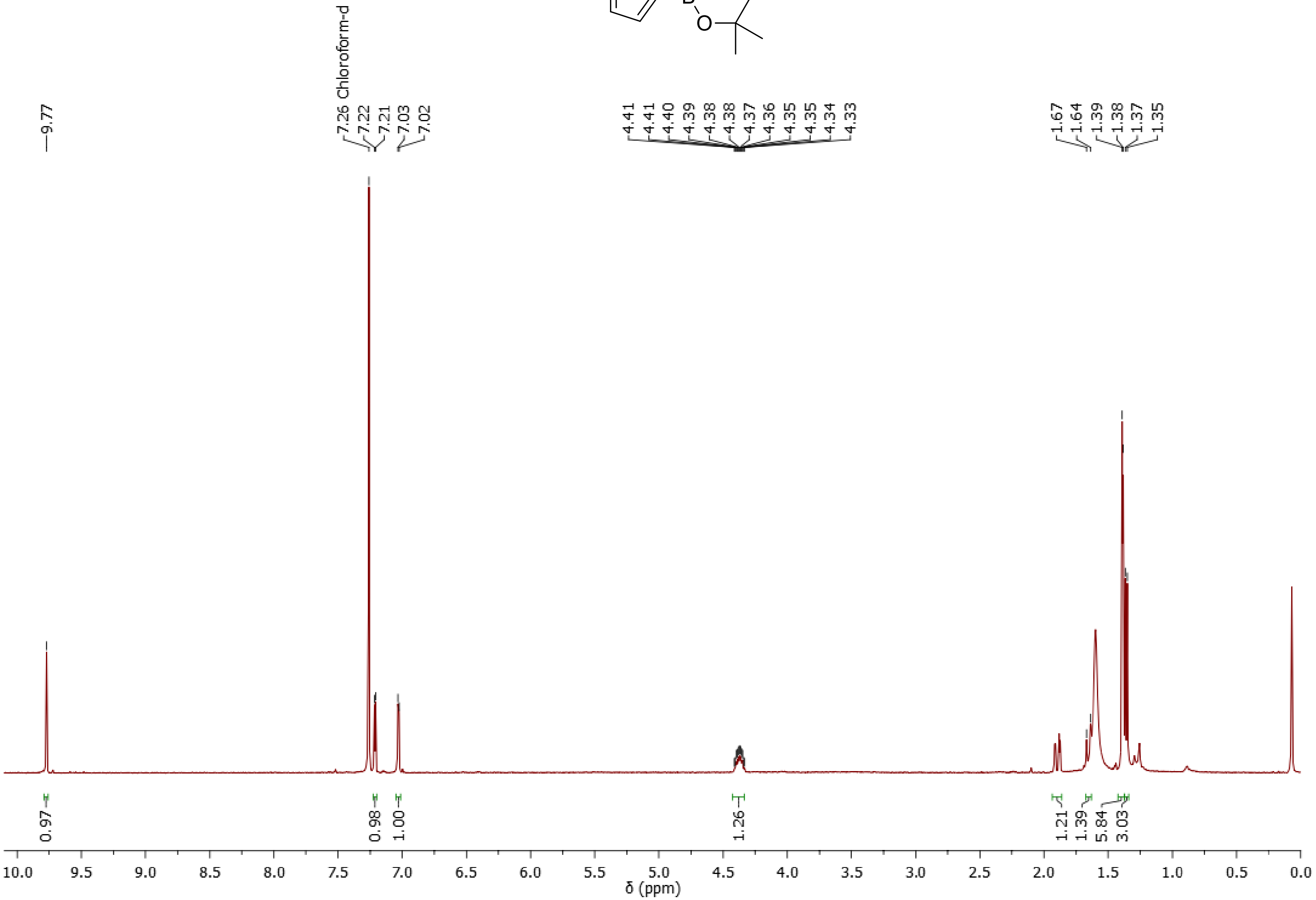

^{13}C 101MHz

3bb

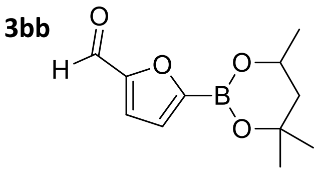

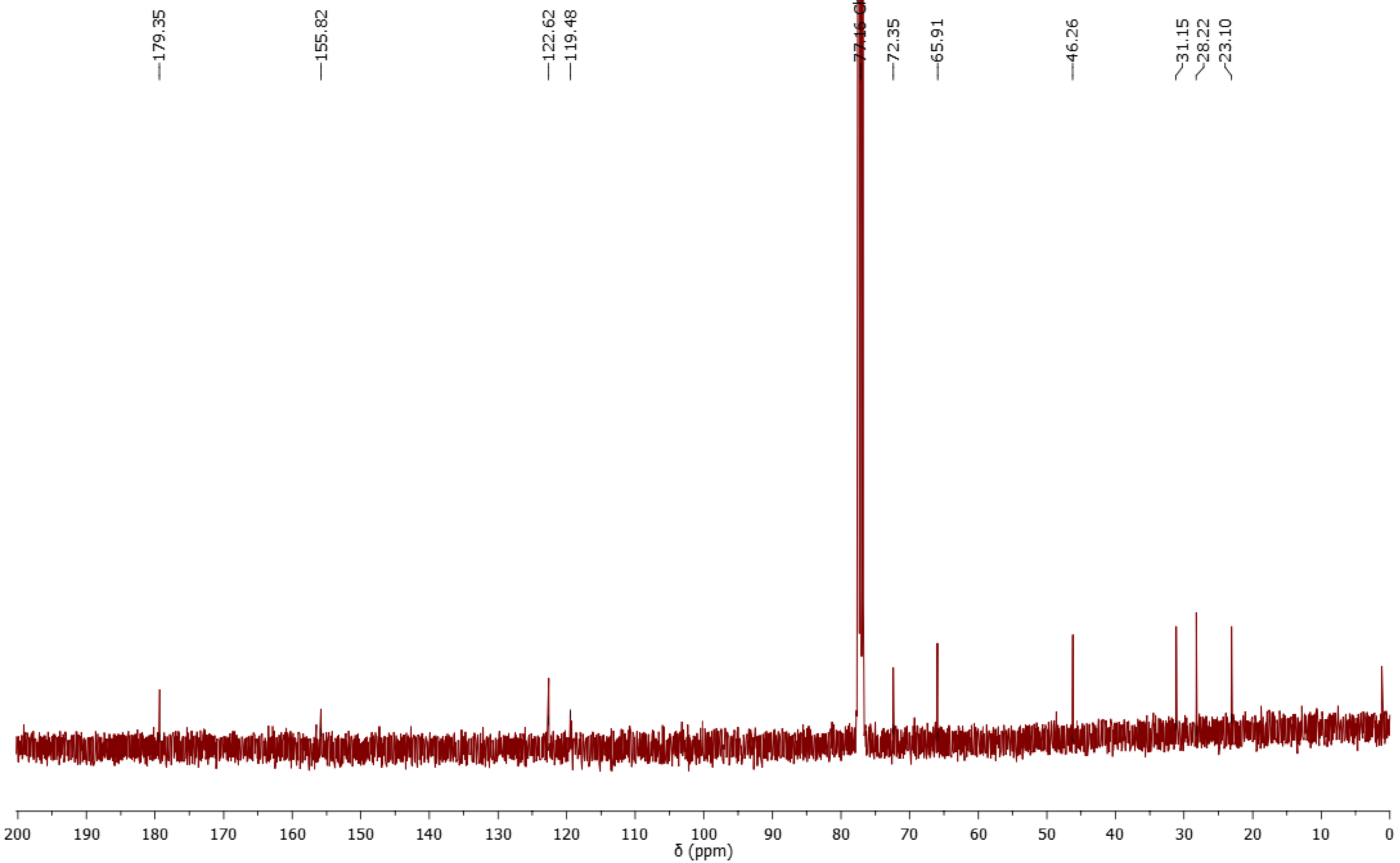

^{1}H 400MHz, $CDCl_3$

3bc

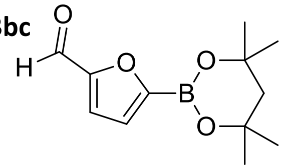

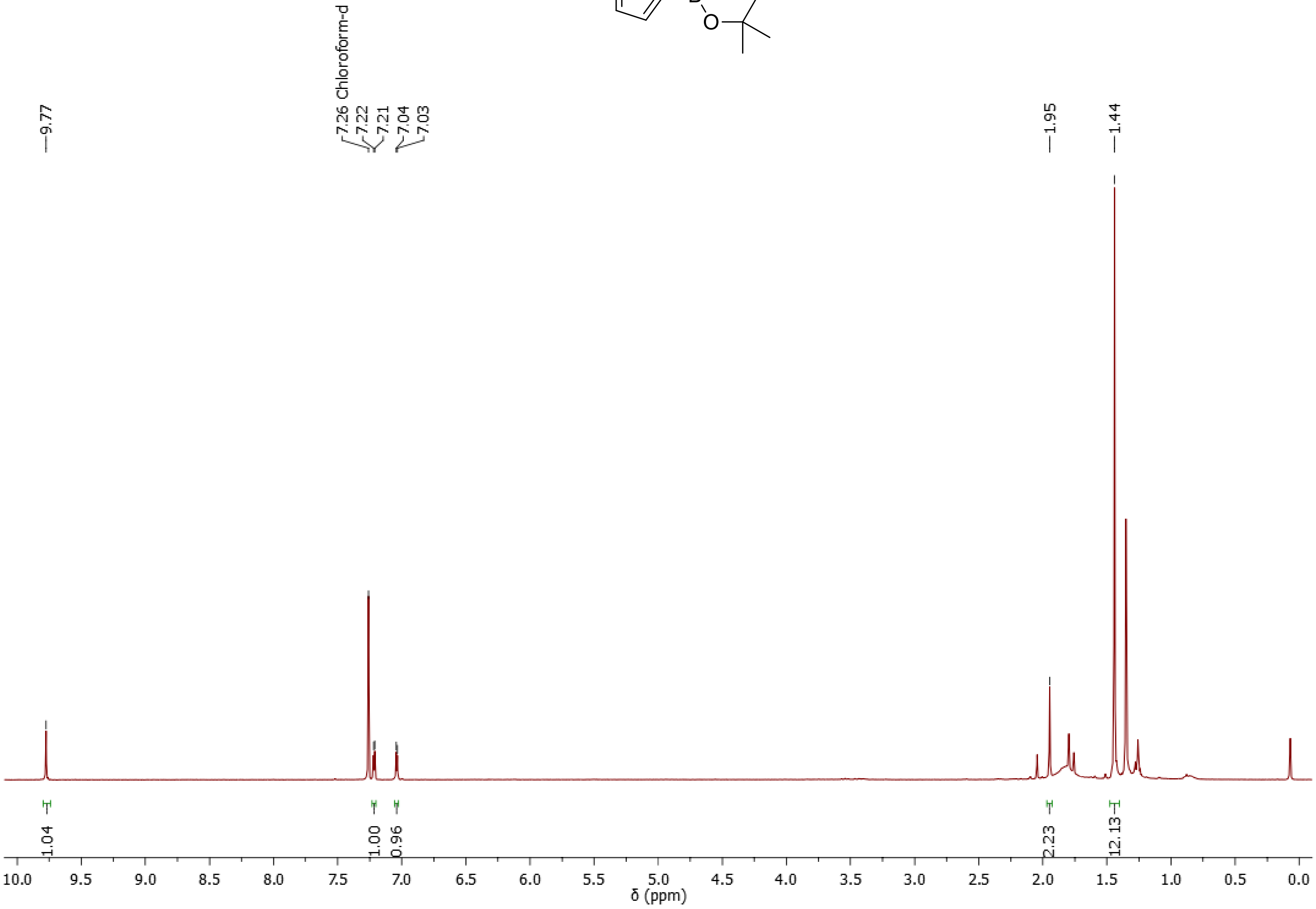

^{13}C 101MHz

3bc

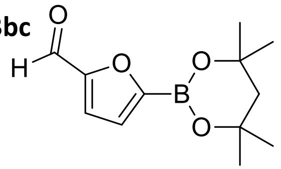

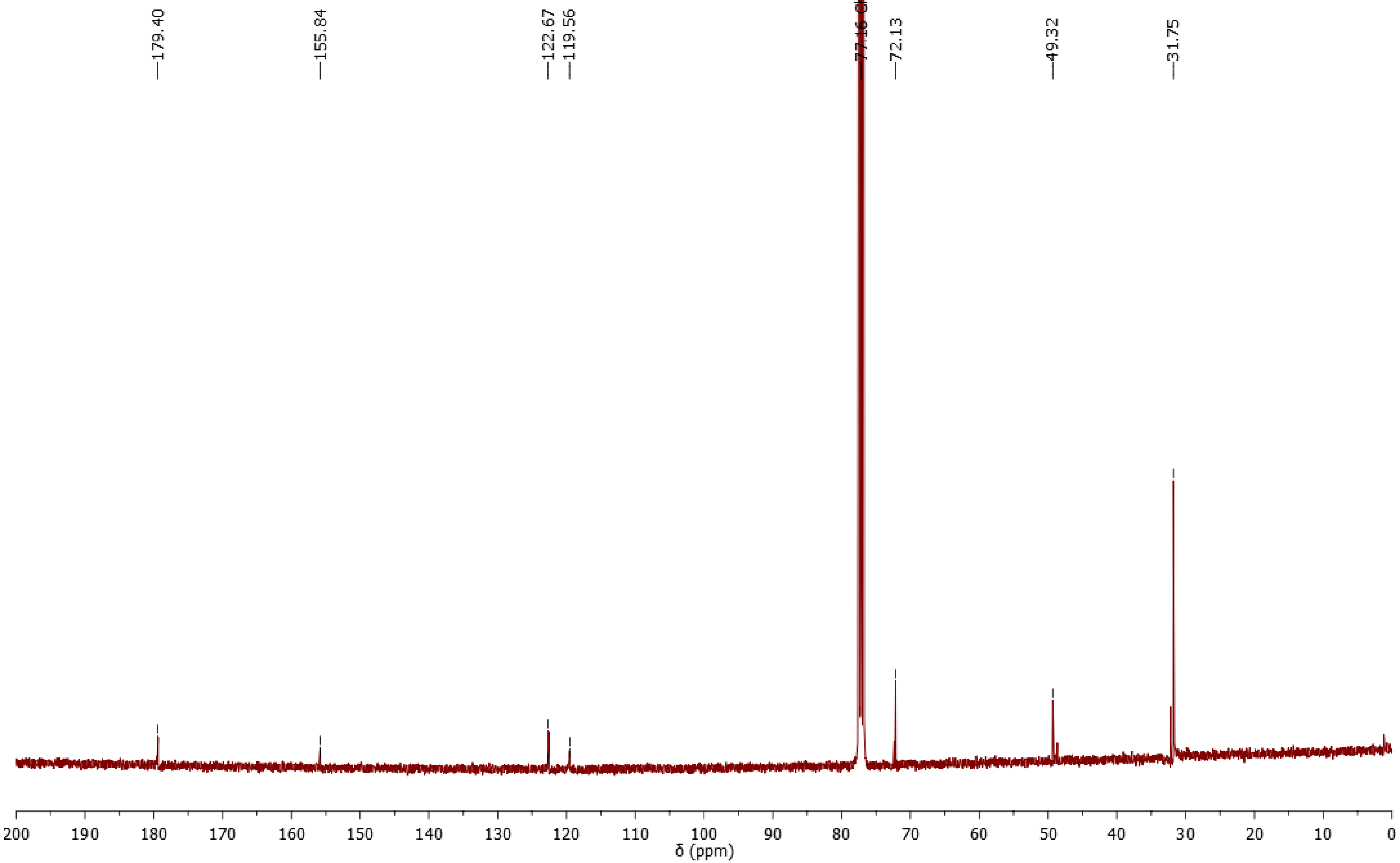

^1H 400MHz, $CDCl_3$

3bd

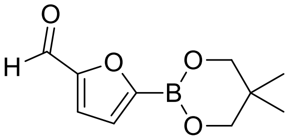

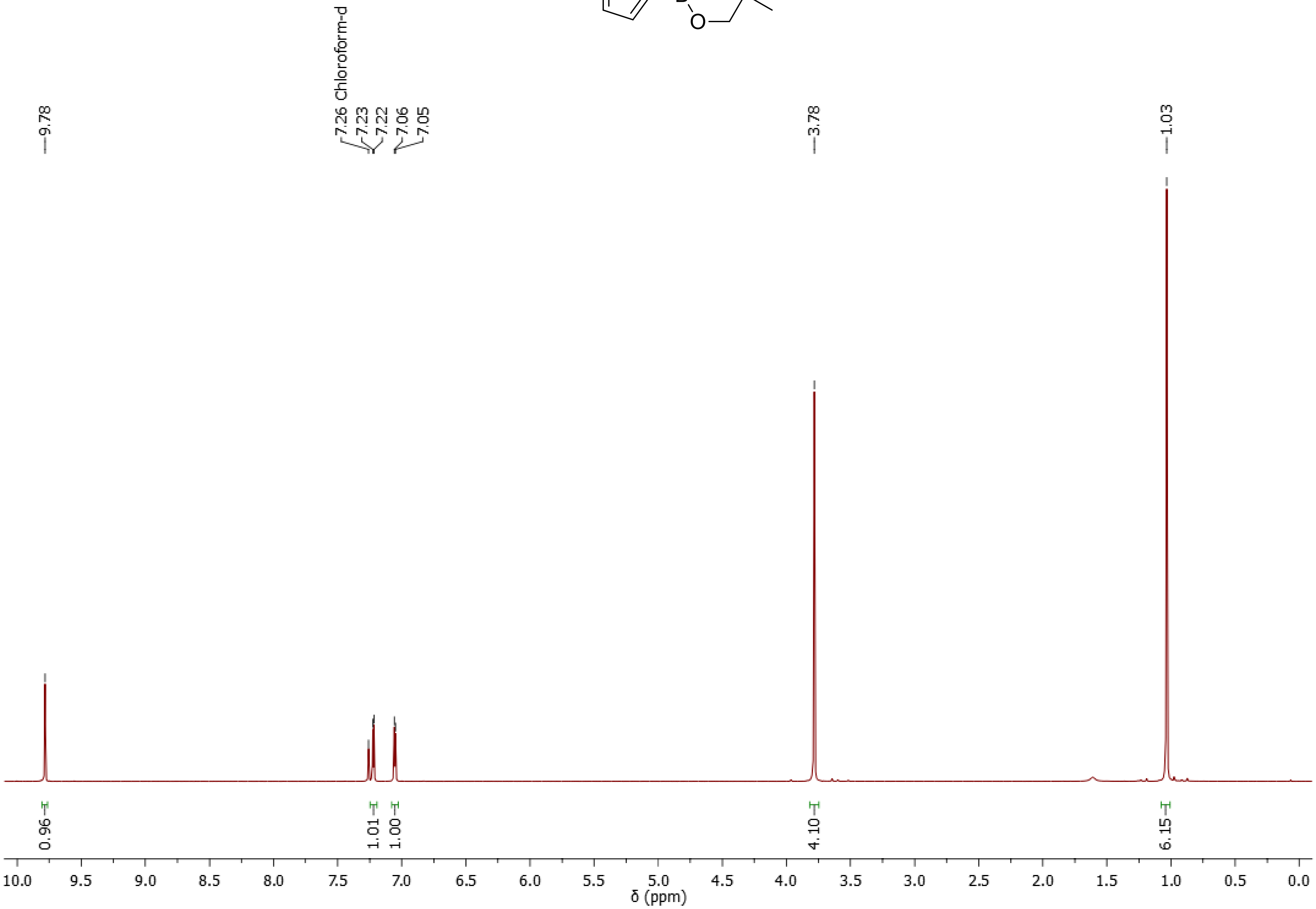

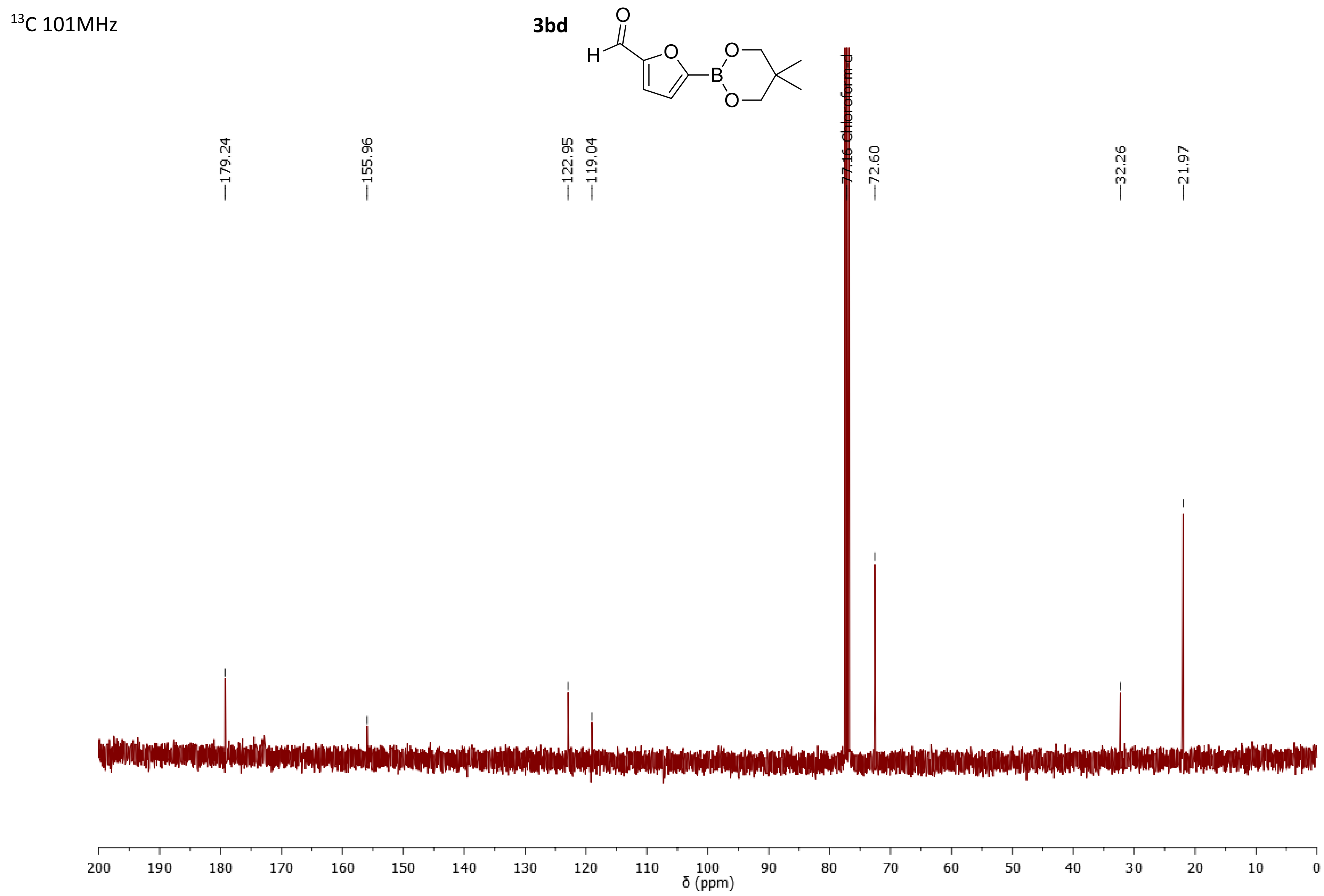

13C 101MHz
3bd
O
H
O
B
O
O
179.24
155.96
122.95
119.04
77.16 Chloroform-d
72.60
32.26
21.97
200
190
180
170
160
150
140
130
120
110
100
90
80
70
60
50
40
30
20
10
0
δ (ppm)

^{1}H 400MHz, $CDCl_3$

3ca

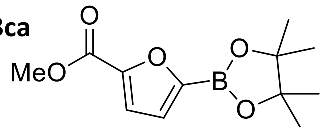

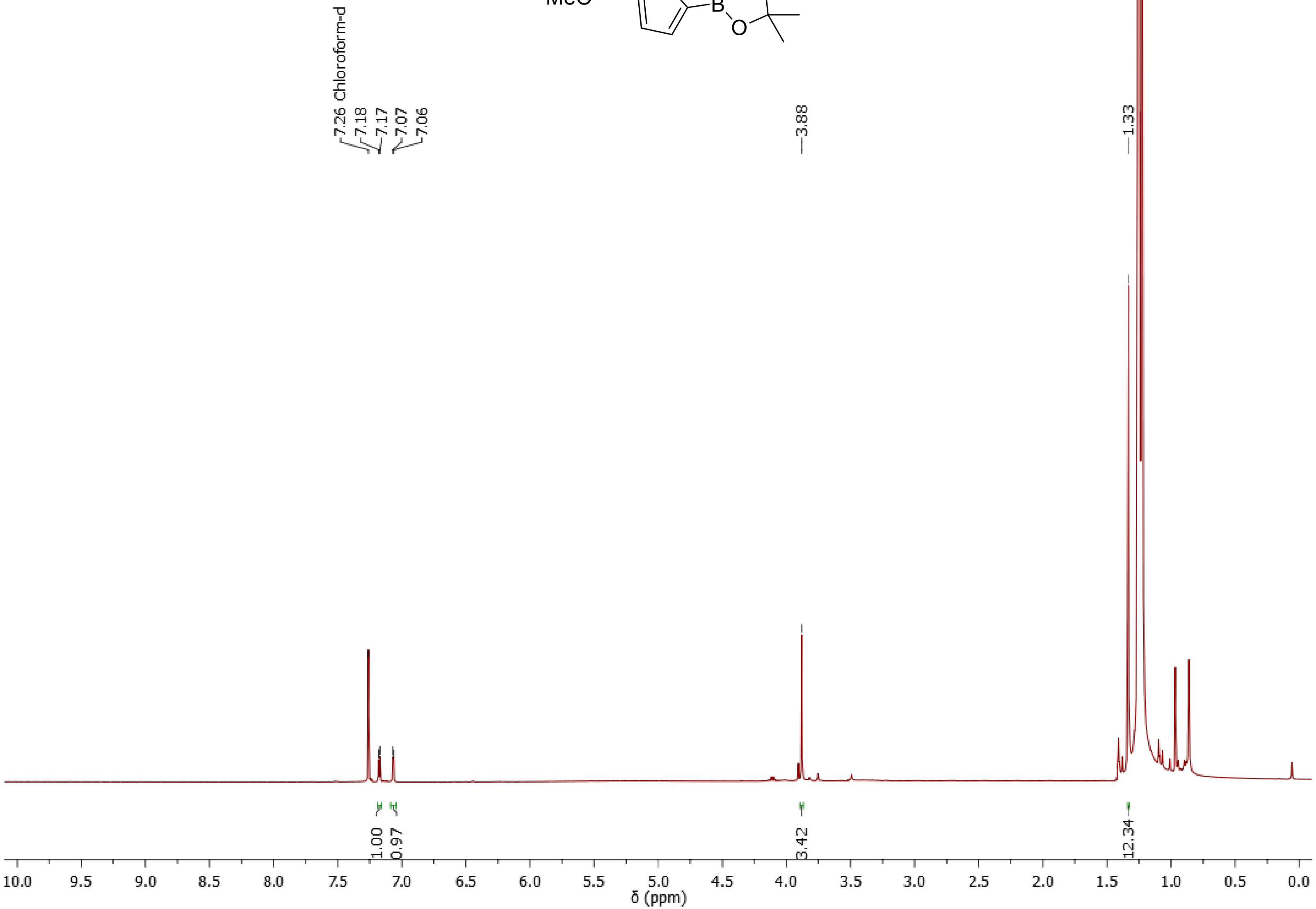

^{13}C 101MHz, $CDCl_3$

3ca

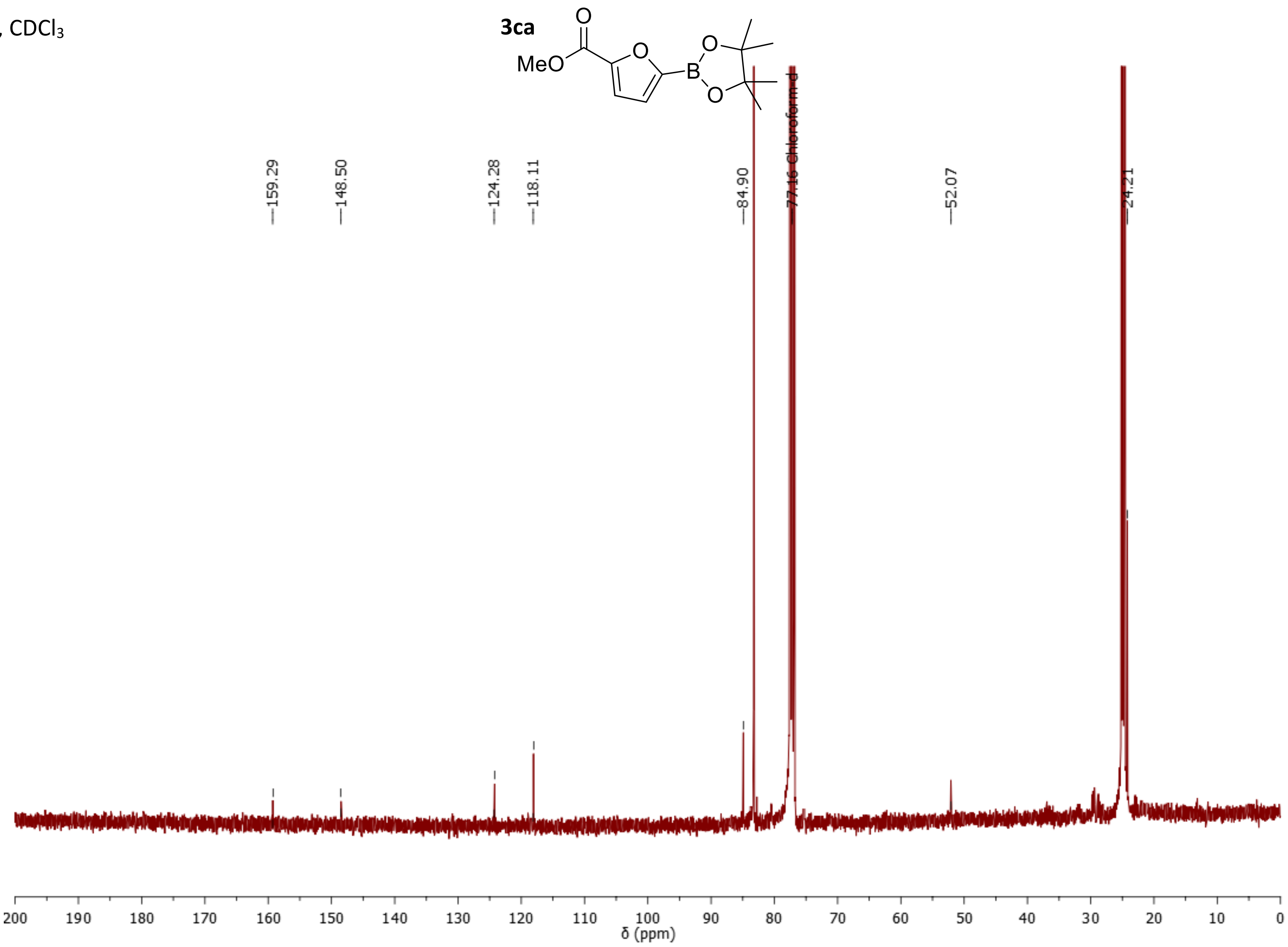

^{1}H 400MHz, $CDCl_3$

3cb

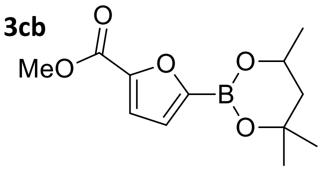

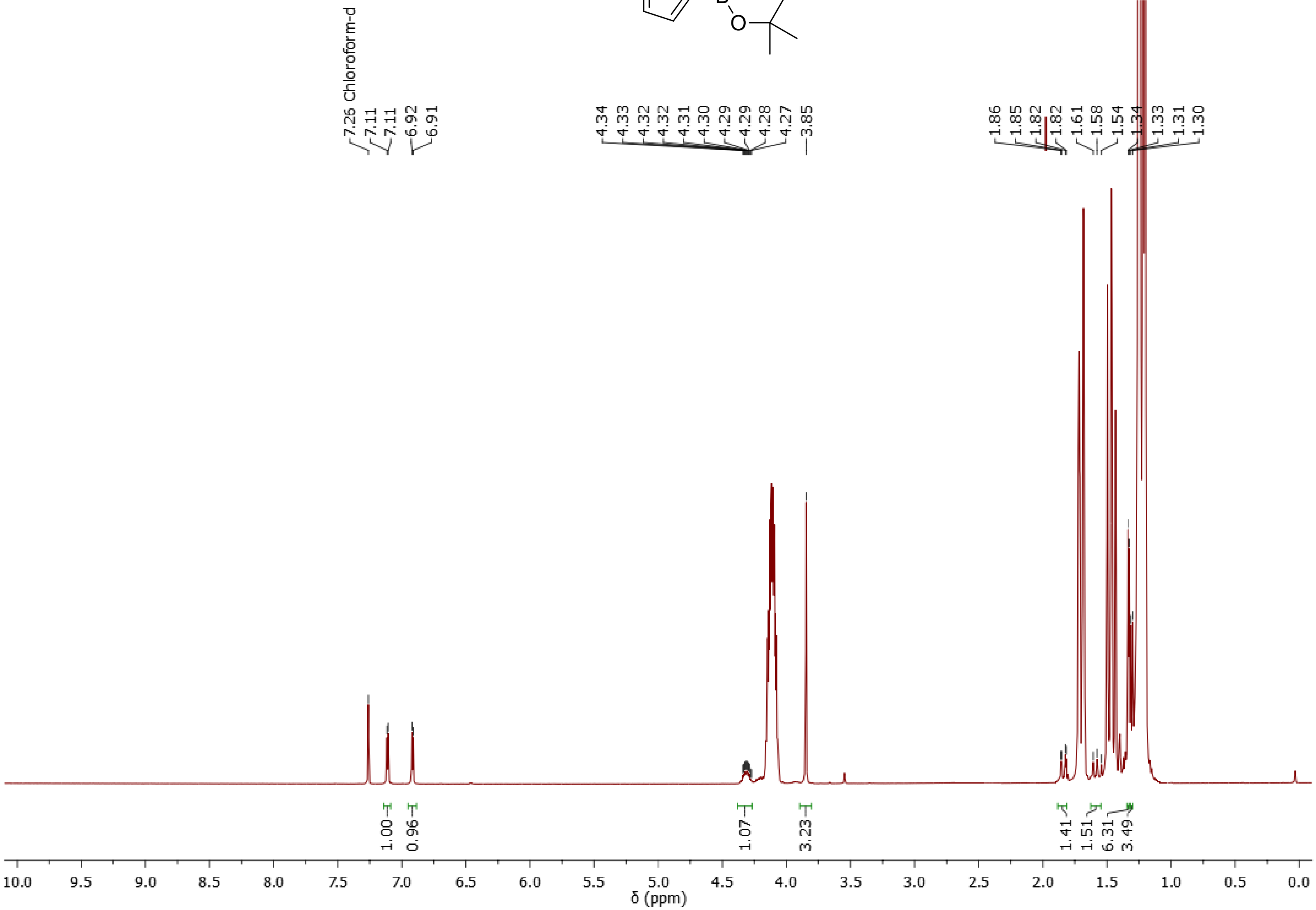

^{13}C 101MHz

3cb

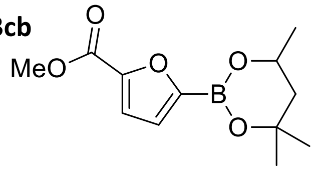

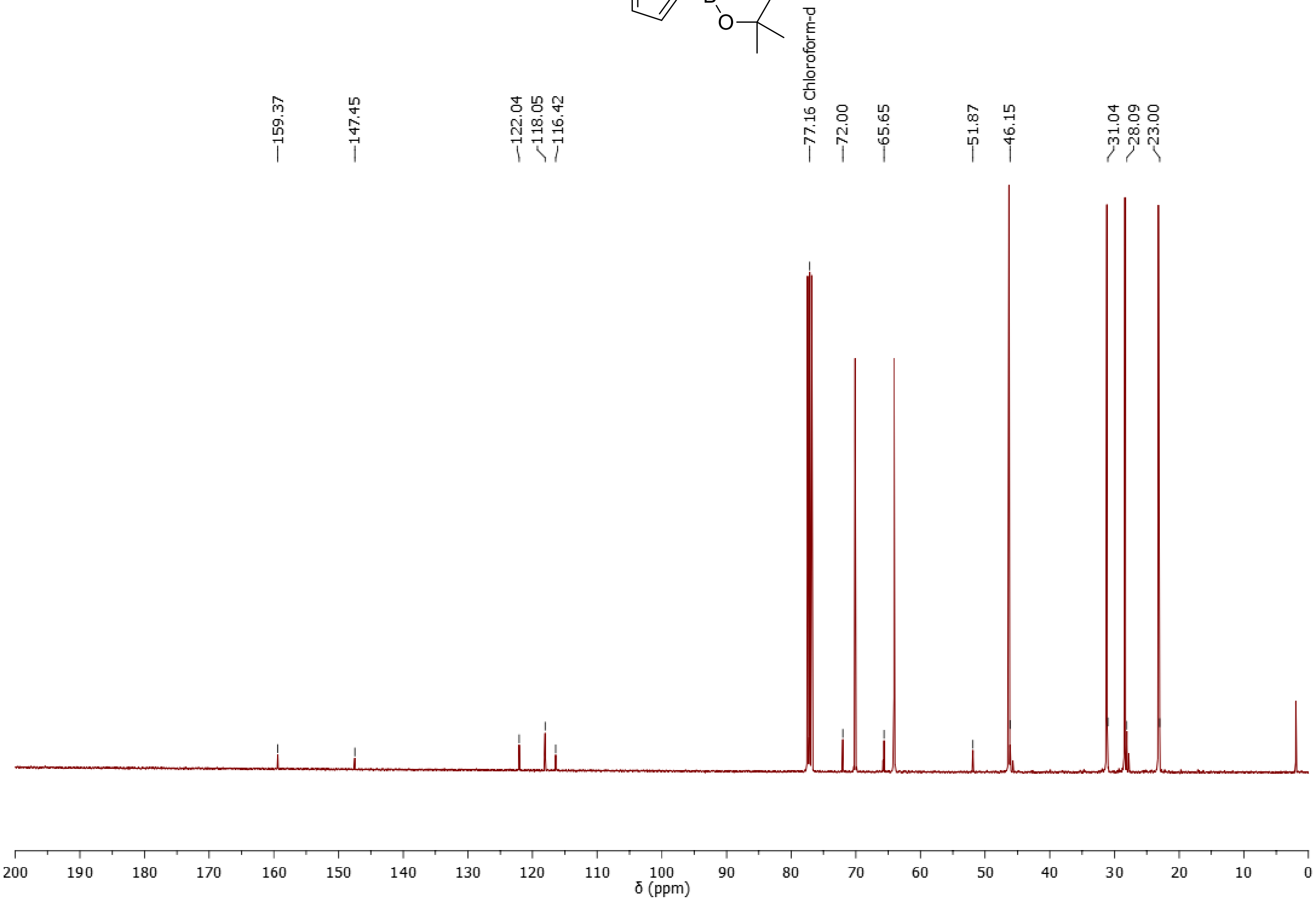

^{1}H 400MHz, $CDCl_3$

3cc

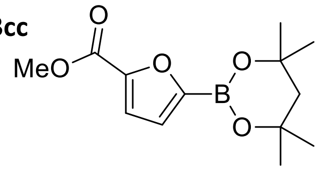

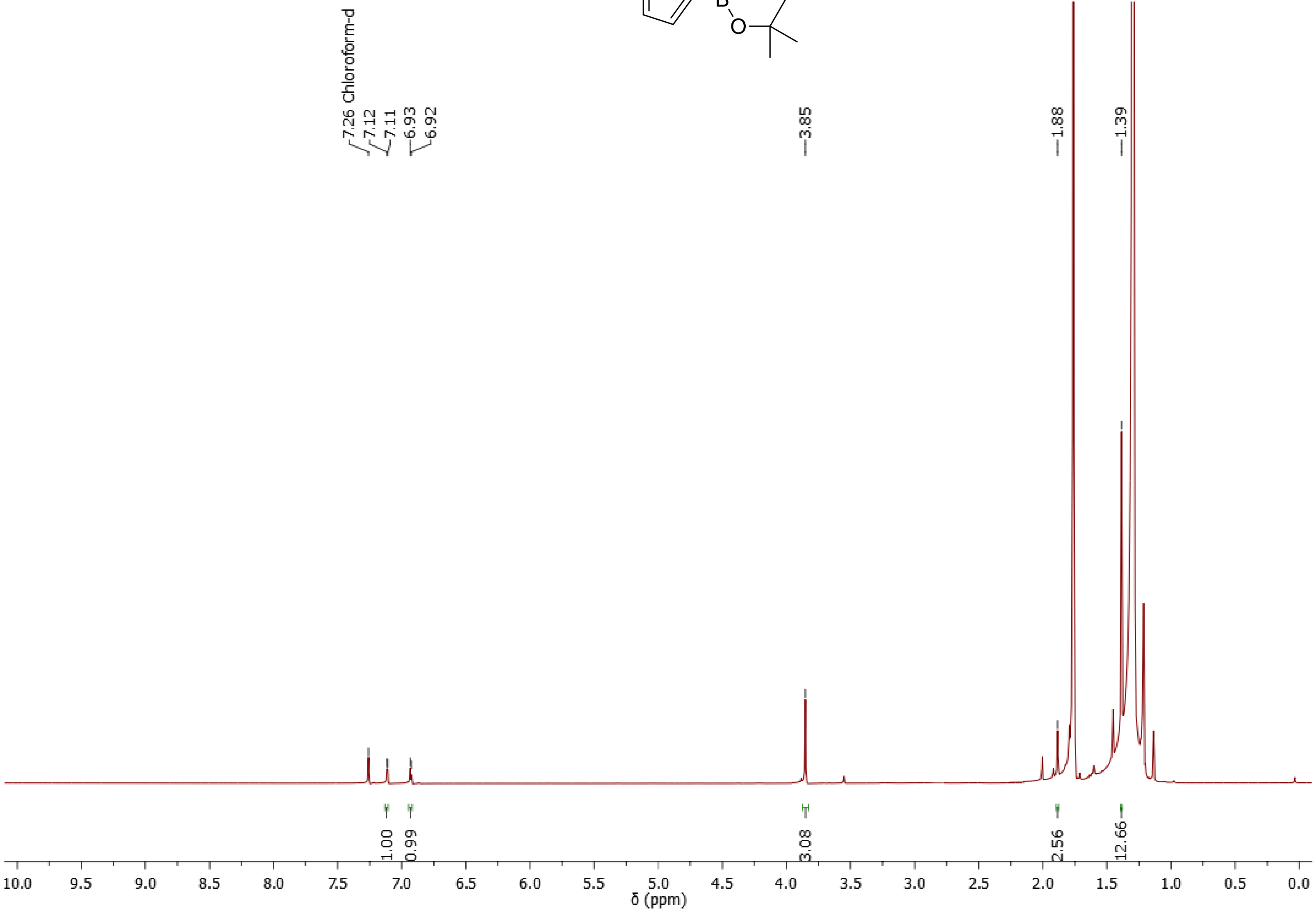

^{13}C 101MHz

3cc

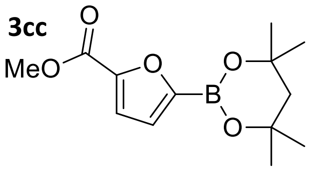

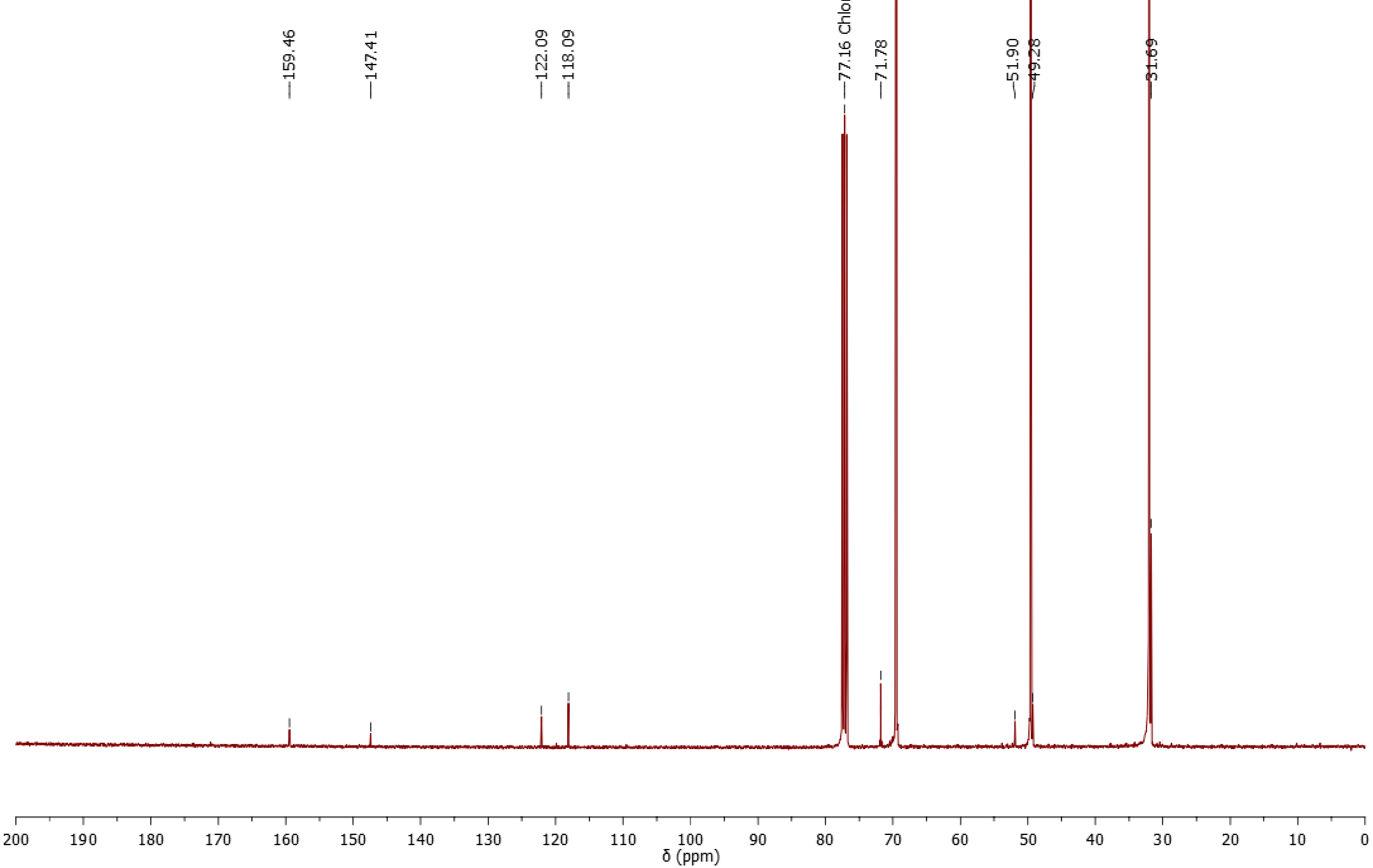

^{1}H 400MHz, $CDCl_3$

3cd

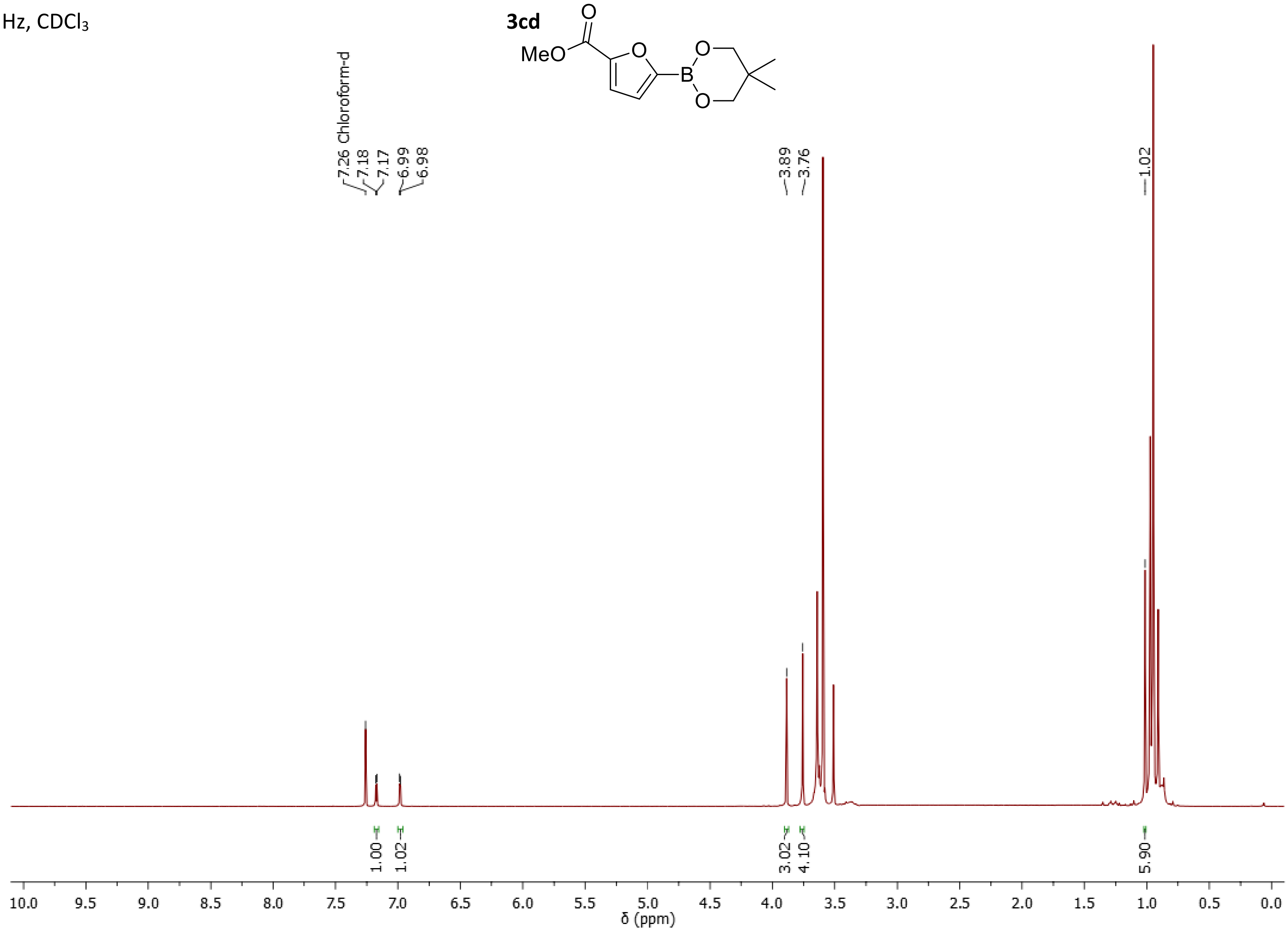

^{13}C 101MHz

3cd

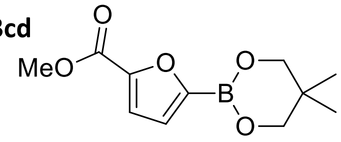

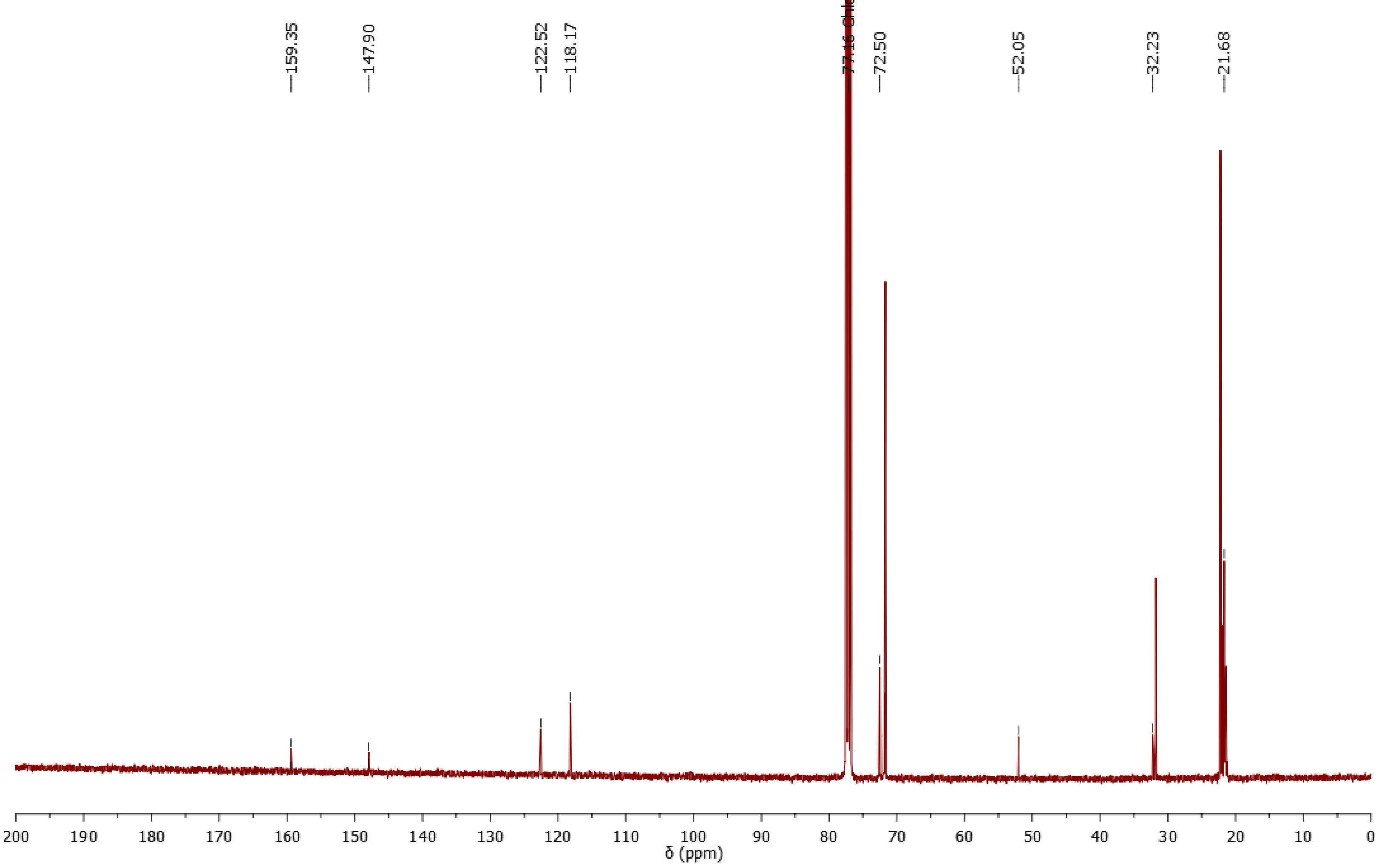

^{1}H 400MHz, $CDCl_3$

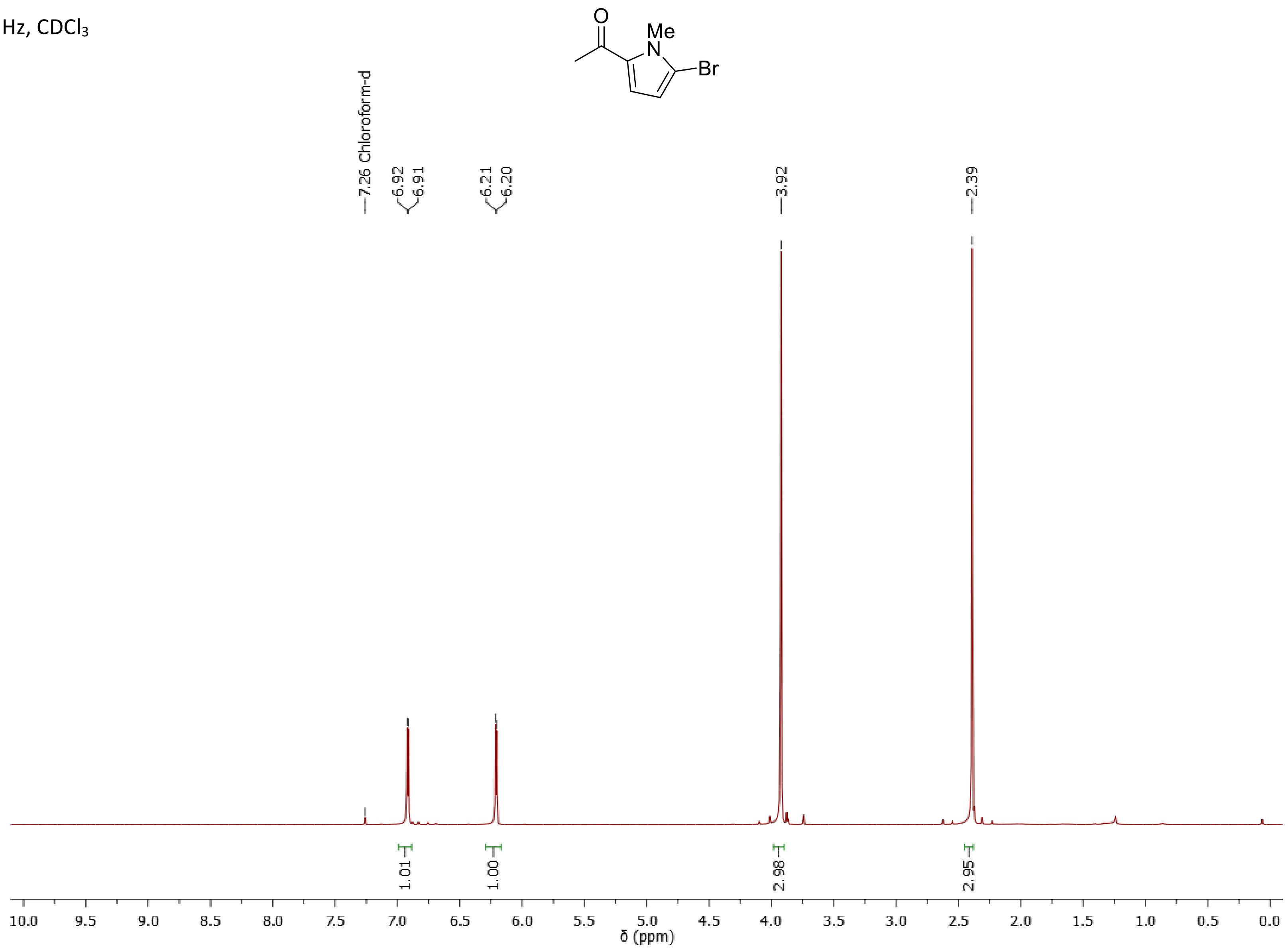

^{13}C 101MHz, $CDCl_3$

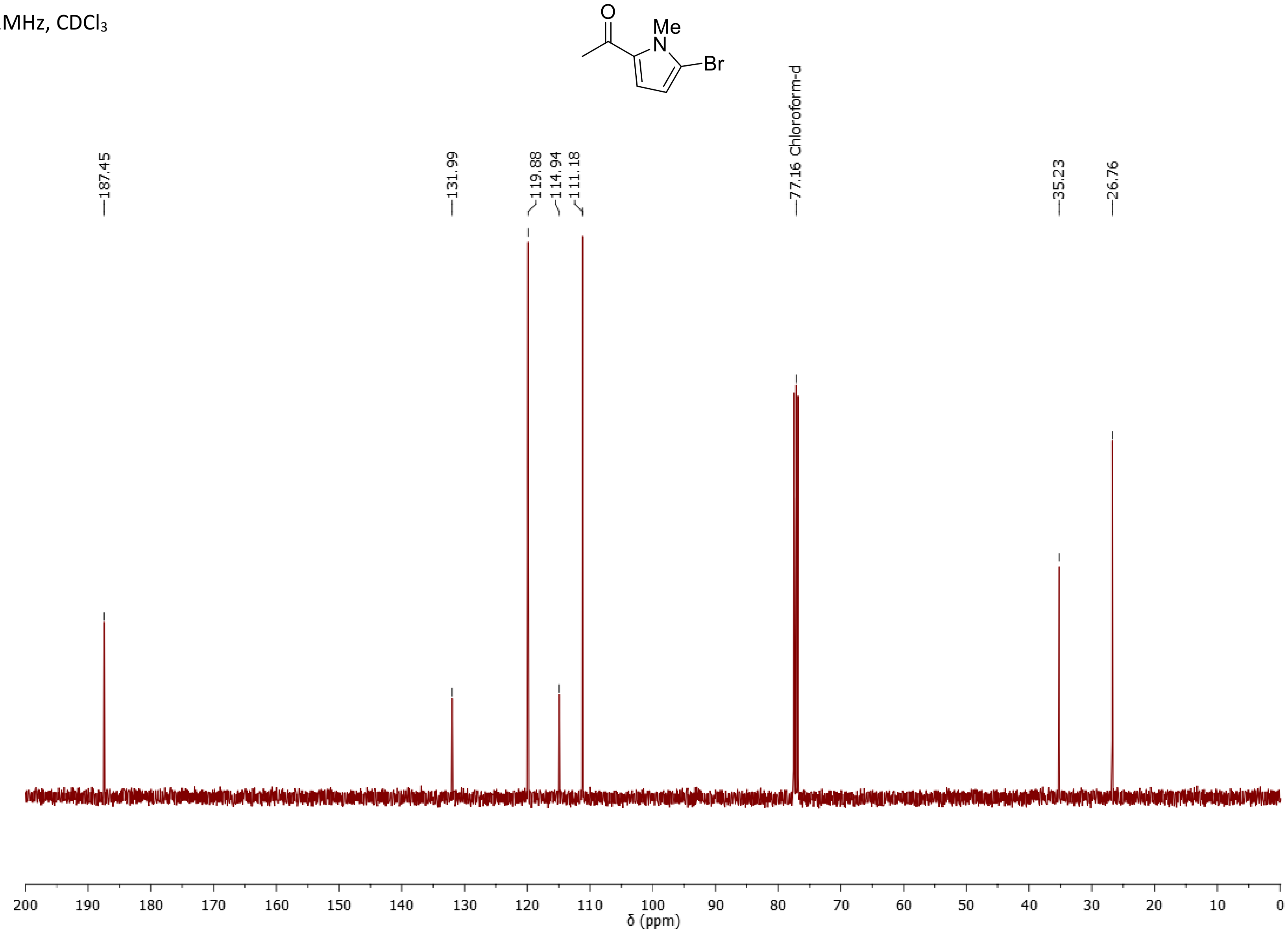

^{1}H 400MHz, $CDCl_3$

4aa

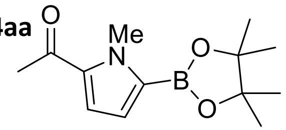

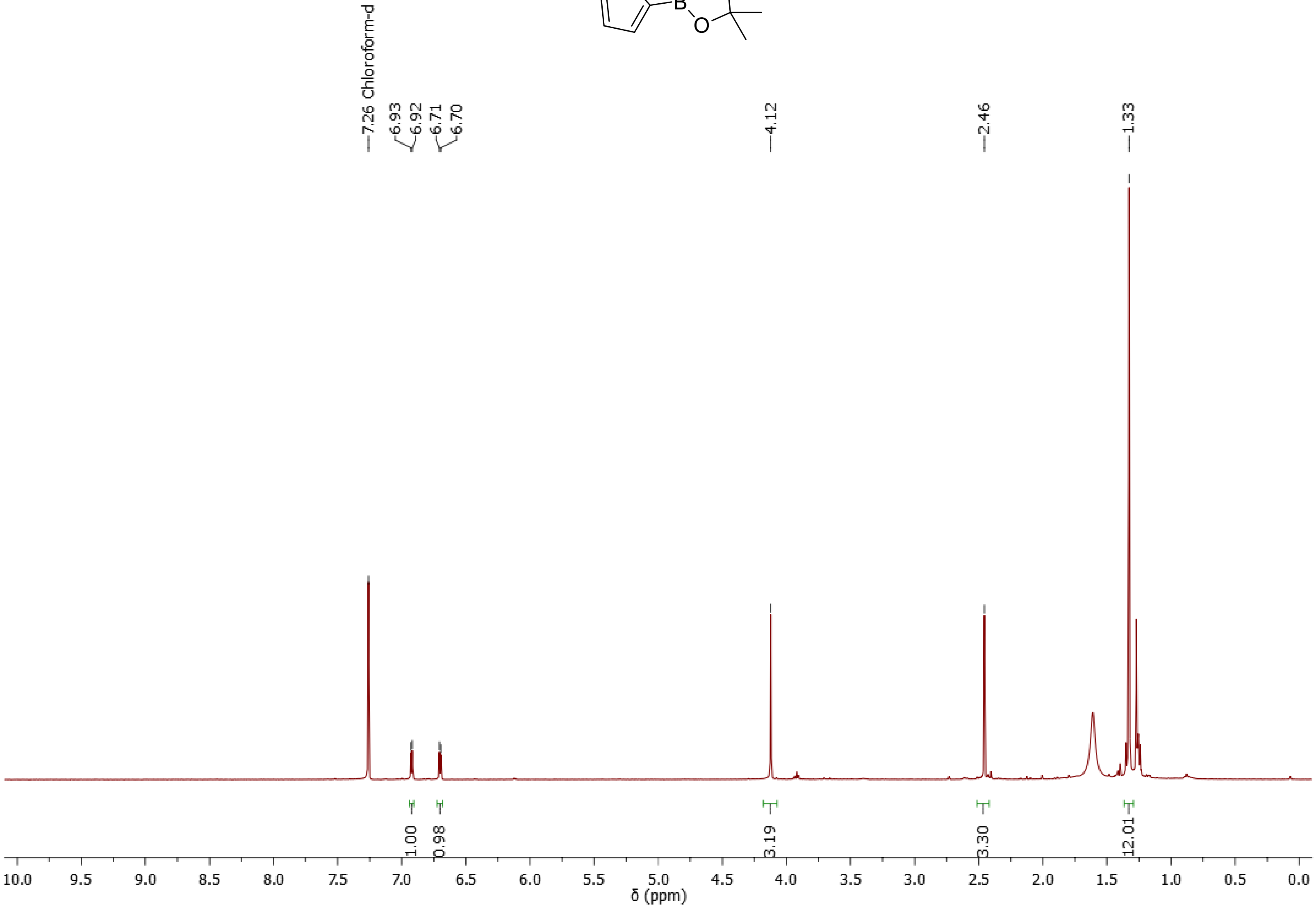

^{13}C 101MHz, $CDCl_3$

4aa

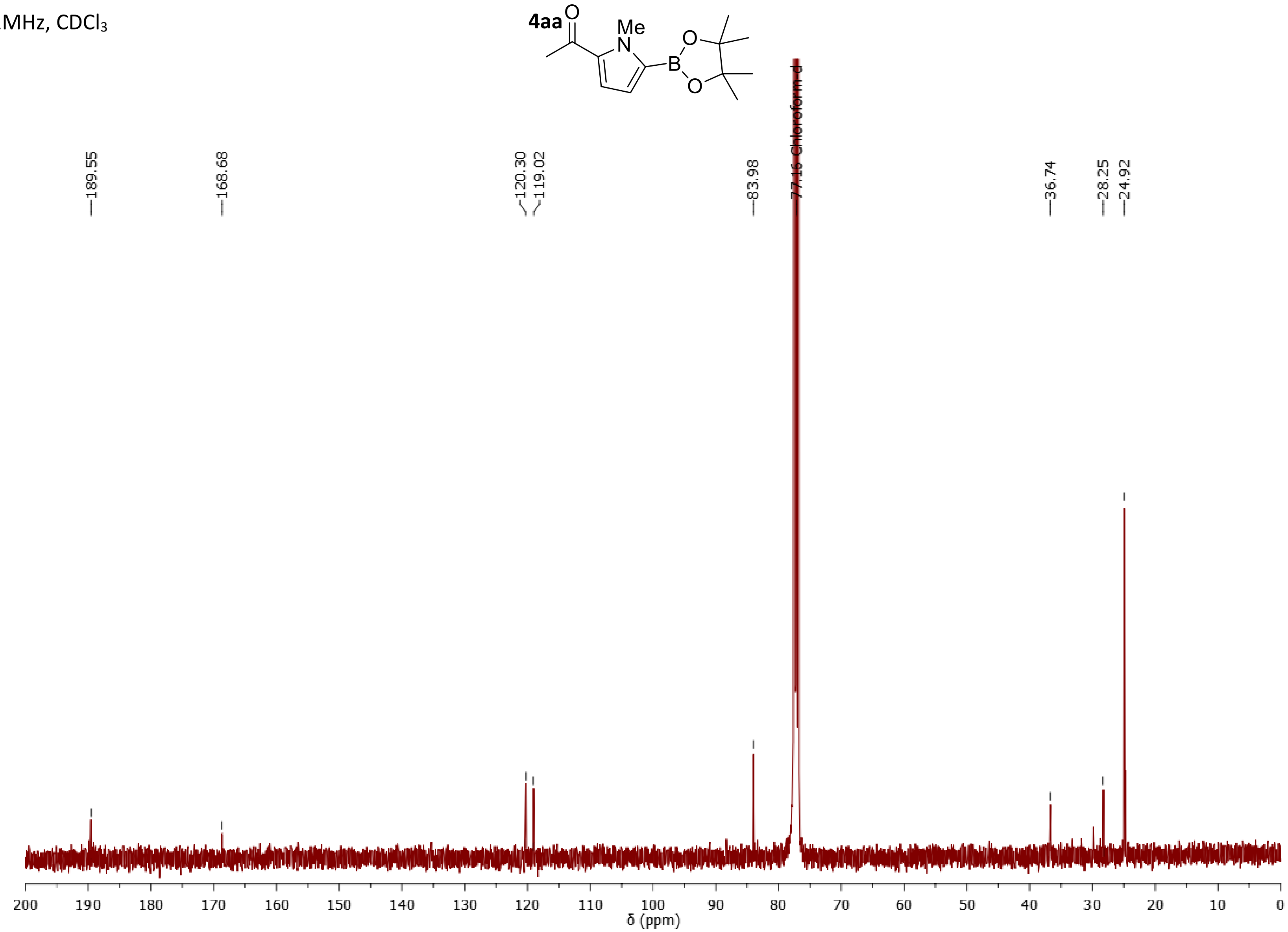

^{1}H 400MHz, $CDCl_3$

4ab

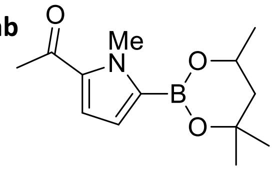

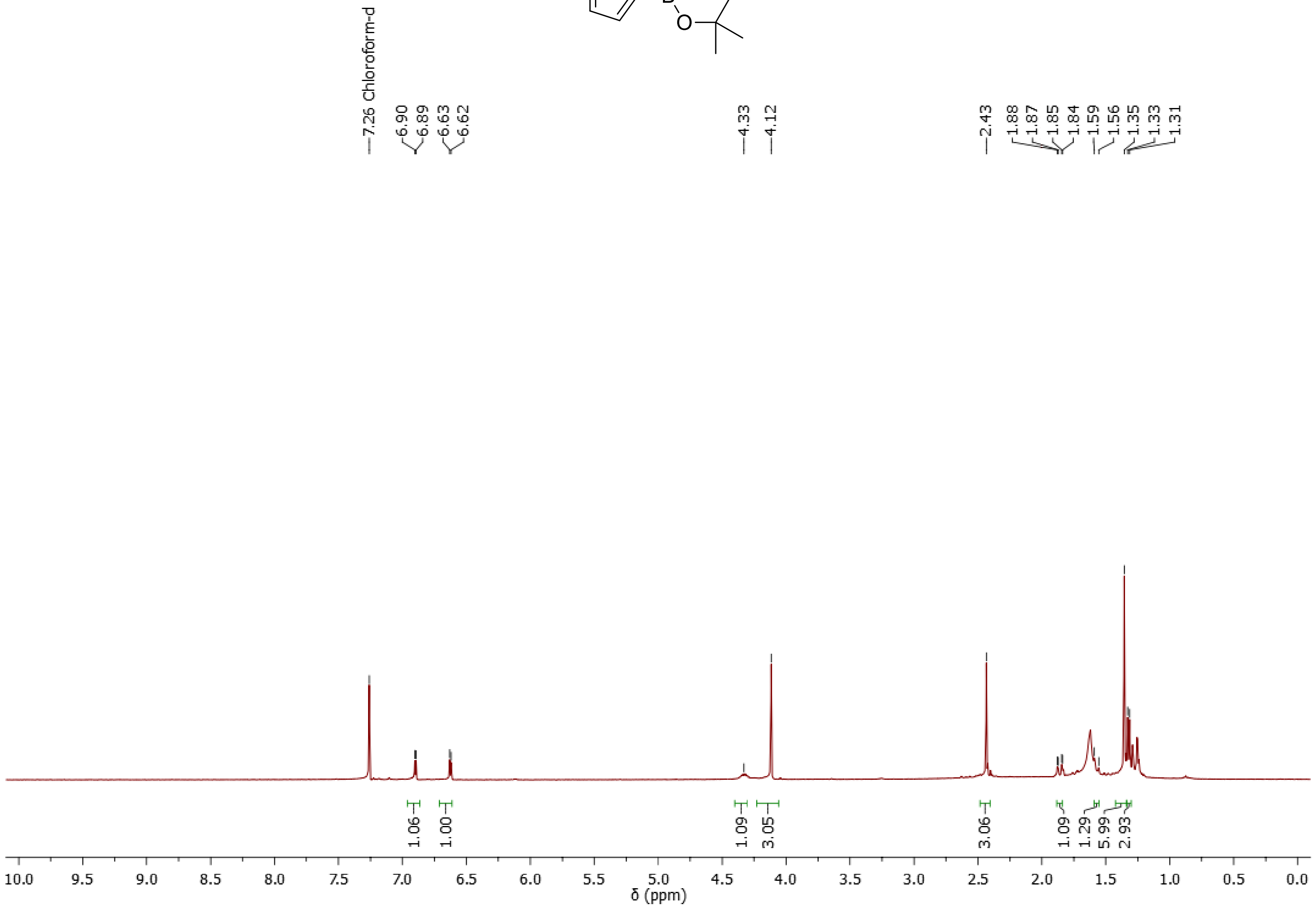

^{13}C 101MHz

4ab

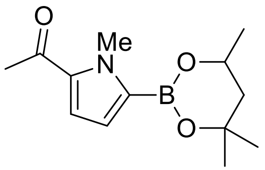

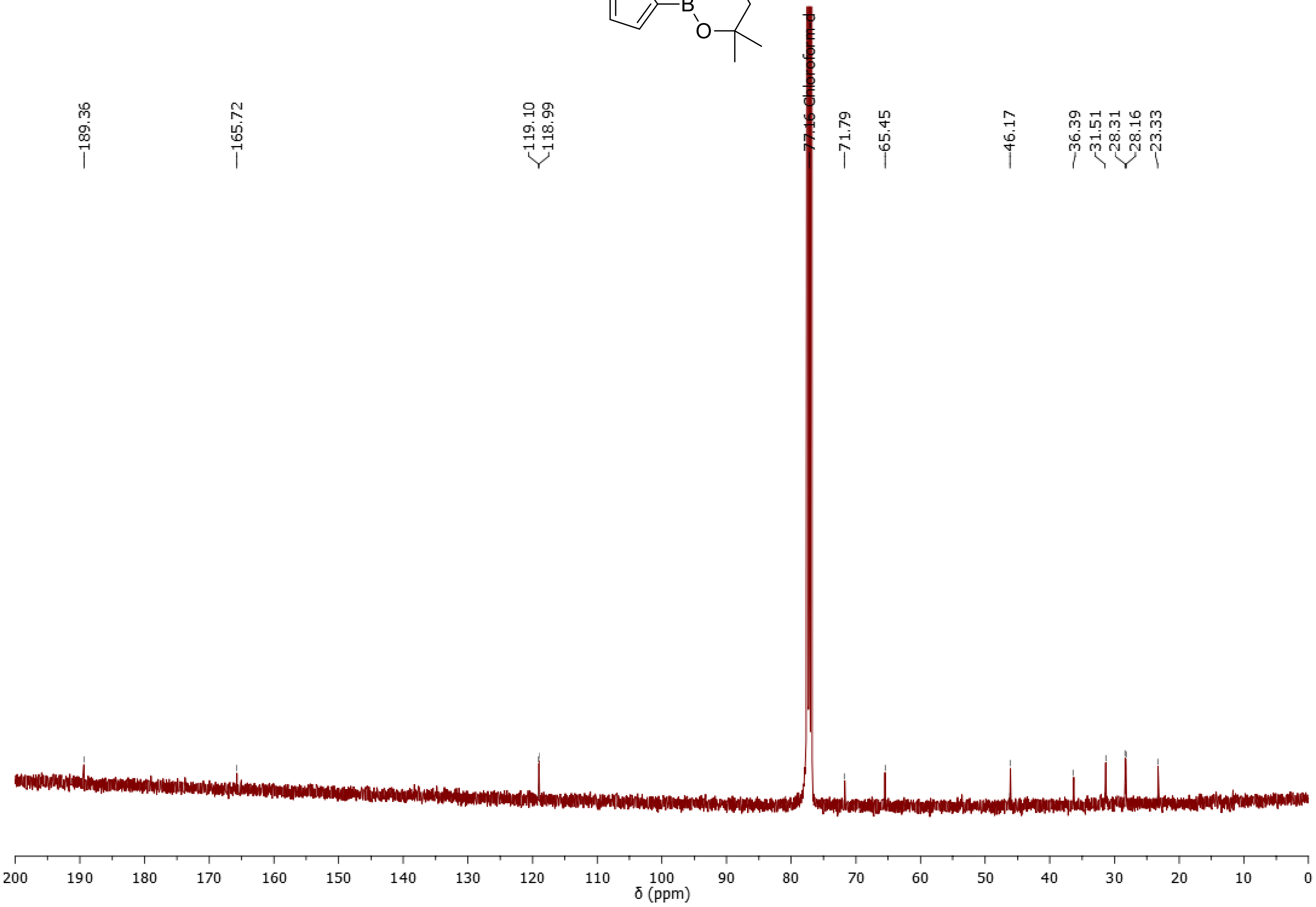

^{1}H 400MHz, $CDCl_3$

4ac

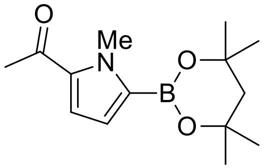

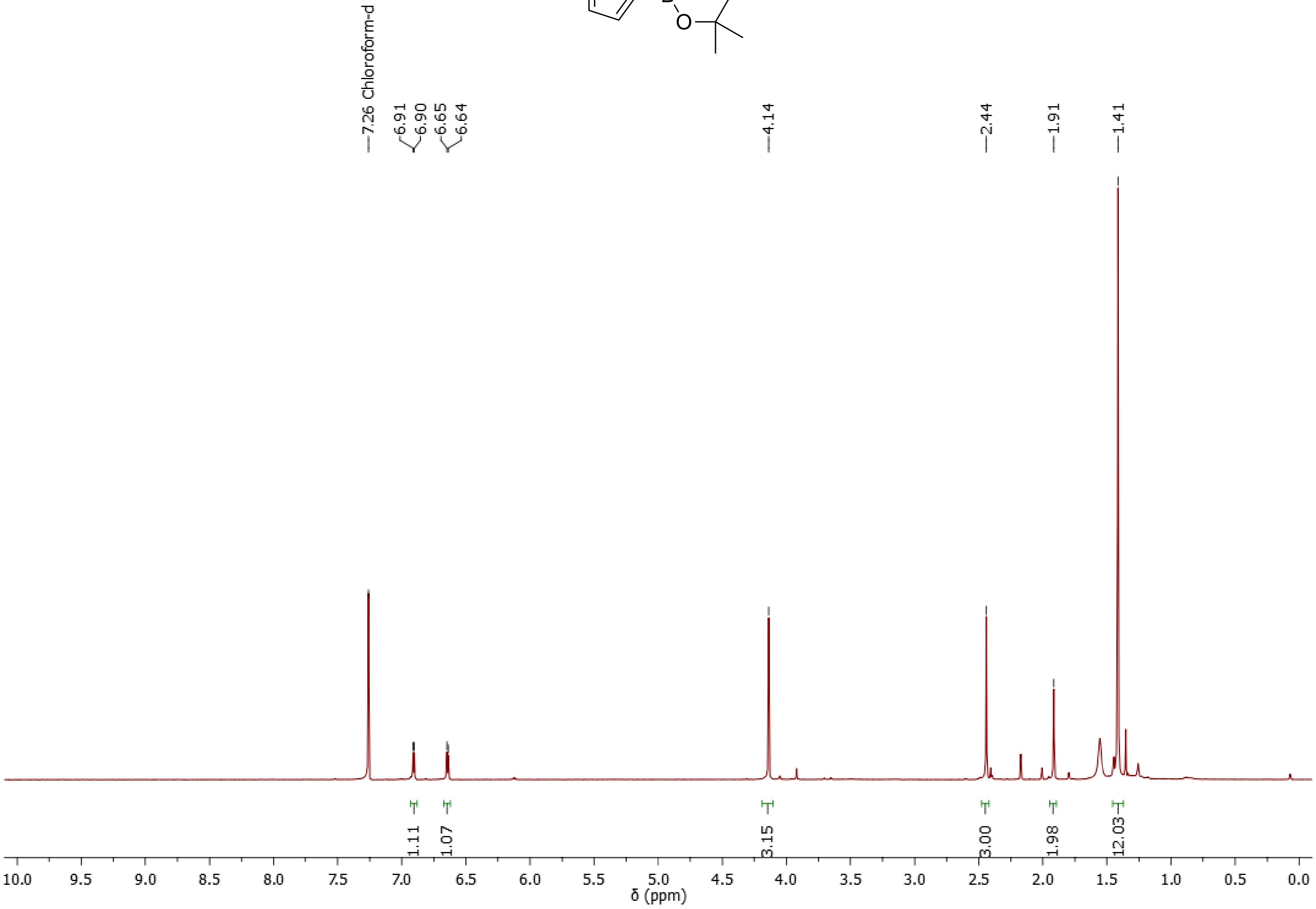

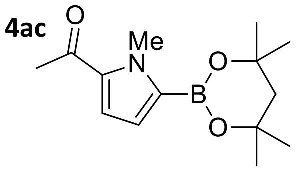
4ac
O
Me
N
B
O
O

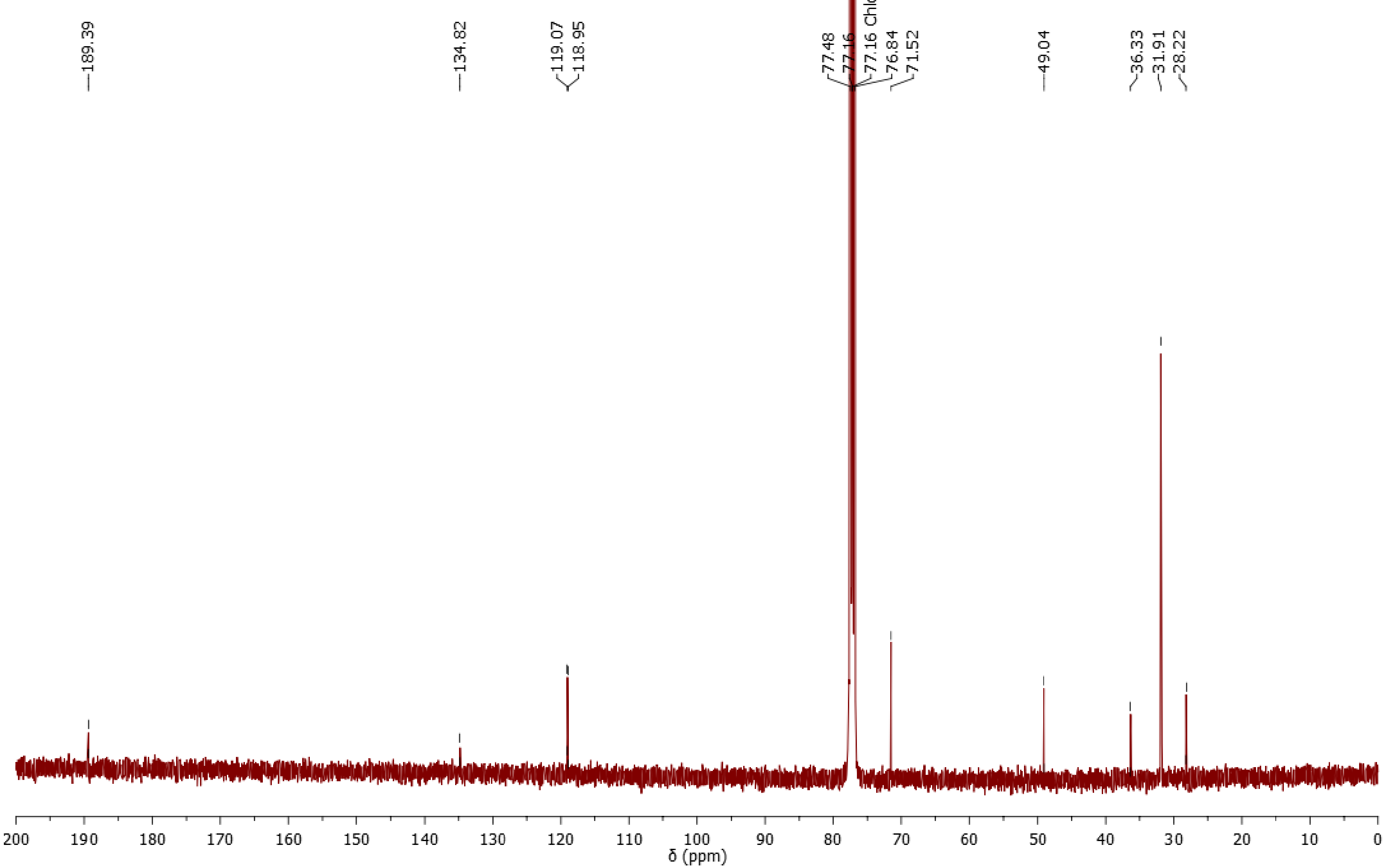
13C 101MHz
189.39
134.82
119.07
118.95
77.48
77.16
77.16 Chloroform-d
76.84
71.52
49.04
36.33
31.91
28.22
200
190
180
170
160
150
140
130
120
110
100
90
80
70
60
50
40
30
20
10
0
δ (ppm)

^1H 400MHz, $CDCl_3$

4ad

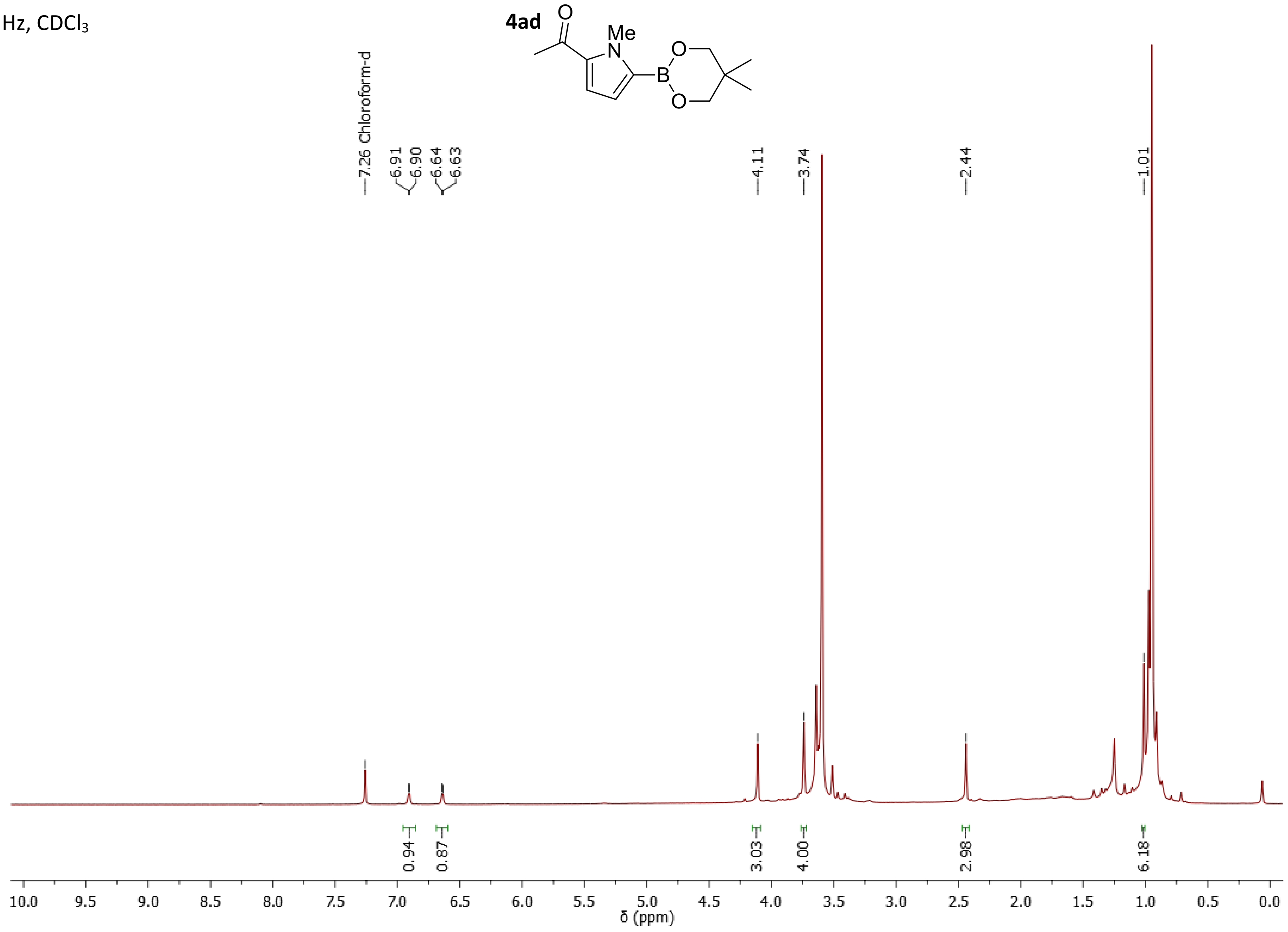

^{13}C 101MHz

4ad

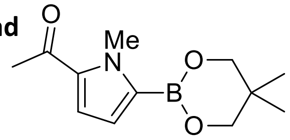

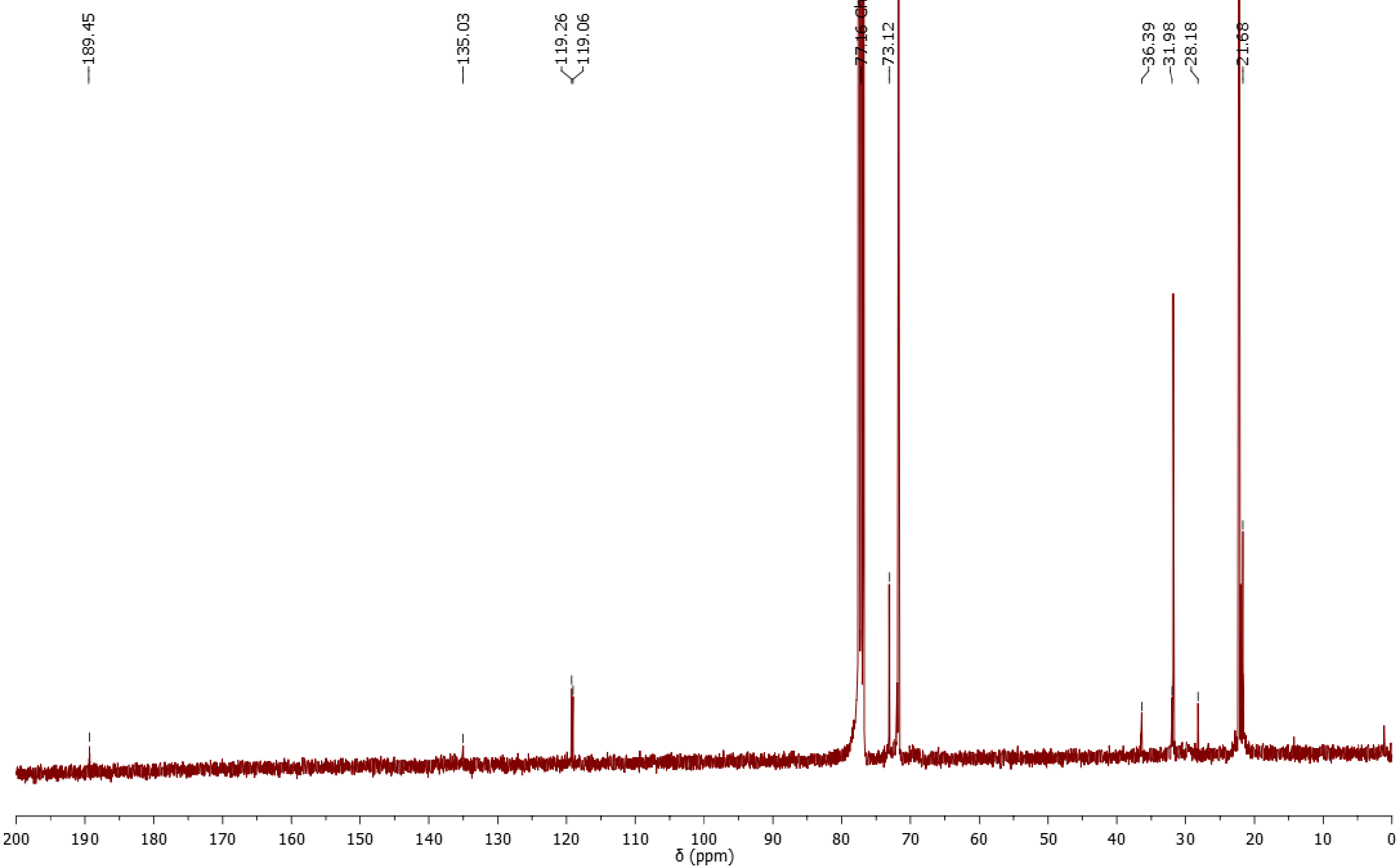

^{1}H 400MHz, $CDCl_3$

4ba

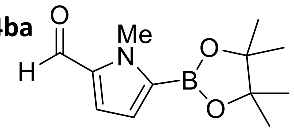

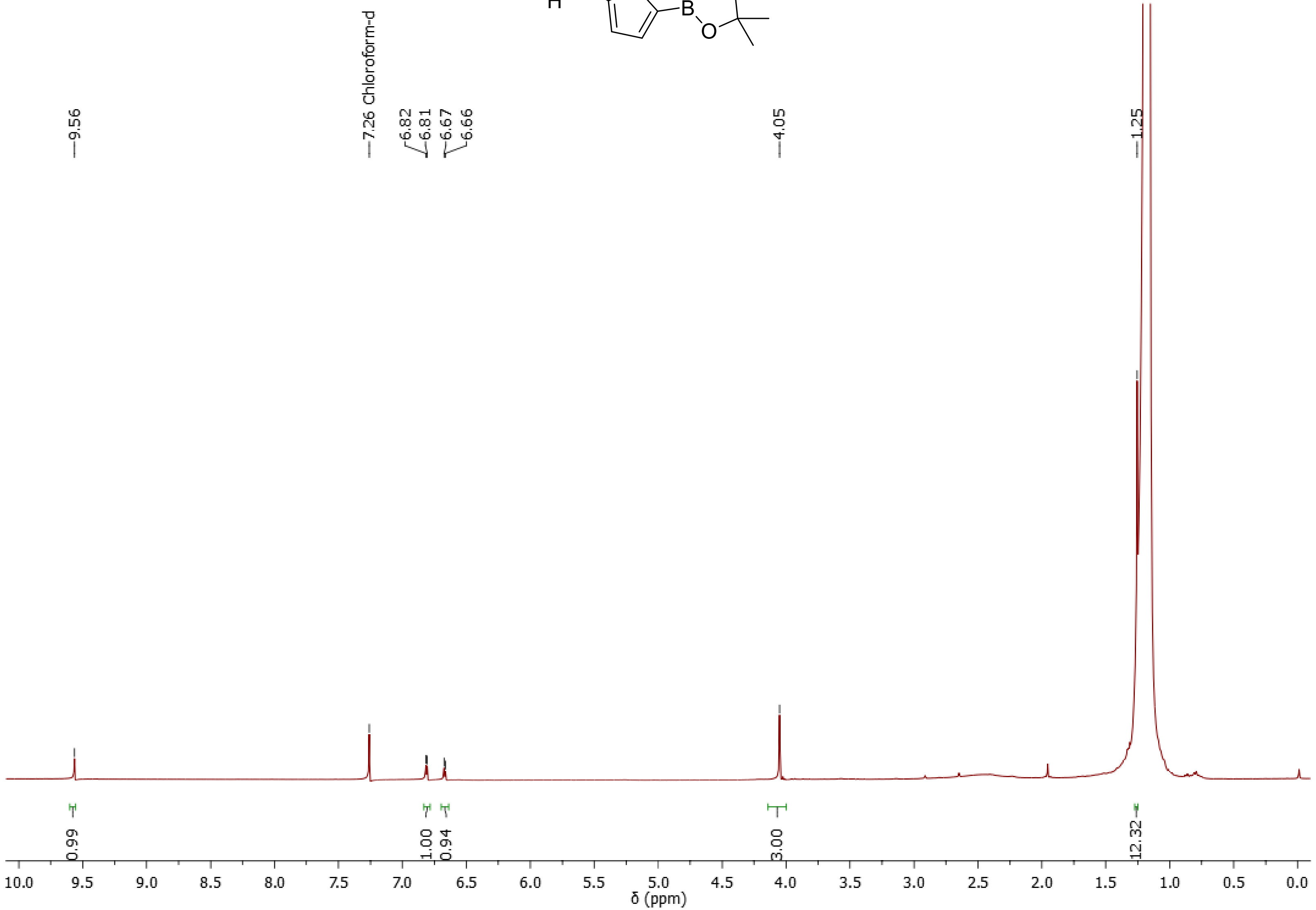

^{13}C 101MHz, $CDCl_3$

4ba

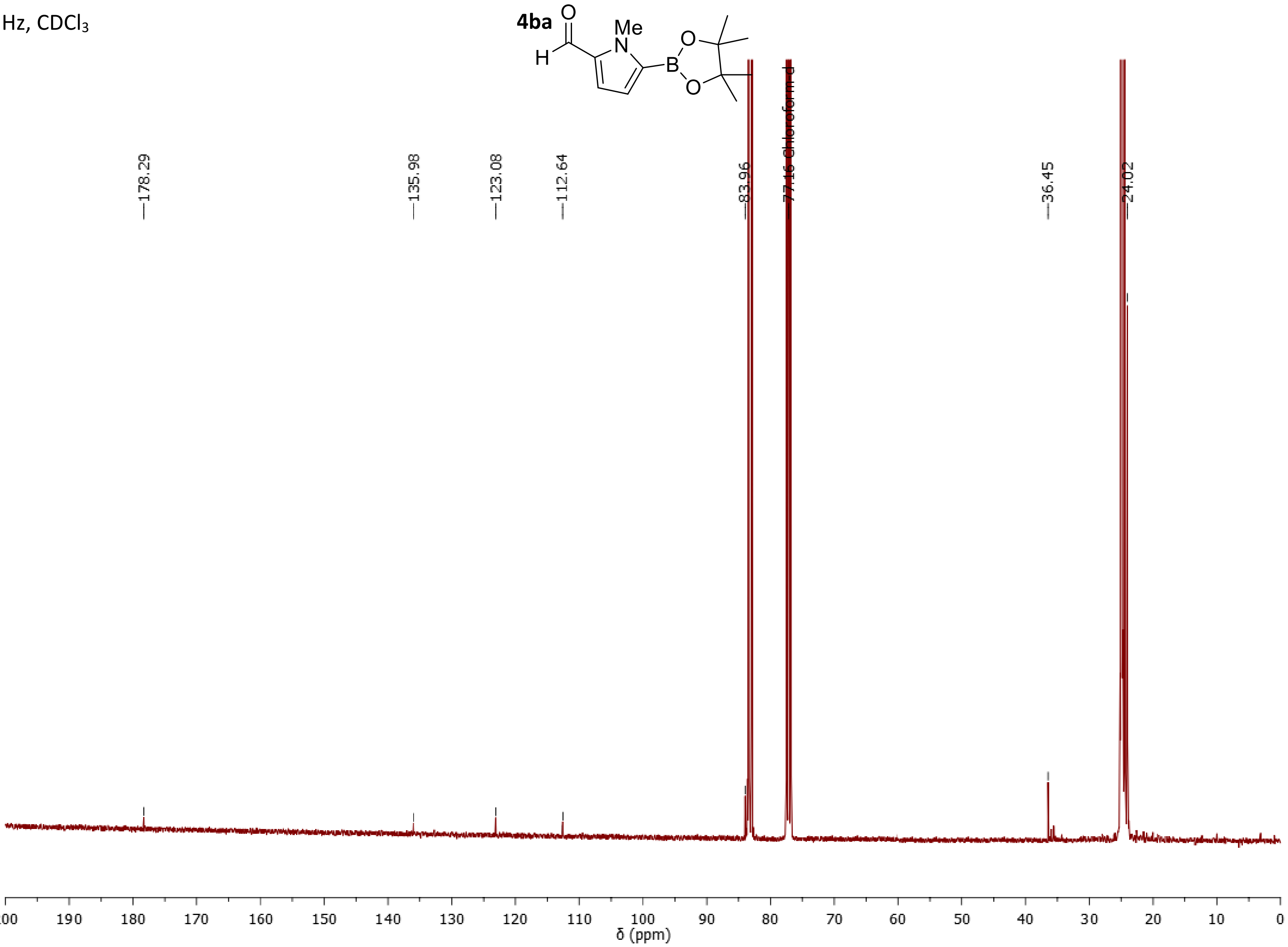

^{1}H 400MHz, $CDCl_3$

4bb

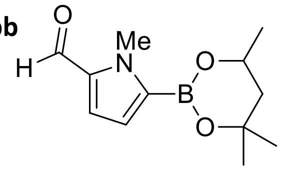

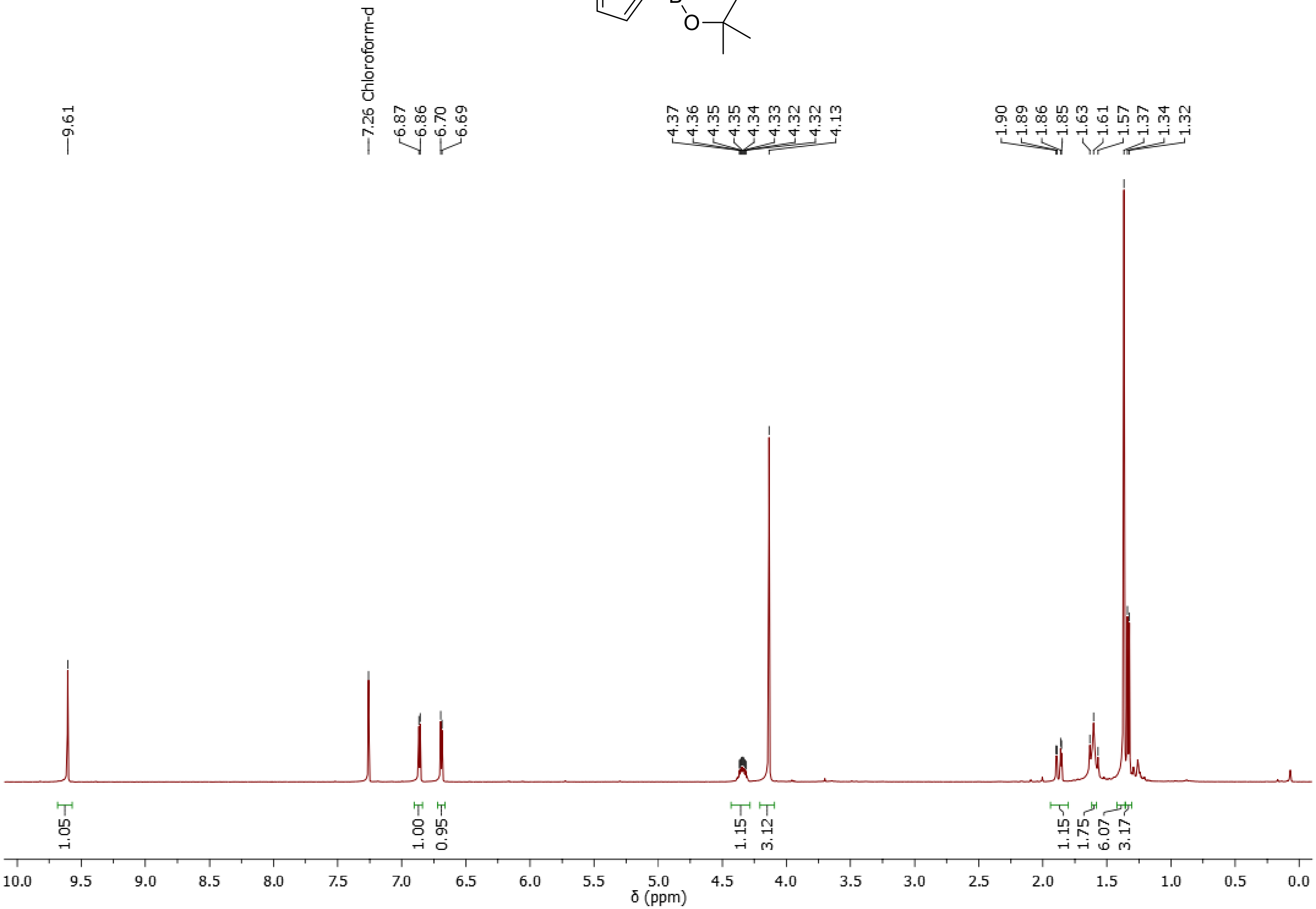

^{13}C 101MHz

4bb

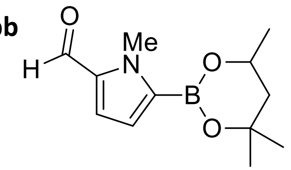

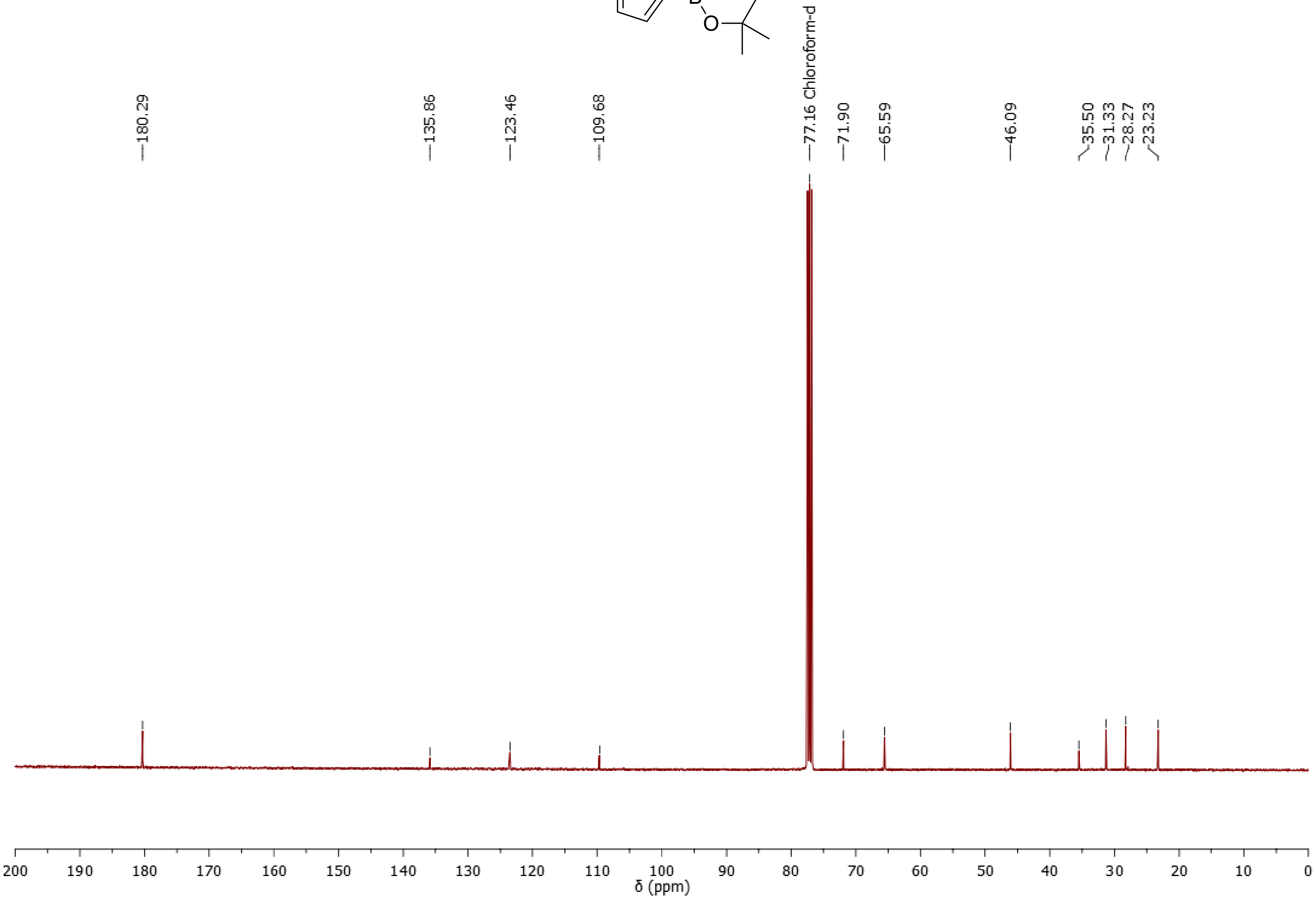

^{1}H 400MHz, $CDCl_3$

4bc

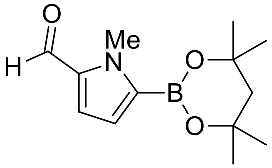

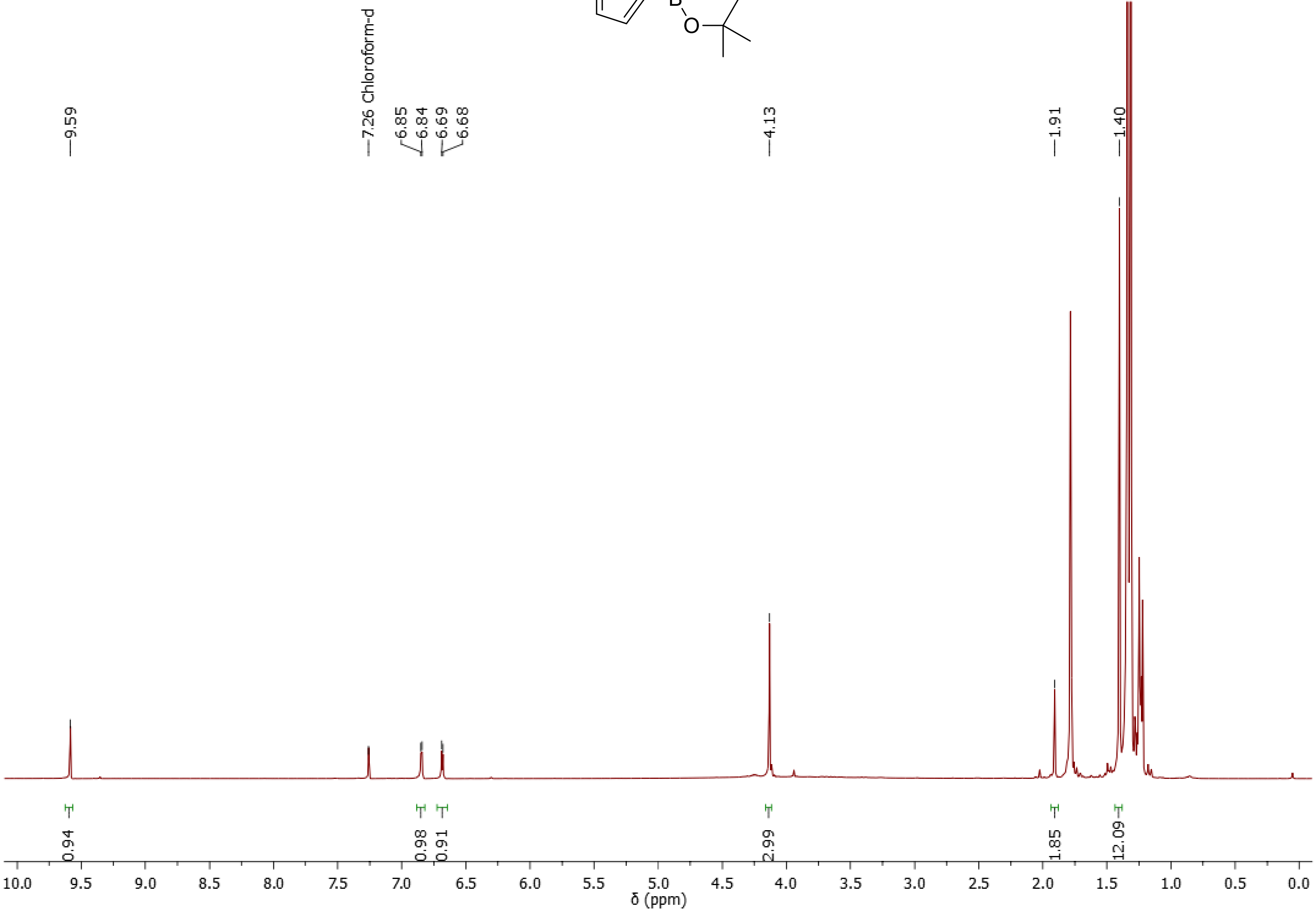

^{13}C 101MHz

4bc

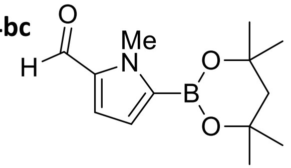

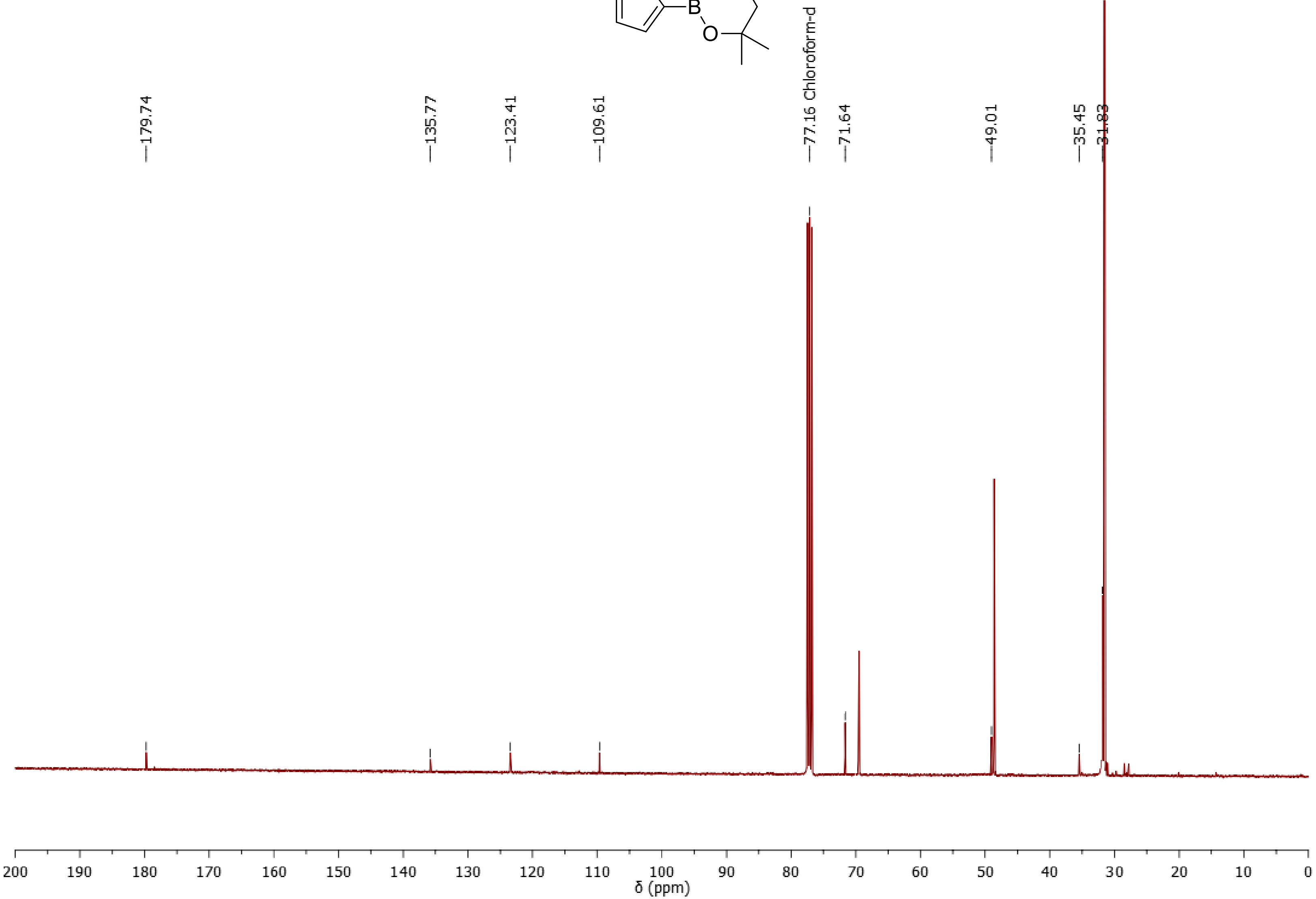

^{1}H 400MHz, $CDCl_3$

4bd

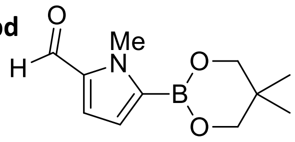

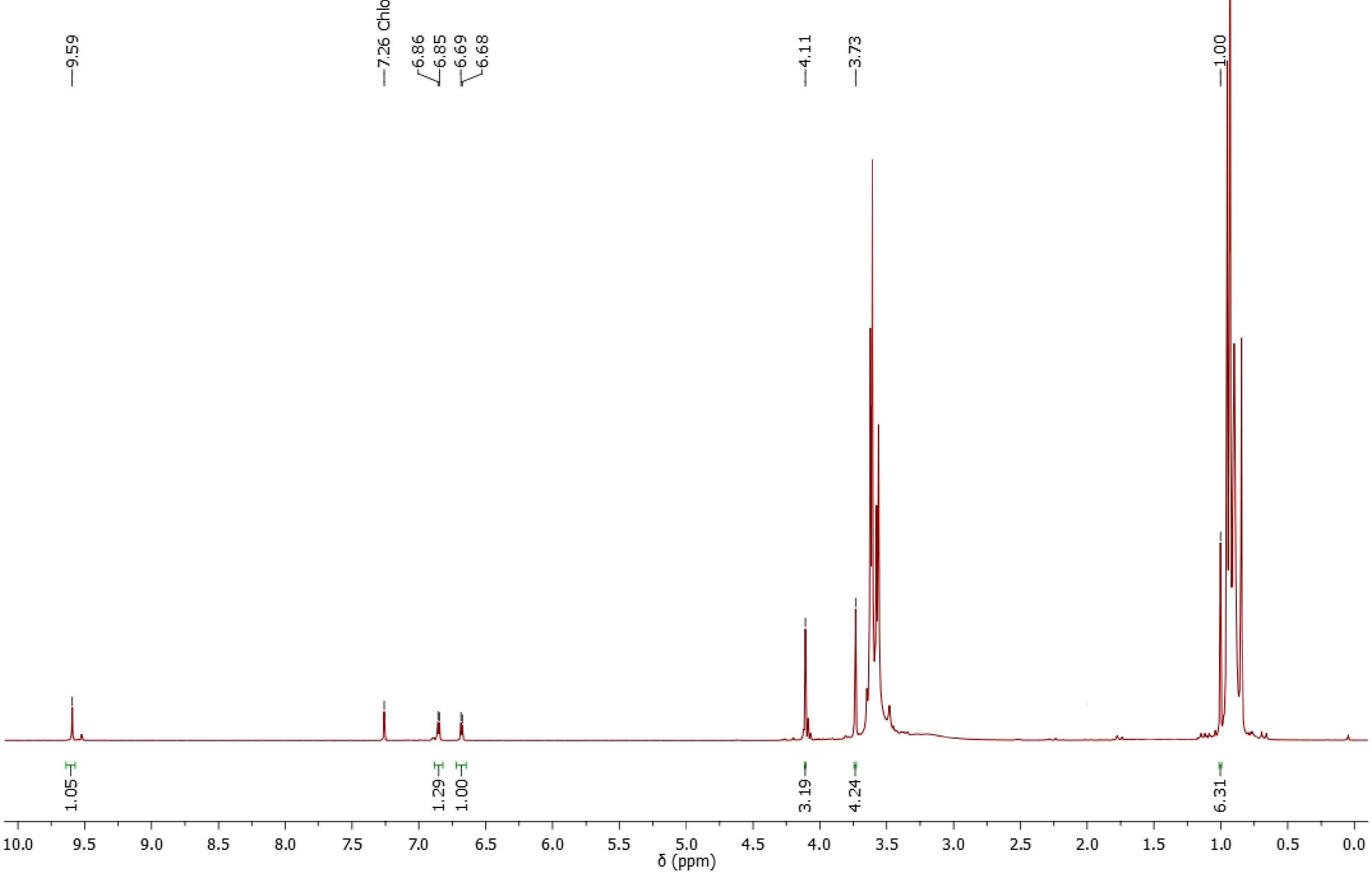

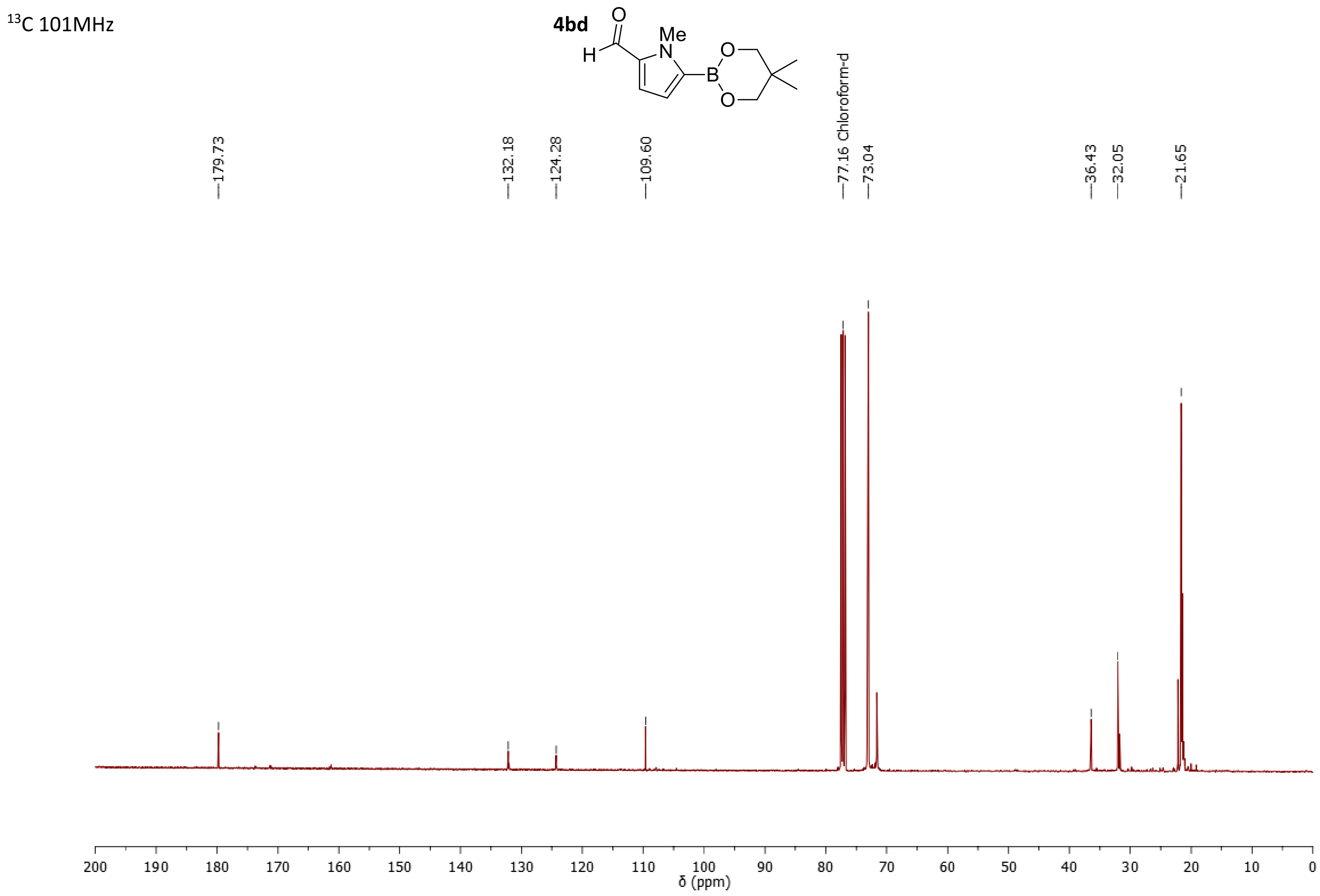

13C 101MHz
4bd
O
H
Me
N
B
O
O
179.73
132.18
124.28
109.60
77.16 Chloroform-d
73.04
36.43
32.05
21.65
200
190
180
170
160
150
140
130
120
110
100
90
80
70
60
50
40
30
20
10
0
δ (ppm)

^{1}H 400MHz, $CDCl_3$

4ca

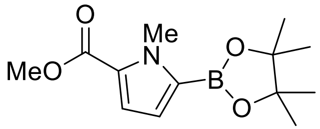

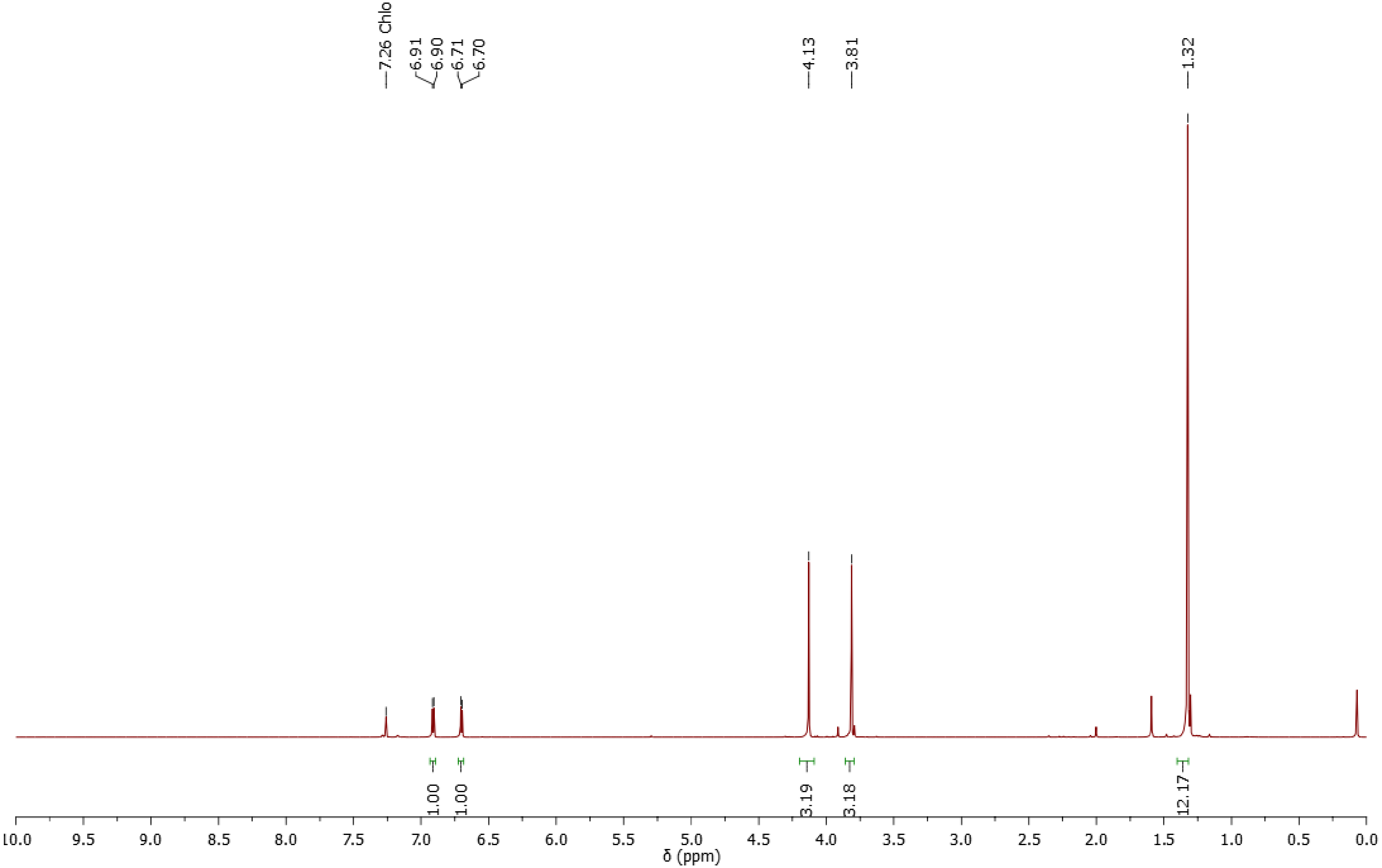

^{13}C 101MHz, $CDCl_3$

4ca

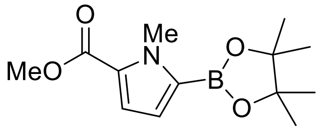

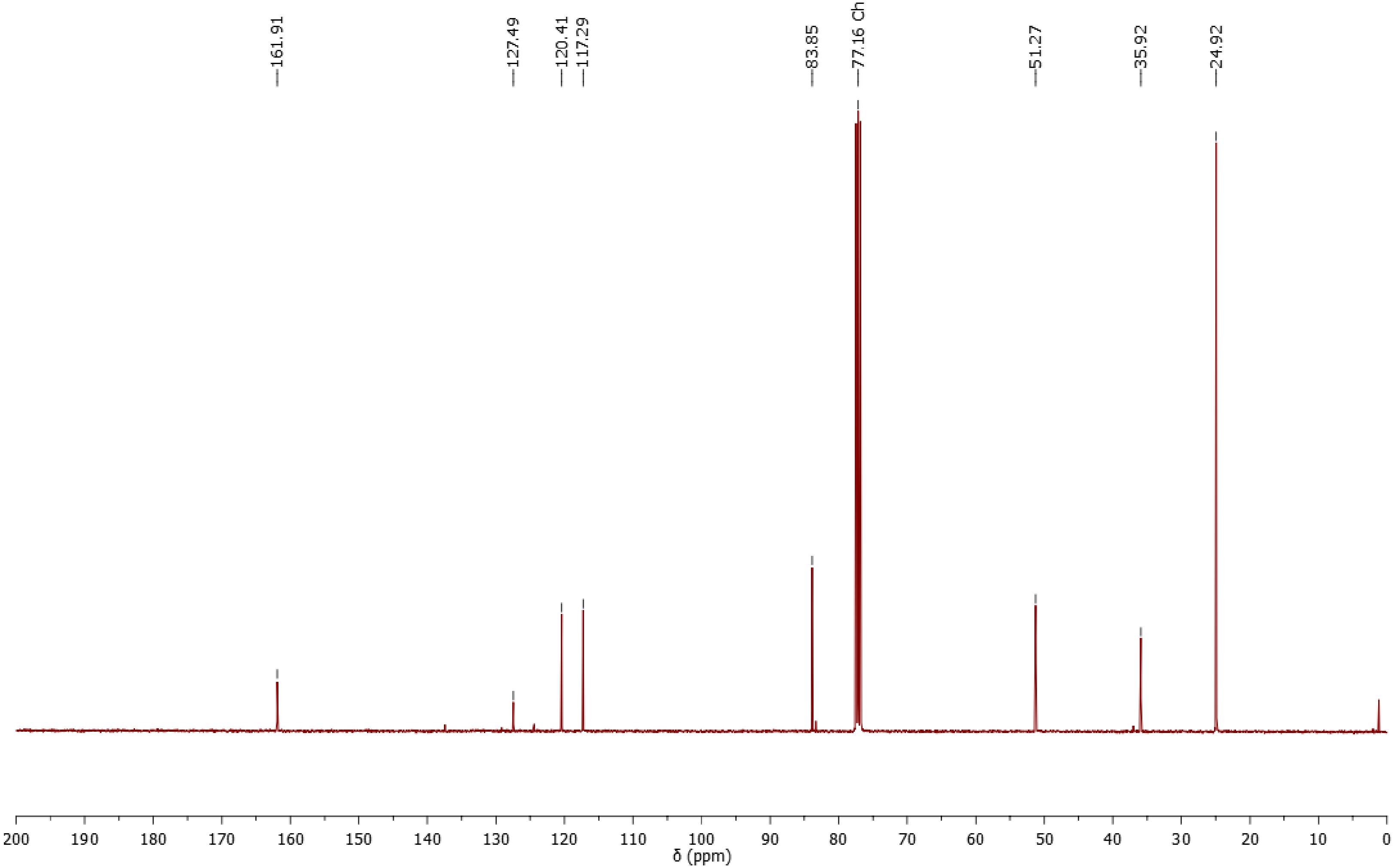

^{1}H 400MHz, $CDCl_3$

4cb

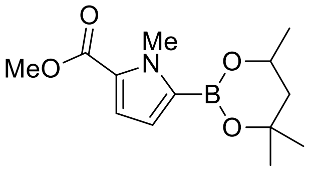

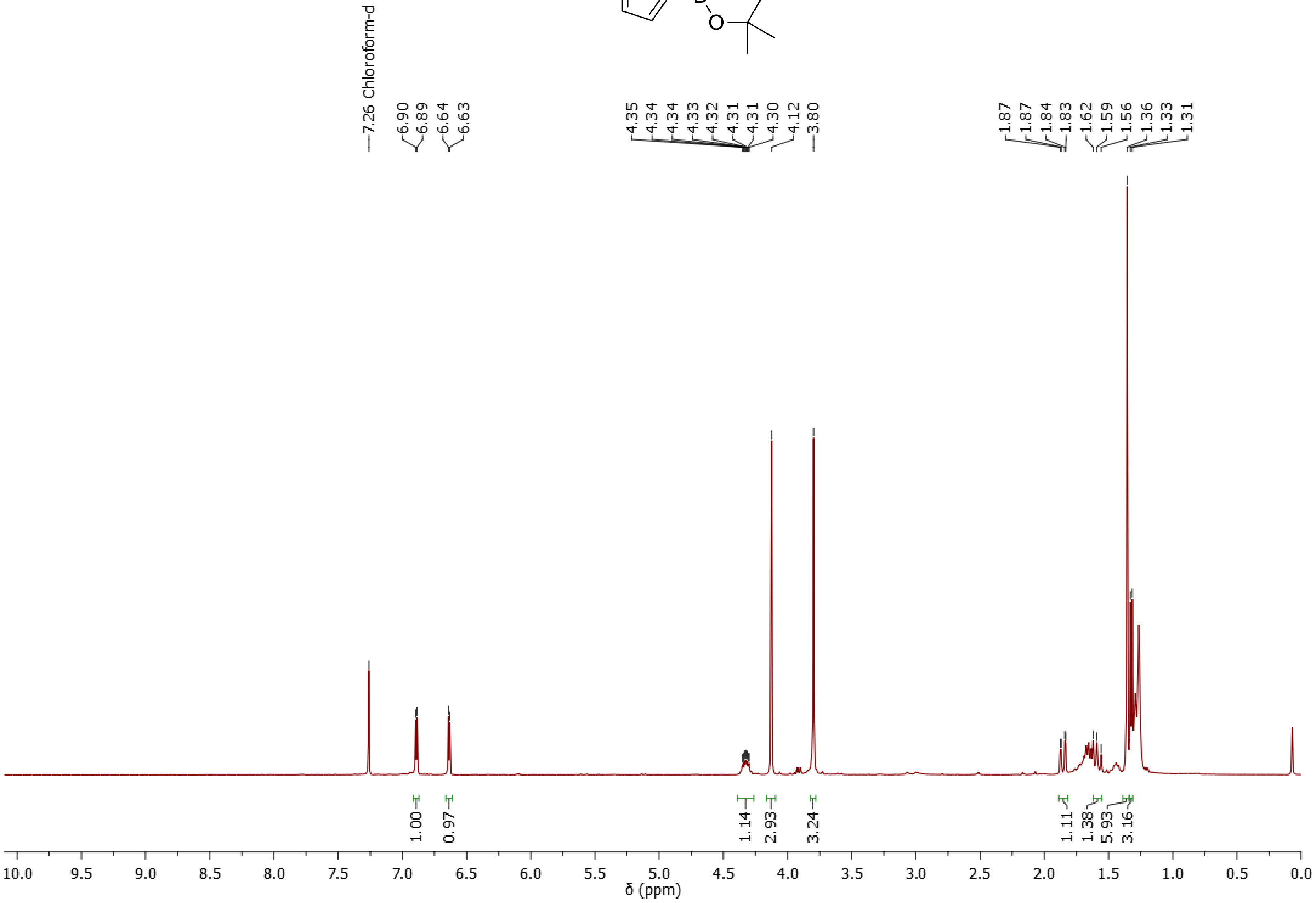

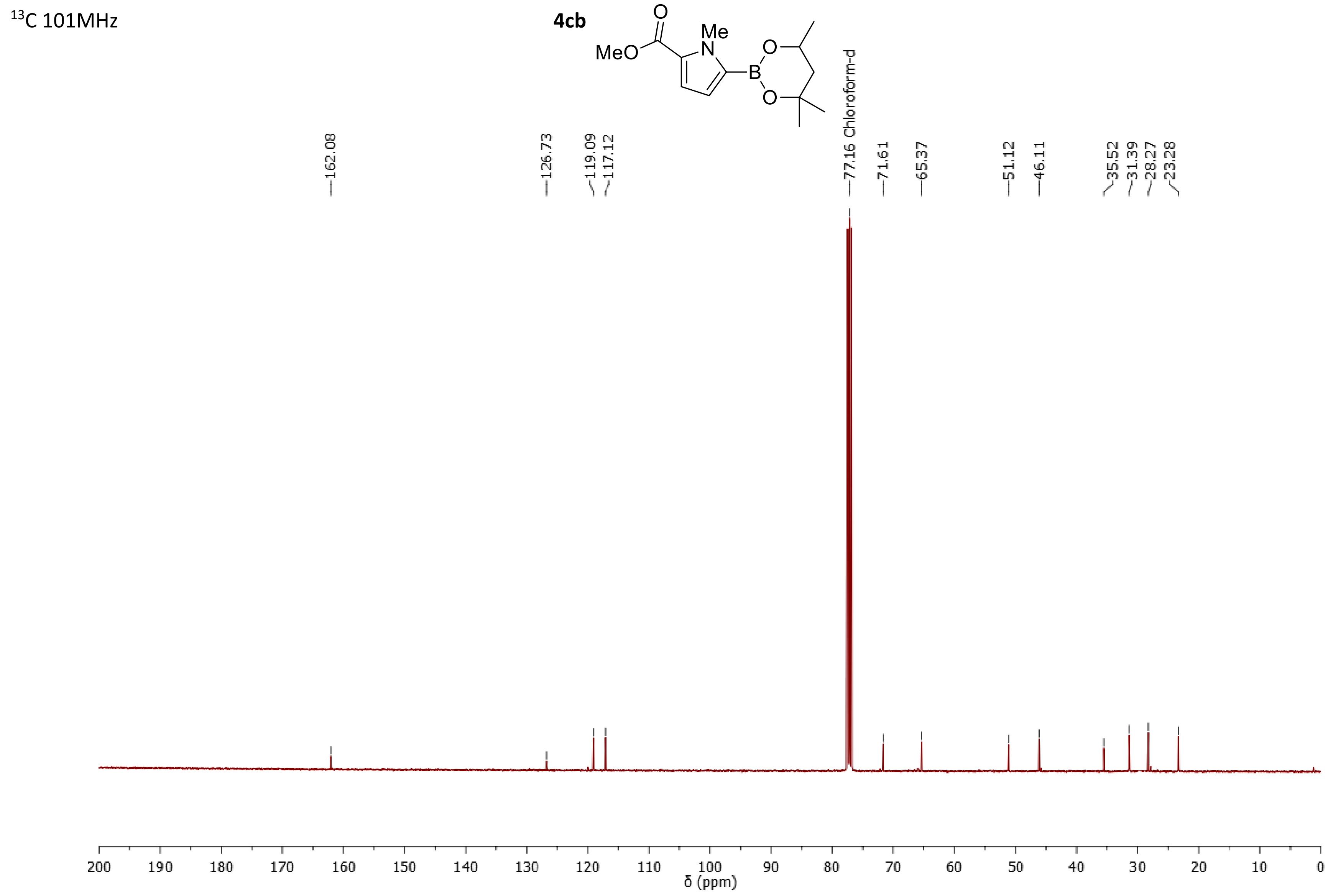
13C 101MHz
4cb
MeO
O
N
Me
B
O
O
162.08
126.73
119.09
117.12
77.16 Chloroform-d
71.61
65.37
51.12
46.11
35.52
31.39
28.27
23.28
200
190
180
170
160
150
140
130
120
110
100
90
80
70
60
50
40
30
20
10
0
δ (ppm)

^{1}H 400MHz, $CDCl_3$

4cc

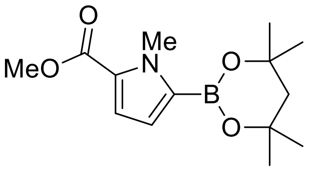

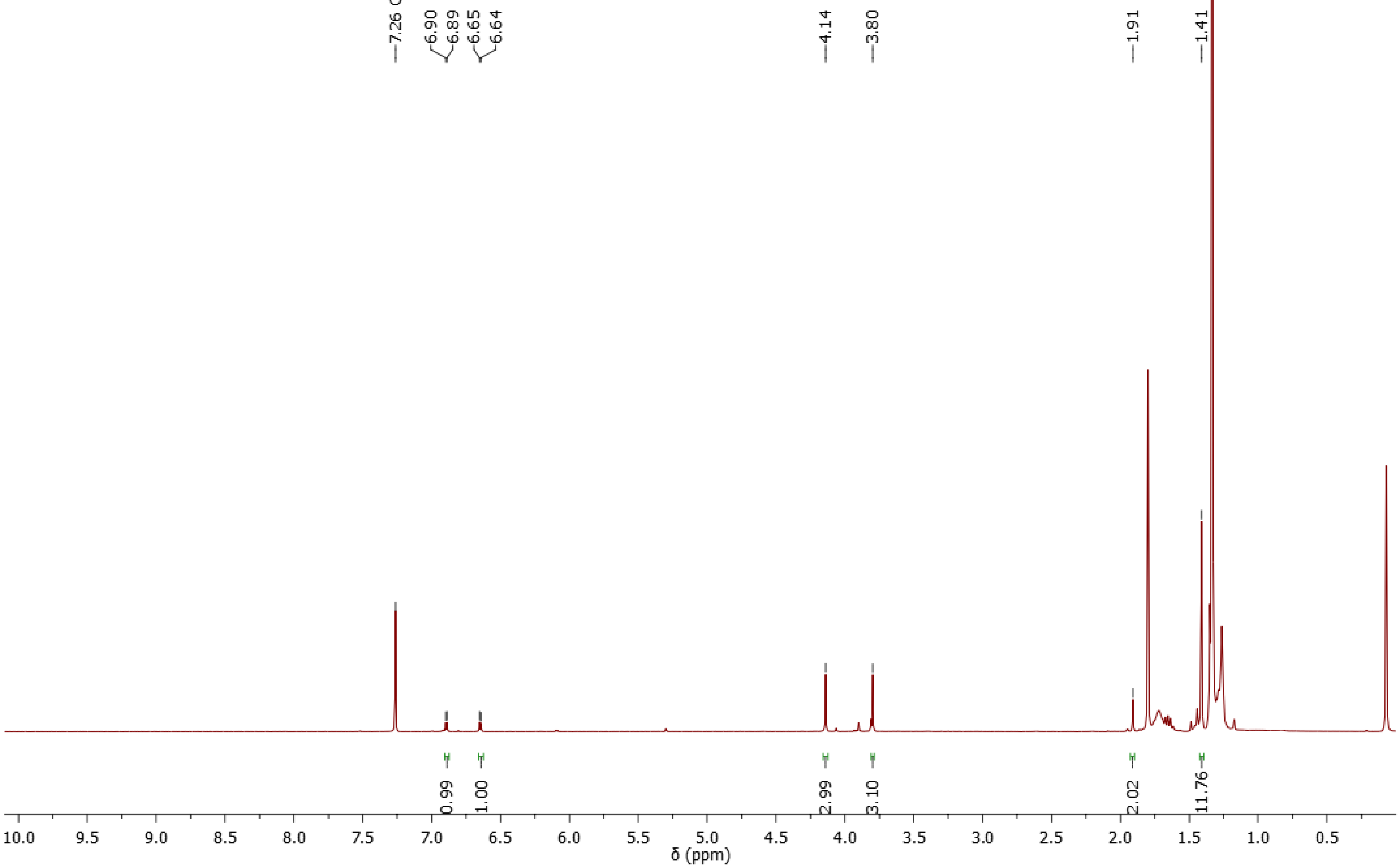

^{13}C 101MHz

4cc

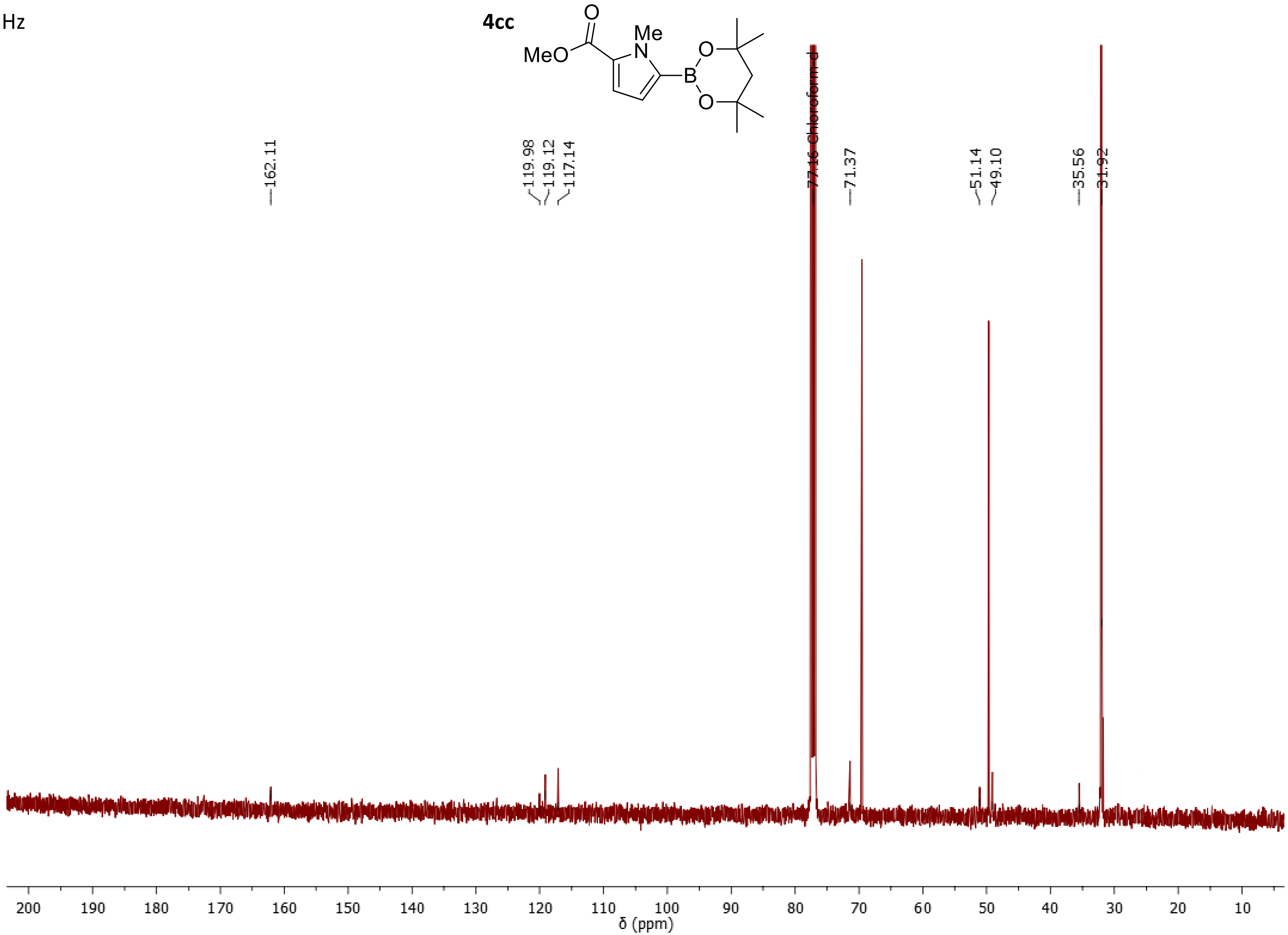

^{1}H 400MHz, $CDCl_3$

4cd

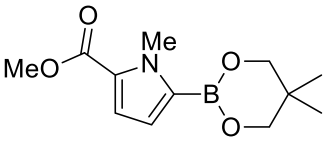

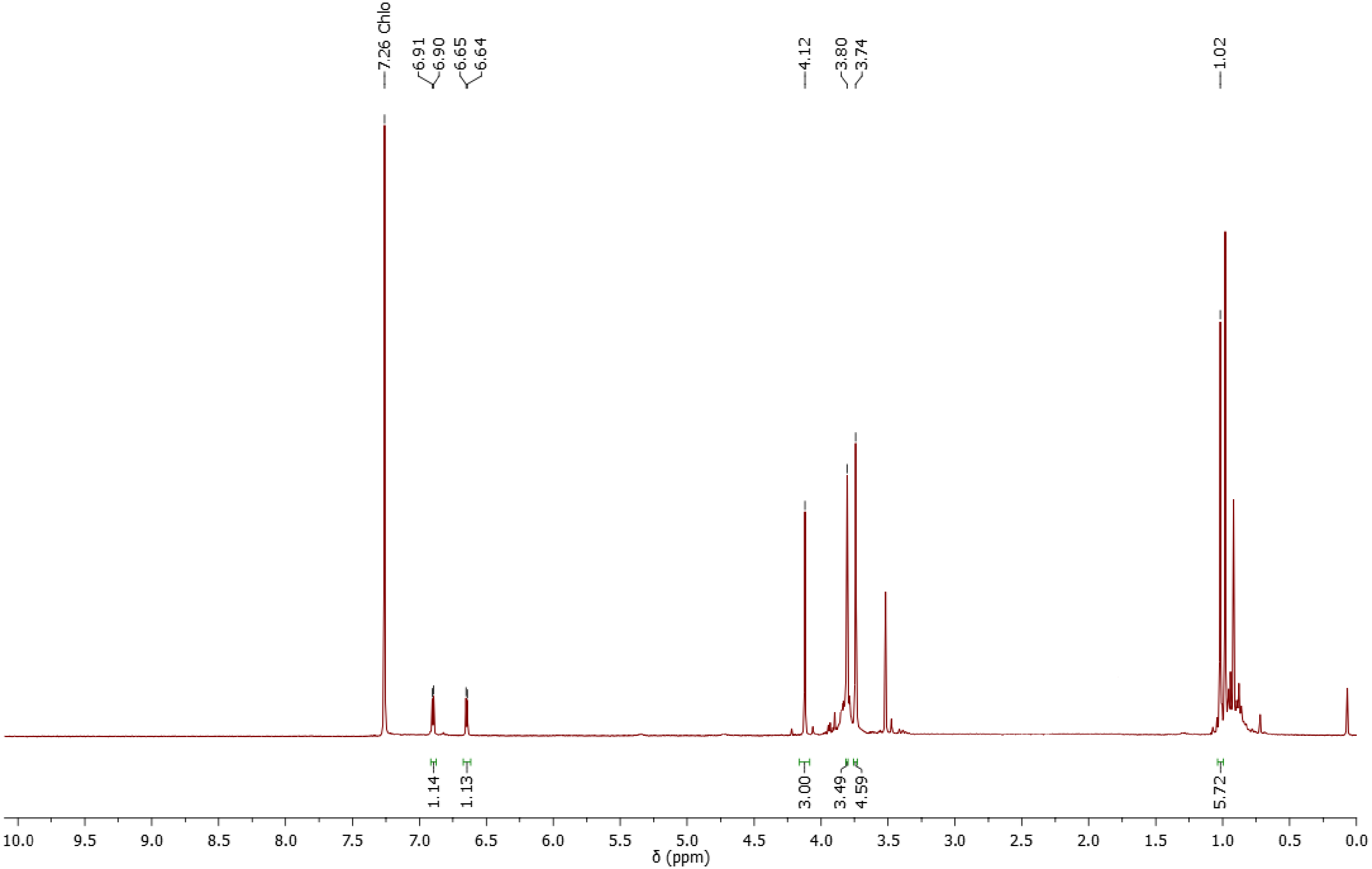

^{13}C 101MHz

4cd

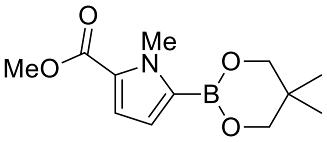

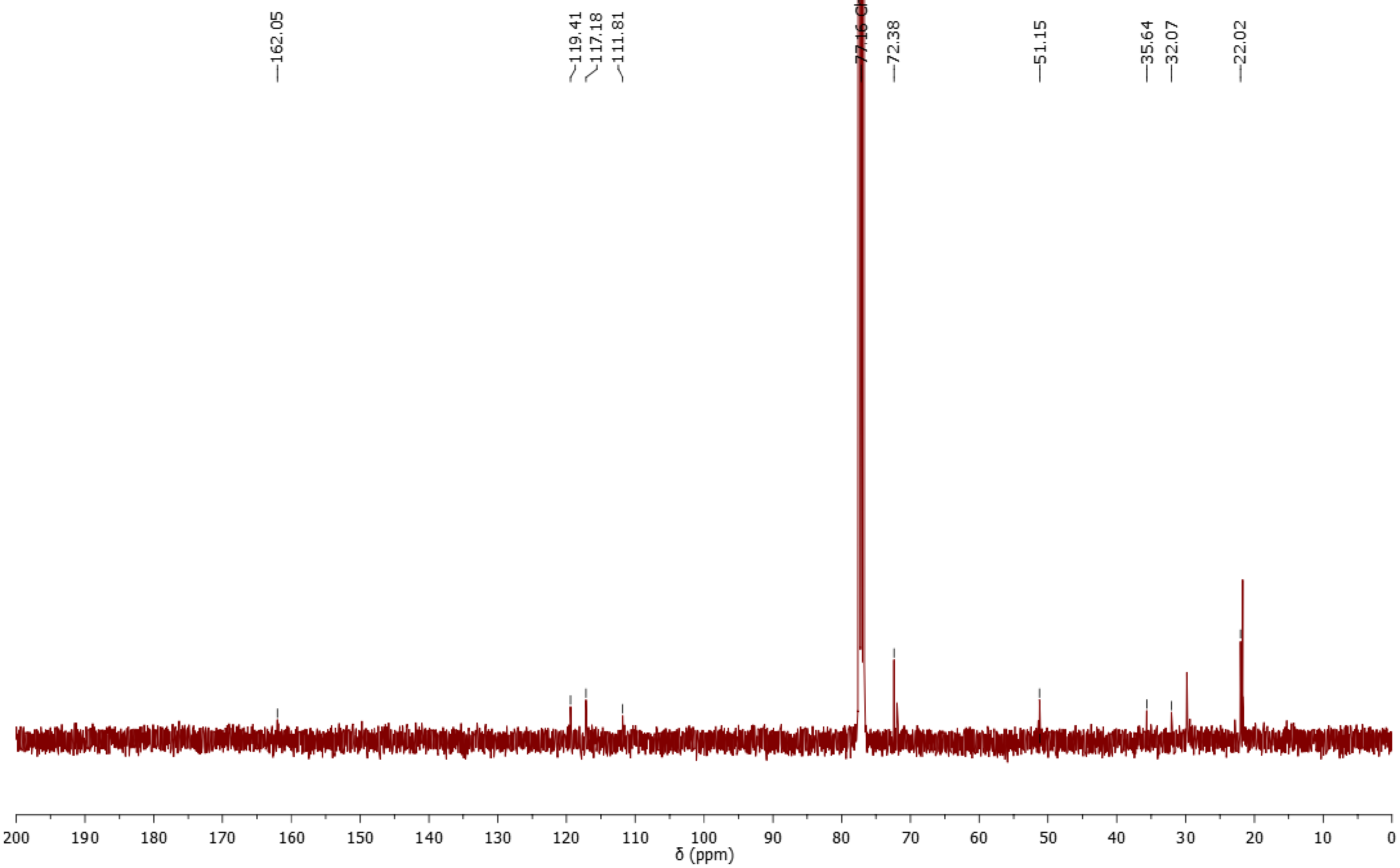

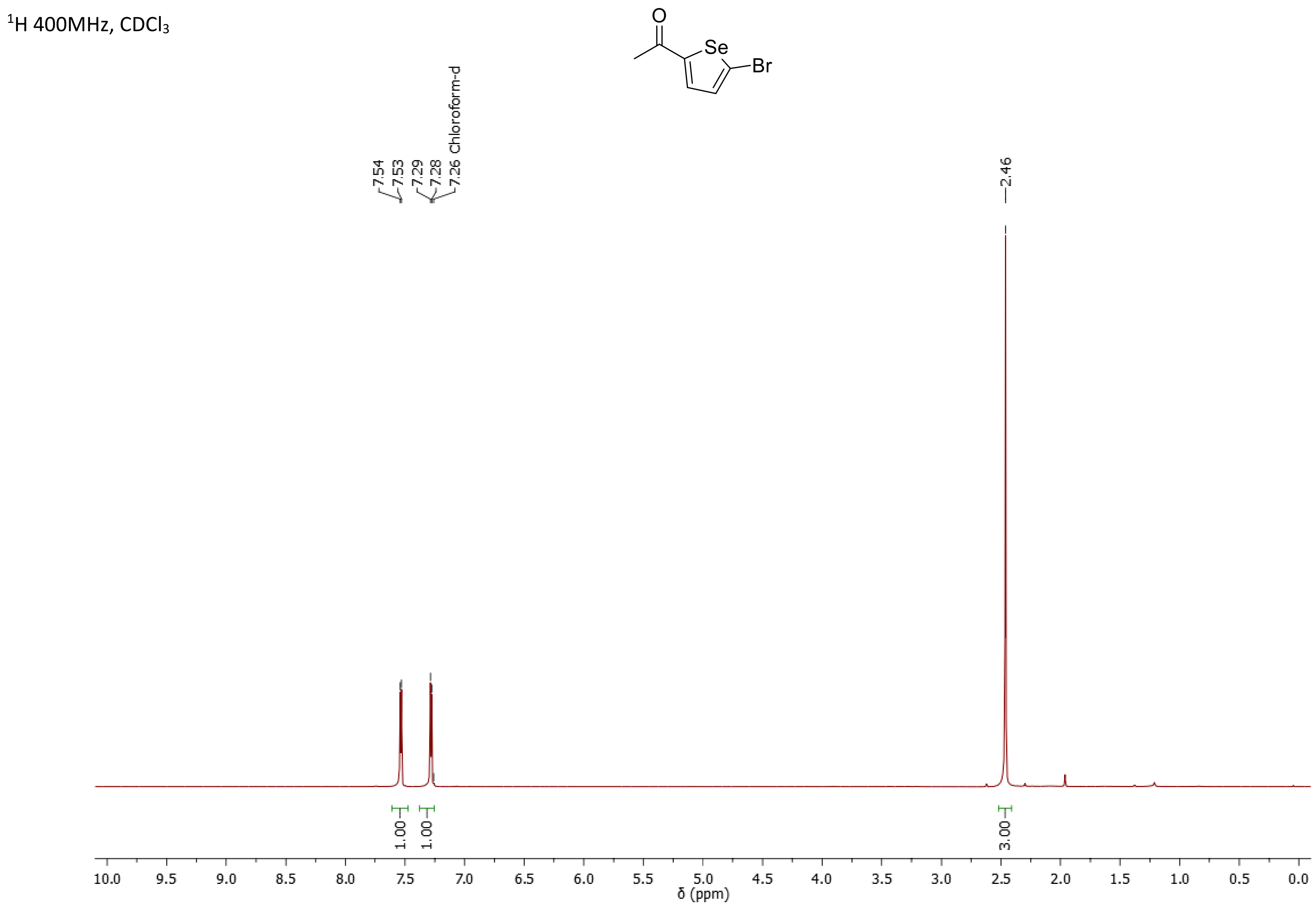
1H 400MHz, CDCl3
O
Se
Br
7.54
7.53
7.29
7.28
7.26 Chloroform-d
2.46
1.00
1.00
3.00
10.0
9.5
9.0
8.5
8.0
7.5
7.0
6.5
6.0
5.5
5.0
4.5
4.0
3.5
3.0
2.5
2.0
1.5
1.0
0.5
0.0
δ (ppm)

^{13}C 101MHz, $CDCl_3$

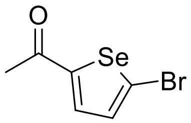

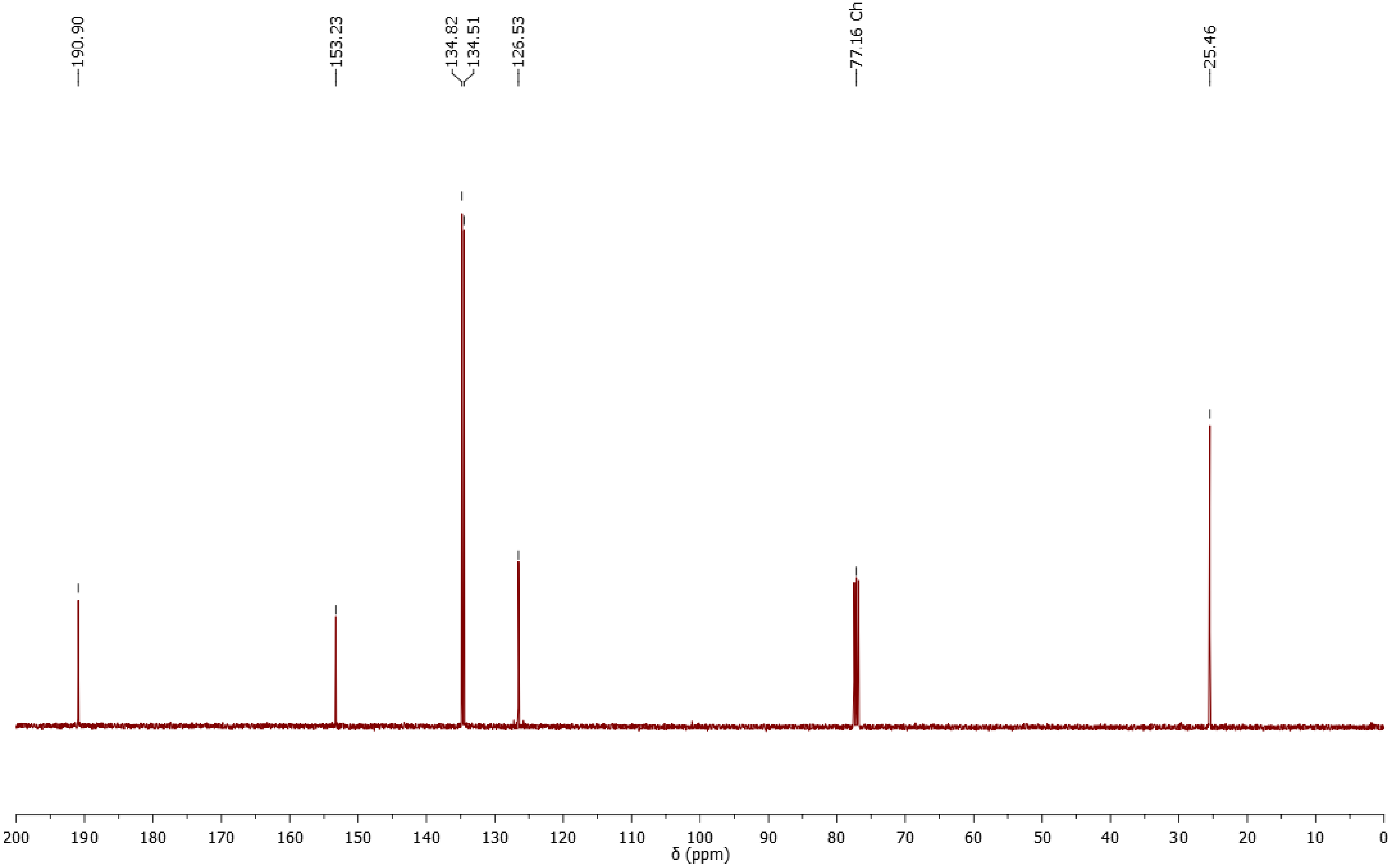

^{1}H 400MHz, $CDCl_3$

5aa

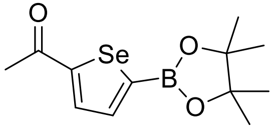

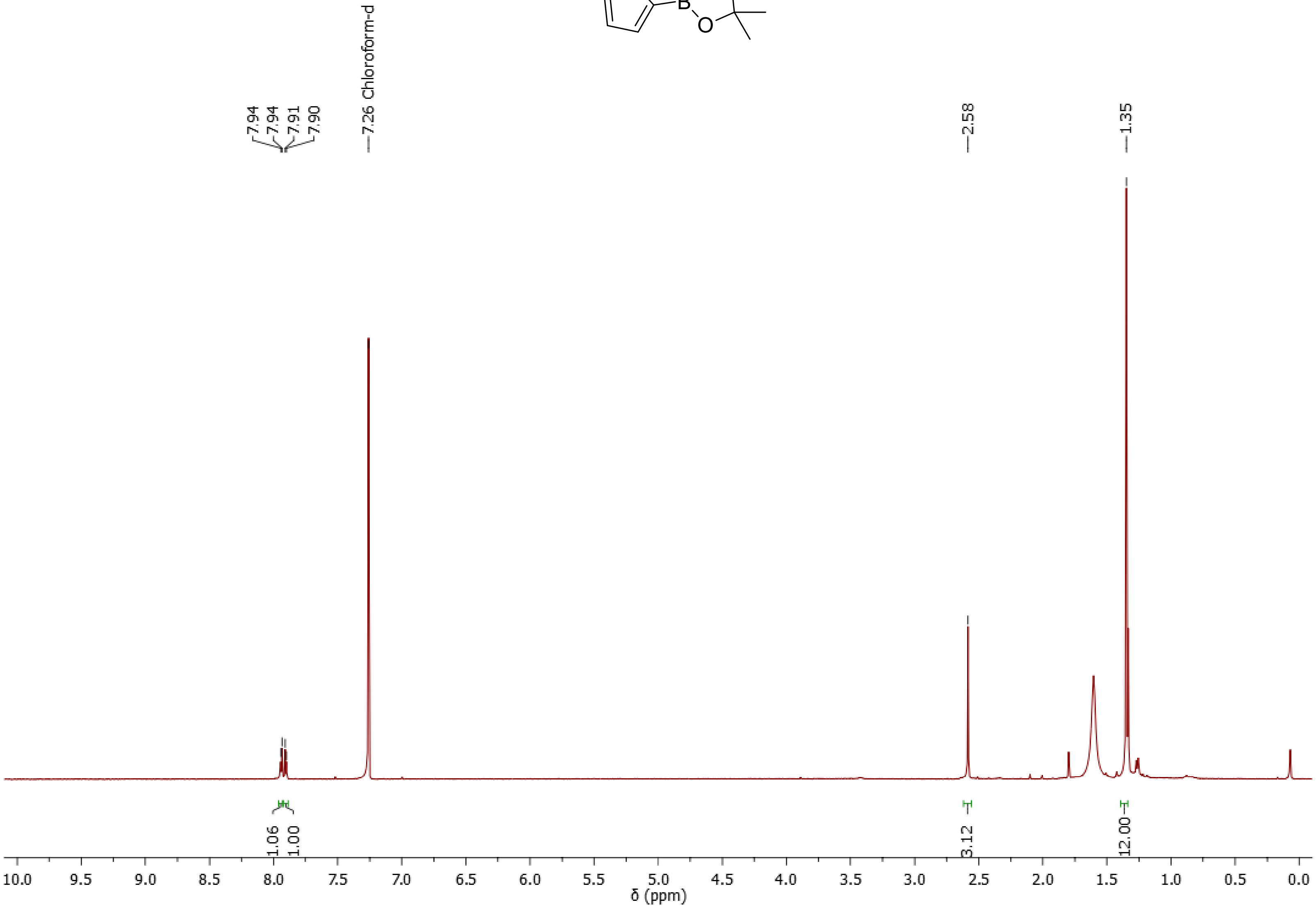

^{13}C 101MHz, $CDCl_3$

5aa

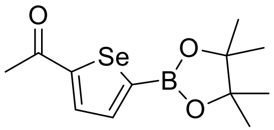

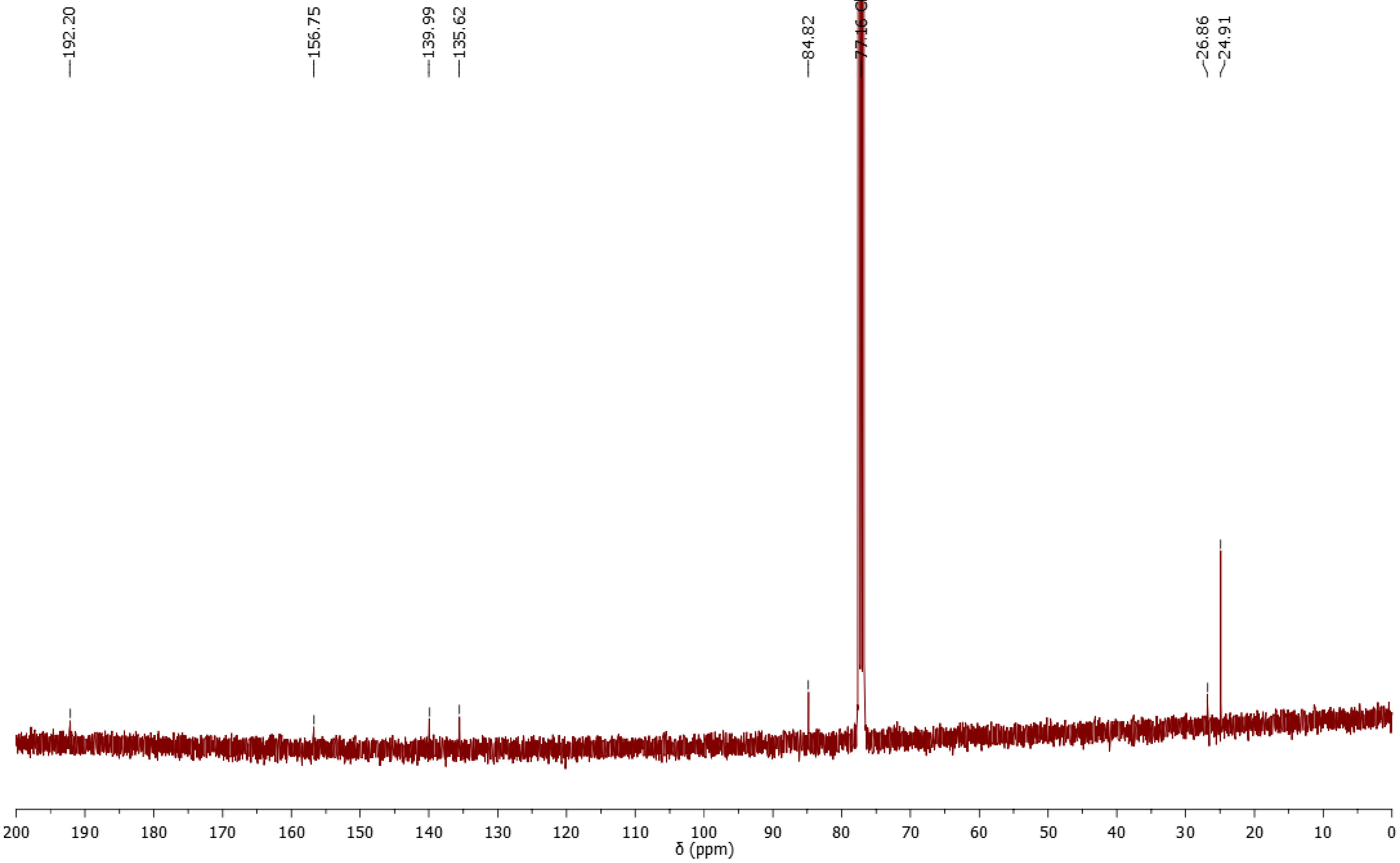

^{1}H 400MHz, $CDCl_3$

5ab

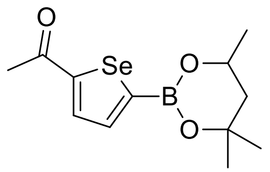

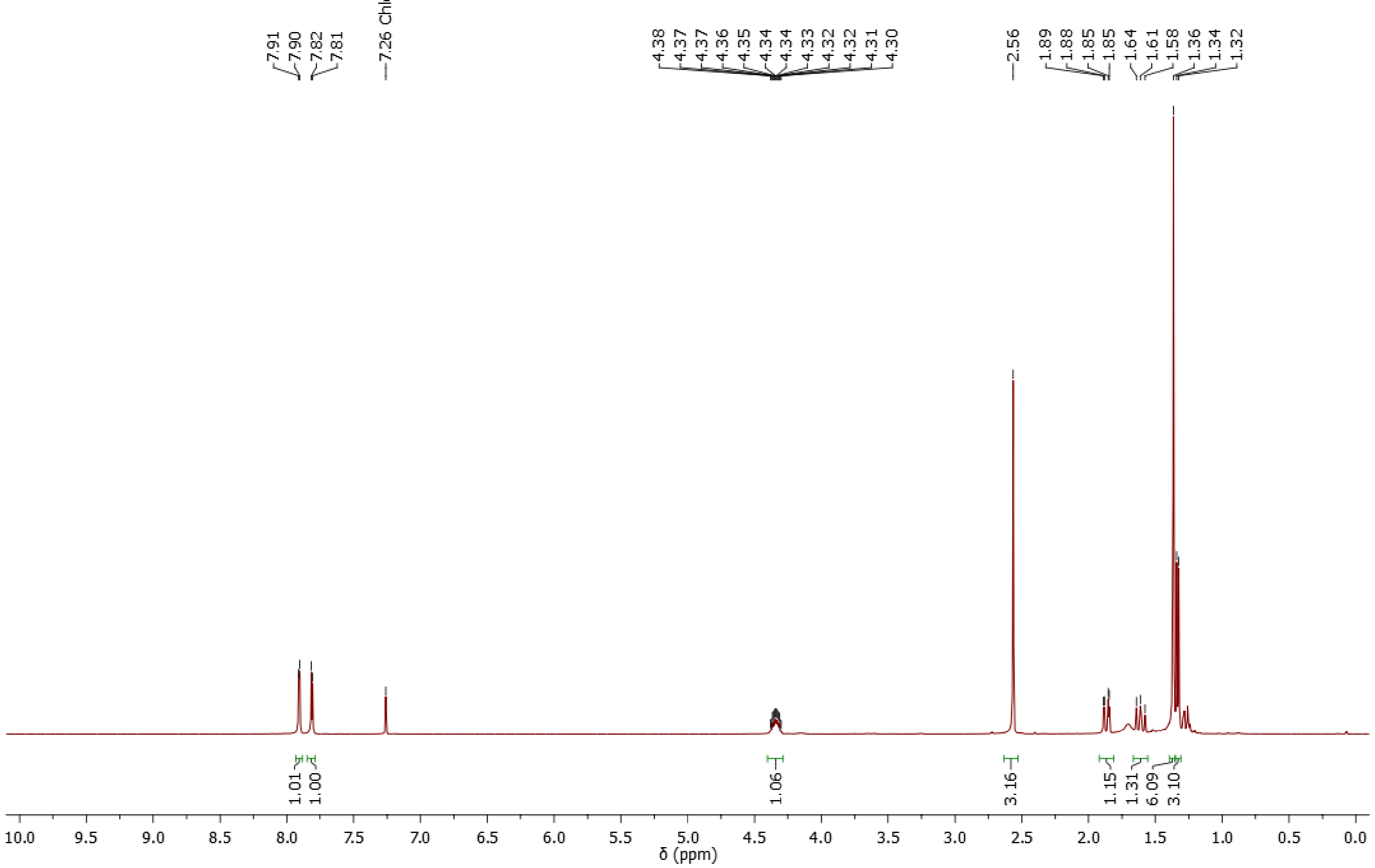

^{13}C 101MHz, $CDCl_3$

5ab

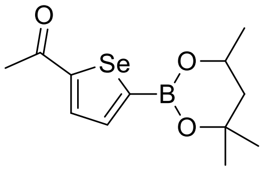

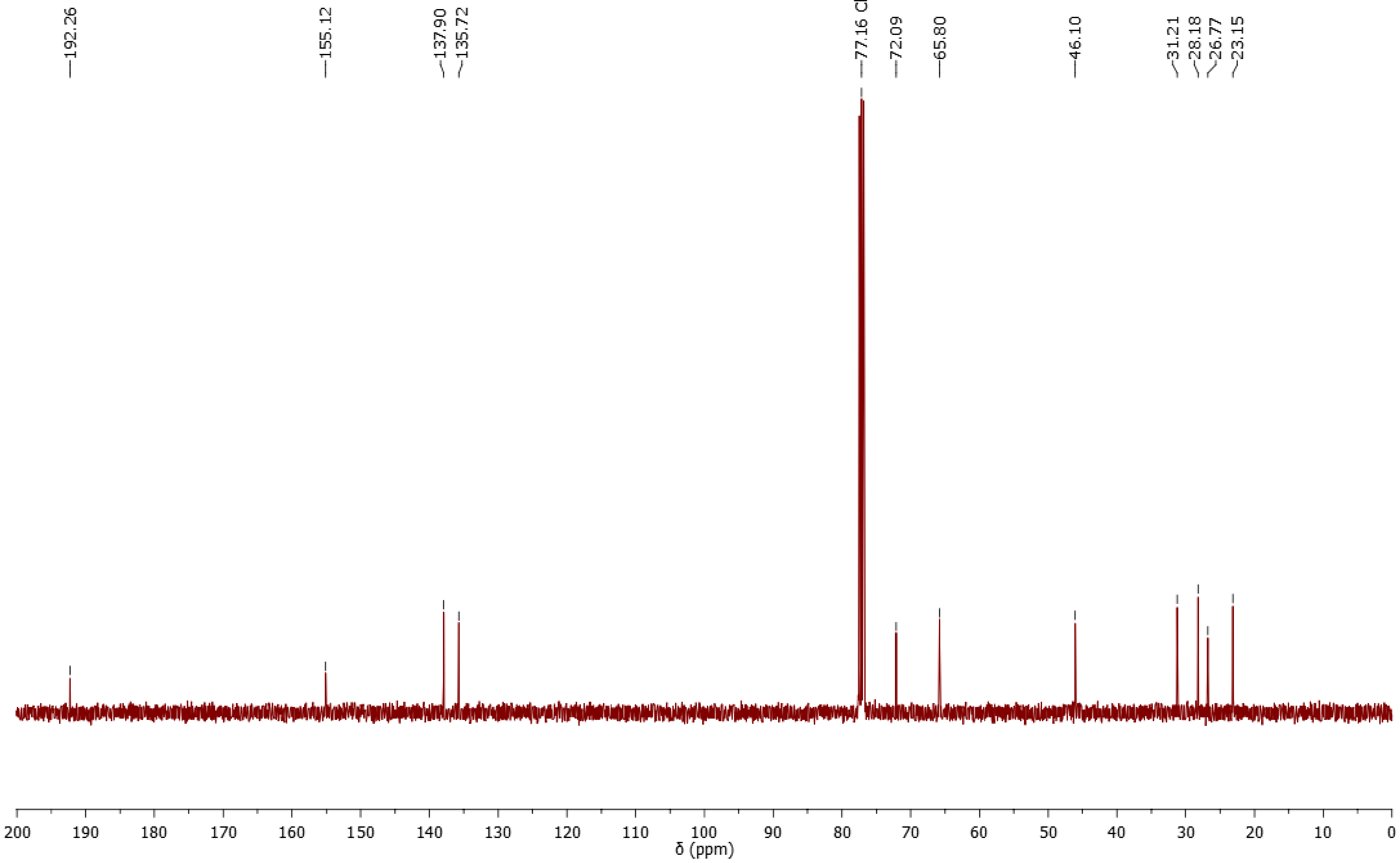

^{1}H 400MHz, $CDCl_3$

5ac

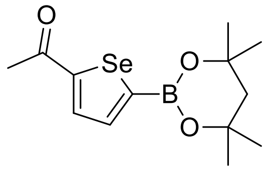

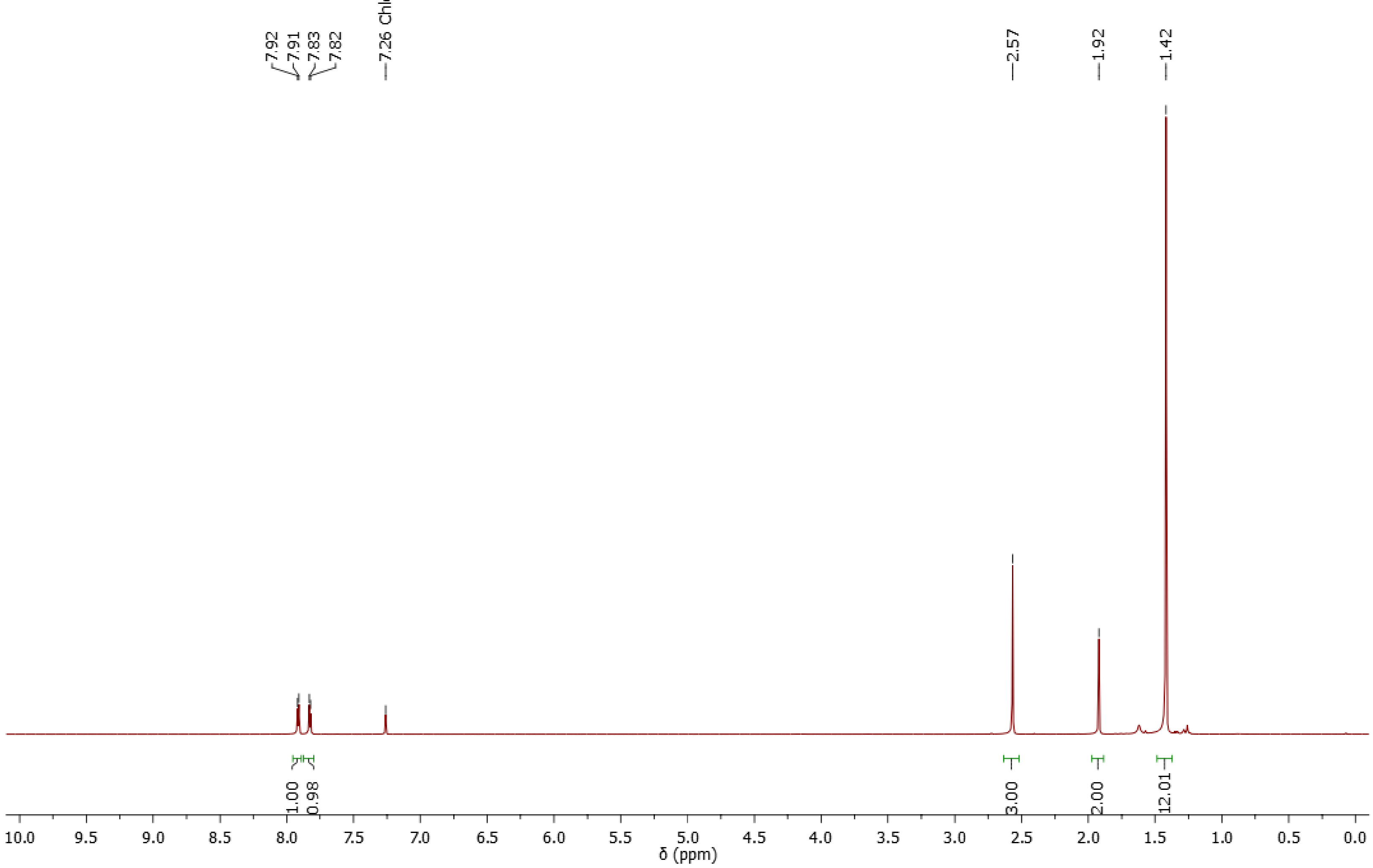

^{13}C 101MHz, $CDCl_3$

5ac

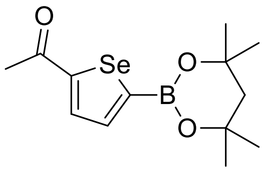

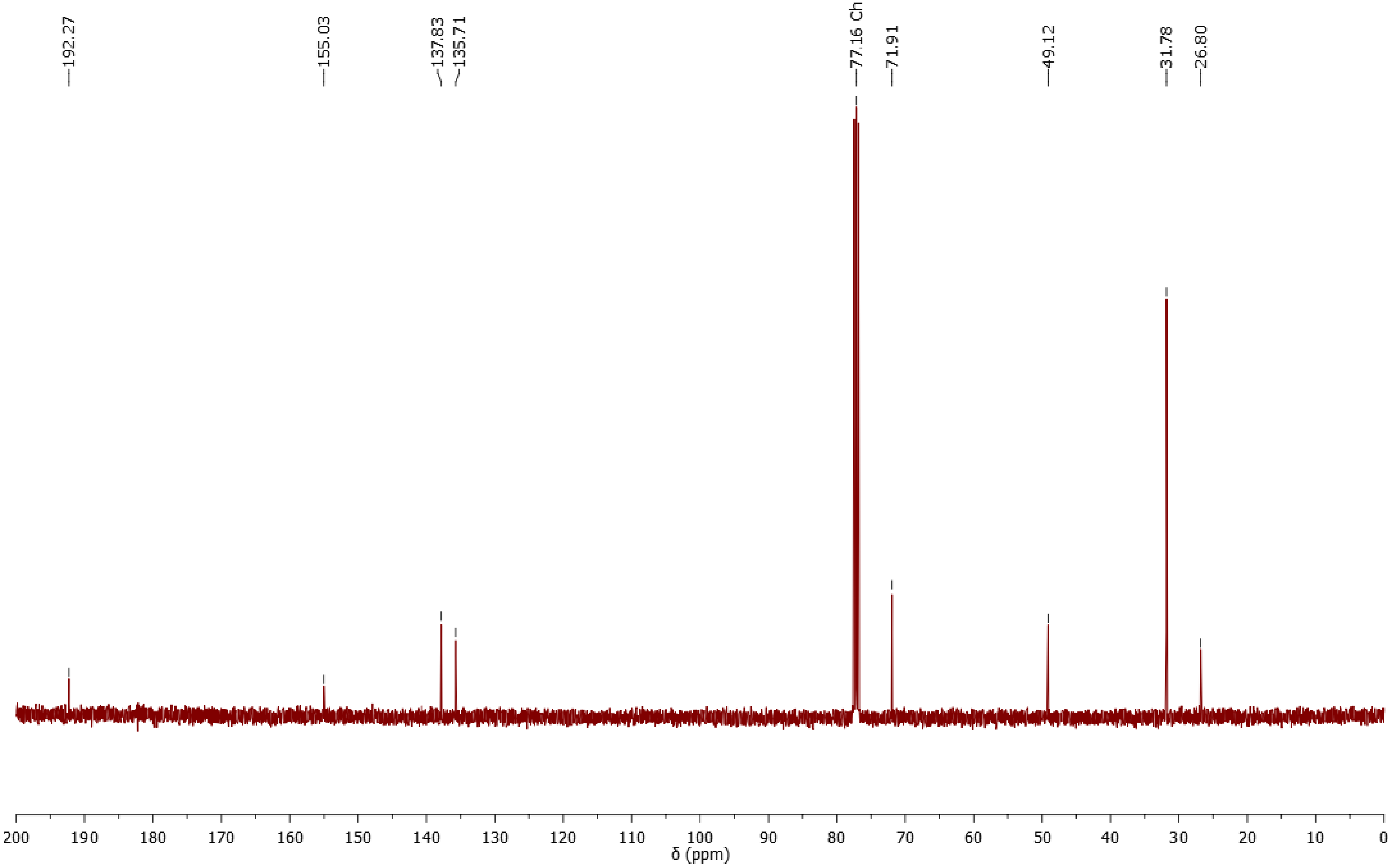

^{1}H 400MHz, $CDCl_3$

5ad

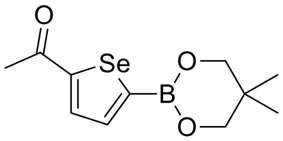

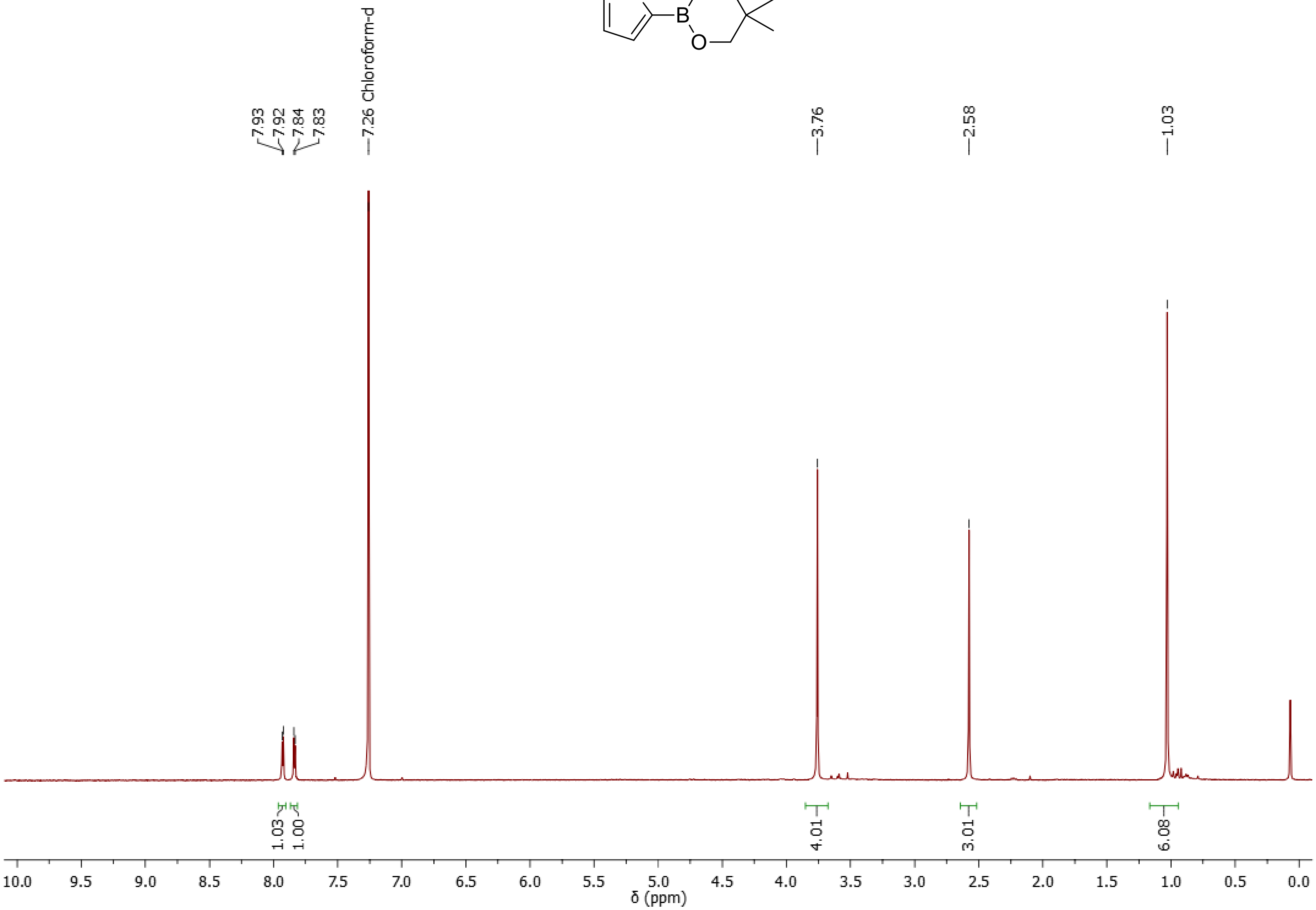

^{13}C 101MHz, $CDCl_3$

5ad

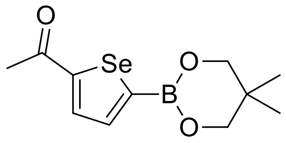

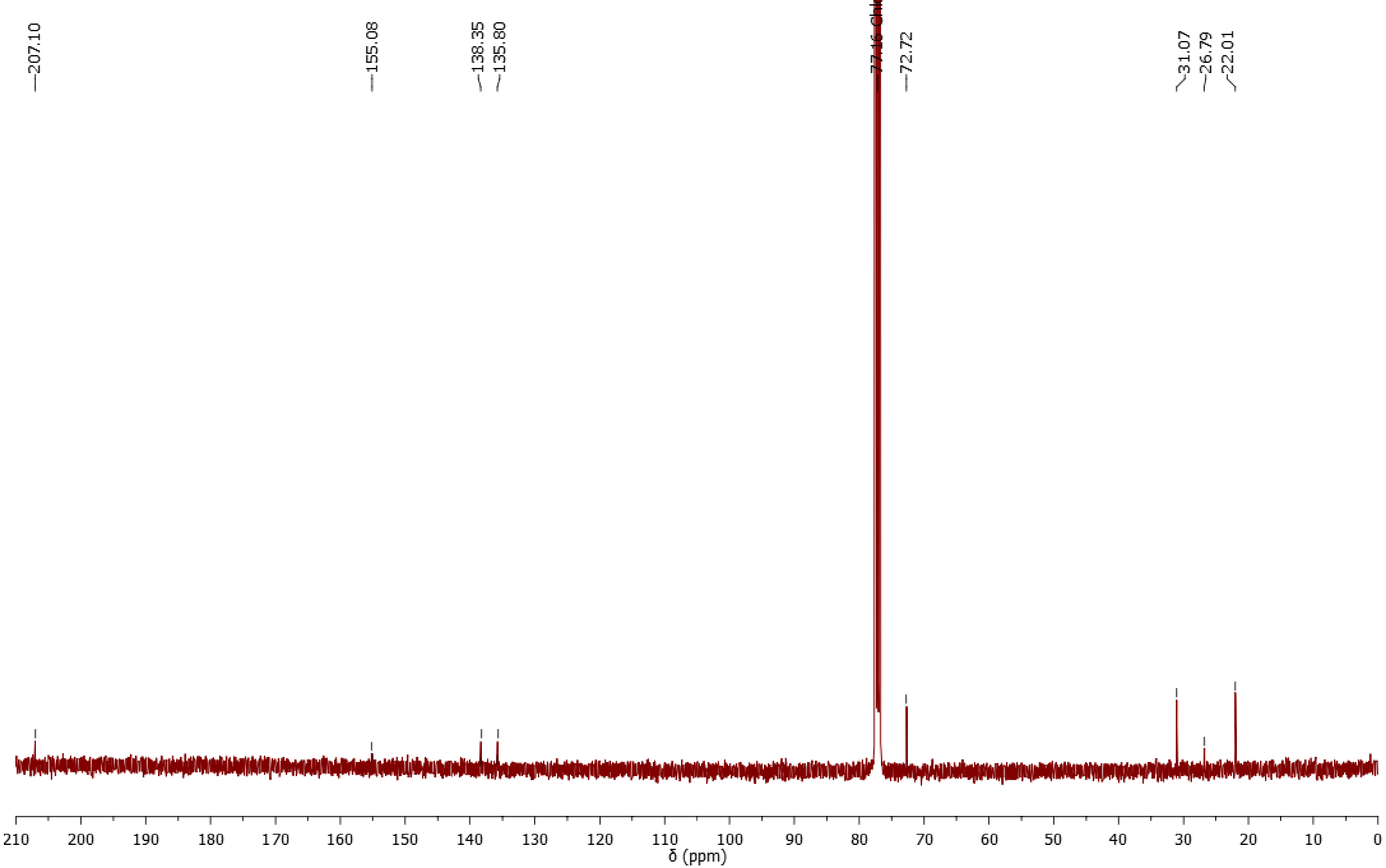

Thiophene thioether

^{1}H 400MHz, CDCl$_3$

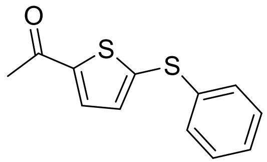

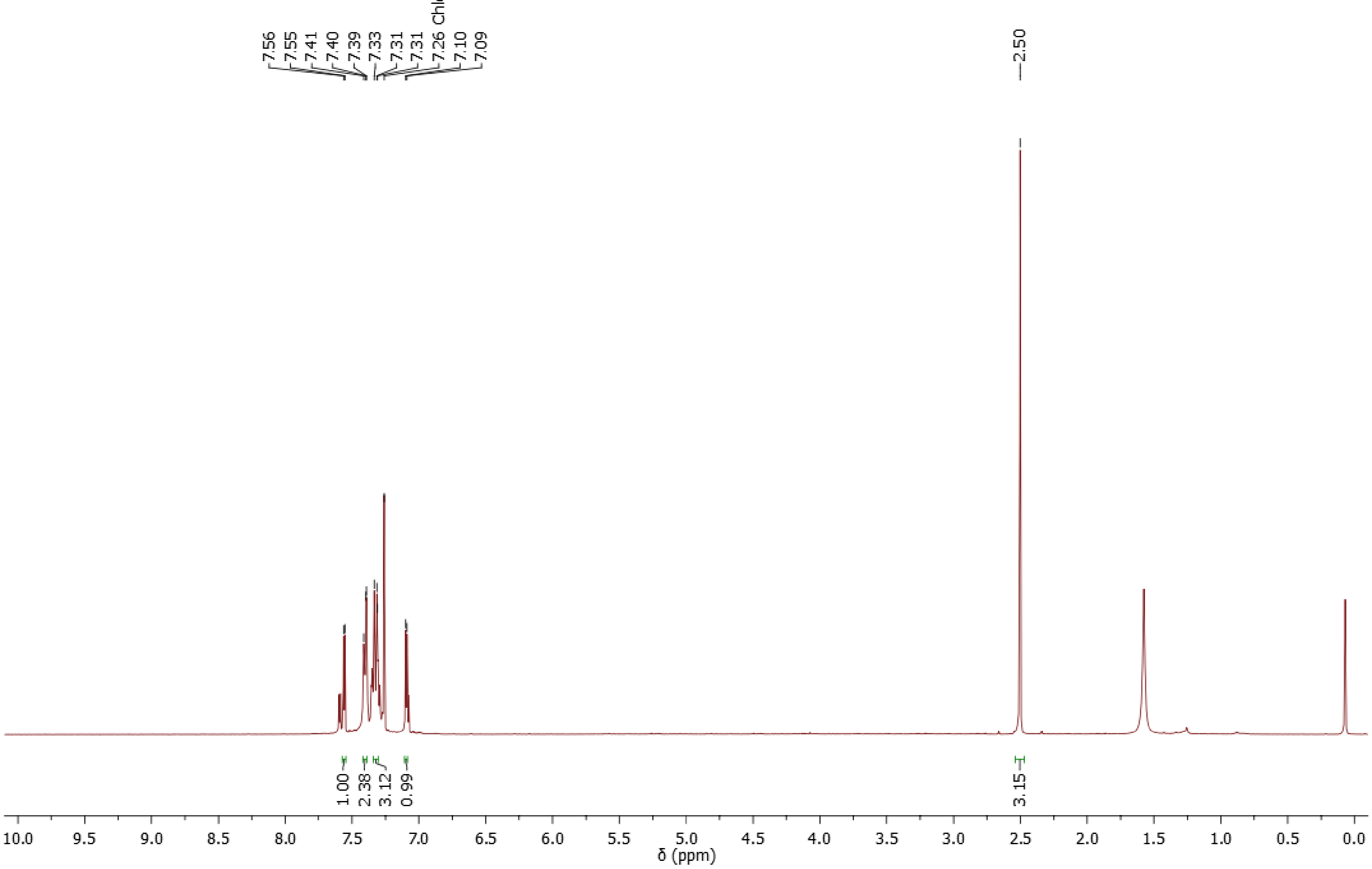

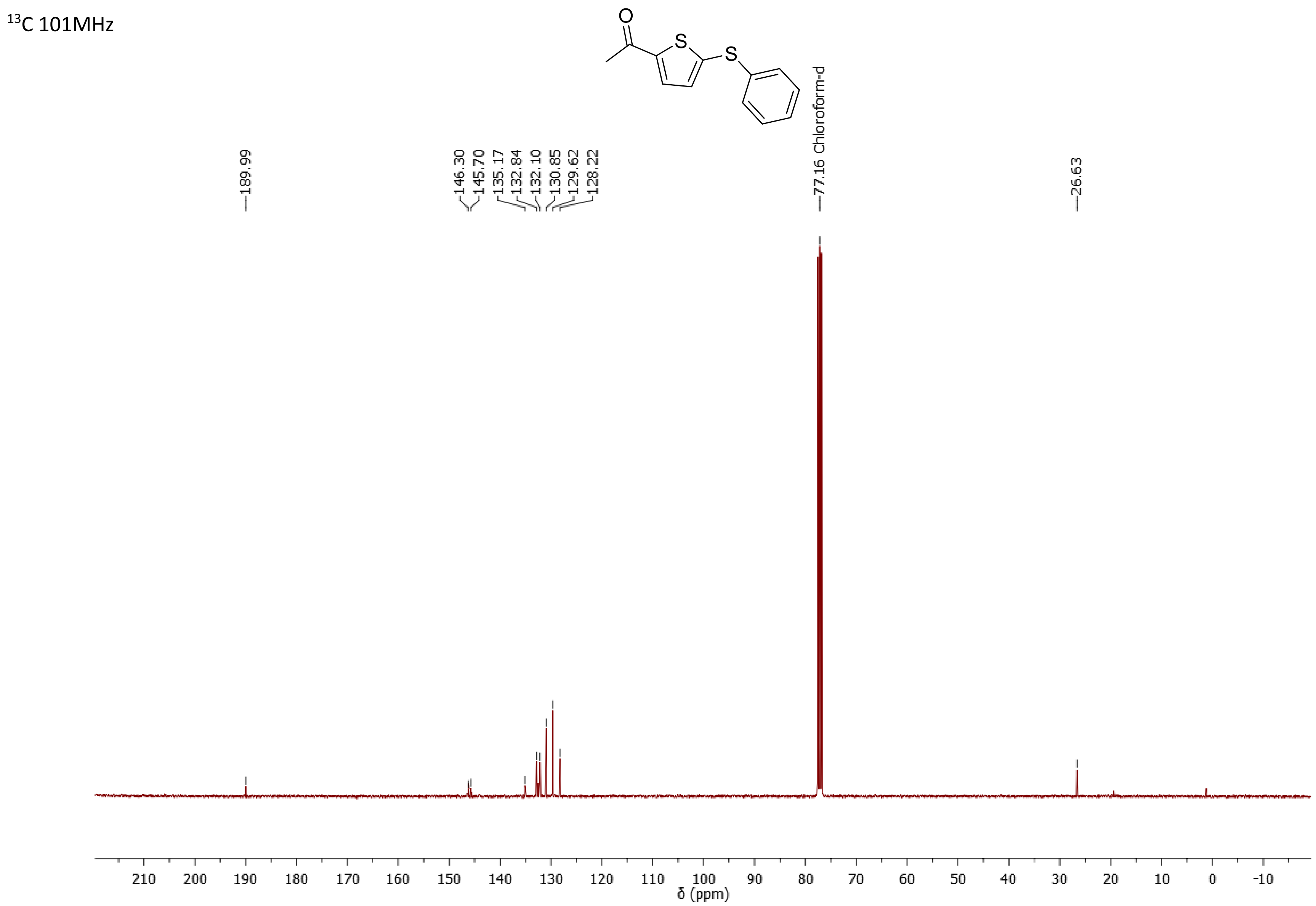

^{13}C 101MHz
O
S
S
189.99
146.30
145.70
135.17
132.84
132.10
130.85
129.62
128.22
77.16 Chloroform-d
26.63
210 200 190 180 170 160 150 140 130 120 110 100 90 80 70 60 50 40 30 20 10 0 -10
δ (ppm)

Furan thioether

^1H 400MHz, $CDCl_3$

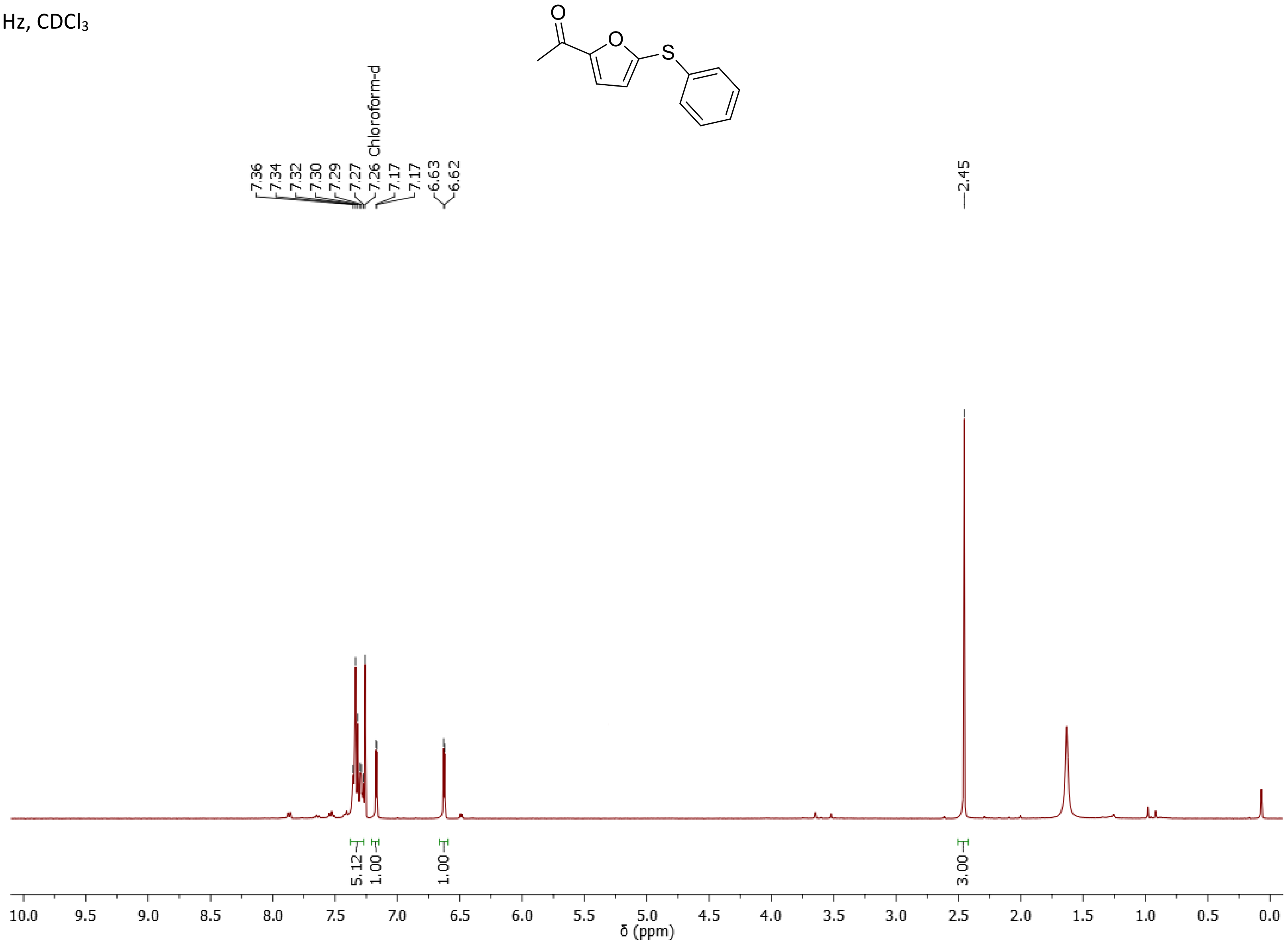

Furan thioether

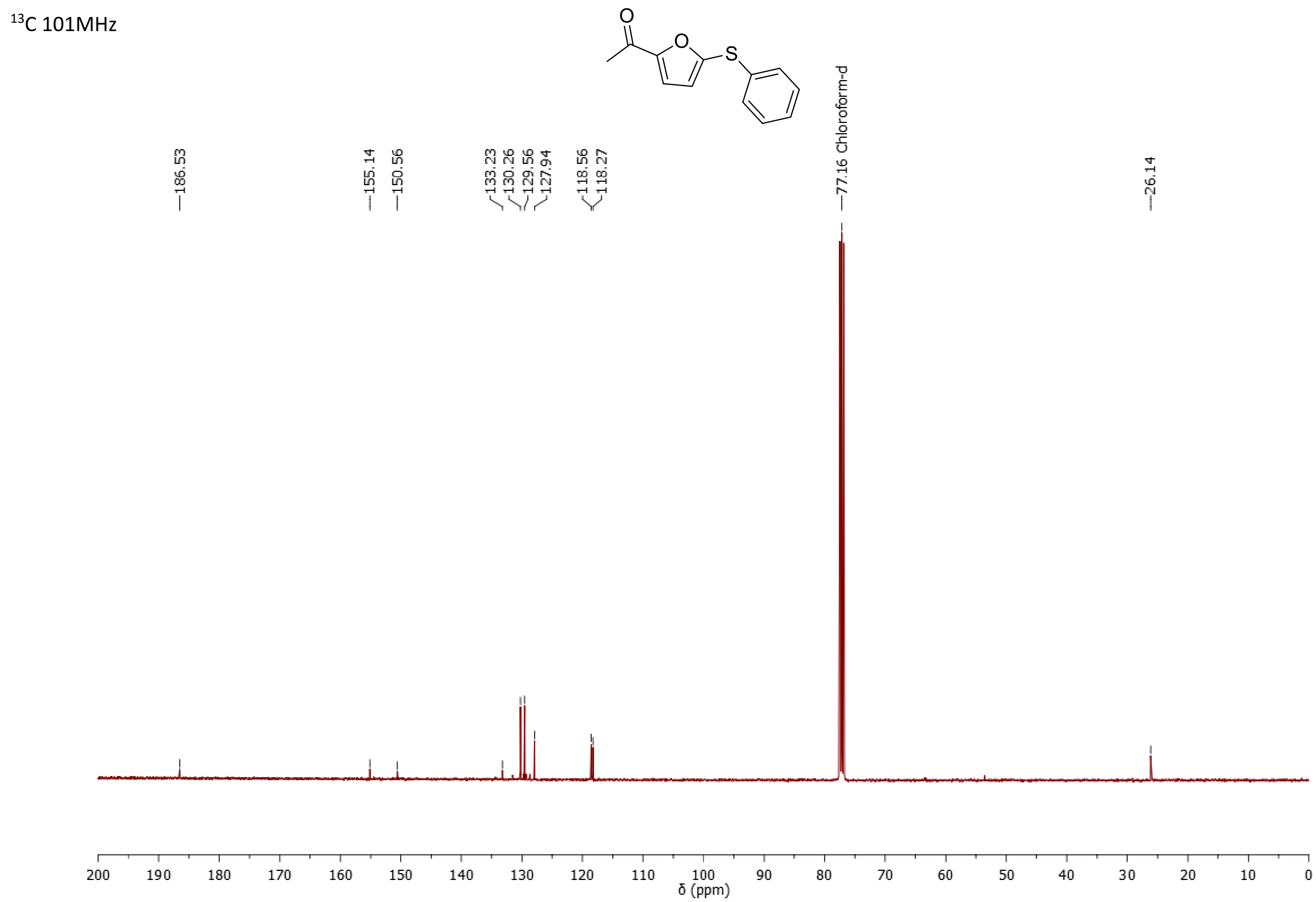

Supplement: Supplementary file 1 — ol1c00451_si_001.pdf [file ol1c00451_si_001.pdf]
